# Supplementary material for: Perceptions of pre-exposure prophylaxis among sexually active adolescent girls and young women in Zimbabwe–A qualitative study
Source: PLOS Glob Public Health. 2025 Dec 2;5(12):e0005396. doi: 10.1371/journal.pgph.0005396 (PMC12671731; doi:10.1371/journal.pgph.0005396)
Supplement: S1 Appendix — (DOCX) [file pgph.0005396.s001.docx]

S1 Appendix: Discussion guide with acted scenarios for focus group discussions.

**Opening statements:** Thank you for taking time to have this discussion with us. As you may remember, my name is , and my colleague here is . Today we will have a discussion on your views on PrEP (Pre-Exposure Prophylaxis) to understand PrEP acceptability, reasons for the low numbers of people who take up PrEP, and issues with how well it is taken amongst adolescent girls and young women. This will give us important information on how we can develop new ways of providing PrEP services to improve the number of Zimbabwean adolescent girls and young women who take it up and remain on it among those who need it and are willing to use it. Our discussion will take between one and a half to two hours to complete.

In this session we will have both role plays and discussions on the study topic. For role plays, we will ask you to split into three groups. Each group will act out a scene on how adolescent girls and young women would typically react to certain scenarios related to PrEP and perception of HIV risk related behaviours. Each group will be given a description of the scenario that they are supposed act out and will have only three minutes to agree on content of the acting scene. We will then ask the group to in turn act out their scene, which will be followed by a full group discussion that explores what has been acted out.

Before we start on our session, we would like to all agree on small rules that will help us have a fruitful discussion. *(****Moderator to ensure a participatory approach to setting the ground rules, which should be written on flip chart. Moderator to ensure that one of the rules is that FGD participants will only use participant IDs rather than true names when addressing each other).***

As we have already agreed, we will not refer to each other by name during the discussions, but by the numbers we have given you. Actresses will use the names that are supplied with the scripts/scenarios. The recording of our discussion will not be labelled by any of your names, but only by the number that we have assigned to this group. Do you have questions before we begin?

# Knowledge of PrEP

1. Have you ever heard about PrEP, if so, what is it?
   1. Where did you hear it from? Let us to go the scenarios.

# Acting out scenarios

- Please can you split into three groups. ***Each group is then given one of each of the scenarios below to discuss within three minutes before acting out begins.***

| **Chido and Koko**   - Chido is 16 years old. - She is having a sexual relationship with an older man who is aged around 50s. - She recently started taking PrEP as she is worried about contracting HIV. - She thinks her friend Koko who is 19 years old is also at risk of getting HIV from her sexual relationships and she suggests to Koko that she also takes up PrEP. - Do a role play of the discussion between Chido and Koko, covering what you think would happen in real life if Chido made this suggestion. | **Mai Bhobhi and Mai Juru**   - Mai Bhobhi is 23 years old and is married. Mai Juru is 21 years old and is married. - Mai Bhobhi’s husband has a habit of having girlfriends. - She is worried about getting infected with HIV. - She heard PrEP being advertised on radio and she went and got initiated on PrEP and it has been 6 months since she started using it. - However, she is now thinking of stopping the use of PrEP. - Do a role play of the discussion between mai Bhobhi and mai Juru, covering the reasons why mai Bhobhi   now wants to stop using PrEP. |
| --- | --- |

**Three friends: Peppa, Sky and Princess**

- They are all in their twenties and have sexual relations with their partners and they go to the same school.
- They have been selected to come up with a PrEP program for AGYW for an organisation named Ceshhar which focuses on sexual and reproductive health.
- They are preparing for their presentation, do a role play of their discussion covering the things they think should be part of the program.

Now let us have our discussion of the scenarios.

1. Let us consider the scenario between Chido and Koko.
   1. Are there really people like Chido who have sexual relationships with older men? Please explain.
   2. What do you think, is Chido at risk of getting HIV? Please explain.
   3. Is it common for adolescent girls and young women to share their health decision with other people?
      1. Who do young people typically share such information with? Who is typically asked to give advice? Does one ask only other close friends?

Please explain

- - 1. What are the other sources of information on PrEP?
  1. Where best can adolescent girls get information on PrEP?
  2. What do you think about Koko’s response when Chido suggested she take up PreP? Why do you think she responded this way? Is Koko’s response typical of what would happen in real life?
  3. What other responses would you expect someone like Koko to make in real life?
  4. What do you see as the benefits of PrEP for adolescent AGYW?
     1. What about for other young women like you?
  5. Are there any other barriers to the uptake of PrEP amongst AGYW that we have not discussed?
  6. Are there any other facilitators to the uptake of PrEP amongst AGYW we have not discussed?
  7. Where can adolescent girls and young women access PrEP services? What are your views on these locations? Please describe what works well and what does not work so well regarding these locations? Please list all potential venues and the advantages and disadvantages of each.

1. Let us consider Mai Bhobhi and Mai Juru’s scenario
   1. What do you think about this scenario – in real life are there married adolescent girls and young women who are in similar situations like Mai Bhobhi? Please explain
      1. Would it be easy for a woman like Mai Bhobhi to decide to start using PrEP? Please explain. *If not covered by discussion probe for barriers and facilitators to PrEP uptake specific to married AGYW*
   2. In real life does it happen that some AGYW might want to stop using PrEP along the way? Please explain. What are the reasons why AGYW may stop taking PrEP?
   3. In your opinion what do you think can be done to help AGYW who are on PrEP to keep using it?
2. Let us consider the third scenario, do you think AGYW can develop their own PrEP implementation intervention that suits them? Please explain
   1. What do you think about the program that the group presented? In what ways does it work well? In what ways does it not work well?
   2. What are the most important things that can make a PrEP program for AGYW successful? Please explain
   3. What are the things that make a PrEP program bad? Do current PrEP services meet the needs of AGYW? Please explain
   4. If not already mentioned,
      1. Probe for the following:
         1. Dispensing location
         2. Dispensing provider (is it a nurse, pharmacists)
         3. Dispensing frequency
         4. Provision of other services at facility providing PrEP (family planning, risk-reduction counselling, pregnancy testing)
3. What is the role of male partners in the uptake of PrEP services? Please explain
   1. Does role/influence depend on stability of relationship with partner?
   2. Do you feel you can easily access family planning services? Please explain
4. We are planning to conduct further research amongst sexually active AGYW to find out how we can further develop a new way of providing PrEP services that suits AGYW.
   1. Where best can we recruit AGYW who are sexually active?
   2. Do you think AGYW would come forward to join the study that is recruiting sexually active AGYW? Please explain. What can be done to make them feel able to join the study?
   3. As part of that research, we would like to test for some sexually transmitted diseases. This will include asking AGYW to take their own vaginal swabs that we will test for germs that are associated with sexually transmitted diseases. Do you think AGYW will be comfortable with self-collecting vaginal swabs for this purpose? Would the recruitment venues you described above still work well if we need vaginal samples collected? What are your suggestions on how we could make vaginal collection of samples for this study work best?
   4. We would provide results of the tests and facilitate treatment for those who are found to have germs that are associated with sexually transmitted diseases. Results need to be given by trained health workers. What are your recommendations for how we communicate that AGYW who test positive for germs that are associated with sexually transmitted diseases should visit a clinic/facility? What are your recommendations for ideal facilities/clinics where treatment can be offered?
5. Zimbabwe has recently approved the use of dapivirine, a vaginal ring which is a flexible, silicone ring that a woman can insert in the vagina for monthly protection against HIV. The ring is designed to provide women with a discreet and long-acting option for HIV prevention. It contains the anti-retroviral drug dapivirine, which is released slowly to reduce the risk of HIV infection locally in the vagina with few effects elsewhere in the body. What are your thoughts on long-acting formulations like this one in promoting uptake of PrEP by AGYW?
6. Do you have any questions or are there other things which are related to this topic that you would like to talk about?

We have come to the end our discussion, thank you for your time and contributions.
