# Supplementary material for: Perceptions of pre-exposure prophylaxis among sexually active adolescent girls and young women in Zimbabwe–A qualitative study
Source: PLOS Glob Public Health. 2025 Dec 2;5(12):e0005396. doi: 10.1371/journal.pgph.0005396 (PMC12671731; doi:10.1371/journal.pgph.0005396)
Supplement: S1 File — (ZIP) [file pgph.0005396.s003.zip › S1_File/AGYW-FGD 01-Translation.pdf]

<u>AGYW-FGD 01- Translation</u>
Facilitator: KC
Note Taker: RB
Date Of FGD: 10/12/2021
Translator: FM

KC: Right so as we start our discussion, thank you again for taking your time to discuss with us today. As you may remember I said my name is KudzaI and I am coming from CeSHHAR Zimbabwe. With me is Rufaro and we are both coming from CeSHHAR Zimbabwe, so as I have explained when I was giving that background, our discussion will be looking at the views about PrEP so that we get an understanding of what facilitates people or young women and girls to accept PrEP and also why the numbers are low or reduced among people who are using PrEP. Also, what other issues can adolescents' girls and young women experience. As I have said before, the reason we are seeking this information is so that we can come up with programs that are acceptable and used by young girls and women because a program can be made then it is not used because it will not be acceptable, it is not usable.

XXX: Uhh.

KC: So, the reason we are conducting this discussion is for you to help us with your knowledge so that we can come up with effective programs, so that we can come up with programs that young girls and women can use in future years. Aah as I have said that we shall have role plays and discussions so these ones we shall properly explain them to you when we get to that stage. We have set our ground rules the ones we have discussed there, so let us make sure that as we are doing our discussion, we just follow our ground rules for things to go smoothly. Aah the numbers we have given you is your name, do not say out your actual names during the discussion even when you are now doing role plays do not say your actual names, use those numbers. So firstly, my first question that I want to ask is, have you ever heard about PrEP?

ALL: Yes.

[Someone coughs]

KC: That's fine, most of you resp…responded that you have heard about PrEP. Can you explain to me what you heard, what is PrEP? The knowledge that you have, what is PrEP? Uhh number 4.

[Someone coughs]

04: It's a pill that prevents HIV.

KC: Uhh, thank you, others. Are there others who want to add to what PrEP is?

XXX: Haa what number 4 said is correct.

KC: What number 4 has said is correct, eeh number one.

01: Ehh it is taken before.

KC: It is taken before what?

01: Before the sex.

KC: Alright that is when you take this pill.

01: Uhh.

KC: Alright, uhh. What about how it is taken, what has been said that it is taken before, how long is it taken? Number 08.

08: It is taken for one month.

KC: One month.

08: Taking it every day.

KC: Then after one month, what happens?

08: You then decide if you want to take it again, if you want to take it or its either you want or you stop.

KC: Alright.

08: It will now be your own decision.

KC: If you still want to continue or stop. Okay, are there others who have something to add on PrEP, on what is PrEP, how is it taken? Are there others with a different view from what number 08 has said? Uuh number 01.

01: When taking them, you are supposed to take them the time you take your pills, if it is eight make sure you always take them at eight but if you fail to meet that time you can take it when you get the opportunity but you are not supposed to say if you fail to take it today, then you take two the following day, you just take one.

KC: Alright, thank you number one. Are there others with something to add? All this information that you have said about PrEP, where did you hear it from? Even those who did not say anything, the information that you hear about PrEP, where do you hear it from? Number 08.

08: I have used it before, that is where I know it from.

KC: You have used PrEP?

08: Uhh.

KC: Alright for you to use PrEP, where did you hear the information from?

08: It was a study.

KC: Alright.

08: It was sort of research; they wanted to check if it is effective.

KC: Alright.

08: If it is effective in to preventing HIV and AIDS.

KC: Alright.

08: That is how I heard about that information.

KC: So, for that study what was discovered about PrEP?

08: It is effective.

KC: It is effective.

08: Yes.

KC: Horaiti, okay.

KC: Alright, okay.

08: But it works if you are using it, if you are not using it, it does not work.

KC: Alright.

08: If you are taking the pills, it works but if you are someone who takes them today then you do not the following day, forgetting them.

KC: It does not work.

08: Uhh there will be so many high risks that you can contract it.

KC: Okay, so how long did you use it?

08: Six months.

KC: Continuous six months, alright thank you. Others this knowledge on PrEP, number five.

05: I… I just heard about it at the clinic.

KC: Uhh.

05: It is an issue that was talked about when we were at the clinic.

KC: Okay.

05: It was being told to girls and young women that there is treatment that is there to prevent one from contracting HIV.

KC: Alright, which clinic is it?

05: XXX

KC: XXX, alright it's okay so it was giving… was it information that was being given to the youths, it was being given to… women who are pregnant? What kind of people was it being given to?

05: It was being given to the girls who were receiving treatment.

KC: Ooh they were receiving treatment at that time, alright. Thank you. Are there others who want to add information on PrEP. Others said they heard about it at the clinic, others said at a study that was conducted, are there others with something to add?

[Silence]

KC: Alright it's okay, if there is no one with something to add, we are now taking a break to go to our role plays that we have talked about, right. So those role plays that I have

talked about have… there are three role plays, I am going to read them, but I am going to give each group their paper that clearly explains your role play, right. So, our first role play is about Chipo and Koko right that is their names.

Scenario 1 Chido and Koko

Chido is 16 years old. She is having a sexual relationship with an older man who is aged around 50s. She recently started taking PrEP as she is worried about contracting HIV. She thinks her friend Koko who is 19 years old is also at risk of getting HIV from her sexual relationships and she suggests to Koko that she also takes up PrEP. Do a role play of the discussion between Chido and Koko, covering what you think would happen in real life if Chido made this suggestion.

KC: So, our role play of Chido and Koko, they are two friends, one has already started to take PrEP because she thinks she has a chance of contracting HIV. She thinks that her friend also has the chance of contracting HIV so should take PrEP, that is why she is talking to her about PrEP so the discussion or the role play is what we want to do… showing Chido telling Koko about PrEP. We want to hear what Koko's response will be, what she thinks about the PrEP that is being talked about. The second role play, our second role play has Mrs Bhobhi and Mrs Juru.

Scenario 2 Mai Bhobhi and Mai Juru

Mrs Bhobhi is 23 years old and is married. Mrs Juru is 21 years old and is married. Mrs Bhobhi's husband has a habit of having girlfriends. She is worried about getting infected with HIV and this is Mrs Bhobhi. She heard PrEP being advertised on radio and she went and got initiated on PrEP and it has been 6 months since she started using it. However, she is now thinking of stopping the use of PrEP. Do a role play of the discussion between Mrs Bhobhi and Mrs Juru, covering the reasons why Mrs Bhobhi now wants to stop using PrEP.

KC: Right, so this is the second one of Mrs Bhobhi, so we want to hear the reasons why Mrs Bhobhi may want to stop using PrEP. What you think may be the reasons. The third one is about two friends again; sorry I took the wrong paper. It's about two friends, aah sorry they are three friends. These friends are Peppa, Sky and Princess.

Scenario 3-Peppa, Sky and Princess

They are all in their twenties, twenty-twenty something, they are just like you right. They have sexual relations with their partners, and they go to the same school. They have been selected to come up with a PrEP program for adolescent girls and young women for an organisation named Ceshhar which focuses on sexual and reproductive health. They are preparing for their presentation, do a role play of their discussion covering the things they think should be part of the program.

KC: So, these are three friends who are still discussing that we have been chosen to come up with a PrEP program representing their school, this is needed at CeSHHAR right. So, you are coming up with a program highlighting that the program is supposed to be like this, with this and that so that is role play number three. So, we are now pausing our recorder briefly so that you can have five minutes to discuss your role plays. After five minutes we will come back and continue, so when we are now continuing number one will do their own then we ask some questions. Number two will do then we ask questions, number three will do then we ask questions then we finish, right. So, I am now pausing our recording.

[Recording paused; however, it stopped and saved the interview which resulted in having two audio recordings for this discussion. There is need to check if the settings of the recorder can be adjusted to increase the time allowance for pausing a recording]

KC: We are now continuing where we had left off, so we are now continuing looking at our role plays. Role play for Chido and Koko can start, so Chido and Koko do your role play for us then we will ask questions. We will ask questions after watching the role play. Come and speak loudly so that our recorder captures everything.

08: Okay so our role play what we have done is, we have given each other characters as in who is going be Chido and who is going be Koko, so number one is Koko, then I am Chido.

KC: It is okay.

08: So, I am Chido and I am sixteen years old, I am having sex with my blesser who is 50 years and above. So, I think that I am at risk of what, of contracting HIV so I decided to go and take PrEP. I have taken PrEP then my friend who is called Koko, I mean so… we

are friends, so I already know that she also has a blesser. So as a friend I will say let me go and tell my friend and help her that I have pills that I am taking.

KC: Uhh.

08: They can help you to prevent what, contracting HIV.

KC: Uhh.

08: So, don't you want to take them as well, so she is the one who is going to give me the response.

KC: Alright so now do the role play approaching her telling her your issue. So that we hear how Koko will respond.

08: Okay.

KC: Uhh.

Role play 01

C: Hi Koko, how are you?

K: I am fine Chido, how are you?

C: I am fine.

KC: Raise your voices so that the recorder can capture.

C: So, I have my little issue which has been troubling me, so I decided to come and tell you as my friend what is happening. Isn't you know that I have a blesser who is… who is a bit older in age? You never know what these blessers will be doing aside.

K: Yes I know.

C: So, there is a clinic I went to and I heard that they give pills that prevent the risk of contracting HIV & AIDS, so I am now taking these pills. They give you these pills and you take them every month. You decide the time that you would like to what, to take them since you are my friend and you tell me the stories of the person that you have sex with I

thought maybe it would help you if you what, also take the pills and be protected from HIV.

K: *You said they are taken every day?*

C: *Yes, they are taken every day.*

K: *But it will now be as if you are taking ARVs, every day.*

C: *It is better because you will be preventing HIV. What is better, not to take pills and contract HIV or to take the pills and not contract HIV.*

K: *Taking the pills is better but everyday… alright where do you get them from?*

C: *I get the from the CeSHHAR clinic at DIC or Mbare depending with where you want to go.*

K: *Aren't they nearby clinics that give the PrEP?*

C: *Aah they are the only ones that I know of, I do not know if there are others.*

K: *If I decide we will go together but aah everyday is too much because I heard that the way the pill is so big.*

C: *[Chuckles] Being big is not an issue you just take it… it is right to prevent than to refuse to then you contract the disease, you just take. You just take it after eating, you just take it at night when you are about to sleep so that you won't be affected by them or how big they are.*

K: *Aah since you are already taking them we shall just go together and get them.*

C: *Aah alright.*

[Other group members clapping hands]

KC: Thank you Chido and Koko so what we are now going to do are the questions looking at the role play that was done by Chido and Koko, right. Ahh then we will proceed to the other role plays, moving like that with our discussion moving like that. So, we are going to look at the role play of Chido and Koko, how was it? Did it bring out what happens in real life? Uhh what number?

07: No that is correct.

KC: What is your number?

07: It has fallen.

[People laugh]

07: Number 07.

KC: 07 Alright.

07: Haa that is what really happens, when you tell someone, they first refuse but it depends on the way you would have told them, how you would have talked to them and the kind of friendship you have.

KC: Alright, uuh. Others how did you view it? Number 05.

05: What mostly happens is that most people do not understand about PrEP so people will have questions like the questions that Koko had that it is big. Plus, a person will be confused that you will now be like someone with HIV because you are now taking them every day.

KC: Alright.

05: So, this is what really happens in real life.

KC: Aah alright it's okay, aah looking at this role play right. Are there people who are like Chido who have sexual relationships with older people or these blessers? Are there people like that?

06: Yes there are there.

KC: What is your number again, it has fallen.

06: Number 06.

KC: Number 06, number 06 you said there are there. What kind of relationships do they have, are you able to explain how this will be happening?

06: It differs someone will be having their person… older while they are young.

KC: Older like how, give us an example.

06: Or even around 45.

KC: Okay, you…

06: You are in your twenties. Because they want, girls want money.

KC: Alright, alright that is what pushes them.

06: Yea.

KC: Okay, 08.

08: Yea it happens and most of our generation that is what is now happening.

KC: Alright.

08: Aah yes these girls have their boyfriends but there will be someone who will be blessing (spoiling) them because you find out that maybe their boyfriend… saying my boyfriend is not doing this for me. So, she will be saying if I have a relationship with this older man, he will do everything for me, he will be giving me money.

KC: When you say doing everything, what is it? Everything…

08: As in all the needs that are needed by the girl.

KC: What are the girls' needs?

XXX: Like…

KC: Yes, list the things?

[Participants talking at the same time]

XXX: Like being bought for clothes.

XXX: Clothes

XXX: Being taken out some people want that.

08: Or having someone paying for your nails, some people just want that.

XXX: Or even a phone.

KC: Uhh, 05.

05: In my own view, I think blessers are now common.

KC: Uhh.

05: Blessers are common among young girls.

KC: Uhh.

05: It’s very… its now rare to see a girl without a blesser because boyfriend yes they have. He is for walking around with being seen by your friends that you have a boyfriend a handsome one but meeting needs most boyfriend are not meeting needs. Needs are being met by blessers.

KC: By blessers.

05: I have a phone that I want, then the blesser blesses you with it.

KC: Okay, yes 03 you were saying something about needs.

03: I was saying some of us want to be sent to school.

KC: Alright.

03: Maybe I have my child who needs to go to school, who doesn’t have a father who needs to be looked after, he will be helping me to look after them.

KC: Looking after the children. Alright okay, aah looking at this story of Chido, do you think Chido is at risk of contracting HIV?

05: Uhh yes.

KC: Uhh number 06.

06: If… if she is using PrEP, using it well she may not contract it.

KC: Alright but let’s say… let’s say without including PrEP looking at the fact that she has a sexual relationship with an older man.

08: Haaa….

KC: Is she at risk of contracting HIV?

08: Straightforward.

KC: 05.

[Many mumbling]

KC: 05.

05: She is at a very high risk because this blesser has a wife. Chido also has a boyfriend or boyfriends.

KC: Alright, uhh.

05: So, the chances, the chain is big.

KC: It is big.

05: Uhh.

08: I want to add up.

KC: Uhh.

08: Also, what blessers do… [Interjection]

XXX: They do not use condoms.

08: You are not the only one who will be blessed.

KC: Alright.

08: You are blessed as the five of you sometimes, you will all be blessed.

KC: When you are in one place?

08: At the same position, he knows that when I leave here I shall see Chido, I shall see XXX, I shall see XXX, I shall see this one, I shall see this one, you will be many.

KC: Alright.

08: Also, you will be in a place… plus like in most cases like what we hear some girls saying, they are evil those people.

KC: The blessers?

08: Yes, because they can spite you by transmitting the disease when you are unaware.

KC: Alright.

08: Now the girls, the problem that the girls have is that when money is flashed for them, when they are just given a mere hundred dollars they forget about their health.

05: You do not even ask the condom.

08: They will now be saying whatever happens.

KC: Alright because they would have been blessed by what makes them happy.

08: You end up sick, just imagine if he has five people that he blesses. If he has sex with all these five without protection, all of you will contract it.

KC: Are the chances big?

08: Because most of these people are already sick, that is the truth.

KC: Ooh they are already… aah no what will they be sick of? Diabetes or Bp?

XXX: AIDS!

KC: With HIV.

XXX: Yes.

08: Haa they will be already sick.

KC: Alright, uuh, okay. Continuing does it usually happen that young girls or young women share their decisions on issues to do with health. Or discuss with other people issues about their health. Does this usually happen, you are the young girls and young women? Do you see that happening, do you do that sharing with other people health issues, discussing them? This side wait a bit let's go to this side, so that we change sides, number 01.

01: Aah when it comes to the issue of health, it is rare for people to share knowledge but when they don't have and it is the issue on PrEP they can share but when they have contracted it now to share aah… it will be spread around.

KC: Alright, you said it will be spread around, so will they be afraid that it will spread around when they talk to someone about health issues?

01: Yes, they will be afraid that it will spread around that is when discrimination might occur. After telling someone you will now hear people saying, do you know her status.

KC: Alright.

01: It comes out.

KC: Alright are there others with something to add, which ages are these… yes number 07.

07: Also, when you have said it even when you have not told anyone that I am HIV positive they can… when you start talking about HIV they may start to suspect. That thing that… they may… the rumour may spread without you having told them.

XXX: Haa true.

KC: Ooh, right just discussing HIV issues one may start to think that I am positive.

XXX: Yes.

07: Yes, that how did you think about it, to talk about it.

KC: Ooh, alright.

07: Yes so…

KC: Alright, 08.

08: Also, I think discussing health issues depends with the relationship between two people.

KC: Alright.

08: It is just like I have my friend that I say I am close to. I really know that even when I am told I have an STI today, I tell no one else except her.

KC: Alright.

08: She is the first one that I will tell that things are not alright; this is what I have done.

KC: Uhh.

08: So, it depends with the relationship, how you relate.

KC: Uhh.

08: You cannot just say from nowhere when you see someone from the neighbourhood you tell them your secrets.

KC: Alright but is it possible to talk about the issues…

08: It depends.

KC: It depends with who you are telling, I do not talk with just everyone.

08: It is not possible when you just come across someone in the neighbourhood, they will say this woman is crazy.

KC: Hmmm, alright, okay, okay. Aah what about when we look at… we have talked about it, we have talked about it though in the beginning when we asked where you are getting this information about PrEP from, where did you get it from right.

08: Uhh.

KC: You said the clinic, you said from a study that was researching on PrEP, right.

08: Uhh.

KC: Where else do you know of where such information about PrEP can be obtained? If someone goes to those places they will get information about PrEP? Just general not considering where young girls can get them from. Just general where an older person or young person can get the information on PrEP at these places, where you know. Number 05.

05: Maybe for someone who likes reading if they check on the internet.

KC: On the internet.

05: But from what I know mostly its from the clinics.

KC: In clinics.

05: Because you have found groups that will be talking about it.

KC: Alright. Number 04.

04: Yes if you go to the bars and meet people like us empowerment workers we can meet you and tell you about PrEP.

KC: Alright.

04: Then you come to the clinic and get help.

KC: Alright, aah this point that you have said about empowerment workers, what will they be doing at the bars, empowerment workers?

04: In bars we will be looking for sex workers, the ones we would like to come and get PrEP if they are negative.

KC: Okay. Alright, alright.

O4: Uhh.

KC: So, they will be sort of PrEP champions?

04: Yes.

KC: Encouraging about PrEP, alright. Aah you have answered the point I wanted to ask next but I just want to add there. You said young girls who are still growing up can find empowerment workers who will be moving around in the bars. Is there anywhere else young girls or adolescent girls and young women can get information about PrEP, is there anywhere else you know of or where you think?

08: It depends the things can just happen like you are conducting a study like this.

KC: Uhh.

08: There are some who move around in… places like you can… people can say let us go to XXX we have our study and inform people who are there about this thing called PrEP.

KC: Alright.

08: That is how people will be learning.

KC: How people will be, alright.

08: Yes.

KC: Alright, it's okay moving forward right we now want to look at the response that Koko gave when Chido talked about her taking PrEP, right. Why do you think Koko responded the way she did, we all witnessed how Koko responded right?

XXX: Uhh.

KC: The way she responded was like someone who was not very interested in PrEP. Why do you think she responded that way? Looking at what happens in everyday life, things that happen every day, her responses, and answers.

08: Aah for me I think it's very weird.

KC: Uhh.

08: You may say this person is telling me about PrEP that prevents HIV is it because they see me as a sex worker.

KC: Alright.

08: Like I have sex with a lot of men.

KC: Uhh.

08: Or I love sex so much, do I just have sex with men all the time.

KC: Alright.

08: That is how a person views it, then what is also being said that it is big. She would have heard the information; the pill is big for sure.

KC: Alright.

08: They will be thinking that with the way the pill is big, haa will I be able… because others who would have taken it may be telling you the symptoms that if you take it you will feel powerless. You… you start feeling weird, others start to gain weight so one will now be thinking that aah I will start looking like a sick person.

KC: Alright.

08: Haa so let me just leave it so they will respond in a cold-shouldered way.

KC: Uhh. Are there others with something to add to the scenario that just happened, role play that has been done, right. Do you have other responses that might happen on what have been done by Chido and Koko, there are some responses that someone like Koko can answer that did not come out in the role play that has just been done? Is there anything else that can be said by someone who would have been told to say my friend why don’t you join the PrEP program? Being the first time to hear about it that did not come out in this role play, are there other responses? Number 05

05: Some people are… I can say they are blunt; they just tell you openly that I am not interested.

KC: I do not want.

05: I do not want.

KC: That is another response that aah what you have told me, I do not want. Are there other responses? [Silence]. There isn’t.

08: Or to scold you.

[Cell phone ringing]

XXX: Another may tell you that I shall… I want to try.

KC: Okay, when you are looking at it what do see as the advantages of PrEP to adolescent girls and young women? What are the advantages in your own view? Number 05.

05: For someone who will still be growing.

KC: Uhh.

05: Who still wants to enjoy.

KC: Uhh.

05: When we are enjoying we meet a lot of different people.

KC: Uhh.

05: It also puts us at great risk of contracting the disease so if you are someone who is taking PrEP you can't… the chances are greatly reduced and when you decide to get married you rest with good health.

KC: Alright. Others the advantages of taking PrEP to young girls or young women that did not come out, that Chido did not say out in the role play. What are the other advantages of PrEP?

XXX: I am just encouraging that PrEP should be used continuously even if you use condoms because others say when they are using condoms, I do not have the need for PrEP, condoms can burst.

KC: Alright they give protection when condoms burst. Uhh anything else? [Silence]. Alright aah what about the barriers, we were talking about the advantages… advantages of PrEP, what can make someone want to take PrEP. Is there anything that can cause PrEP not to be taken among adolescent girls that we have not talked about? It was mentioned that the pill is big, side effects were mentioned that others would have heard about side effects, is there anything else? From discussions, number 01.

01: When we look at the girls who are still living with their mothers for you to be seen holding the bottles of pills they may think that my child is now sick. To tell them that it's PrEP that is used for this you will have to tell them that it prevents HIV then they will say are you going to the bar so it discourages them to take what…

KC: PrEP.

01: The PrEP.

KC: Alright, uhh are there others? Uhh number 04.

04: Others will be young women with husbands.

KC: Uhh.

04: Then take PrEP it will bring disputes in the house that why are you taking PrEP.

KC: Why are you taking it, then they start fighting in that house. Alright, number 05.

05: Aah I heard that PrEP is taken the same time.

KC: Uhh.

05: When you said eight, you stick to eight so that may cause someone to fail to adhere to that time that every day at eight you know I have to take PrEP. The issue of time again may cause someone not to take PrEP.

KC: Aah alright, okay. Alright moving forward aah where can young girls and young women get PrEP programs? Let's say someone wants PrEP where can they get them, the services where can they get them?

[People talking in the background]

08: I heard about it, not so sure if it's true.

KC: Uhh.

08: Someone told me that it is sold in pharmacies.

KC: It is sold in a pharmacy, uhh.

08: You can go and buy it in a pharmacy.

KC: Uhh.

08: I think even if you go to other health facilities.

KC: Alright.

08: Just a general facility you can get them.

KC: Pharmacy, general clinic, any other places where PrEP can be obtained if someone wants it? [Silence] Alright let us look at two places that have been mentioned. At the pharmacy when someone wants to take it at the pharmacy, let's say PrEP is being found at the pharmacy, what do you think about obtaining PrEP from the pharmacy? The advantages, the disadvantages of obtaining PrEP from the pharmacy. Number 05.

05: Obtaining PrEP from the pharmacy has the advantage that you will be alone, confidentiality. You go at the counter and buy your PrEP and come back home.

KC: Alright.

05: But you are supposed to have money to be able to buy it.

[Someone coughs]

KC: Alright so you have brought out two points.

05: It becomes a disadvantage.

KC: Its advantage, confidentiality. Disadvantage is that money will be required to get it from the pharmacy. Anything else about the pharmacy that you want to talk about? [Silence] Alright, what about when we are looking at the clinic, the general clinic. Getting PrEP from the general clinic, is that feasible, is that not feasible. Then give advantages and disadvantages, number 04.

04: Obtaining them from the clinic is good, sometimes you may be given for free, you do not use money, but the disadvantage is if I go… or if number 05 arrives and sees me taking PrEP she can move around saying aah she was getting ARVs.

KC: Alright, alright you have brought out two. The advantage and disadvantage of it, someone else. At the clinic, general clinic, number 01.

01: Aah if they are local clinics and you stay there, if you get PrEP before you get to your home your mother would have been told about it by the nurses from there.

KC: Alright.

08: You will find it all over.

KC: Alright, she was here getting PrEP, your child, we prescribed it for her.

05: We gave her.

KC: Okay, alright. What about clinic and pharmacy, are there any other places that we may want to discuss about, their advantages and disadvantages besides clinic and pharmacy? [Silence] What about PrEP that you are given in the community, maybe there is a community health worker in the community who gives young girls PrEP, would you want it? Advantages and disadvantages.

06: Haa this one is not feasible.

KC: Uhh why?

06: Haa you will be talked about.

KC: The issue of confidentiality will still be there.

08: Haa she now has AIDS.

06: Or she was in the bar and is now coming to take PrEP.

XXX: Telling your mother.

[Participants mumbling]

KC: Alright, so the community one is not feasible.

05: It is not feasible.

KC: It is not feasible, alright we are now moving on to role play number two, right. Number two is the one of Mrs Bhobhi and Mrs Juru so number two, group number two come and do the role play then we do our questions. Then our discussion will move like that.

[Group two members moving to the front]

KC: Do not forget to speak loudly when you are speaking.

XXX: Let us say Mrs Bhobhi, who is Mrs Bhobhi [chuckles]. You are Mrs Bhobhi?

XXX: It's you?

XXX: I am Mrs Bhobhi [Mumbling]

KC: So just to serve as a reminder, the one for Mrs Bhobhi, that is the one we said Mrs Bhobhi and Mrs Juru are… best friends and both married. Mrs Bhobhi's husband has girlfriends, right and she was concerned that she might contract HIV because of… that reason. So, when she heard about PrEP, she now has six months taking PrEP but she is now thinking of stopping the PrEP, we don't know what happened to her. So that is where your role play is focused at, we have reached the point that Mrs Bhobhi wants to stop taking PrEP, we now want to hear how the discussion goes. Including the reasons why Mrs Bhobhi now wants to stop taking PrEP, Mrs Bhobhi saying out what she thinks.

03: I think Mrs Bhobhi wants to stop taking PrEP because maybe it is causing side effects.

KC: Alright so for it to come out well, do a small role play. The three of you like what Chido and Koko did but two people are going to do it. So, we said it's Mrs Bhobhi and Mrs Juru right.

03: Uhh.

KC: So maybe Mrs Bhobhi can start by approaching Mrs Juru saying I was on PrEP but this is what happened. I don't want it anymore because of these reasons that is what we want for the points to come out as you do the role play.

03: Uhh.

[Someone coughs]

Role play 2

MB: Mrs Juru how are you my friend?

MJ: Are you well?

MB: I am fine, aah I have come, that PrEP... I have been taking it for a while now but haa I am feeling something in my body. I am just feeling this body weakness, bored by it. Aah I am now thinking of stopping this PrEP it is boring me, does it even really work, this PrEP.

[Papers being flipped]

MJ: Aah do not stop taking PrEP my friend, what is happening with this PrEP?

MB: Aah I am just feeling this body weakness. It is boring me, haa it's making me uncomfortable. Right now, I know I am supposed to take it, I am now like someone who has HIV. Haa the things are… it is now boring me.

MJ: Are you taking it everyday my friend, are you taking them on time my friend?

MB: I am taking on time, sometimes I forget but I don't know what could have made me forget. It would have just happened for me to forget but most of the times I normally take it on time. But haa does this PrEP really work.

MJ: This PrEP works my friend, why don't you go to the clinic and find out what will be really happening.

MB: Yes at the clinic they will just be saying it works but where will I reach taking PrEP because of my husband's situation, I am now acting like someone on treatment.

MJ: Be strong my friend, be strong.

[Other group members clapping hands]

KC: Aah thank you, you can sit down. Looking at the role play of Mrs Bhobhi and Mrs Juru right, what do you think about the role play? In our everyday lives, are there married young girls or young women who are experiencing what Mrs Bhobhi is experiencing. Let us start there, are there young girls who are in Mrs Bhobhi's situation who are married

07: People like that are there but what is now happening is that it now depends on the friend that you socialize with. When they… who you would have told that PrEP is causing some reactions in me, what and stuff. Eeh how much they can encourage you; one may say it is your choice if you want to stop, stop. It now depends on how much they are encouraging you to be able to continue to…

KC: Using. Uhh alright. Others, do you think there are young girls who are married and are in these situations like Mrs Bhobhi's? Uhh number 01.

01: Yes, there are many, eeh there some young girls who would have eloped to a man who already has a wife, right.

KC: Uhh.

01: So, it's obvious that man will be unfaithful, they take this one, they take this one sometimes where he goes to the bars, so the risk that the young wife will contract the virus will be high.

KC: Alright, okay. Will it be easy to a person… to the wife like Mrs Bhobhi, a young married wife but has a husband who has other girlfriends. Will it be easy for them to want, to reach a decision of saying aah let me start to take PrEP, will that be an easy decision? Number 03.

03: It is a difficult thing, but you will not be having a solution. You would have seen that it is the only way, that is the one that can make me survive because if you listen to your husband, you will be put on risk.

KC: It is hard, but they won't be having an option, number 05.

05: I see it as a hard decision and most young women fail to take it.

KC: Alright.

05: Because when you are a married woman, these pills will be seen in the house, one may fail to understand that it's for preventing HIV. When they see them for the first time… they will have their own opinion that maybe you have the disease.

KC: Alright.

05: It starts causing violence so the decision is a difficult decision.

KC: What can encourage young married women to use PrEP? Married women, what can encourage them to use PrEP, in general from what you know or what you think? Uhh number 05.

05: In my own view, just like the way it was being encouraged to use family planning where they were told at the health facility.

KC: Uhh.

05: Going to the be weighed, these are things that are just taught that let's use family planning.

KC: Uhh.

05: PrEP should just be taught like that, that we have PrEP which works like this, then someone chooses on their own whether they want it or not.

KC: Alright. Moving on to our next question right, aah this role play, do you think it happens that a young woman who would have started to use PrEP can decide not to use the PrEP anymore. When they have taken it for complete six months then they just say I am stopping taking PrEP, does that happen?

XXX: Uhh.

KC: Number 08.

08: Yes it happens.

KC: It happens.

08: I probably think that it will now be their own decision, it will not be a decision or let's say… let's say symptoms.

KC: Uhh.

08: Because when you start to take PrEP, some do not have symptoms others have symptoms. You may feel it for the one month that you would have started, feeling unstable so after six months you will not be feeling it. It will be her decision that she would have taken.

KC: Ooh.

08: Maybe it will now be causing problems in her house then she says iih…

KC: Let me stop.

0: Let me stop.

KC: Alright, uuh. She just said that it does happen, it's a decision made because there will now be problems in the home. Is there anything else that… that causes a young married woman who has started PrEP? Number 07.

07: It is just like let's say you will be in marriage; you have been married.

KC: Uhh.

07: So, let's say your husband has seen them, the other thing that happens is that, like one can get married.

KC: Uhh.

07: When her parents are dead, you know that some people get married because of the situation.

KC: Uhh.

07: So, then they decide to take this PrEP because my husband is doing this and that. Then the husband will say for you to stay here stop what…

KC: PrEP.

07: So, you will stop because you know you have nowhere else to go, the situation that I am in is the one now causing me to just stop.

KC: To stop.

07: Because I want to stay.

KC: Alright, uhh. Others number 02, what do we think, the reasons why young married women and girls stop using PrEP? Stop using after six months or several months when they started using it.

02: Uhmmm….

KC: Uhh, number 01.

01: Uhh sometimes when you have gone to a family function, right.

KC: Uhh.

01: When you have carried it, maybe you would have said you will take them at ten. Then you are at a function, and you are seen taking pills and swallowing. Aah they will say this one's wife; did you see her. Look at her around this certain time, look around ten she will be swallowing her pills, she must have the disease. So, you may decide to stop and just say aah…

KC: Let me just stop.

01: Let me just stop.

KC: Uhh number 04.

04: Aah or to just relax and see as if your husband is now alright.

KC: Alright, he has changed his behaviour.

04: Then you decide to stop and say it seems to be well these days.

KC: [Chuckles] That he is now correct, he is now alright, no one will be fighting with anyone.

04: Uhh.

KC: Alright. In your own opinion what do you think can be done to help adolescent girls and young women who are using PrEP to continue using it. So, they do not stop when they have started using PrEP, what can be done? Number 08

08: Aah personally I think based on situations of thinking about what other people may say when they see me taking PrEP, what will they say.

KC: Uhh.

08: Just be confident to know that I… I am doing this for my health.

KC: It is mine.

08: It doesn't matter if they say that I am sick.

KC: Uhh.

08: If you don't take the PrEP you should know that they shall laugh about your disease.

05: Then you will take ARVs.

KC: Alright.

08: You are now sick from HIV so it's better to just take them, when they will be saying this one is taking them but you will be knowing that you are protected because people are difficult.

KC: Uhh.

08: When you are doing good, you are said to be wrong, when you do wrong there is nothing that you do that is considered as right so you are supposed to be a focused person. When you have made the decision to take PrEP.

KC: Alright.

08: Just make the decision despite what people will say.

KC: What will they say about it, alright. Is there anything else that can be done, let's say maybe at the clinic or by the government or health related that can encourage adolescent girls and young women to remain on PrEP? That can help where there is… this one said the individual themselves should know what is important to them health wise, right. Is there anything else that can be done to help this person, number 01.

01: Aah with education it may work, people should be educated about the advantages of PrEP. Even at school to the adolescents who are in Form one or Grade seven, they should be taught about PrEP so that by the time you get into the world you will be aware. Even the boys will be aware that there is something called PrEP.

Kc: Alright, uhh. Anyone with something to add? Number 04

04: Yes, it is just like us we have self-help groups, we are supposed to talk about such issues whenever we meet. When we meet, we tell each other about PrEP so that we may continue to encourage each other.

KC: Alright.

04: So that no one will default.

KC: Alright, uhh. Moving forward right, we now want to move to role play number three which is our last role play right on what we were doing. So those who were doing role play number three about three girls in school who have been given the task to come up with a PrEP program for adolescent girls and you women. Making it for the organisation called CeSHHAR, can they come and do their role play coming up with the program. Bringing out what should be in the program, what they think are the advantages, going like that.

[Group 3 going to the front to act out scenario 3]

05: Isusu tiri…tiri shamwaro three Peppa, Sky naPrincess saka inini ndini ndichaita Peppa.

KC: Uhh.

XXX: Sky.

05: Sky and Princess.

KC: Alright.

05: We have been asked to come up with things that we think should be included in the program that we want to make for CeSHHAR on PrEP.

KC: Uhh.

Role Play 3

Peppa: So aah girls this program that we have been given I think the people we want to work with, young girls and women so we want to… we have been asked to go and teach them about PrEP. I don't know what the others think we should include in this program. I was thinking that… when we get into the field to work with people, a young person who is in her twenties should also be there.

Sky: Uhh.

Peppa: Such that she can be acceptable to the community that she is going into since she will be meeting with young people. It makes it easy when she is being asked questions. I don't know what your thoughts are.

Sky: I was thinking, let's say older women that we are going to work with. It now depends with the person going to work with them if you really understand what you are talking about so that when you are explaining to another person they are left with an understanding because if it is young women they may just say aah what can you young people tell us. We were born a long time ago so it can take time to talk to older women for you to reach an understanding so what you have said about moving together with young women, I think it is alright because you can understand each other.

Peppa: Uhh.

Sky: You will be the same age group.

Peppa: So, it means the person should be well educated about PrEP.

Sky: Uhh, with good understanding so that when a person asks, you will not face difficulties answering because you are the one who went with the issue. You are supposed to explain

well to people. So that they know and really see that haa I have accepted that truly these things can help me in my life.

Peppa: I don't know Princess what do you think?

Princ: Yea I think it is right and to go and explain to someone you need to have… fliers or something that shows there are people who were assisted by PrEP. So that they can be able to partake of the PrEP. So they can be able to use it.

Peppa: Yea I think the issue of fliers that you are talking about is right and the issue that one may find it difficult for you to talk to them in the presence of other people.

Princ: Uhh.

Peppa: But if you give them a flier to read, if they are addresses of places they can get PrEP they choose on their own and go.

Princ: Uhh.

[Slight clapping of hands]

KC: Thank you group number three, so we now want to look at our group. The group had the task to come up with the PrEP program, a PrEP program that is being made is for adolescent girls and young women, right.

XXX: Uhh.

KC: So, we now need different points right. Looking at this role play do you think young girls and women can come up with their own program, on their own that is ideal for them for PrEP. Coming up with the PrEP program on their own, if given this task can they come up with their program without help from someone else. Number 05.

05: Personally, I think it is a difficult task because in the communities that we are going we are meeting with young women and girls. What have been said before that young women when you start telling them about PrEP their mind will start by wondering what you have seen on them. So, it is a difficult task to see that… but it is possible.

KC: Alright, thank you number 05. Alright for now let us focus on coming up with the PrEP program that works for young girls and women before you go with the program into the

community. While we are coming up with it, let's say the way we are in here we have been asked to come up with PrEP program. Are you able to come up with this program as girls without the presence of other people? Without health workers, without the Ministry, without CeSHHAR, in the absence of anyone else, number 07.

07: We may fail because there are things that we are not aware of.

KC: Alright.

07: What we would need your help with.

KC: Ok.

07: You have experience, you now know more.

KC: Okay, uhh. Others? Uhh number 08.

08: I wanted to say that we may fail to come up with it because we would need advice from health facilitators.

KC: Alright.

08: Because when I am telling someone that PrEP, she is going to ask me if I have used it, what is it used for. Maybe we want to come up with the program but I am not knowledgeable…

KC: Right.

08: So, people would want what is approved by the health people if it is feasible or not.

KC: Aah the… the way you are saying it on your own that coming up with a program that you want is difficult, but you need support from different people in the health sector about PrEP.

08: Uhh.

KC: Alright I get it. What about the program that was made, they said the program should have a young person, right with adequate information about it so that when they are asked they can answer?

XXX: Uhh.

KC: What do you think about the program that has been made by Peppa and Princess? You have seen the program that they made, what do you think about it, say out what you think about it. Was anything left out, should something be added? Number 07.

07: I think that it is good eeh that there be fliers as was said that time.

KC: Uhh.

07: It makes it easy for you because when someone likes it you just give them a flier.

KC: Uhh.

07: Then, those who like what is on the flier you can get in touch with us on this number or you can come to my house. Maybe in the neighbourhood, you can come to my house maybe I can show you where it is done or the address of where you are supposed to go, it will be there. A person should just do what is easy for them but the idea of the flier seems to make sense because talking to a person is difficult.

KC: It is difficult.

07: Uhh.

KC: Aah alright, it’s okay. Let us say you in here have been asked to come up with the PrEP program for young girls and women. What will the program that you will come up with look like, what will it look like, what is important? The PrEP program for young girls and women that you can come up with, what will it look like? Number 05.

05: Aah I think the program that we will come up with will be explaining what PrEP is.

KC: Uhh.

05: How does it work and its advantages.

KC: Uhh.

05: When we come up with such a program, it may be accepted better by young women.

KC: By young women. Okay do the others have something to say. The program that you can… what are the important things that should not be left out of your program? Number 04.

04: Aah before we get into what PrEP is or anything else, we are supposed to learn people.

KC: Uhh.

04: When I say learn people, learning to talk to people well.

KC: Uhh.

04: Like when you say something some people take time to understand so you are supposed to learn people, relate well with them, having patience.

KC: Alright, okay. Alright so let me give an example of a program right, aah let us say I have been asked to, alright. I have been asked to come up with a program of children… of a creche, of ECD that encourages them to come to school. The program that I want to come up with for ECD children, they will have classrooms with colours, colours with pictures, picture right. I want each class, at that school I want them to eat lunch or eat biscuits, eat chips in the afternoon. I want to have a playground outside so that they go out and play in the afternoon getting some air, right. That is the program I want to come up with for ECD children, so when coming up with the PrEP program for the girls that is what I want to hear when you are asked to come up with a program, what important things you look at to be included in the program. Anyone with something to say, number 07.

07: I don't know but that is what I think.

KC: Uhh.

07: That these days with the way we are living.

KC: Uhh.

07: People are now rushing where there is something.

KC: Uhh.

07: That is where they are rushing to…

KC: Rushing to.

07: Rushing to, when they are told that we will… we will give you $3 dollars we want to teach you something. An individual will say instead of spending the day sleeping, let me go to the $3 but if you say come, we have something that we want to teach you, one will say aah I will be burnt by the sun.

KC: Alright.

07: Why would I be sun burned, let me sleep. It is not important.

KC: Okay.

07: So, if you are able to do even a little something that you give people so that you can be able to gather people, even if you stop it later on. After creating what…

KC: The program.

07: Yes.

KC: Alright, an incentive should be there in the program.

07: Uhh.

KC: Alright what kind of incentive?

07: Like when we are working with women.

KC: Uhh.

07: As women we mostly like or… when it is a woman if they are given money that we are talking about, three dollars.

KC: Uhh.

07: An individual will say aah my budget had fallen short of three dollars, let me go.

KC: Let me balance things.

07: Let me go.

KC: Okay.

07: Uhh.

KC: Uhh number 04.

04: Besides money, if you give them things like these [showing a packet of biscuits] and drinks.

KC: Biscuits.

04: Some people like that.

KC: Okay number 05.

05: For young girls if the target is young girls.

KC: Uhh.

05: Maybe if you help with sanitary wear and say we are…we shall give you pads, we shall give you this. This will be important to young girls.

KC: It is important. Alright, okay. There is something I now just want to ask on this program, let's say we have come up with it. When looking at… because your program should have the place where the PrEP pills should be given. What kind of places would you like PrEP to be accessed? Like PrEP should be obtained from such and such a place, what kind of places would you want to get PrEP from? Number 07.

007 It is just like here.

KC: Uhh.

07: This is a place where when someone comes to no one knows where you are. It is far, haa even… you get into town and pass by. So, it will be good because if someone says they are going there, you just think they just came into town when she has come to the Avenues.

KC: Alright, uhh.

07: It's a place that is just…

KC: It is… there is privacy.

07: It is private, it has privacy and not public. At the health facility you can just bump into someone and they… yes.

KC: Number 05.

05: Aah I don't think it should be far as such.

KC: Uhh.

05: But it should just… some… somewhere private. Like you can just pick a random place or behind OK.

KC: Alright.

05: That is where we are doing our things, if it is holding campaigns getting into people, then we just say behind OK that is where we are holding our campaign. Somewhere that is closed when someone comes, they will not be seen.

KC: Alright, somewhere closed where people do not see where the person is going. What about the people that give PrEP, what kind of people would you want? Nurses, which nurses, from the private or general? The ones who will give you the PrEP pills, what type would you want or what you can describe to say aah we want someone with this kind of behaviour to give us the PrEP pills? Uhh number 04.

04: If they are nurses, some will not have problems because they will be on their jobs. And when they are on their jobs they are taught and are able to keep issues of what they would have found on a person.

KC: Alright.

04: But if they are from the community, the ones we were talking about, haa it is a challenge.

KC: It is a challenge.

04: Uhh.

KC: Uuh others what kind of people would you want to give you the PrEP pills? Number 08.

08: We want someone who is courteous.

KC: Someone courteous.

08: Who talks to people well. Not one who speaks rudely saying yeah, yeah, yeah [Speaking in a high-pitched voice].

KC: Alright, uhh number 05.

05: I think for the one who gives PrEP, they should not be someone from the community that is distributing the PrEP.

KC: Alright.

05: Let them be just a mere person.

KC: Who is coming from another community.

05: Who is not from the community, someone we do not know.

KC: Okay.

05: One whom when they give you, I do not get stressed thinking that it's now all over.

KC: Alright, uhh, number 07.

07: Saying what number 08 has already said, we do not need someone who is not courteous so that when you get there you can say aah nurse these pills I am feeling this. Is this what they do then she will respond to say haa do not trouble me, some do that you are now troubling me. Just go and take them quietly.

KC: Aah alright.

07: A person… a person should respond well then, they see that this issue that this person is talking about, then they get a way of counselling them to say no that is how they are.

KC: Alright.

07: Yes.

KC: What about the time to take PrEP? Time that is feasible… as you are in here representing other young girls and women who are not here. Time to… [Someone coughs] time to take PrEP what time would you want. Uuh number 05.

05: I… in my own view they are not supposed to… when giving people we give them from 8 until 11.

KC: Uhh.

05: As long as they are working hours when we say at the clinic one should just be free to take the PrEP.

KC: Uhh.

05: Because sometimes that is when they got the opportunity but when you are now limiting them they may now say it is better for me not to take the PrEP, and rush to work.

KC: Alright, because of time. Number 04, you are raising your hand.

04: Aah still on that issue of time I saw it when we were working at XXX… like this time. There are times when Covid cases are many they… they… time may be limited.

KC: Alright.

04: They may say we are serving people from 8 up to 12.

KC: Alright they no longer do as usual; they would have changed their time.

04: Yes, they will be changing.

KC: Alright, what about…

05: Curfew, there is curfew.

KC: Alright number 08 said previously that the time she took PrEP she took the pills… [Someone coughs] that was for one month, right. That is what I want to ask on the time frame, when you take the PrEP you would want a supply that when you go to the health facility you will go back again after… What time will be ideal for you, is it two weeks, one month, six months, one year? Number one.

01: I think three months is better.

KC: Three months after every three months, I go and collect.

01: Yes, I go and collect.

05: At least if I am given a supply every three months.

01: You just get tested and get your pills.

KC: Then I am given, so I am given a supply that is adequate for three months?

01: Uhh.

KC: Others, are there some who have a different view to three months?

08: I wanted to say six.

KC: Six months.

08: If you give me six my bottles.

KC: Alright, ooh when I come for the first time.

08: Give me six bottles now. Then you come back after six months.

KC: I get a supply that is enough for six months, I do not return to the health facility.

05: What makes some people not to take PrEP.

KC: Uhh.

05: They don't have the time to go and take PrEP so when they are given a supply of three, six months, they can forget for a while. When they finish and realize the bottle is left with five, I am now supposed to go back.

KC: Okay, alright moving on to the next question, right. We have now finished about the role plays; we now just want to ask a few questions that are left then we finish our discussion. Aah when looking at our male sexual partners, right. I want to ask that in your own opinion do you think male partners have influence over the taking of PrEP by young girls and women? Number 08.

08: Others do not even want.

KC: Others do not even want.

08: Even if it is their boyfriend, he does not want.

KC: Why?

08: Aah a person called man; I don’t understand them.

KC: [Chuckles]

08: They are mysterious, let me help you, I want you to be helped. I want it to help myself, no do not take so not telling him in the first place is the best.

KC: You just take in secret.

08: You hide your bottle because you shall... either you shall both die, or you take your pills and finish.

KC: Uhh, number 05.

05: I… I think men have a lot of influence.

KC: Uhh.

05: Because when it comes to married women, it is difficult to take pills every day.

KC: Uhh.

05: You can stay in the house without your husband knowing but there are some who can discuss about that. If he agrees, another may agree and say aah it’s alright, you never know you may end up taking them together with your partner, taking them together.

KC: Taking them together, get onto PrEP.

05: On PrEP.

KC: Others do you think male partners have influence on the taking up of PrEP by someone especially young girls and women? [Silence] We are about to finish, let us now be brief and finish. Number 01.

01: Yea they have great one because when you tell them that I am taking PrEP they will say haa if it is like that we have to break up.

KC: Then you break up.

01: We break up then you can take your PrEP.

05: He will say I think you are sick.

08: You are a prostitute.

[People talking at the same time]

07: Why do you want PrEP.

XXX: You are in a relationship…

07: I want to ask.

KC: Uhh.

07: That the PrEP… [Someone coughs] can it not, let's say you have gone to get it.

KC: Uhh.

07: Right, there are those little pills, small papers where pills are placed [Someone coughs] can they not be used to put PrEP pills then they do away with the bottles.

KC: Like paracet and ibuprofen.

08: Aahh.... [laughs].

07: Because those big bottles, those ones [Speaking loudly].

XXX: That bottle if it makes a sound.

07: If it makes a sound when it falls even when in the handbag if it is mistakenly shaken.

XXX: Also, still the pill…

08: It is big.

XXX: It is big.

05: It is like an ARV.

08: Paracet (shortening for paracetamol), this pill haa.

KC: Alright.

05: Also, the paracet (shortening for paracetamol), is taken every day at eight.

07: No, you know, changing time you can change time. Even in the morning, you can look for your own time especially for… for someone who… who is in a marriage. Maybe your husband spends the whole day at work.

KC: Alright.

07: You can say in the morning when he leaves for work, in the morning you take them.

KC: So the point that seven is talking about with regards to packaging of PrEP, is the point you are talking about.

07: Yes.

KC: That we do away with these bottles, then use packaging that is… [Interjection].

07: Or even in a paper because bottles are not ideal.

KC: When someone sees it, they will start to think that haa…

07: Yes.

KC: This person is on treatment.

07: Aah because the bottle is a problem.

KC: Alright.

08: When a bottle falls it makes a sound, [imitating sounds of a bottle falling down].

05: It is similar to the ARV one.

KC: Alright so moving forward to our next question. We have a study that we want to conduct, after… aah where I said we shall have many discussions in different places. When we finish, we shall do a survey that is a bit bigger. On this survey we are doing it again with young women and girls who are sexually active here and there, right. We want to hear from them also ways in which we can come up with a PrEP program, giving PrEP services that we were talking about in here. That are ideal for them so that when this program is now there, they can utilize it freely without any problems, right. So, the first thing we want to hear from you right, where can we get adolescent girls who are sexually

active. Places that you know of yourselves that aah they will be many, just mention that this place, number 07 then we proceed.

07: Uhh XXX

KC: Where exactly is the place?

07: XXX.

KC: What is done there?

07: XXX is a growth point.

KC: Uhh.

07: But there is a house that is known to be for sex workers.

KC: They stay there.

07: Yes, then there XXX also there are people like that also. XXX, the XXX there are many you will not even finish them.

KC: Okay, number 04.

04: XXX, at XXX's girls are found there. In XXX, you go to the shades.

KC: Name the places like it's a bar, a market, a school, where exactly?

04: Okay XXX at XXX's there is a little bar that is there. Then XXX, there are shades it's a bar, then XXX is a bar.

KC: Alright. Others are there other places where girls are mostly found?

08: Mostly at bars.

05: Aah I think for girls where you can get many of them is getting into bars.

KC: That is where they are found.

05: Uhh.

KC: Alright but the setup of this study we will be… let's say it is here at the XXX. We pitch our tents so the places I am asking about are like, let's say we have pitched here. Close

by and they will hear that there is a program for young girls who are sexually active, coming to that place.

08: Haa on their own direct they will not come.

05: Anyway, places where they are found are those that offer entertainment.

08: In the clubs, especially in the clubs.

05: Especially where there is entertainment.

KC: Where there is entertainment, that is where we find those age groups.

05: Uhh.

07: Also transport, the issue of transport.

XXX: Haa true.

07: You cannot say to a prostitute (female sex worker) come here from XXX; they will tell you that aah I do not come for that. So, transport, you supposed to have your transport to fetch them, wait for us at this place. Also, these people are easy because they have their older mothers. It is just like XXX that I am talking about, if you just go there and say we want to go to mother XXX. That is the one you talk to, her children. Her children, they are her children too.

KC: Okay.

KC: Okay so moving forward right, this study we are talking about that we are going to conduct sorry in the future. It includes being checked for sexually transmitted infections, STIs. [Someone coughs] So this includes asking the adolescent girls and young women to take for themselves. For this test an individual will be taking their own sample, on their own, they are taught how to take the samples on their own, right. In your own opinion do you think adolescent girls and young women will be comfortable to take their own samples than for a nurse to take them for them.

08: Haa it is comfortable.

KC: Alright.

08: Because there are some who are not comfortable being tested by a nurse, they want to test on their own.

KC: Uhh number 07.

07: There I have a question for number 08. You can test yourself, right but you know there are some people who are stubborn.

KC: Uhh.

07: They can test themselves "_+", unless maybe it is that little thing that test and show results then give them, that will do. Not for me to see it on my own…

KC: No, they just take their sample; they take their sample a vaginal swab. It will be tested, and results will come out later maybe between four to six weeks.

07: Aah so it is alright

KC: Then they are told when they indicate that they want their results. They will be told that results are now out at this place, go and get your results.

07: Aah if they are like that, that is alright.

KC: It will be alright.

07: Yes.

KC: So, the places you have mentioned are the bars, XXX, the XXX. Are they ideal for girls to be comfortable to take their samples at such places, places like that?

07: Since they are people who are not shy.

KC: Uhh.

07: They can do it because they are not shy. They are not shy.

KC: Okay.

07: Uhh.

KC: Uhmm anything else, you said since they are not shy it is possible. Anything else that is feasible that you can advise us on to say aah the point that you have stated that a person

will take a sample. I think for it to all go well there should be this and that for the samples to be taken well and be kept well. Anything else you can think of? Number 07.

07: You can pitch a tent.

KC: Uhh.

07: Like what has been said before, those tents that work with… those… those who do circumcision. You can pitch your tents so that it seems as if it has sentimental value because someone may just pass by the road and like it.

KC: Ooh

07: But if it is an open space.

KC: It can be a problem.

07: That can be a problem but if you just do it, if you just pitch your tents telling people that we have this and that. Those who are interested come so we can assist each other.

KC: Alright so moving to our question, we are left with one question, the last one. I mentioned before that those who would have taken samples shall be asked if they want their results or not. Those who would have indicated that they want their results will be given by the health worker who learnt that job. What can you advise us to tell adolescent girls and women who would have been found positive or found aah with germs that have something to do with sexually transmitted diseases, they are supposed to visit health centres? What can you advise us on how we can talk to them that your result is out but it came out like this? So that they can be able to go and seek help, number 05.

05: I think just like what happens with HIV testing.

KC: Uhh.

05: When you have not yet been told your results.

KC: Uhh.

05: Whether they are good or bad, you are first given counselling so if they work that way that they counsel you first, I think the results can be accepted better by a person.

KC: They can be accepted, others.

08: That is what I wanted to say.

KC: What can you encourage those who would have tested positive, after getting their result they are supposed to get treatment for the STI. Which places can you advise these adolescent girls and young women to get their STI treatment from, places that you can say this one and this one… is ideal for them to get STI treatment? Number 02.

02: Aah I do not have.

KC: Alright, number 07.

07: It is just like now we have here and Mbare. You know that those are the main issues or even here those are the main issues discussed.

KC: Alright.

07: So, if possible you just add that we now want to open another place. Or at this place, it will just be adding but for now when someone gets such an outcome you can just tell them that for it not to be much of a problem you can go to Mbare that is where there is what…

KC: That.

07: That.

KC: Alright anything else, eeh number 05.

05: I think some of them will not be sex workers that we will be working with.

KC: Yes, yes.

05: A person is supposed to be given the opportunity to choose, where are you comfortable receiving treatment from.

KC: Alright.

05: We have these places but where are you comfortable.

KC: Where, alright then they give their choice.

05: If it is a local clinic they can just go.

KC: Alright but number 05 where are you comfortable with going?

05: Aah I am comfortable going to the sex workers clinics.

KC: Alright not the public ones.

05: Not the public ones.

KC: Yes number 07.

07: I have a question will you be using it for… eeh will you be mixing them, the sex workers and the married women. Will it really…

KC: Erh…

07: Work out well or?

KC: What we will be doing right. What I have explained for those who will be coming to the study, in the study. There won't be groups like sex workers on their own, adolescent girls who go to school who engage in sex here and there. What is important among all of them is that they have sex, where they do it, the trade they do it for is not the criteria for them to get into this study. So even if they mix, they will not be aware that this is a sex worker, they are into sex work. This one who has sex with her boyfriend here and there, so they won't be any grouping of that sort for them to know that these are sex workers, these are not sex workers, but they will all be in the same study.

07: But being… isn't you know that sex workers know each other.

KC: Uhh.

07: They know each other.

KC: Uhh.

07: So, you know, they can say let's say what I know. We used to say when we… if you… when you are sitting, they would just say this seed cannot be kept, talking about things of…

KC: Uhh, uhh.

07: So, you know someone who is not into it. They will just start to say listen to what she is saying.

KC: Ooh alright, I get it so… on the study right. We will be saying we come and camp here then we work with those who will be coming here so we will be assuming that those who will be coming here will be associated with key populations program.

07: Alright.

KC: Then we go like to a college, then we meet with students who will be there.

07: Alright.

KC: So, it will not… someone will not feel out of place that iih the people that I am seeing coming to the study that I am a part of and what they are saying.

07: Alright.

KC: So, we will be stationed at that place, then we work and finish and go to the next.

07: Aah ooh.

KC: Such that they will be just interacting… same group, with the same age.

07: Haa I have understood.

XXX: Haa that is true.

07: Because it is a problem.

XXX: I know that at XXX there was a war.

07: Mixing them is not practical.

04: Because others will be married women and will just have been told to come. Someone who have taken their sister-in-law and come with them then us as sisters we start speaking our issues.

KC: Alright.

04: Aah then this one will say if you take my husband I will deal with you.

07: Yes, and people start fighting.

04: And we said what did you say, we started what we do at the bar at that moment.

KC: Ooh and you wanted to have a fight.

04: Yes.

KC: With the first wife [chuckles]. Aah it's okay so our last question right then we finish, when I said this, I don't know if the others had arrived yet or not. I said that PrEP is going to have different types, but it will all be PrEP. In Zimbabwe we still have pills like the ones on that paper right but there are other types that are being made. There is a ring that you can place in your vagina, and it stays for one month there working. There are injections that are being made, so there are different things that are being made. PrEP that when you take, you do not take it every day, when you take it you some time like 30 days or 60 days then you go and renew it. So, in Zimbabwe there is this one called Dapi… Dapivirine vaginal ring right that I have explained right. So that one was proved to start to be used in Zimbabwe, but I don't think it is now available.

XXX: Uhh.

KC: But being approved it was already approved for it to start being used so it also gives protection, it protects for one month, one month and so forth. How do you view it this kind that work for a long time, to promote? What are your views, what are your views on these long-term ones, long term methods? Number 07.

07: Personally on… on the ring that you are talking about there are some men who can start to touch you.

KC: Uhh.

07: They are going to place their finger; won't it be felt that this person has something she has put?

KC: Alright.

07: Like a woman's protector it sits well, it closes like this. Will it not be felt?

KC: That there is something that has been placed inside. Uhh number 08.

08: Yes something on that ring.

KC: Uhh.

08: I know it, so it cannot be felt because what can I say. The way it will be placed in the vagina it will be placed like this [demonstrating with her hands] so you… you insert it while folded like this. It gets there and settles in a round shape.

07: It…it twists number eight like a protector.

08: Yes, it just gets there and settles well. Even if he places his finger it just gets in he will not touch it… why would he want to feel that round. It cannot be felt it just gets in and goes out, getting in and out, getting out, getting out.

KC: Uhh. Number 04.

04: While we are still there like that woman, I was feeling a bit lazy of taking the pills, it has helped me I am now just inserting a ring, I am lazy with pills.

07: Does it also help in that you don't fall pregnant as well?

KC: No.

08: No.

KC: For ring, for ring it protects.

07: HIV.

KC: HIV.

07: Alright, alright.

KC: Number 05.

05: If they are people who live in places that are… for those who stay in places that are not free like… as I mentioned earlier this is including young girls some are still staying with their parents.

KC: Uhh.

05: If I have my ring, I don’t have to worry that my mother is going to come across the pills.

KC: Uhh.

05: In my bedroom I just insert my ring.

KC: Going forward like that.

05: If I have my boyfriend that I did not want knowing that I am using PrEP, he will never know.

KC: Okay.

05: I think it is alright than to take pills every day.

KC: Alright anyone with something to add as we close our discussion, number 03.

03: There are those who do drugs.

KC: Uhh.

03: And those who drink alcohol, they will not say I did not forget to take the pill. Even if you forget the ring will be there inside.

KC: Alright, it helps uhh.

XXX: What about injection?

KC: Injection, yes there is an injection again it is being manufactured, it is in the pipeline so that you can also be injected.

XXX: It is better.

XXX: That one is better.

08: I think those are the easiest methods.

KC: Than the pills.

07: Because for someone to go and get bottles it is unlikely, no.

[Others laughing]

KC: Alright, uuh.

05: If we are doing the ring many women will accept it.

KC: They will accept it.

05: Because it's just inserting the ring, right.

07: Haa even with sex workers you will succeed.

XXX: Ring is perfect.

07: Ring is ideal.

KC: Alright.

05: A person does not face challenges.

08: You will be married because of the ring.

KC: Ooh you can be married because of the ring.

08: You can be married because of the ring.

[People laughing]

07: Because you will be aware that after one month, I am now going to change I am going to get another one. Also, you will not miss getting an opportunity even if you are in marriage. Will you not get even one day where you can say as he leaves going to work, you also leave and go and get your ring [claps], you are done.

KC: Alright, are there others with anything to add? Anyone with a question on what we have been discussing before we close our discussion. Is there anything to add, anything to subtract, none?

07: Haa we are done.

KC: If there is none, I want to thank you a lot for the time that we had especially the responses that you were giving. They are helping us a lot to make this PrEP accessible, to be acceptable. To be liked especially by the young people, girls who are engaging in sex even if it is here and there or frequently, right. So, I want to thank you a lot for your time and your views. Our discussion has ended here.

[Participants clapping hands]

The End
