# Supplementary material for: Perceptions of pre-exposure prophylaxis among sexually active adolescent girls and young women in Zimbabwe–A qualitative study
Source: PLOS Glob Public Health. 2025 Dec 2;5(12):e0005396. doi: 10.1371/journal.pgph.0005396 (PMC12671731; doi:10.1371/journal.pgph.0005396)
Supplement: S1 File — (ZIP) [file pgph.0005396.s003.zip › S1_File/AGYW-FGD 02-Translation.pdf]

AGYW-FGD 02 Translation
Facilitator: KC
Note Taker: SM
Date Of FGD: 18/01/2022
Age group: 15-19 years
Translator: KC

KC: Alright as we start our discussion, I would like to thank you once again for your time that you have availed for us to do this discussion today. Uhm, I have mentioned that I am Kudzai, my colleague is Sharon we are both from CeSHHAR Zimbabwe. Today we will have a discussion on your views on PrEP (Pre-Exposure Prophylaxis) so that we understand acceptability of PrEP, reasons why there are few people taking PrEP and how it is being taken amongst adolescent girls and young women. This will provide us with important information on how we can design new interventions of providing PrEP [Baby sounds in the background], so that we improve the numbers of adolescent girls and young women who take it and continue taking it to those who would want to use it. Our discussion will take between an hour and half to two hours to complete. In this discussion we will have role plays and discussions on the aim of this study. In the role plays we will ask you to be in three groups. Each group will do a role play on how adolescent girls and young women view PrEP issues and how they perceive their risk of getting HIV. Each group will get an explanation about the role play they are supposed to act out and will have only 3minutes to agree on the content of their role play. We will then ask each group to act out the role plays followed by a discussion looking at what would have transpired in the role play, right. So, as we have agreed when we started, we are not saying people's names in the discussion. The numbers you have, that we gave you those are your names in the discussion. So, let's use those numbers in the discussion, when you want to say your point you say number 10, then you say your point so that we know that number 10 is the one who was saying these things, right. Uhm to start our discussion, my first question before we go to the role plays, my first question is that have you ever heard of PrEP? Have you ever heard of PrEP?

15: Yes, we heard about it.

KC: Alright, who says yes they have heard about PrEP, may they what is it they have heard about PrEP, what is PrEP?

15: PrEP stands for Pre-Exposure Prophylaxis.

15: Which means that if you are sexually active you can…if you have never been tested or you are sleeping with someone who is HIV positive you will be taking the medication, taking the oral PrEP so that you remain negative.

KC: Alright.

15: That's what I know.

KC: Alright, thank you number 15. Others, are there others who have heard differently from what number 15 has said? [Silence] Or is that what you wanted to say as well?

SOME: Yes.

KC: Alright, what about the PrEP, the forms of PrEP. Do you have forms of PrEP that you know of that for PrEP there is this and that? Yes number 9.

09: There are pills and ring.

[Sound of a microwave being used]

KC: There are pills and ring?

09: Uhm.

KC: Do you have much information on how the pills and ring work, what you know or what you all know? [Background Noise]

09: The pills, the pills you take them at the end of the month, ring you put it on after 2months.

KC: After 2 months.

09: Yes.

KC: Okay, other is there anything else? The pills and ring, are there other forms of PrEP that you know of or what you would want to add about the pills and ring mentioned by number 9?

[Silence 14:14-14:16]

KC: Alright, what about PrEP, like there are some who spoke and others who have not, the things you know about PrEP, where did you get the information? Where did you first know PrEP from? Information on PrEP…where did you hear PrEP from, number 9?

09: Infor…PrEP information we heard about it from a CeSHHAR hospital in Mbare.

KC: Alright, Sisters clinic in Mbare, is there anywhere else? PrEP information that you know, where did you hear it from? Number 15.

15: As young people, where we stay, we usually meet and have such discussions. Guests from clinics, from different organisations just discussing, that is how we knew.

KC: Alright so the way you meet is there an organisation that will be arranging the meetings or is it something from the community? Who organised that for you to be meeting and getting such information?

15: An organisation.

KC: What is it called?

15: Shamwari yemwanasikana.

KC: Ooh, shamwari yemwanasikana so how often are they done?

15: Aah we used to meet monthly but due…because of COVID we have spaces, we now do it on the phone.

KC: Ooh, you are now doing it on the phone so on the phone how will you be getting the communication.

15: Through WhatsApp.

KC: Through WhatsApp, alright, its fine. Number 10 your hand was raised?

10: For me PrEP I got it, information on PrEP I got it through CeSHHAR. CeSHHAR when I first knew about it, it came at…at the clinic then they said they want young people from 16 to 20 something.

KC: Ok.

10: They said…started telling us about HIV/AIDS, telling us the advantages of PrEP and its disadvantages. Then they told us that PrEP is taken by someone who is HIV negative so that is when I knew about PrEP through CeSHHAR.

KC: Hoo, alright, ok through CeSHHAR are there others? It was mentioned that through CeSHHAR, Sisters the one for CeSHHAR, Shamwari yemwanasikana, another organisation that is involved in these PrEP issues. Are there others with somewhere else they found the information which was not mentioned?

[Silence 06:43-06:45]

KC: Alright if there is no one we now want to move to our role plays we mentioned before where we will split into three groups, right. So, our three groups are like this, group one there is Chido and Koko, group two there is Mai Bhobhi and Mai Juru, then group three there are three friends who are Peppa, Sky and Princess right. So, for the groups, the Chido and Koko one is like this.

Scenario 1 Chido and KoKo

Chido is 16 years, she is having a sexual relationship with an older man who is aged 50 years, who is aged 50s and above. So Chido has started taking PrEP as she is worried about contracting HIV. So, she thinks of her friend Koko who is 19 years as well whom she thinks she is at risk of getting HIV from her sexual relations and she suggests to Koko that she also takes PrEP so there should be a role play between Chido and Koko telling us what you think will happen if Chido says that. So Chido is telling her friend Koko about PrEP that she started using so the scenario need to show how Koko will react. Let us it is a real lufe situation that has happened that you have been approached by your friend telling you about PrEP, how would you react as Koko, that is the first role play.

Scenario 2 Mai Bhobhi and Mai Juru

Then the second one has Mai Bhobhi and Mai Juru, both are married. Mai Bhobhi is 23 and she is married, Mai Juru is 21 and she is married as well. Mai Bhobhi's husband has a habit of having girlfriends quite a lot of times and she is worried about getting HIV. So, she heard about PrEP on radio, being advertised and she went and got her PrEP. She now has 6 months taking PrEP right, but now, she is now thinking of not using PrEP. Do a role play between Mai Bhobhi and Mai Juru bringing out why Mai Bhobhi now wants to using PrEP, that is the second one.

Scenario 3-Peppa, Sky and Princess

Scenario 3-Peppa, Sky and Princess

Then the third one is amongst three friends who are in school in their 20s and these ones they have got sexual relations with their partners, and they go to the same school. So, they

have been selected at their school to design a PrEP program for adolescents girls and young women for an organisation called CeSHHAR. They are designing a program. So, they have been asked to design a programme that is preferred by adolescent girls and young women. So, in their programme they must design a programme that…that if presented to them they will go and take PrEP if they are interested in PrEP.

KC: So these are our three role plays that we want to do, right? So, what we will do we will get in groups, we are 8 so one will have 2 and others will be 3,3. Then the Mai Bhobhi one even you are 3, only 2 need to be selected to do the role play after doing your discussions. So Chido and Koko we are taking number 9 and number 4, you are the ones doing Chido and Koko right. Then for Mai Bhobhi and Mai Juru I am taking number 11, number 15, then the…the one for three friends there will be number 13, number 10 and number…what number is yours, on your hand, number 16 right. So now we are breaking, we allocate about 2minutes to discuss in your groups right, on what you want to bring out in the role plays reading what is written on the paper that I gave you, then we come back… [Someone clears throat] and we continue so we are now breaking for now. [Background discussions]

KC: I pause here, where there is the record.

KC: Alright so we are now continuing with our discussion right. So, we start with group 1 for the first role play for Chido and Koko right so they are the ones who will first do theirs, after that we discuss then we move to the next role play and we discuss. We move to the next role play and we discuss, then we will be left with a few questions then we finish. So, we start with Chido and Koko, come, come in the front and do your role play raising your voices so that its well captured by our recorder. [Background noise] Yaa, stand side by side.

Role play 1

Chido: Aah hello.

Koko: Hello.

Chido: How are you Koko my friend. [Sound of a handshake]

Koko: I am okay, how are you?

Chido: I am good, how is home?

Koko: Its fine.

Chido: How is work, is work fine?

Koko: Work is fine.

Chido: Haa that's fine, we are see that.

Koko: Uh.

Chido: Iii, I have come with some gossip my friend, that I heard going round in the community. PrEP what, what PrEP, I am taking PrEP. Have you heard about it?

Koko: PrEP, what is that, eeh my friend you want to get us killed now?

Chido: No isn't it they are being taken by us sex workers.

Koko: Iiih I will use the condom my friend, eeh PrEP, PrEP. You hear about this and that, I am no longer listening aah for me condom is the best.

Chido: Which condom, condom has some disadvantages that it can burst. PrEP is good in that you take it in your own time, it protects from HIV, you do not get HIV.

Koko: Uhm my friend I have had so many incidents where it burst and I am still going there, you take it.

Chido: No, my friend, no do not do that, let us take PrEP. My friend do not be like that.

Koko: Plus, for taking pills is difficult, it chokes me, aah for to take pills aah no.

Chido: If the pill does not work well for you, there is the ring my friend.

Koko: Ring for what?

Chido: That is being inserted for 2months.

Koko: Uhm, now that's satanism, I cannot do that.

Chido: Aah, my friend we want to look after our health.

Koko: Uhm, my friend do not say that, ring for what, ring for what?

Chido: To be inserted.

Koko: Aah I am looking for an engagement ring, then I go a PrEP ring, what for my friend.

Chido: My friend do not be like that, no my friend do not be like that.

Koko: Are you not being too much my friend.

Chido: If it's a problem, they are saying an injection is coming, don't you want it?

Koko: Iiii iiii I am tired, there is a Covid one, then PrEP iih.

Chido: Koko do not be like that, let us look after our health.

Koko: I am looking after mine with a condom my friend.

Chido: No, it's okay.

Koko: I am committed; I do not know about you. [People laughing and clapping hands]

KC: Thank you Chido and Koko, thank you for your discussion. So now we want to have a discussion from the role play done by Chido and Koko right. Firstly we want to ask that are there girls who are like Chido who have sexual relationships with older men when they are young? Is it something common?

SOME: Uhu.

KC: Number 11.

11: Yes.

KC: Yes expla…explain that they are there who does this and that.

11: There are young girls who sleep with men without using protection.

KC: Alright, without protection, not using protection.

11: Yes.

KC: 16.

16: Yes, they are there even in…this generation we want to date older men because they are the ones who gives us money quickly and the money will be a lot.

KC: Approximately how much… [Interruption]

16: Aah maybe she can get $50 USD.

KC: Uhm.

16: So, if I want to date a young man he can give me $10 of which $10 does not cover anything for me, I would want a lot of money. So… [Interruption]

KC: What would you want it to cover?

16: Aaah.

KC: What are the things that it should cover for young girls, what should it cover that money? 10.

10: Uhm, number 10, what is happening is that the young people money to cover school expenses we are not getting it at home so I end up saying no I am just sitting yet our generation there is competition for dressing. There is a type of dressing that is there, I end up getting envious that no my friend is dressing so well and eating whatever she wants yet I am just sitting at home. I am not going to school, the money is not enough, the food its not that good so I end thinking that the older men are better because these older men can say I want maybe one round. For that one round if he says he will give me $40 USD at least that $40 USD I can buy some things that I want.

KC: Uhm.

10: But what is happening is that the older men do not want to use condoms. So I end up saying I can take a risk because we would be having some permanent clients and others who are not permanent so I can risk a bit to say aah let us use a condom but they end up being angry so I think PrEP is….

KC: It’s alright.

10: Yes.

KC: Why do older men do not want to use condoms?

10: Condom they say it will not be pleasurable, and it will not be erect but if you… [laughing]

KC: Speak, speak, why?

15: 15 I want to add on more, some they say we do not eat a sweet in its wrapper, we want real live [Chuckles]

KC: Alright.

15: Because they say that in a condom it is not pleasurable as compare without a condom.

KC: Ok.

15: So, plus I noticed most men who says they do not want to wear a condom, those are the older men who are infected, and they want to infect us.

**: That they want to pass to us.

KC: Alright.

15: Because they do not want to do what, to wear a condom. So, this PrEP one of the things is that if you refuse to wear a condom, I take my PrEP I am not at risk but now without a condom is expensive compared to with a condom.

KC: Hoo, alright. So, if he says he will give some money, it's a risk life of saying aah this is between life and death, let us just do it.

KC: Alright.

15: It would have increased a bit.

The money would have increased, alright. Are there others who want to add on the issue of having relationships with older men, the adolescent girls, is there something to add? [Silence] Alright what about someone like Chido, we mentioned that Chido is 16 years and has a sexual relationship with an older man. Do you think Chido is at risk of getting HIV? Yes 10.

10: Yes number 10, Chido is at risk of getting it because one, which one is Chido there…One Chido is refusing, are you Chido? [Asking the role play]

Chido is number 9.

10: Chido?

KC: Uhu.

10: Yes, Chido is at risk of getting HIV because she is refusing to take PrEP, is it you?

**: Its Koko.

KC: Alright, so Chido the risk we are asking her is that she is…she has a sexual relationship with an older man so that is what we are asking that her sexual relationship with an older man, is she at risk of getting HIV because of that relationship?

10: Yes.

KC: Does it put her at risk?

10: Yes.

KC: Why? How is she at risk?

14: Because we don't know when she started taking PrEP, maybe they have been together with that man for a long time having unprotected sex, so she is at risk.

KC: Alright, others, what makes Chido or girls in Chido's scenario be at risk of getting HIV? What puts them at risk, 16?

16: Because we have sex with a man, we were not tested. I don't know his status, so we just have sex without using any protection.

KC: Alright.

16: So, you know…

15: Chido is at risk because she is only taking PrEP, she does not care about the condom because she is depending on one thing, but I suggest that Chido uses a condom whilst taking PrEP it will protect her a lot.

KC: It will protect her a lot, alright. Ok, 10.

10: Yaa, haa, PrEP when its available right, PrEP I think that it will want us to take PrEP, and we leave the condom behind because in the market the men do not want the condom. So, I want to ask that PrEP for her to be at risk is it not 100% such that if I take PrEP every day, I cannot get infected because if she on PrEP to a greater extent she is alright because she is taking PrEP. She is safe rather than we say she is on PrEP, then we add the condom.

KC: Alright.

10: It will not work.

KC: Alright, on that note PrEP is very effective if used consistently such that you take it at times you were told at the clinic or hospital to take it but PrEP does not offer protection against STIs. PrEP does not protect from pregnancy right so that's where the condom comes in. If she uses PrEP plus condom, they are protected against the STIs, she can protect against pregnancy so that is why its advised that if someone uses PrEP, they must not stop using condoms. Let them use their condoms along with the PrEP to be protected from a lot of things that are coming at that time.

**: Uhm.

KC: Alright, so as we proceed, I want to ask on health issues amongst adolescent girls and young women, is it something common that adolescent girls share or talk to other people about their health decisions? It may be just discussing health issues, or I am sick, I have gone to the hospital, I have done this. Do they discuss with other people these adolescent girls? Yes 16.

16: Haa there is a risk on that one.

KC: Uhm.

16: Because I would say I have told my friend about my health issues, I don't know that I am…I don't know that what I told her will she tell people or keeping to herself so if she cannot keep it to herself. Obvious she will spread it in the community that aah that child, she had some STIs. She is now on HIV treatment, she got infected with HIV, you know.

KC: Ok, uhh others with something to add, 10.

10: Plus, I have told this one right.

KC: Uhu.

10: One I don't know if she can keep it to herself, two she may say to me go to the hospital. A hospital which is not the CeSHHAR ones or the ones for the girl child. Its just the same as if I have an STI, if I go to the public hospital, lets say they will tell you we do not have medication. They will write a prescription for you and you will be told to go and buy in the pharmacy. The pharmacy might be at Zengeza 2, where I am known by people there.

KC: Uhu.

10: They will start judging you saying how old are you, you want to buy this. They are already judging you; you are actually buying; I am not sure how it is.

KC: Alright.

10: So, on that one haa…

KC: It's a challenge.

10: It's a challenge to tell someone that I am sick.

KC: Alright, okay are there others? Yes number 9.

09: From my point of view the discussion should not…just discussing related issues but not telling each other our secrets.

KC: Ohh, no secrets?

ALL: Uhu.

KC: General?

ALL: Uhu.

KC: So, the adolescent girls don't they have even one person whom they can share with even a best friend. Are there people they can share with their intimate issues that are health related, are there people they can share with?

15: Yaa, number 15 personally I think I have one person whom I know if I tell them my issues she can assist. That aah you are getting it all wrong here, go and get help on this there are counsellors. There are a lot of people whom if you talk to them nicely, they will open up and the other issues is that in our communities it is difficult for people to share because stigma and discrimination is still not yet what…

KC: Over.

15: Not over with people and people have one problem of ignorance because when one is said to have had an STI, how are they supposed to get assistance. So, its needs some knowledge for them to understand.

KC: Hoo, ok. Alright is there anyone with anything to add? Alright, uhm as we proceed do you think adolescent girls can get…there was a time when we asked where you got the information you are sharing for PrEP right. These adolescent girls where do you think they can get PrEP information, that information, where can they get it from or we say someone who is interested where would they get the information, 9?

09: Like now we are participating in a PrEP study, we may be able to tell others about PrEP, where they can get it and how they take it.

KC: Alright, giving each amongst yourselves, number 14.

14: What I see is that if only this information can be viral in many places. In the radios, TVs, all over for people to know about the oral PrEP.

KC: Alright.

15: I think if we have roadshows because our generation if we hear that there is something taking place we rush there so if we can have that where people will be told through the road shows so and so, it helps.

KC: They can be assisted.

15: Yes.

KC: Alright, it's ok. Uhm, as we proceed right, looking at our discussion that we were having or the role play between Chido and Koko, what are your views on Koko's responsive? When she spoke, she was told by Chido to take PrEP, lets start from there. What do you think of Koko's responses looking at real life situations? Uhh 15.

15: Aah, Koko showed that she does not want, she said I do not take pills they affect me. She was informed you can have the ring, she said I want an engagement ring not that ring you are talking about.

KC: Uhm.

15: She showed that she does not want it saying aah PrEP I don't think it…I don't see how it helps me. I think using a condom is better than PrEP, she resisted that, she resisted that.

KC: Alright, why do you think she responded in that way, she had such a strong resistance, why is that so, 9?

09: Uhm, in the community we live in if PrEP issues are discussed, different issues come up. Being said aah PrEP, the pill is too big, it's difficult to swallow, maybe that is why she was refusing.

KC: Alright.

09: Because it's as big as the HIV one so it's difficult to swallow.

KC: Alright so that is what is discussed in the communities.

SOME: Uhu.

KC: Alright, yes 15.

15: Full information on PrEP Koko has not yet received it. If she finds someone who can sit down with her, explain to her and tell how PrEP works. I think in future Koko will understand and will be able to take it.

KC: Taking PrEP.

[Someone coughs]

15: Uhu.

KC: Alright, are there others? Why do you think she responded in such a way, 10.

10: Newness of the drug as we know it PrEP it's a new pill that has been recently introduced so we have our beliefs that if something is new…Some say it makes you sick, some say you need to take PrEP before you eat or after eating, actually after eating as it makes you feel dizzy. Some say it has different colours; Koko might be afraid wondering which one is the real one and how does it…where do I get it because this one did not give her knowledge on how everything works.

KC: Alright, okay, okay are there other responses that we can expect from someone like Koko who has been approached and being told about PrEP for the first time by her friend? Are there other responses that were not disclosed that we can expect to get if it happens in real life? Are there other responses that can come out that did not come out in this role play, number 10?

10: Us as young people, when we are moving around, we refer to ourselves as virgins.

KC: Uhh.

10: All of us do not want to be like that.

KC: Alright.

10: So, the more she has been told about PrEP, if it was me in real life I would ask you why did you tell me, is it that you have heard I am sexually active.

KC: Alright.

10: That is what one will tell you that aah you made the wrong assumptions about me, I am actually a virgin. All that you are telling me…but now us as sex workers we are frank with each other. Aah my friend there is PrEP so, so, so but now these teenagers they are still

growing up so I will tell you that what you telling me is something else, I don't want to hear it, do you think I actually do those things.

KC: Alright, ok, ok, uhm are there other responses that we can expect? [Silence] Alright as we proceed, do you… [Someone coughing] what do you think are the benefit of PrEP to adolescent girls and young women? What are the benefits for PrEP that you can point out, yes 10?

10: Aah from my view the rates of people getting infected with HIV are increasing, daily basis people are getting infected with HIV. So, I think PrEP for us we benefit in that maybe PrEP if PrEP is well introduced and being taken consistently, males who are positive will…people who are positive will get few because we will be aware of PrEP so if we use PrEP and condoms we can benefit a lot.

KC: Ok, alright.

15: I want to add.

KC: Uhu.

15: Sorry I'm going to crush her point.

KC: Yes.

15: Take a look at our statistics for people testing positive, they are getting low. I am sorry, I am going to crush you most people now have knowledge on knowing that HIV is not AIDS. It's just a mile that we took because for one to have AIDS it will be HIV that was not sustained.

KC: Uhh.

15: So, a lot of people now know that PrEP we use it and most of them know it and one will never tell you that I am taking PrEP because they will think some things are unstable.

KC: Alright.

15: Taking HIV treatment pills, one cannot disclose freely, it will be your personal life.

KC: Ok, ok.

16: But most people now know.

KC: They are now using, what about the PrEP benefits, benefit for using PrEP what is it? What can we say are the advantages of using PrEP, which ones would you mention that PrEP is good for A, B, C, D?

16: For me personally its more beneficial to me because there is nothing that is painful that you were born negative and then as you grow you get positive.

KC: Alright.

16: That…for you to understand that you are already infected when you got it from someone, it's just painful.

KC: Alright, ok, ok.

16: So, PrEP came and shortened another life that we were going to get into without knowing.

KC: Ooh ok, ok are there others with something to add on the benefits? [Silence] Alright as we proceed, in your view and from your interactions in the different communities where you are coming from what do you think can stop, like what Koko did that she refused that she does not want PrEP, she is not interested. What can be barriers for adolescent girls to taking PrEP?

09: From my view, PrEP, the containers where they are packaging the pills are just the same with the ones for HIV [People giggling]. If they change those containers, even if they put in plastic packaging maybe it can work, and they also need to change the size of the pill. [People laughing]

KC: Ok, so firstly you talked about packaging, that container we are seeing on that picture, is that the one you are referring to?

09: Yes.

KC: So, you said if this container changes to a plastic one, what kind of plastic?

09: Haa, even like the ones for paracet.

KC: Like the paracet packaging?

09: Uhu.

KC: Alright, it's okay what is the benefit of those ones? Making that change, how does it help?

09: It helps in that if I meet my client I will not be able to open my container which will be making a sound in the bag… [People speaking at the same time]

KC: Hoo.

09: I just take my plastic and take my pill, now this one has too much labour [People laughing]

KC: Alright, then on the size you said it's too big.

09: Uhu.

KC: What size would you want, the pill what size would you want if you can give some examples that we are familiar with?

09: If it's the same with the painease one.

KC: Like the pain ease, paracet?

10: Yes, plus let it be round such that it's understandable even if you say to someone it's another form of a tablet.

**: You just say I am….

10: Plus, colour, colour, are they white in color these PrEP pills?

**: They seem to be blue.

KC: They seem to be blue, uhh.

09: Those are the ones that are taken…are they not the same with the HIV ones?

10: No.

09: They are not.

10: I actually take PrEP.

KC: Alright, so colour should change from blue to what?

10: At least white, white one will not know what the tablet is for plus the way it is… [showing her hands]

KC: The shape becomes round?

10: Yes.

KC: Ok, number 13.

13: Plus, about the container that has been mentioned that it's too big, if you meet your client if he sees you opening it and taking a pill from it. He would want to know what that container is for, isn't it for HIV pills. He would want to understand what the pills are for.

KC: Alright, ok, alright I understand so the points that have been raised are about PrEP that the pill should change, the container should be changed.

**: Uhm.

KC: Alright, is there anything else, yes 15.

15: Sorry guys I'm going to crush her I wanted to ask what time PrEP is taken because people are saying if you meet a client. You cannot say if you meet a client then you say I now want to take the pills.

10: No.

15: I must come from home being all settled so I want to understand what time.

KC: Alright so she wants to understand about the time you talked about, let us start with 13 then we come to you.

13: Let us say he has come to see you, maybe you have not taken your PrEP pills. Then he comes before you take them of which time that will be the time to take PrEP, and you cannot take that big container. Sometimes you cannot go outside or go to another room to be able to take the pills.

KC: Alright, ok. Alright its clear, right?

15: Yes.

KC: Alright, it's fine is there anything else besides the pill being big, besides the packaging of the pills? Is there anything else that can be a barrier to taking PrEP, number 10?

10: Yea, what I think the places that you are…its like here at CeSHHAR I liked it for one thing, if someone sees you getting here, very few know what is done here.

KC: Uhm.

10: But places like the clinics there is a corner that people know that this corner if for ART pills. People who are on ART they go to this corner, people who are…for PrEP they go to this corner, so I think if you can change the collection point and make it in such a way

someone can just get in and not even know what is done there. Not to have a designated corner, that is for female sex workers.

16: Or those ladies who work at night.

10: Yes, it will not give a good picture in our community. Plus, haaa ehe….

KC: Alright so the place.

10: The collection and the people who provide it they should not be rude. Those who give out PrEP, that what makes other people not to go and collect it because those old women they ask a lot of questions.

KC: What will they be asking?

10: When was the last time you had se, this PrEP its…they first ask if you are sexually active, do you have a male partner?

KC: Alright are there any other barriers? Ok, as we proceed what are the facilitators that you can think of, what are the facilitators to PrEP uptake among adolescent girls, what can encourage PrEP uptake among the age groups like yours? What are the facilitators? What can be the facilitators, we talked about the barriers, we listed all of them, what can be the facilitators? What can be the facilitators, you talked about the barriers, in mentioning the barriers you were also mentioning the facilitators. That if you modify your packaging, if you change your pill, it can be taken in a better way, right.

15: Uhm.

KC: Those are some of the things that we are asking, what can encourage people to take PrEP.

15: Yaa, I think that there is time when you do visits to us in our areas, they move around with cars such that everyone now knows [chuckles] that is a hospital vehicle. If it comes and parks at the gate, I will be in a difficult position such that if I am asked I am not sure how to respond.

KC: Alright so that might be a barrier?

15: Yes.

KC: Alright okay its fine, 10.

10: But still on that issue, we do not want to lie the CeSHHAR staff do not just unannounced, they first ask you if you comfortable with it.

KC & 10: For us to come.

10: For you to just see them unannounced but they will ask you because its like I have started taking PrEP, they let you know that if you go for 3months, 4months without coming after you have started taking it we would want to know the reason why.

KC: The reasons why.

10: Or you might keep on appearing in their database, yet you have passed away or you are sick or there is another reason that needs…but that visit they first call you. Maybe some people they just go unannounced because maybe I do not want to tell my relative, maybe my relatives they do not even know I am going out at night so they must…but if they call me that's also good. Maybe I am a single mother so that will be fine.

KC: Alright, what about from the facilitators, are there any that you can think of that…Lets say for example would it motivate you to take PrEP, what would encourage you to take PrEP? What can motivate you to say aah if this and this is available, I will use PrEP.
[Silence]

KC: Nothing will motivate you?

10: I beg your pardon.

KC: Things that can motivate you, what we can say facilitators such that if those things are in place, it will make the adolescent girls want to take PrEP.

10: Hoo.

KC: Yes, for them to be motivated to use PrEP, what can motivate them to use PrEP?

10: Aah, as an adolescent girl PrEP, you should make the customer happy. Do like the road shows that we talked about earlier, if you are making your plans do not just put PrEP out there. If you think that, you sister Kudzi if you think that today I want to go to Zengeza and socialise with XXX and others. You know organisations like yours can come with t-shirts, caps or sanitary wear and you introduce your topic saying there PrEP, its like this and that. You will see many women taking PrEP.

KC: Alright.

10: So always make us happy not just to…yes PrEP is exciting for us but you must go an extra mile in making us happy "_" [People laughing]

15: Please the customer who busy beer… [singing]

KC: Alright. 12, is there anything you wanted to say.

12: That's what I wanted to say.

KC: Aah alright, its ok. Uhm from what you know what are the places where adolescent girls and young women can get PrEP services? Where can PrEP be accessible from in the communities we live in Zimbabwe, where is PrEP is found? 10.

10: Yaa, PrEP is available in clinics.

KC: Which ones, they are different? There are public ones.

10: The public ones.

KC: There are private ones as well.

10: The public clinics, I didn't know about PrEP…I only knew PrEP being given through CeSHHAR because the CeSHHAR clinic comes at…at…at the public clinics like most of the ones in Chitungwiza. Every Tuesday so that is when I knew that there is something called CeSHHAR, there is something called CeSHHAR. At the CeSHHAR clinic you get PrEP but I am not sure about the other public clinics, if you go there and say I want PrEP, is it now available.

KC: Ok, ok are there any other places?

10: Population.

KC: Population services?

09: Uhm.

KC: Alright, its ok. What other places do you think will work well in giving PrEP which have not been mentioned such that…If you are asked to suggest other places that you think can work well. Where else can PrEP be given out at, that can work well, 10?

10: I think that in…as you know the CeSHHAR staf…[Background Noise and short pause]

KC: Alright you may continue.

10: As you CeSHHAR staff know we survive by selling sex, so I am thinking if you do what OPHID does. Do you know that OPHID knows that there are ladies, they do what they call moonlight.

KC: Uhm.

10: They pitch their tents and do some outreach and they usually do them during the night such that they come and pitch their tents. They say no we do not want sex workers who end up being sick, come and let us give you condoms, we give you this and that. Such that everyone will get tested and get what, PrEP. One gets tested and gets her PrEP, if there are condoms you then give us. I think if peoples see a tent pitched during the night they will come and say aah what are you doing here and you explain to them.

KC: Alright.

10: You explain it to them, so…so…so…so and you give them PrEP, I think we will then like it.

KC: Alright, so we do those outreaches, the moonlight of during the evening.

10: The evenings ones because in the afternoon who will come, people do not want to be seen. In the evening if they come that will make us happy right. Then you close your tents, and each person goes in one, one. This thing of you coming and you up your lights. Maybe I test positive, or something has happened inside, or I want to tell you that I have an STI, but I do not want someone to know about it.

KC: Alright.

10: So, if you do your things well.

KC: Alright so you only want things that offer privacy?

10: Privacy.

KC: Alright so the night outreach of moon light, are there any other places? Are there any other places that you can suggest…Alright, so we now want to do role play number two, we have finished Chido and Koko's role play, we now want to do role play number 2. Role play number 2, is for mai Bhobhi and mai Juru so mai Bhobhi and mai Juru may come upfront and do your role play and we start the discussion and we proceed like that.

AGYW-FGD 02 Translation
Facilitator: KC
Note Taker: SM
Date Of FGD: 18/01/2022
Age group: 15-19 years
Translator: KC

[People whispering]

Role play 2

Mai Juru: *[Laughing] Hie.*

Mai Bhobhi: *Hi mai Juru.*

Mai Juru: *You are around?*

Mai Bhobhi: *I am around, how are you doing?*

Mai Juru: *You do not know the gossip going around. They are saying your husband is being promiscuous, he is sleeping around a lot.*

Mai Bhobhi: *My dear I have an issue that I wanted to talk to you about that is bothering me.*

Mai Juru: *Tell me my friend, let me sit down.*

Mai Bhobhi: *My husband is being promiscuous; he is sleeping with many women. This is something that is affecting me in that I am close to getting infected with HIV.*

Mai Juru: *What about PrEP that is being widely discussed, have you heard of it?*

Mai Bhobhi: *So I just heard…I heard on radio about this PrEP programme so that is what I am currently using but I am thinking of stopping it.*

Mai Juru: *You want to stop using it? You want to die, you know there is nothing more painful than being infected when you are innocent. When you are innocent, you just get infected and you accept it saying thank Mhofu (an African totem), be reasonable.*

Mai Bhobhi: *I am now just accepting the situation, I am really thinking of stopping it.*

Mai Juru: *Why would you want to stop using PrEP when its protecting you, there is no way you will change him, he is your husband but do not stop using PrEP.*

Mai Bhobhi: *Haa I am thinking of not using it.*

[People laughing and clapping]

Mai Juru: *No if you stop using PrEP you would done a painful thing. Really a grown woman like you speaking such words that do not have value. No fear God, let me go home. [People laughing and clapping]*

KC: Thank you mai Bhobhi and mai Juru, right firstly the discussion is centred on mai Bhobhi and mai Juru, what are your views about this scenario? What do you think about this role

play in relation to our everyday life? Are there any adolescent girls and young women who are married who are facing such situations like what mai Bhobhi, is it common for married young women?

10: Yea its common, it's happening us adolescent girls and young women we are dating mai Bhobhi's husband.

KC: Yes, mai Bhobhi.

10: So they are things that the man will not be coming back home, not doing this and that. You will be hearing stories that he has passed by with someone else in the car, he has done this so this is happening, people are going through this.

KC: Alright, we have said its happening. Is it an easy decision for someone like mai Bhobhi, a young woman like mai Bhobhi to decide let me start using PrEP? Is it an easy decision for one to make? For mai Bhobhi to decide that I am now using PrEP, is it an easy decision for adolescent girls and young women who are married for them to start using PrEP?

15: No number 15.

KC: Uhh.

15: It's not easy for mai Bhobhi to agree with that, she has her situation with baba Bhobhi, she is hearing that he is being promiscuous. Mai Bhobhi then thought if I do not take action I will be infected and for sure she will be infected because our husbands do not want to use condoms, that is not a secret.

KC: Alright.

15: So mai Bhobhi seems there is something else pushing her to stop it.

KC: To use…

15: Yet she had started, and she is thinking I am 21 years old, I got married recently. What about when I get to 70 something years, just taking PrEP. I think that the thinking she has for her to stop.

KC: To stop it, but its not an easy decision, alright. Why is it not an easy decision, why is it not easy to decide to start using PrEP because of her situation, why is it not easy, its difficult?

10: Yaa, it's difficult because, one baba Bhobhi, they are husband and wife and we culturally we know that a wife does not make final decisions. A wife does not decide that today I want to start taking family planning without agreeing as a family.

KC: Uhu, uhu.

10: 2, baba Bhobhi if he comes across the pills container in the house I do not think it will…fo how long will you keep on hiding them. Something will happen baba Bhobhi will pick those pills or one day he does not go out of the house. You will be staying in a one room, you will not know how to take your pills so PrEP for the married ones, does it not require discussing and agree first plus with the men, there is no way of agreeing to anything because men will always deny that they are being promiscuous.

KC: Uhu, uhu.

10: He will ask how did you think of this, so how is it taken because these people are married yet they are staying in a one room, how will it work. Its just difficult for your husband to find out that you are now taking family pl…family planning. That is why we had said if they can be packaged in a similar way with paracet because you can just tell him I have a headache, you go ahead and take the pills.

KC: Alright.

10: This container iih.

KC: It's a challenge?

10: Yes because there is no way you will continuously take them for two days. When you are caught you just say I am taking pain ease, or look for something else to say.

KC: Alright, what about…adolescent girls and young women who are married, what are the barriers to them using PrEP? Or…challenges in them taking PrEP, what makes them not use PrEP, the married ones? What are the barriers for using PrEP amongst adolescent girls and young women who are married, which can be the challenges in using PrEP, yes 10?

10: They need information to say first of all PrEP does not have side effects because the…the ones we know, what do we call them? Most prevention things people say they have some side effects.

KC: Uhm.

10: So will it not have any side effects in the future such that you will fail to conceive or…we want to know how it works.

KC: Alright, ok.

10: So, if you give them adequate information.

KC: Information, knowledge might motivate them to take PrEP.

10: To take PrEP, yes.

KC: Alright, ok. Anything else that can be barriers or motivators to them taking PrEP, the married ones? Do you think their husbands have an influence in PrEP being taken by these women?

**: Uhu.

KC: What, what kind of influence do they have?

16: I think there are other men, you would have agreed maybe he is the one who is positive, I am negative. We agree with him that it's okay baby, I love you, I love you as you are but when it comes to taking PrEP he starts saying you are now stigmatising me but we…we would have agreed. For you to explain to him that I am trying to protect myself, he starts saying you told me until death do us apart, we must do the same thing.

KC: Ok.

10: You know it differs with the character if the husband in the home.

KC: Uhh, ok alright, others, 10?

10: I want to respond on that, saying for the discordant couples, those ones were the other one is positive, and the other is negative. They do not have protected sex; they do not have protected sex. They have a way of doing it, they are educated about it at the hospital but from what I know they are not properly using PrEP. You use PrEP but they are told something at the hospital, I really do not know what sister Kudzi how it's done but there is something that is done to them at the hospital.

KC: Alright.

10: They do not just make up things on their own, they have things that they get from the hospital.

KC: Alright.

16: Yes we never know because people will be two in their home, you get it so it's difficult to explain what will be happening.

KC: Alright. Others what do you think, do the husbands have an influence of saying do…do not take PrEP for the young women in marriages? Are there others with a different thought? There is no one? [Silence] Alright, is there…number 10 had already mentioned some of the things to say if they get information, the married women can be motivated to take PrEP. Is there anything else that you think can motivate them to take PrEP, the married women, is there anything else, yes 9?

09: Another motivator, if you start by giving information to the men and they understand how PrEP is taken, how it is taken.

KC: Alright.

09: This reduces the violence in the homes.

KC: Alright, 10.

10: I think isn't it when one goes to book, when they book their pregnancy. You go together as husband and wife, that is the chance to talk more and discuss PrEP when they are together.

KC: Alright.

10: So that in the future it will not a new thing.

KC: Alright, ok is there anyone with something to add? Alright do you think that in real life it can happen that adolescent girls and young women can stop using PrEP when they have started using it like mai Bhobhi but she is now thinking of stopping it, does it happen?

**: Uhu.

KC: If it happens why, the reasons why someone who have started using PrEP and they now want to stop using it saying I do not want to continue using it, number 10?

10: I think it's what is discussed amongst people, when I started knowing about PrEP there was someone who came at to our home…PrEP at the public clinic. By that time, the way I had heard of it was that there were some XXX students who tried making their own drug, the way it was said it was done but this tablet they want to see if it does not kill or not.

KC: Ok.

10: If you get it but you will be getting an incentive of $10 that is how I heard it but its only given to sex workers only, its not given to other women, only sex workers. I said ooh, if you have an identification, you bring what, what. Then I went and I got it. The way they were doing it, what they do…sex workers like us were saying you get your containers, some only wanted money.

KC: Alright.

10: So sometimes to please the customer sometimes it does not work because you are pleasing them but they are not taking the pills. It is said they would take the containers, I used to do it too, they get the containers, and you say nurse I am taking them consistently and you get home, and you throw them away.

KC: Uhuuu.

10: Because you would have heard that its making people ill or other people saying they are feeling dizzy because of it. Some will be saying its too big, it chokes, some will be saying a lot of things about it, then you will be left behind. So in the public hospitals they will ask you if you are taking your pills, they will ask you a lot of questions and you will be lying to them.

KC: Uhu.

10: So, I think when it was first introduced, people were scared of it, aah it's an HIV pill, even today people still say that. Why do you like that PrEP, its smelly, it causes nauseous, they say a lot of things about it so I think the issue of knowledge if it's well discussed and educated it will go well.

KC: Alright, ok, ok others what can make someone discontinue using PrEP when they have started using it and they just think of stopping like what mai Bhobhi is doing? What can be the barriers to PrEP continuation?

15: Number 15.

KC: Uhh.

15: I think its lack of counselling; one would have started taking the PrEP pills without being told the good and bad things so they will be thinking about a lot of things such that they will be thinking about a lot.

KC: Alright.

15: So, if people are well educated in a polite way, if they are educated, I think it will help.

KC: Ok, alright, lack of counselling, thank you. Others, is there anything to add before we continue, things that can make someone discontinue taking PrEP, what can be the barrier? That can make someone to say I do not want to use PrEP, when they have been using, number 9?

09: Maybe she will be having too many side effects.

KC: Uhh, on what?

09: In her body, in her body.

KC: Alright, side effects.

09: Uhu.

KC: Ooh alright that might make him stop using them?

09: Uhm.

KC: Ok, what can be done to assist adolescent girls and young women who are currently using PrEP for them to continue using it. For them not to decide to discontinue when they have started using it. The suggestions that you can give us to say aah if you do this I think it will help for them to keep wanting to use it since they had started, number 10?

10: I think PrEP should be readily available.

KC: Hoo.

10: Things like PrEP should not run out because if you say its not available and I go and borrow a container from a friend when I do not want them to know.

KC: Alright.

10: They are things which should be readily available, if I know that the clinic comes every Tuesday. Then you are told today Tuesday they did not come, yet I wanted them to come, I wanted to get my pills. That will make me stop taking it and you will not convince me, I will say you are not organised so do not give me your medication.

KC: Alright, ok alright others what can motivate adolescent girls and young women to keep on using PrEP? This one mentioned consistence, let it be available at all times, the services to continue being offered, is there anything else? [Silence] Alright if there is nothing, we are now proceeding to role play number 3. The one for Peppa, Sky and Princess, the three girls who have been assigned a task at school to design a PrEP program that will be acceptable amongst young women. So may you come, the three of you and do the role play, the last role play.

Role Play 3

[People talking]

KC: So, its PEPPA, Sky and Princess.

Sky: Aah hi Peppa.

Peppa: Aah hi, how are you Sky?

Sky: I am well [Whispering]

KC: Group number 3 let's be serious and we proceed, we started late so may you act so that we finish.

10: What are we supposed to do?

KC: You are supposed to design a PrEP program that is acceptable by adolescent girls and young women. So, in your program you are explaining to us, discussing how the program is supposed to be like this and this.

10: Alright.

Princess: Hi Peppa.

Peppa: I am well.

Princess: Aah Sky.

Sky: I am well.

Princess: How are you ladies.

Princess: Aah ladies did you hear about the PrEP program that is coming.

Peppa: Aah we heard about it.

Sky: We heard about it through the grapevine, but we never knew how the things will be done.

Princess: There is an organisation that came saying let's design a PrEP program, I don't know, what do you think about it, how can it be done?

Sky: What is PrEP used for?

Peppa: What they will do doing the people who are taking about this PrEP?

Princess: Aah we have lost the story here.

KC: [Laughing] We want you to discuss like for example that I gave you early right. Let's say we have been asked to design a program for early childhood development (ECD) that will make the children want to come for ECD. So, the program I am designing for ECCD is that there should be an ECD centre that is close to the children so that they will not have challenges with walking to school.

Princess: Alright.

KC: If there is a school bus, things like that.

Princess: So, I think the PrEP program they should look for a place in the community because these days due to COVID moving around is not good. You will not have the money sometimes so I think those who are designing the program, when we are designing the program if could find a good place. A good place where we can go and do our things there.

Sky: Uhm, for PrEP are they not able to bring an injection because aah taking pills is burdensome, the pill is too big.

Princess: Yes, you can say that again mai Pipi.

Sky: Aah it's too much, they should see what they can do.

Peppa: Don't you know anyone who works there, someone you know who can speak on our behalf that the girls are complaining, they want an injection.

Princess: Injection is better, you can say that again.

Peppa: Yes, I cannot be caught by my parent taking it because injection you just get it at the hospital.

Princess: Or something like depo.

Peppa: Eeh now my mother can just walk in and find me taking the pill.

Princess: She will ask what it is for.

Sky: What is it for.

Princess: So, I think if they can fix that plus the people giving PrEP should be able to maintain confidentiality.

Sky: Yes, they should be able to maintain confidentiality because it's a challenge.

KC: Alright.

[People clapping hands]

KC: Thank you group 3 so let's discuss a bit about group 3 right. Uhm, do you think the program they have designed, from what we heard them discussing, they mentioned where PrEP is accessed from, should be from the community. There should be privacy at the place. The ones who give out PrEP they should be able to maintain confidentiality.

ALL: Uhu.

KC: Such that they do not go around disclosing people's issues plus they mentioned the pills should be replaced with the injection to make things better. What they said do you think it can work well if a program has been designed like that? Does it work, is it acceptable to adolescent girls and young women such a program, number 9?

09: It will work well because taking pills daily is painful as compared to just getting your injection once, you will go for a long time with it.

KC: Alright, you said it works on the injection, number 10.

10: I think in the community is also okay. In our community we have what we call community what, what?

09: The community care worker (CCW).

10: Yes, the elderly women, like us we now have the knowledge.

KC: Uhm.

10: You give me my PrEP, my containers. I now have the knowledge; I go in the community and will staying at my home. My other colleagues that I am telling, instead of having challenges of not having the CeSHHAR outreach clinic coming. They just know there is aunt XXX, we go and collect from her.

KC: Alright.

10: Yes, so in the community, it will be better for us as we will be familiar with each other, next who is going to collect for us. Next there are times when you want to go and sell some things, with the pill you cannot move around. I cannot go for six months maybe I will be at work so if they could address that.

KC: Alright, ok, are there others with anyone to comment on the program that has been designed? What do you think works well or what you think does not work so well on this program that has been designed? Is there anything else? [Silence] Alright, as we proceed what things are important to you as adolescent girls and young women that makes a PrEP program for adolescent girls be successful? Such that if the program is rolled out, it will be successful, and PrEP will be used, and adherence will be high. What is important to you that should be part of the program whenever a PrEP program is designed? [Silence]

KC: Is there anything else that we…maybe that we did not mention…You talked about confidentiality, is there anything else that you think is important that should not be left out besides the confidentiality issue, is there anything else, number 10?

10: When PrEP comes…

KC: XXX where are you going?

10: She is coming back, when PrEP is available provide it along with the condoms, not to just bring PrEP without the condoms.

KC: Alright, combining with other things that I wanted by adolescent girls and young women.

10: Uhu.

KC: Alright, PrEP and condoms, is there anything else? Alright, what do you think works well, you mentioned some of the things saying if there is no confidentiality it will not work well. Is there anything else that can make it be a bad one?

10: Yes, those who will be giving out PrEP will be tarnishing the image of PrEP because you will be saying for me to go to the clinic and get PrEP you will be asked a lot of things so sometimes it might be better to stay at home.

KC: Alright, ok I want to ask on the location, dispensing location in the community. What about the pharmacy, is it okay to dispense at the pharmacy?

10: If you give the pharmacies, number 10 you might find it being sold and we will not get it.

**: It's sold.

KC: PrEP in the pharmacy will be sold.

**: Yes, it will be sold.

10: So we will not be able to afford the money, our clients are giving us less money so I don't think…I am looking for surviving, I am looking for food. I have gone to get some PrEP but there are free condoms, they are things that should be given for free.

KC: Being given for free.

10: So to charge any money it's a big no because if I get $2 from a client, then at the pharmacy is being sold, I cannot…

KC: Alright so a pharmacy is not ideal, what about at the clinic, in the public clinics? Will they work well for adolescent girls to get their PrEP there, number 10?

10: They can work but they need to improve, 1, 2 the people who give it in the public clinic they treat people badly. They think that those sex workers are now coming, they don't regard us as people. So, we ask that those who will be giving PrEP may they be good people.

KC: Alright, people who treat people well.

10: Yes, not to be so loud such that you will be heard they have come for PrEP, the sex workers have come, I do not want.

KC: Alright, are there any other places that you think will be ideal to get PrEP for the adolescent girls and you women? Are there any other places, besides the pharmacy, the clinic, is there any other place, number 14?

14: Even in churches, to educate church women.

KC: Alright.

14: For people to take.

KC: Alright, okay in churches, is there any other place? Alright, what about the people who give PrEP, it was mentioned earlier that the people who give PrEP should have an attitude. We want to understand more on this one, what about the pharmacists, can they work well besi…not looking at selling or things being sold, the pharmacists can they give PrEP?

10: Yes, the pharmacists, I think everything that we get for free these days I think what happens in a beerhall they are just placed there, and you collect for yourself but now PrEP it's not possible. I think the in pharmacy, most pharmacists they treat people well. Not knowing in the public, yes at the clinic because they might get overwhelmed because there are now a lot of people.

KC: Ooh alright.

10: At the pharmacy once you get there, they should put a tag, and a person do…you then require some knowledge so getting from the pharmacy does not work so well.

KC: Alright, ok, alright. What about the frequency, someone mentioned pills one takes for one month, then ring its inserted and lasts for one month or two. On the frequency let look at oral PrEP, the pills which frequency would you prefer?

10: Just being given?

KC: Of using oral, the one we are using we take the pills every day, you take them every day.

10: Yes.

KC: So, they are the ones we are asking, the frequency which one would you prefer for the adolescent girls, the PrEP frequency, number 10.

10: I was thinking if you say for the pills right.

KC: Uhu.

10: A pill that is taken once a week.

KC: Once per week.

10: Or even stopping the pills and just use the injection because if I get the injection I will not forget, I will not…because the pill maybe I have travelled, isn't it PrEP you have your notebook.

KC: Uhu.

10: I have travelled from here; I am now going…I once did that, and I left my PrEP here when I was travelling to Chinhoyi. When I got there, I remembered that I forgot my PrEP, now I have a client what should I do. They said they want a number from the small book so what I should I do; I cannot give you PrEP. I was thinking for PrEP it's something that I cannot lie to you saying I am taking PrEP when I am not and I am asking for you to give me even five tablets so that I can use during this journey then continue with the ones at home, but you do not want, I don't know what really happens. So, I think the injection, you just travel.

KC: Alright so for example, injection how frequently would you want it? Should it last for a month?

10: 3 months.

KC: That lasts for 2months?

09: 2 months I think they are okay.

**: 3, 3months.

KC: 3?

ALL: Yes.

KC: Alright. What about the ring, vaginal ring, it lasts for how long?

10: Ring where will it be inserted? On the arm. [Someone laughing]

KC: No ring it's inserted in the vagina.

15: Vagina.

10: Hee?

KC: In is inserted like the way one puts on a female condom; that's how you put it on. It sits like that and offers protection.

09: But it will be producing…

KC: It will be producing some medications at regular times, offering some protection.

10: But it does not have any side effect?

KC: That is still under research.

10: Alright. Aah on the ring, we do not want the ring because if you introduce those rings…most teenagers will not come. The young women will not come, they will not come because we are shy.

KC: Uhm.

10: For my vagina to be seen, I do not want with it. Then ring, the ring, loop we always lie to each other in the community saying anything that is inserted in the vagina has some side effects. So, the ring, is it like the ring that we put on the fingers.

KC: Uhm aah it's like the one on the female condom.

10: Ooh those ones.

KC: Yes.

10: Aah we do not want it.

KC: That's the shape it…

10: Those people who say things like on the loop, they say if a woman has loop she feels in a certain way…so if its inserted deep inside, we do not know where it will reach… [People laughing] then she feels…it will be affected so injection is good like, these ones they will make us lose money.

KC: Alright, what about the pills, how do you prefer the frequency.

**: Even once.

KC: Which frequency would you prefer?

15: Even once per month.

KC: Even once per month, alright.

10: You just take your pills and take them at the clinic.

KC: Alright, its ok. In terms of locations which we discussed earlier, would you prefer locations where there are other services being offered? Such that you get family planning, you get STI treatment, you get PrEP, many services at the same place, number 10?

10: If you do it like what is done in Mbare, Mbare is blessed. They walk to the hospital, for us

KC: Alright, it's okay so we have finished the role plays, we just want to ask a few remaining questions then we will be done.

**: Uhu.

KC: I want to ask about the partners that you might have, the men…their role, the male partners in the relationships with adolescent girls and young women do you think they have an influence in PrEP uptake? Men whether married or not just male partners in the relationships, do they have an influence, number 10?

10: I once asked my boyfriend to accompany me to the clinic when we were coming from town. What do you want to do there my dear, I said that aah I want to go and take my PrEP. Then he asked what is PrEP that you want to go and take. When I tried explaining, he said aah it's now for…he broke up with me saying if it's now about the pills…men if they just hear pills…Then I went alone and I showed him and he said no you are lying because of what we have said before that PrEP looks the same with ARV. There is no difference, so he was now saying what is this aah so I…men do not want it.

KC: Ooh, ok with that association?

10: Yes.

KC: Ooh alright, ok is there anyone with anything to add on the role of men? [Silence] Alright, currently do you feel like you can easily access family planning services? Adolescent girls and young women, is it easy for them to access family planning services wherever they what, is it easy, 10?

10: In the public clinics, what I told you before that the nurses there are so unfriendly.

KC: Uhm.

10: If you go there wanting to get loop, they will tell you if you have had a child before?

KC: Uhm.

10: Yet if I am asked if I have had a child, what they are asking me is none of your business one, if you tell them that I do not have a child…if only they were asking. Once you tell them that I have never had a child, they now say we will not give you loop. [Door opening] You see, you will come here tomorrow complaining saying you gave me loop what, what, yet I am at work. All those things that you are asking me it does not concern you, right one.

KC: Uhm.

10: Secondly the payments at the public clinics, they close on weekends if you want to have the loop if it's the weekend. If you want to get your family planning after it has expired and you want to get it over the weekend, I am now going at the Population, at Population they require money.

KC: Uhu.

10: So, I was thinking if only CeSHHAR can have its own clinic, I can come at whatever time I can. I know that from my CeSHHAR nurses I can get all the things that I want because when I come here, I have never been told we are not giving the injections because the nurse is not around.

KC: Hoo.

10: It will be there so CeSHHAR if you build something closer right where we can get family planning, the nurse will not be asking me questions because they know that that's my work. These people want to…they have too many questions that they ask, such a young person like why you want to have the loop.

KC: Hoo.

10: So, we will end up going to Population where we pay $3, what can we do?

KC: They I can get it without anyone asking me a lot of things.

10: Noone will ask me plus at Population, they are not that busy, here if I say nurse I have something that is troubling me, maybe I got impregnated. They give you counselling but there they will say you are coming here telling is about your pregnancy, so we do not want that.

KC: Alright, others do you think family planning services are easily accessed, we are almost done, [Someone laughing], we are now moving to the next question. There is…we explained it when we were piloting the consent forms when we were explaining that we will have another study component that will be done later. Researching on developing a PrEP program for adolescent girls. So, we want to see places where we can get adolescent girls and young women, being comfortable coming to the study. So, we want to hear from you which places can we find adolescent girls who are sexually active whether sex workers or not, which ones can they come to that will make them feel comfortable, number 9.

09: Here is okay.

KC: At DIC (Drop-in Centre)?

09: Uhu.

KC: Ok, number 10?

10: If it's in our area, it's like at those head offices for water payments. If someone sees me going there, they will not know what is being done there, they will just assume I am going to pay may council rates.

KC: Alright.

10: Now if you go to a clinic, everyone who has come to book her pregnancy they will want to know what you are doing there.

KC: What you are doing there.

10: But if you go and ask because most programs even the Mavambo (a local NGO), the CeSHHAR team used to come there. It's a place where you will have your office where…and all of you there are sex workers so no one will…[Interruption]

KC: What are those offices?

10: Like where I am talking about.

**: Administration.

10: Where people pay their water bills.

**: At the council.

KC: At the council, at the council offices?

10: Yes.

KC: Ooh, ok. Alright.

10: After that, when someone has come from their home, if you provide refreshments next time we will come.

KC: Refreshments like which ones?

10: Like before, Mavambo used to bring biscuits and drinks.

KC: Alright.

10: Yes, but for an grown up, drink and biscuits I will not want it because I have my appointment with an older man who wants to give me $5. You have called me here, I will not stay for a drink so I will not come.

KC: Ok, alright. Others, number 9.

09: Even at school if you come doing your workshop, no one will know what is being done, they will think we are in school.

KC: Ooh, at school, the school set up, ok. Alright is there anyone else on the places that we can get sexually active adolescent girls?
[Silence]

KC: Alright, uhm so part of the study it involves people who will…not all of them some who will self-collect vaginal samples for STI testing right. Do you think adolescent girls will be comfortable with self-collecting and not having a nurse to collect. She will just be taught how it's done and be given a container and closing it after collecting. Will they want it? Number 10? Alright so self-collection is acceptable?

**: Yes.

KC: So, all the places you have mentioned at the council offices, at schools, at places like these ones will they work well especially on that party of collecting vaginal samples, do the places you have mentioned work well.

10: It will work because…at the council, these offices that are no longer being used. At the council there are curtains, those curtains but if it's the clinic we do not want our clinic,

there are no curtains. Someone will just pass through pepping like if someone who pass through now, we can see him.

KC: What if there is, let's say at the clinic but there is…let's say a public clinic but they have pitched some tents.

10: With that kind of bed.

KC: Yes.

10: Its okay.

KC: With that stretcher…that set up is possible.

10: Yes, that can work

KC: Alright.

10: As long there are not many people.

KC: Alright, on the results, those who want to know their results they will be asked if you collect your sample would you want to know your STI results. If she says yes, she will be informed that the results are out, you can collect them. So, I want to ask how do we tell them about their results? Too…would want to…to…what ways would you prefer to be told your results, number 10? May others also speak so that we finish.

10: I think…thank you…we have done all the things, the…the results how do you tell me. I thought if you call me just like how you called me, aah haa XXX how are you, I am well. May you please come to DIC (Drop-in Centre) and make it clear from the start there is no bus fare but we want you to be there.

KC: Hoo.

10: Then you know if XXX is able to come or not.

KC: If she cannot make it.

10: Because when you came, we would have discussed that results so, so, so.

KC: I want them.

10: So, if you receive the phone call you know the issue.

KC: It's the results.

10: Not to disclose the results over the phone, maybe I am with my rich client, and he will start asking what this is, he would want to know the issue.

KC: Hoo alright.

10: So, if you call XXX are you available, we want to see you at DIC (Drop-in Centre), I will come.

KC: We give results in person.

10: In person, if I throw them in the road, it's my choice.

KC: It's my choice.

10: Not to say…

KC: Alright, let's say the results have come out being positive for an STI. For treatment where would be ideal for adolescent girls to get treated for an STI? Where they would go and get treatment, 10.

10: Here.

KC: At DIC (Drop-in Centre)?

10: At DIC (Drop-in Centre), even if I come here with my rich client and he waits for me outside, they will not know what's done here.

KC: Alright.

10: Now you are being told they are not there, its full, DIC (Drop-in Centre), has closed so, so. So, I was thinking that if the results are positive, they call me here and offer me treatment. Not at the clinic, there are people who know me, hearing me scream because of that injection. [Making noise [People laughing] People will wonder what is happening.

KC: Alright, ok, ok are there any who want to add other things, we have finished, this is the last question. Are there others with different views from what have been discussed? There is none? Alright, thank you we have finished our discussion. Thank you for your time, thank you for your views, is there anyone with a question, is there anyone who want to ask something before we close? [Silence] None, alright, if there is none thank you.

10: I want to add something sis Kudzi, is it still recording?

KC: Yes.

KC: Alright, thank you so much number 10. Thank you for your time, we have to the end of our discussion.

The End
