# Supplementary material for: Perceptions of pre-exposure prophylaxis among sexually active adolescent girls and young women in Zimbabwe–A qualitative study
Source: PLOS Glob Public Health. 2025 Dec 2;5(12):e0005396. doi: 10.1371/journal.pgph.0005396 (PMC12671731; doi:10.1371/journal.pgph.0005396)
Supplement: S1 File — (ZIP) [file pgph.0005396.s003.zip › S1_File/AGYW-FGD 03-Translation.pdf]

AGYW-FGD 03 Translation
Facilitator: KC
Note Taker: FM
Date Of FGD: 24/01/2022
Age group: 20-24 years
Translator: SM

KC: Alright, so as we start our discussion, I would like to thank you once again for your time that you have spared, I know you have limited time, and you want to rush home, but I just want to thank you for your time for us to have this discussion. Like I said before, my name is Kudzai and with me here is Fadzai, we are from CeSHHAR Zimbabwe. We would like to discuss issues concerning the PrEP programme or in other words medication known as PrEP which stands for Pre-Exposure Prophylaxis so that we understand its acceptability, reasons why a few people are taking PrEP, as well as issues regarding PrEP being taken by young adolescent girls. This will enlighten us as well as inform us in deciding how best we can design new programmes or improve existing programmes of PrEP, right. Uuh the discussion we are going to have, like I said before, we will try our best not to take a lengthy time because we have started a bit late so we will try to shorten it, right. So, like I explained earlier, we are going to have role plays demonstrating issues we will discuss. So that we see if the issues are spelt out, how well we will discuss it, please do not forget to raise your voices and also feel free to express your thoughts, we really want to understand your views. Before we start, is there anyone with a question? [Silence] Anyone with a question? [Baby making sounds]

KC: No one? Alright. So, my first question is, have you ever heard about PrEP?

XXX: No, I haven't.

KC: Is there anyone who have heard about PrEP?

XXX: Not yet.

XXX: No.

XXX: Not yet.

KC: All of you in here, you have never heard about PrEP?

ALL: Yes.

KC: Is this the first-time hearing about PrEP?

ALL: Yes.

KC: Alright, so I will just explain right, like I explained before in the beginning. PrEP stands for Pre-Exposure Prophylaxis, so it is a drug that is being used to protect one from getting infected with HIV, right.

XXX: Uum.

KC: So, it is a drug that is taken by someone who believes is at risk of getting infected with HIV. So, in Zimbabwe the PrEP drug that we have so far, is in tablet form, right. One is given the tablets to take them for a specified time from the clinic or from the hospital that you will take it for this long, right. So, that's what we have on PrEP at the moment. So, if there is no one who has ever heard about PrEP, and without much to talk about PrEP we now want to get into our groups that I have talked about, right. So those small groups I have talked about there are stories there… [Facilitator asking the scenarios from the notetaker]. So, what I'm doing now is that I'm now pausing the recorder so that we can discuss in our groups then we will continue right. So, firstly what I will do is that, I will explain all the three scenarios.

Scenario 1: Chido and Koko

The first group comprises of two girls, the two girls are friends. One is named Chido and the other Koko. Chido is 16 years old, right and Chido is being intimate with elderly man in his 50s. She has just started taking PrEP, since she has realized that she is at risk of contracting HIV. She also thinks, Koko, her friend who is 19 years old is also at risk of contracting HIV since she has multiple boyfriends she has been intimate with. Chido goes to Koko with the intention of telling her about PrEP, so that she can take PrEP. So, this role play is centred on Chido telling Koko about PrEP, that there is PrEP that is…it can do this and that, personally I am taking it. Would you also want to take it? So, we would like to hear how Koko responds, what will she say about this story she has heard, right, that's what we would want to hear.

Scenario 2: Mai Bhobhi and Mai Juru

So, the second role play is about two women Mai Bhobhi and Mai Juru. One is aged 23 and the other 21, both are married and are staying with their husbands, right. They are just like the

women we have here today, right. But Mai Bhobhi's husband has having a system of having multiple girlfriends occasionally, right.

[Baby crying and mum calming the baby down]

So, Mai Bhobhi has heard of PrEP on the radio and realizes her situation that her husband is promiscuous, and she decides to take PrEP. She went to the hospital and was given PrEP, she now has been taking PrEP for 6 months now, not too sure of what happened to her, and she is deciding not to take PrEP anymore, she meets Mai Juru her friend and tells her that PrEP is like this and that, I no longer want to take PrEP. So, we would want to hear the reasons that she may be having, why is she no longer interested having taken the pill for 6 months, but she no longer wants PrEP, what are the reasons, right

Scenario 3: Peppa, Princess and Sky

The last one is about three friends, these three friends are still in school. Aah, they learn at the same school, but they are now being sexually intimate in their lives, right. So, at their school they have been selected to design a programme about PrEP, such that it can easily be liked and taken by adolescent girls. So, they need to design a programme in such a way that if it is brought to a community such as Mbare, adolescent girls and young women easily adopt the programme and make use of it. So, they are designing, putting together all their views and thoughts that are important in the programme, so that if the programme contains this and that, young adolescent girls quickly adopt taking PrEP, right.

KC: So, those are the three scenarios that we have so I will give each group. We are now pausing and discuss for two minutes. Two, three minutes to decide your acting then we discuss two, three minutes, and come back as soon as the time is over. When we come back, we will do role plays as we do our discussion so that we will not take much time so now…

[Recording paused for role play discussions]

KC: Right, we are coming back to the group.

[Participants still discussing]

KC: We are now coming back; we are coming back. Let's come back, let's come back.

XXX: We are back.

KC: Chido and Koko group come back to the main group, come back, come back, come back, come back. Right, so we are now starting, Chido and Koko are the ones starting. Don't stand up, do it while sitted there with your babies, right.

Some: Alright.

KC: Sit there, so, Chido and Koko you can act out now. Those who are acting as Chido and Koko can you…

XXX: Put the recorder closer to you.

KC: And raise your voices so that it will come out clearly.

XXX: [People laughing] Don't laugh.

Role Play 01

Chido: Hi, Koko, my friend.

Koko: How are you my dear?

Chido: I'm fine, I was on my way coming to your place.

Koko: Is everything ok, my friend?

Chido: Aah uhmm I have a sugar daddy that I am seeing.

Koko: What's wrong my friend.

Chido: Aah he is promiscuous, he has a lot of money and all ladies like him. [Baby crying] he is having unprotected sex, I'm now…I realised that I will end up getting infected with HIV. That's when I heard about PrEP that it protects from getting infected with HIV. Aah, I now have some months taking it, I thought of you my friend that you are also at risk with your sugar daddy. Aah, better to go because we can't let go of these men, we have nothing, we don't have any food or clothes. If we let go of them, we would have done nothing, but I have realised that aah, it is important now that there is PrEP

program, let's go and get those tablets my friend. Taking them so that we what we will not be infected with HIV.

Koko: It's all good my friend, aren't there some injections? If they see us taking tablets, will they not suspect those other things?

Chido: Aah it doesn't matter dear, what we want is to avoid risking, that they will be…that they will think that we are taking some tablets for HIV when we want to protect ourselves, it doesn't matter.

Koko: There is nothing wrong my friend so we can just go. Thank you for coming with good advice.

Chido: That's good.

Koko: Thank you, my friend.

Chido: That's fine my friend.

[Other participants clapping hands and cheering for them]

XXX: Well, done!

XXX: Haa, this one is the best.

XXX: Haa, they have done it very well.

KC: Thank you Chido and Koko, thank you, right. Right, so what we are now doing is that we are now discussing role of Chido and Koko thinking that…if there is anything left going forward.

XXX: Uhh.

KC: So, firstly there is a point raised when they were doing their role play. There is someone who said we need them because they buy us things. Do you think in real life we have ladies like Chido who have relationships with people who are older than them, do we have such people?

XXX: The need for money.

XXX: Yes, because of the need for money.

KC: Right, anyone who would have said yes, let her say there are some who get involved in such relationships because of such, such things. Uhh, we are beginning to discuss now, anyone who wants to say something you just raise your hand and say out your point, number 20.

20: They are many because they will need food and clothing.

KC: Alright, uum, she said they will need food, so they will be doing it so that they can get food, and clothes, yes.

XXX: They are many because some do not have parents who can support them, so they just think of doing anything.

XXX: Some will be treated roughly.

XXX: Some will be treated roughly they don't know they were just born and left like that and they were dumped.

XXX: They don't know their parents.

KC: Uhh. Alright, others. Is there anything else that drives young ladies to get into these relationships besides food, being taken care of, is there anything else? [Silence] Alright, do you think a young lady like Chido who has a sexual relationship with someone older than her is at risk of getting HIV? If she is at risk, her risk is, is she at risk, let's start from there?

XXX: Yes, she is at risk.

XXX: She is at risk.

KC: Uhh raise your hands and share your ideas, is she at risk? Yes, number 18.

18: Yes, she is at risk.

KC: She is at risk.

18: Yes.

KC: How is she at risk, how is she at risk? What is exposing her to the risk? 18.

18: Some men who would want to have unprotected sex with her…unprotected sex since they are giving them money.

KC: Alright, uum, Unprotected sex puts them at risk. Is there anything else that put her at risk? [Silence] Alright, what do you think when we look at health issues for youths and young adults, right, do you think youths and young adults do share or do they share issues to do with their health with other people? Issues to do… issues to do with their health or when one gets sick, tested, or anything, do they tell others, these adolescents and young adults?

XXX: Yes.

KC: Do they share?

XXX: Yes.

XXX: They do not share.

XXX: It's a secret.

XXX: Mostly it is a secret.

XXX: There is nothing like that.

XXX: A few.

KC: She said there is nothing like that, they do not share, and some said a few. Why don't the adolescents disclose? 24.

XXX: They are afraid of being laughed at.

24: Some share, when you share with someone, some people cannot keep a secret, they will share with everyone, and people will laugh at you.

KC: Uhh, others, they fear being laughed at, they fear that the one they would have told would not keep it a secret, it will spread all over. Is there anything else that hinders them from disclosing? [Silence] Alright, but isn't there anyone who can share even with

someone? Even one person to share with those health-related issues? [Silence] There is none?

Some: There are there.

24: They can share with their parents.

KC: They can share with who?

24: With their parents.

KC: Their parents?

XXX: Uhh.

KC: Yes, some, some can share, they share with just a little, yes, 26.

26: Someone you can share with is a nurse, the one you can see that she is the one you can share with.

KC: Alright, the nurse who will be helping you. Uhh, others, 25?

25: Pastors.

KC: Pastors from the church?

25: Uhh.

KC: Alright, okay, so these people you have talked about that aah, nurses and pastors, parents are the ones you will be…are the ones thought to be secret keepers…

ALL: Eeh.

KC: Information.

XXX: Yes, they keep it secret.

KC: Alright, okay. Alright, looking at PrEP issues, right, you see like…I asked you and you have all said you have never heard about PrEP?

XXX: Uhh.

KC: Is there anywhere you think you can get PrEP information? Let's say you want information on PrEP, where do you think you can get it, or where you can suggest that these places. Or if such…such places can have information about PrEP, adolescent girls and young women can get PrEP information? Uhh, you can speak, 20.

20: Health facilities.

KC: Health facilities, uhm, 21.

21: In schools.

KC: In schools.

21: Uum [Baby making sounds].

KC: Others, where else? Health facilities…

XXX: There is no other.

KC: Where?

XXX: In beer halls.

KC: In beerhalls, uum. Why these places, in schools, health facilities, beerhalls, why in these places?

XXX: [Baby still making sounds] Sorry…sorry my baby.

KC: 22.

22: That's where most people meet most of the time.

KC: That's where most people meet most of the time. Others? [Silence] alright, it's okay. We are now moving on to Koko's response, right.

XXX: Uum.

KC: Right, when Koko was told about PrEP, she said she might like it, right?

XXX: Uum

KC: But she first asked that aren't there any injections, tablets, if I'm seen taking tablets, I will be associated that maybe I'm now involved in other programs, right.

ALL: Uum

KC: Alright, looking at Koko's response do you think those are the responses we can get in real life when someone has been approached by her friend and told about PrEP? Do you think that's the response that will be given after being told about PrEP? Are those the responses that will come out?

XXX: Uum

XXX: Yes.

KC: Is that so?

XXX: Yes.

KC: Eeh 24, alright, aah aah why do you think people will respond this way? Looking at Koko's response, why do you think Koko responded that way, 18?

18: Because they would have found ways that help them protect themselves from HIV.

KC: Alright, uum, others why do you think Koko responded the way she did, 24?

24: Because she knows that her husband is also promiscuous.

KC: Alright, uum okay. Aah, what other responses can we expect from this scenario that we had, what other responses that did not come out in the role play? Wh…what are the other likely responses? One has been told about PrEP by her friend what else can she say which was not said here? [Silence] 25.

25: There is another response that, I wanted to ask first then talk later.

KC: Uum.

25: Asking that, like you have collected those tablets, if you forget to take them is there any effect?

KC: If you forget taking them, what happens is that, as with any other tablet if you forget taking it, the time you remember you quickly take it. You will not have to compensate for the whole day if you had forgotten to take them, you will not take them like for yesterday and today. Aah aah, only from the time you remember then you continue.

25: Alright, like I have gone for a funeral, and I forgot my tablets at home. I have gone to a funeral and have about 2 days.

KC: Uum.

25: Can I take them the time I will get back home?

KC: Yes, you must continue but taking a break also has some effects that's why it is encouraged that when you are taking like when you are taking an antibiotic course, take your course everyday until it is finished. Do not say like today you forget and then you continue and today you forget it affects the way the medication works, so it is just the same with this it had to be adhered to. If you forget and remembers it you have to continue from that time you remember it.

25: So, that is another challenge that can happen or that aah, what if I forget to take the tablets, I will assume that I'm safe but the time I forgot to take them has an effect, some people forget for real. One may travel and forget the tablets.

KC: Okay.

25: But maybe if there was a tablet that can be taken maybe once, you take it once and you that…

XXX: [Interjects] or per 6 months.

25: … you will be protected for 6 months or for 3 months at least we can take them.

KC: Alright, okay I get it. Uum is there any other response? This one said she can say that aah, what if I forget to take the tablets? Is there any other response that can come out of Koko and Chido's role play? [Silence] Alright, looking at PrEP you have heard about here for the first time, do you think PrEP is good for young girls and women, is it something good? 20.

20: Yes, because men nowadays are promiscuous.

KC: Uum, alright, so how is it good when they are promiscuous?

20: For protection.

KC: Alright.

20: And prevention beforehand.

KC: Alright, uum, others, is there anyone with a different view from that it is good for prevention? Anything else why it is good? [Silence]. Alright, it's okay, in your view what do you think can hinder, there is something that was said in that group that aah being seen taking the tablets people will think that…it is something that we have realised as a barrier to the uptake of PrEP, people might start to suspect that aah maybe. Is there anything else that will be a barrier for people from taking up PrEP? Looking on the adolescents, is there anything that can be a barrier to them from taking up PrEP services, something that did not come out in the role play? Is there anything that can be a barrier to them? There is nothing? 25.

25: What I think can be a barrier is that, if people see you taking PrEP they will think that your husband is promiscuous and they will despise you that aah, maybe her husband has many sexual partners. Or they will despise me that I'm a sex worker, that look, why is she taking PrEP.

KC: Alright, uum anyone else? Yes, 24.

24: Yes, there is something because if her husband has many sexual partners. He might stop his wife from moving around because he knows that his wife maybe if she moves around she will do just like what, what I do.

KC: Alright, alright, so that can be a barrier.

24: Yes.

KC: What about the facilitators to PrEP uptake? We have realised that facilitators that have been mentioned one would have a partner with many other sexual partners.

ALL: Uum

KC: So, she can be encouraged to take PrEP.

XXX: Uum.

KC: Is there anything else that can motivate someone to take up PrEP, youths and young adults, any motivators? Not what we have said that one might have a promiscuous partner, is there anything else that can facilitate the uptake of PrEP? 26.

26: Iih, what I encourage is that some of us we will be breastfeeding so we will be doing it so that children will not be infected or other things that might happen.

KC: Alright, alright, she said so that you can protect your child [Baby making sounds] so that we will not spread the virus to the baby right. Alright, uum, in your opinion where do you think adolescent girls and young women can they get PrEP services? Where can they go and get PrEP?

XXX: In schools and health facilities.

KC: Uum

XXX: In beerhalls.

KC: In schools, health facilities, beerhalls, anywhere else where they can get PrEP services? Let's say someone wants the services where else can the services be available? [Babies making sounds]. Alright, what are the advantages of getting PrEP in schools? What are the benefits of getting PrEP in schools? Uum.

XXX: Because those are the places where most people meet especially at school that's where many adolescents are.

KC: Alright.

XXX: At school.

KC: Alright.

XXX: At health facilities we meet adolescents at health facilities, we meet there.

KC: Alright, uum [Baby sneezing]. What about beerhalls what are the benefits of having PrEP in beerhalls? Is it fine having PrEP in Beerhalls?

XXX: Uum

KC: Uum, 24

24: Beerhalls are good in that that's where most people meet, and also, we don't know the status of some of the people we will be with, that's where most relationships start from.

KC: Alright, okay, alright. Aah, what about the pharmacy, is it okay having PrEP at the pharmacy?

ALL: Yes. yes.

KC: There is someone who hasn't said anything [People laughing].

XXX: Yes.

KC: 19 say something [Everyone laughing] why is the pharmacy good, having PrEP at the pharmacy?

19: Because that's where most people go to buy medicines for children you can tell them that PrEP is available in here.

KC: Alright.

XXX; Even coming out on TV.

XXX: Even privacy.

KC: Privacy at the pharmacy is it attainable. Okay uum, is there anything else why a pharmacy is good? [Silence] what about the disadvantages of a pharmacy? Is there a disadvantage of having PrEP from a pharmacy?

XXX: Aah it is safe.

KC: 25

25: I'm thinking that those pharmacies, there are some pharmacies for locals who can sell to you saying it is PrEP but yet it is not the original one.

XXX: It is fake.

KC: Oh, alright, that you can be unfortunate and get something that is not original, something that is fake.

25: Yes, because you don't know that…

KC: Okay.

25: One can just sell for herself.

KC: Alright 26.

26: Plus, the other thing in pharmacy the price is high so you will not be able to buy.

KC: Yes, the issue of price can be a disadvantage.

ALL: Uum

KC: Alright, are there any other places that you can think of that we did not think of? What about in the community?

XXX: Uum

KC: Is it okay that in the community you will have like a house where you can go and get PrEP? Can it work?

ALL: Yes, eeh.

KC: 20

20: Iih this is not good in that I might go and get them then the person will move around telling people that [Clapping hands] she has taken the tablets. Eeh eeh she is now promiscuous [Others laughing]. Yes, and my husband will hear that, why did you take them, and I will start being beaten, and the marriage will be destroyed.

XXX: It will be destroyed.

20: So, community is not appropriate.

XXX: Maybe it is a secret.

KC: Alright, the community is not appropriate.

XXX: Of which the health facility is safe.

KC: Alright, so the community is not appropriate, but the health facility is safe.

XXX: It is safe.

ALL: Uum

KC: Right, we are now moving on to role play number two, for mai Bhobhi and mai Juru, it's you on this one. Aah, aah it's you.

XXX: Some of us have forgotten, we are enjoying Chido's story. [Others laughing].

XXX: You will forget.

KC: So, we are moving on to mai Bhobi and mai Juru, that's where we are now. Mai Bhobhi and mai Juru we said that 2 ladies are married right.

ALL: Yes.

KC: But one of them is unfortunate that her husband is having many girlfriends.

XXX: Uum

KC: So, she has started on her PrEP. She now has 6 months taking PrEP but she no longer wants to continue taking PrEP so she is having a conversation with her friend mai Juru. Let's hear why, what could be the reasons? So, speak aloud.

Role play 02

MJ: How was your day mai Bhobhi?

MB: It was fine how was your day mai Juru?

MJ: Fine. Why are you not happy today?

[Talking aside] Aah, this is not mai Juru, it's her. It's you mai Juru?

KC: Start again, start afresh.

MJ: Alright, why not happy amai Bhobhi?

XXX: Raise your voices.

KC: They are saying raise your voices. They are saying we are not hearing you.

MB: Alright.

KC: People from this side are saying they are not hearing you.

MB: Alright. Not being happy like this amai Bhobhi my husband is having many sexual partners. Haa he is being promiscuous.

MJ: Haven't you heard about the PrEP program being talked about from the radio my friend?

MB: I have heard about it and I now have 6 months using them, using PrEP tablets.

MJ: So, what are you thinking, these are the ones that are protecting people from HIV?

MB: Haa, I 'm thinking of not taking them anymore.

MJ: Are you taking them on time my friend?

MB: Taking them on time, will that affect anyone?

MJ: My friend you could just… don't stop taking them my dear. The way your husband is promiscuous you have to continue taking them.

MB: Uum, I think it's done, right.

KC: It's done.

MB: Uum

KC: Alright thank you. [Babies making sounds] So, mai Bhobhi and mai Juru are done with their role play, so, we now want to discuss their role play. So, firstly we want to know if there are ladies in these situations like mai Bhobhi's situation, that they are married but their husband does have other partners, do have girlfriends. Is it common?

ALL: Yes.

KC: Alright, aah if you say yes, you have to say I think situations are different. What kind of situations are they in? For example, how is it like in their marriages? [People talking in the background] 20.

20: Eeh, her husband may not be coming home.

KC: Uum

20: He can spend a week without coming home.

KC: Uum

20: When he comes home, he would like to have sex with you without using any protection.

KC: Alright, alright, ok. Is there any other situation, situation like… like mai Bhobhi? Are there any other situations?

XXX: May you kindly read again amai Bhobhi's situation.

KC: Alright, so, mai Bhobhi's situation, I said its mai Bhobhi and mai Juru and they are all in their twenties. They are all married but mai Bhobhi's husband have other girlfriends so mai Bhobhi thought that she is at risk of getting infected with HIV. So, she went to the clinic after hearing about PrEP on the radio, she went to the clinic and got initiated on PrEP. She now has 6 months taking PrEP but she is thinking of stopping PrEP she doesn't want to continue. So, she is having a conversation with mai Juru telling her that I started taking PrEP for these reasons, but I no longer want to use it and mai Juru is trying to understand why but the reason did not come out why she is no longer interested in using it. So, that is the role play so that's what we now want to discuss.

XXX: Eeh

KC: So, my follow up question is that do you think a married woman like mai Bhobhi, is it easy for her to decide that I now want to use PrEP? Is it an easy decision that she can decide that let me start taking PrEP? Is it easy for married young women?

XXX: It is not easy.

XXX: It is not.

KC: Why is it not easy? How is it not easy? What is challenging about it?

XXX: The husband might think that maybe you are the one who is being promiscuous.

KC: Okay. Alright, the husband might think that maybe it's you who is having extra marital affairs. Is there anything else that makes it difficult? [Silence]. Alright, aah, do you think what we can talk of as, what can be a barrier…the young women who are married what can stop them from using PrEP? Married young women, what can stop them? We have been talking about the adolescents who are not married now we want to focus on the married ones, what could stop them from using PrEP? 25.

25: What I think can stop them from using PrEP sometimes it could be the issue of their husbands because when you discover that your husband is being promiscuous and that's why you are using PrEP but the husband is not agreeing to that because he is doing it silently and he will never do it openly for you to know about it. But you just got to know about it and you saw it good to protect yourself but he might refuse for you to use it yet he knows what he is doing but for him to allow you to take them he might refuse.

KC: Alright, why do you think husbands do not want their wives to use it, why they do not want them to be on PrEP? Why do they not want their wives to take PrEP? 20.

20: Sometimes men just want to fix, they want to fix so that when he gets infected you also will be what…

KC: You will also get infected.

20: You must be infected as well.

KC: Alright, so that we will all be the same in the end.

20: Yes, so that he has a way of dying.

KC: Alright, is there anything else, is there anything else? [Silence] Nothing, alright, as we continue with the barriers, what has been said that a husband can stop you. Are there any barriers for married women in using PrEP, any other reasons? 24.

24: [Baby crying] One might be allergic.

KC: Okay, she will not be able to use it because of that. 25.

25: One might be shy that aah, since I'm married maybe I'm destroying my marriage by disclosing that my husband is promiscuous. I will tell someone that I' doing what, I'm now using it. You may be asked some questions like why you chose to use it, and it would seem like you are portraying your partner in a… Some ladies might just want to cover for their husband, yet they are wrong.

KC: Alright, keeping a good image for your husband.

25: Yes, yes.

KC: Alright, any other reasons? Alright, what can be the facilitators? Is there anything that can motivate married ladies to use PrEP? What can motivate them?

23: I just want to say I don't see any problem with PrEP, it is a good program and its nothing to be ashamed of, because you will be protecting yourself so that you will not get infected because if you don't use it you will be at risk.

KC: Alright.

23: So, I see no problem, it is a good program.

KC: It is good for one to be protected.

23: Yes.

KC: Alright, its ok. Do you think mai Bhobhi and mai Juru situations, especially that mai Bhobhi wants to stop using PrEP. Is it something that is common amongst ladies that after initiating on PrEP for about 6 months and then one would not like to continue using it, is it something that is common? You are nodding your head are you in agreement or you are disagreeing? [People laughing].

22: Haa, it can't.

XXX: Yes.

KC: Eeh

22: It could be that mai Bhobhi sometimes she might be bleeding or having other problems with PrEP.

KC: Because of PrEP they are having side effects.

22: Yeah

KC: Uum, is there anything else that for someone like mai Bhobhi after using it for some time but she is now thinking of stopping, any other reasons? You have talked about side effects, is there anything that can stop the continued use of PrEP? [People whispering] Uum.

XXX: I'm thinking that the reason for her stopping maybe she came across her friend and the friend asked her about it or the friend laughed at her. Or maybe there was some negative talk and she said aah I no longer want anything to do with it.

KC: I now want to stop it.

XXX: Uum

KC: Uum, anyone else, anything ese that can stop someone from taking PrEP? Being laughed at, side effects, anything else? [Silence]. Alright, uuh what can motivate or what can be done for someone who would have been initiated PrEP not to get to amai Bhobhi's situation of wanting to stop, what can be done for someone who would have been initiated to continue using it. What could be done? 22.

22: Just like mai Bhobhi if you have a problem like say bleeding problem she must go to the elders where we are getting the PrEP and report that I have a problem like this…this and get help.

KC: Alright, uum, 26.

26: For me it is like a question.

KC: Yes.

26: Let's say you are taking these tablets do you have to eat something first because sometimes you will be under pressure and you just take them, aren't there any side effects?

KC: No, all medication mostly some, mostly they say you must eat first, then take your medication. So, you are saying that maybe someone would be, taking them without eating anything?

26: [Interjects] Yes and just eat.

KC: And then encounter the effects.

26: Maybe the food could be little, in the morning only and one takes the tablets and doesn't eat anything for the whole day.

KC: Ooh. Alright, okay. Is there anything else to add on what we could do for people, one who would have started using PrEP to continue using it, is there anything else? There is nothing, alright. [Silence] We now want to move on to group number 3 the last one for the three friends, Peppa, Princess and Sky. Aah, 3 friends are at the same school, right. These ladies are in their twenties, and these ladies have their sexual partners. So, they have been selected from their school to come up with a PrEP program such that the program they will come up with must be preferred by adolescents and young women, right. So, we now want to hear from them and then we will go to our questions. At least we have covered a lot, and we are about to finish we are now doing group three for the 3 friends.

XXX: [In a low voice] Is it Peppa by the way?

XXX: Uum, you want to be told.

Role play 03

Sky: Peppa, iih, ladies there is a program that we heard of that is coming up, the PrEP program, for the prevention of HIV. Iiih, ladies I wanted to go and have it as well, how did you go about it for you to be part of it.

Peppa: *I wanted to come to you, to ask my dear, I also heard about it, I have never been there I wanted to hear from you ladies.*

Sky: *Aah, when I heard about it, I heard that there a phone number that is there, so we can look for it and what, and hear how to go about it because I know nothing because I don't understand my husband these days, my friends.*

Peppa: *That's it, ladies let's encourage each other and go, let's hear how it is done, then we what, ladies, so that we know how to go about these PrEP issues.*

KC: Horaiti

Princ: *Ladies isn't there anything that will, when we start taking the tablets will there be no things or effects that will affect us?*

Sky: *Iih, I don't think so, I think it is helpful.*

KC: Alright, thank you group 3, but I will ask you to do again because what I want you to show us you did not show us, right. So, you have been asked to come up with a program, I will give an example. We have been asked to come up with a program for ECD children to encourage them to come to school, right.

XXX: Uum

KC: So, we are saying we are now planning about our program. Our ECD program there must be a classroom at the school. The classrooms should be decorated to attract kids with pictures and other things, right. We want them to pay fees less than $10, right, we also want them to have some food for lunch at school or to be given a school bus that will pick them from home and drop them home after school. We are planning on ECD program, right. So, that's what I wanted you to show us that you are making a PrEP program for the adolescents and young women. So, what is the important thing that you want to be in the program that is what you are discussing that in this program we want to have this kind of PrEP. We want the people who will give us PrEP to be, not names or the pharmacy, or the health facility. We want the PrEP to be available in such places, that's what we…that's what I was expecting, that what I want you to bring out in your role play, so can you just start for about 2 minutes.

XXX: Wait [Laughing] let me read again.

KC: Alright, [Laughing]

[Babies crying and making sounds]

Role play 03 [Repeat]

Princ: Hi Sky.

Sky: How are you, Princess?

Princ: I'm fine. I have heard about the PrEP program that is being talked about, it is being said that it helps people on HIV/AIDS programs. So, I think if we can go and get these considering the way our boyfriends are promiscuous it is good in that we can be helped. But it is good in that we can get it from the health facility.

Sky: Is it only available at the clinic?

Princ: Not knowing if it is only available at the clinic. If it was possible they would make it available even in pharmacies, or even in schools at least it is better because at the clinic you can meet your parents, when you want to see that…when you want to collect PrEP, and you will be in for it.

KC: Okay, alright, thank you, right, so we are now coming together and discuss our last role play number 3, right. Have you understood our role play? Have you understood number 3 what we are supposed to do? All of us as a group did we understand?

ALL: Yes.

KC: Alright, so the role play is that there is need for a program or there is need for a PrEP program for adolescents and young women that's the program that needs to be made. So, firstly, do you think adolescent girls and young women can be able to come up with their own program on their own after being given an opportunity and being together at one place and asked to come up with a PrEP program of their choice, is it something they can do on their own?

XXX: Yes.

KC: Are they able to do it?

ALL: Uum

KC: Alright, uum. So, if they can do it, we now want us to come up with our own PrEP program for the adolescent girls and young women. So, the PrEP program you would come up with, what kind of a program would it be like? What important things do you think should not be left out in that program. Things that you say, this program must have this, and this and this. The PrEP must be available in such...such places and should be given by such…such people. What can you help us with to come up with a PrEP program? [People talking in the background]. Is there anything for us to come up with a PrEP program? What can we say is important for you? You are young women and adolescent girls who are here what do you think is important, something that you will say aah, if PrEP comes like this, I will take it? 25.

25: I think if it could come to us, and we are the people who wants to take part it might be easier for us because we understand each other.

KC: Alright.

25: We can understand each other. Eeh, our issues can be the same.

KC: Alright, so, the program must be led by your peers?

SOME: Uum

KC: Alright, is there anything else that you think is important?

25: Talking to people, someone who is free such that if I ask a question the person can be able to respond to me and I will be able to understand.

KC: Someone who is free to respond will help you talk about this issue.

25: Yes.

KC: Is there something else? What about where you can get the PrEP from?

XXX: Haa, pharmacy and clinics.

XXX: In pharmacies and health facilities.

KC: What you said, the places you mentioned before.

ALL: Yes.

KC: Alright, those are good places for you?

ALL: Yes.

KC: Moving on to, to, to, what I said that in Zimbabwe so far, the tablets that has to be taken every day, right.

ALL: Uum.

KC: On that one what kind of PrEP would you want? Let's say on the tablets, what kind of tablet would you want? Someone once said if it was possible to have a one-month dose, that's what I want to hear.

XXX: Three months what.

KC: That's what I want to hear.

XXX: Something that can be taken only once not that…

KC: Once per what?

XXX: Even in three months.

XXX: In three months.

XXX: In six.

KC: Once per three months, once per 6 months, anything else?

ALL: Yes, yes.

KC: Alright, let's say yes, 20.

20: I would like to ask that let's say it has gone to the pharmacy will we be buying or what?

KC: Usually in pharmacies anything that is put in a pharmacy is for sell. Everything that will be in the pharmacy is for sell.

29: Okay

KC: It's rare to get it for free. Uum, alright, aah instead of…let's say for example somewhere like here where you can get PrEP and get family planning services as well. You get HIV testing, pregnancy testing, would such a place be idle for PrEP distribution for adolescent girls and young women, would it be fine?

XXX: It is alright.

XXX: It is fine.

KC: Would it be good for you?

ALL: Yes.

KC: Why? [Papers flipping] Is there a reason? 25.

25: The easiest place is a health facility because there is everything, PrEP is being taken by someone who is negative, right?

KC: Uum

25: So, you start by testing for HIV and then you will be given your tablets, so everything will be there at the same place. Not that you have gone to the pharmacy maybe you are starting to take those tablets, yet you are HIV positive already.

KC: Oh alright. One stop shop that you just visit, and everything will be dealt with there.

25: Everything will be dealt with, and you will be given your tablets and go.

KC: And you will get your tablets there, it's okay. Do you think currently adolescent girls and young women are able to visit places where family planning services are being offered to get family planning services? Are they able to get family planning services? Is it easy for an adolescent girl or young woman to visit a health facility to get family planning services and get them?

XXX: For adolescent girls it is not easy.

KC: Yes.

XXX: For adolescent girls it is not easy.

KC: Why is it not easy for girls?

XXX: Because can you start taking family panning tablets when you are still going to school, it is not possible.

XXX: It is not possible.

XXX: It is not possible, people will be saying they are indulging into sex and aborting pregnancies, I don't know it is not understandable what you will be doing.

KC: Alright, okay, okay so you said it will not come out well for girls?

ALL: Uum

KC: Alright, we are almost done, right, so, my next question is that in this research that we are doing there is another study that we would like to do in the coming months, right. We will be doing the study with young women and adolescent girls like you. So, we would like your help in this study that we would like to do but we would like to do it with young women and adolescent girls who survives as sex workers.

ALL: Uum

KC: Where would we get people who can come freely without hiding it or being ashamed of being seen, where can we go and carry out that study? We would like to ask the questions like we are doing, we would like to ask them questions about different things, 25?

25: I think like here where we did this one is still good because you said you want people who are into sex work. There is no way you would call people saying come here all those who do sex work we are gathering here in the community or in Mbare by the flats. But here at the clinic no one will know that I have gone to the clinic, no one will know the reason for my coming here I would have just come, I just come across the program.

KC: Alright, so, a place like this clinic is fine? Are there any other places where we can get them?

XXX: Even in schools it is also fine.

KC: Even where?

XXX: In schools.

KC: Schools are also fine.

XXX: You just say that we want girls of such and such age.

KC: Alright.

XXX: Those who know about this.

KC: Alright uum, is there anywhere else we can go and do our study and get the girls who…

XXX: [Aside] Why are people standing up? Those who are standing up.

XXX: Let's stand up, what are they saying?

KC: They are calming their babies who are crying. [People laughing] Is there another place where we can get them? [Others still laughing].

XXX: Haa, I think that's the only place.

KC: Aa, alright. In that study at some point, we will ask not everyone, to those who will take part in the study to take vaginal samples that will be tested for STI. So, each person will take that sample by herself not by a nurse, we just teach them how to do it and they will take it by themselves. [The wind carrying some words away]. Do you think it is a good method, do you think they will be comfortable with that these young women, taking samples by themselves? [People laughing] Would they like taking their own samples? You will just be taught that you get into a room and do this, this, this and get your sample and enclose it in this paper.

XXX: Aah, taking your own sample, it can't.

XXX: It can't.

KC: It can't.

XXX: It is possible.

XXX: Haa. It is fine taking your own sample.

KC: Why, [People laughing] you will be taught.

XXX: Because sometimes one might be ashamed to be looked at.

KC: Yes.

XXX: One will be ashamed to be looked at "_+" [Words carried away by wind] people will just say I will do it by myself, yes but one will not be ashamed.

KC: So, is taking your own sample fine?

XXX: Yes.

KC: Because one might be shy to open.

XXX: One will be shy to be examined by the nurse.

KC: Okay, others is it fine taking your own sample? We are almost done, 20 [People laughing]

XXX: People are now holding babies and bags [Laughing]

KC: Alright, uum, so a place, alright one has taken her own sample it will be taken to the lab and get tested for STI, three STIs. The day that one takes a sample we ask if she wants to see the results of the tests. Those who would have said yes we will give them their results and those who would have said no will not get their results.

XXX: Alright.

KC: Those who…so how would we give them their results? How do we tell them about their results? Let's say the results are out, how do we communicate to them telling them that the results are out? [Baby laughing] how would we tell them? 18

18: You would have taken the phone numbers [baby making the baby sounds], like what is on this paper, if they are any queries, there is a phone number.

KC: Uum.

18: Even on that one you can as well collect phone numbers.

KC: Alright, their phone number and we will tell them their results.

ALL: Yes.

KC: Do we tell them about the results over the phone?

XXX: You don’t tell them over the phone.

XXX: No, you will call them…

XXX: Because they need counselling.

XXX: Come and we can meet halfway.

XXX: Because the results it’s like HIV.

KC: Oh, it is the same with HIV.

XXX: Yes.

XXX: Yes.

KC: We just adopt the same.

ALL: Yes.

KC: Alright, what about treatment services let’s say some have tested STI positive, they need treatment for STI, if an STI is not treated there is something that it affects in a woman, so it has to be treated. Where would you suggest for treatment services, places that you think are fine for the treatment of STI, for adolescent girls, which places are free for them to get treatment for STI?

24: In clinics.

18: In clinics

24: Hospitals.

KC: In clinics, 24, said in clinics and 18. Is there any other place where they can get treatment? Which clinics, public, private, which clinics?

XXX: All health facilities.

XXX: She said government.

KC: Government?

XXX: Yes.

KC: Why are government clinics good for them to get treatment there?

XXX: They will not require much money, usually the private ones require lots of money.

KC: Alright, okay alright. My last question and we are done, right.

XXX: Uum.

KC: There was somewhere where you read that there are other types of PrEP that are still being worked on like injection, like a right. So, so, far in Zimbabwe we do not have injections and rings but there was a time when it was approved to be used but it's not yet available you can't find it, right.

ALL: Uum

KC: But the ring was approved to be used here in Zimbabwe.

XXX: Uum

KC: Right, so ring the way it works, it is placed in the vagina. The ring will be placed and stay there for the number of months specified let's say one month with the ring. So, the ring will be releasing some medication that will protect against HIV for that time one will have it. That's the same with an injection, injection is more like the injections that we use in family planning that's the thing that will be used, so, those are some of the things that are still being manufactured but it is all PrEP, so, looking at it now do you think …

XXX: [Baby crying] sorry, sorry.

KC: What do you think about the rings and injections on PrEP? Are they good, do you think having them is good? Is there any advantage?

XXX: Yes, they are good.

KC: 18.

18: An injection, aah it is good because you will not forget taking it.

KC: Uum, uum she said you will not forget taking it if it is an injection. Anything else what are the advantages of a ring and injection? What about the disadvantages? Are there any disadvantages that you think? Is there any disadvantage?

XXX: Will the ring not affect the reproductive system?

KC: Alright, so those things like rings and injections are not yet there so there are still doing research on them to find out if they have side effects like the tablets that were researched already, and it is known that they have these side effects. So, a ring and injection are still being worked on to find out if they do have any side effects to the recipients. Uum is there anyone with something to say on disadvantages?

[Talking at the same time]

XXX: Will the tablets not make you gain much weight?

XXX: On the side effects.

XXX: The side effects were not talked about on tablets.

KC: Alright, for the ring we are yet to know about them.

XXX: The real tablets.

KC: Alright.

XXX: That we have learnt about today.

KC: For the tablets, the common ones that usually happen are that one may feel nausea, body weakness and headache.

XXX: [Interjects] On a daily basis when you take them every day.

KC: Let's say first week you start taking them.

XXX: Alright, aah alright.

KC: But they are some who used PrEP who are saying it will go away, and you will be back to normal. You will have side effects for the first time. Uum, alright. So, I'm done with the questions. Is there anyone with a question, anything to add, anything to ask from what we have been discussing. There is no one?

XXX: Yes.

KC: Alright, if there is no one, thank you for your time thank you for the discussion we had. Our discussion is over, so we have some refreshments so, we will give you and you will eat when you get home so that we will not continue gathering as a group due to COVID issue. So, thank you for your time our discussion is over.

XXX: Thank you.

The End.
