# Supplementary material for: Perceptions of pre-exposure prophylaxis among sexually active adolescent girls and young women in Zimbabwe–A qualitative study
Source: PLOS Glob Public Health. 2025 Dec 2;5(12):e0005396. doi: 10.1371/journal.pgph.0005396 (PMC12671731; doi:10.1371/journal.pgph.0005396)
Supplement: S1 File — (ZIP) [file pgph.0005396.s003.zip › S1_File/AGYW-FGD 04 -Translation.pdf]

AGYW-FGD 04- Translation
Facilitator: KC
Note Taker: SM
Date Of FGD: 11/02/2022
Age group: 20-24 years
Translator: KC

KC: So, as we start our discussion I just want to say thank you so much for taking the time to have this discussion with us. As you may remember, my name is Kudzai and my colleague is Sharon, today we have a discussion on your views of PrEP which stand for, Pre-Exposure Prophylaxis, to understand PrEP acceptability, reasons for the low numbers of people who take up PrEP, issues of how well it is taken amongst adolescents' girls and young women. This will give us important information on how we can develop new ways of providing PrEP services to improve the information, sorry to improve the number of Zimbabwean adolescents and young women who take it up and remain using it among these who need it and are willing to use it. Our discussion will take between one and ho… one hour to half and hours to complete. Uhm, this section, as we have said before we will have both role plays and discussions on the study topics. For the role plays we will ask you to split into 3 groups, each group will act out a scene on how adolescents' girl and young women will typically react to scenarios related to PrEP and perception of HIV risk related behaviors. Each group will be given a description of the scenario that they are supposed to act out and we have only 3 minutes to agree on the content of the acting scene. We will then ask the group to in turn act out their scene which will be followed by full group discussion that explores what has been acted out. Uhm so, remember to refer to each other to the names and when you want to say your point you first say out your number to say, number 30, then you go on to say out your point, number 34 you go on to say out your point, right. So, as we are starting our discussion, can you… briefly tell me if you have ever heard of PrEP before? Have you ever heard of PrEP and also feel comfortable to use the language that suits you or that you are most comfortable with, English, Shona just changing as we go, it's still fine. Have you ever heard about PrEP before?

Some: Yes.

KC: Alright, so for those who would have said yes, you say yes, and you say your points. Yes, I have heard about PrEP it is this and this and this, 30?

30: I have heard about PrEP it is used before uhm or rather during the period that you will be exposed to HIV [Background noise]. So, maybe you will be going to a party…you would want to go to a party, and you know something will happen.

KC: Uhm.

30: So, you can take PrEP before.

KC: Okay, alright, uhm, she said she heard about it. It is used before you engage in sex, is it any kind of sex that you need to take PrEP before?

30: Yeah, anal sex. It was mentioned that there are days when PrEP is most effective, anal sex, it will be effective in 7 days.

KC: Ok.

R: And then if its vaginal receptive sex 21 days to 30 days.

KC: Alright, uhm are there any other? Uhm, 33.

33: I'm not quite sure about what I have heard, but I heard that with PrEP you can only access it through a prescription.

KC: Alright, yes. That is very correct, so far currently PrEP you can only take it when you get a prescription for you to get PrEP. You can't just walk in and say I want PrEP and you get it without having any written prescription for you to take it. So, that's very correct. Uhm, any other views, any other points on PrEP that we have heard? [Silence] So, others have you ever heard of PrEP before?

ALL: Yes.

KC: You have all heard about PrEP?

ALL: Yes.

KC: You know about PrEP?

ALL: Yes.

KC: Now tell me, what do you know about PrEP? [All laughing] You have all said yes, yes, 34.

34: "_+".

KC: Raise your voice.

34: I am in a sex workers group, so they were talking about PrEP saying I have forgotten to take PrEP what should I do, so that is when I started reading so in that group there is a doctor.

KC: Hoo, ok.

34: So that doctor is the one who would say aah no go to the hospital "_+".

KC: Ok alright.

34: That is when I started knowing about it.

KC: Alright.

34: Doctor would encourage people who would have started that you guys you should go to the hospital.

KC: Alright, so the group I just uhm, what is the purpose of that group?

34: That group has female sex workers, and they empower each other.

KC: Hoo ok.

34: That as you are doing your work you must do something, at the same time. You will be vulnerable to some diseases so that doctor is the one who helps them and then if they have something they need, he helps them. People speak and the doctor assists them then if there is something that he would have been asked, he responds and then we…

KC: Ok, alright, thank you, 34. 30 were you raising your hand?

30: Yeas, I also heard that PrEP you can rely on it even to stop using condoms because it's that effective.

KC: Ok, it's that effective, ok alright. So just to add on what has been said by 30. So, in terms of reliance, relying on it is 100 perce…it is very effective if used consistently. Adhering to what you would have been told at the hospital, but now when you are using PrEP you are not supposed to stop using condoms because there are other things that condoms protect you from, but PrEP doesn't protect you from like the STIs. PrEP does not protect from the STIs. Pregnancy PrEP doesn't protect from the pregnancy so using that in combination, PrEP and condoms it will be very effective you are also protecting from HIV and other things that might come along with having sex without condoms. Uhm, is there anyone else whose hand was raised 37?

37: Uhm, I once went for this other workshop, so they were talking about ex…about sex issues and during that discussion on the issue of PrEP they were saying uhm, it also has like the…the disadvantages that it has.

KC: Uhm.

37: For instance, some can develop kidney problems, but they were saying its quite rare but it's something which is also possible.

KC: Yeah, very true, so PrEP has some associated side effects, so they include the dizziness, the headaches, the nausea. And some might experience them the first week that you have started using it that's when they experience some of those things but as times goes

on, they will clear and go away. Ok, are there any others with something to add? [Silence] Alright, what about in terms of the different forms of PrEP, do you know the different forms of PrEP that are currently available right now, currently available maybe starting from Zimbabwe? Forms of PrEP that are available in Zimbabwe, what do you know, 30?

30: Here in Zimbabwe so far, we have the tablets, then the other ones that are not yet available are the vaginal ring.

KC: Ok.

30: It is said it will be releasing some medication and the injectables.

KC: And the injectables, very correct so in Zimbabwe we only have the oral PrEP which are the pills. Here…have you ever seen the pills? Have you ever seen them?

ALL: No.

KC: You have never seen them?

ALL: No.

KC: So, I brought along a PrEP packaging this one, so this is the box, this is how it looks like, this one so, the container for the oral PrEP. So, to open this you need to pull down and open, turn down very hard [Sounds as interviewer tries to open PrEP container] Right, so this one is too sealed but I'm going to open for you so that you can see the… [Sound of table moving]. So, there is this dessicant for storage so, on the other… other… PrEP pictures, so see there will be blue if you have ever seen other PrEP pictures, you will see they will be blue. So, this one, this one that's the oral pill so that's what we have currently in Zimbabwe, that's what we have, we don't have anything else. But then it was mentioned the vaginal right, so the vaginal ring I just printed the picture so that's the picture of the vaginal ring. So, this one is currently in Zimbabwe it has been authorized by the regulatory, those who authorize the use of medicines in Zimbabwe. So, this one has been authorized for use in Zimbabwe but it's not yet available like the oral PrEP right. And then the injectable here it does not yet have the authorization, it's still being for…uhm formulated, researched being conducted but there are other regions where it has been approved but in Zimbabwe it is yet to be approved the injectable, so that's the form we have currently in terms of PrEP right. So

now I now want us to split to do our role plays, all the three of them. So, for our role plays I will ask you…we are 9 in total, right.

ALL: Uhm.

KC: We are 9, so you will be three, three, three, three in a group so may I ask you to group yourselves into three, three, three. So for our role plays, I will briefly read them out then I will give you the explained role plays.

Role play 1

So, for first role play it is between two friends, it's for Chido and Koko. Chido is 16 years, she is having a sexual relationship with an older man who is aged 50 years, who is aged 50s and above. So Chido has started taking PrEP as she is worried about contracting HIV. So, she thinks of her friend Koko who is 19 years as well whom she thinks she is at risk of getting HIV from her sexual relations and she suggests to Koko that she also takes PrEP. So, we want you to do a discussion between Chido and Koko. Chido telling Koko about PrEP so that she also takes PrEP because she is having relations that can put her at risk of getting HIV. So, we want to hear Koko's response on how she responds to that offer in relation to that it is something that can be happening in real life. That in real life if someone is approached with such an offer by her friend, how will they react on the issue of PrEP.

Role play 2

So, the second one is between 2 ladies as well, but these ones are young married women. One is 23 and the other is 21, both they are married, now their names are… one is mai Bhobhi and the other one is mai Juru so mai Bhobhi has got husband who has a habit of having girlfriends quite a lot of times and she is worried about getting HIV. So, she heard about PrEP on radio, being advertised and she went and got her PrEP. She now has 6 months taking PrEP right, but now, she is now thinking that I do not want to take PrEP, she wants to stop using PrEP. So, we want a role play to be done, a discussion between mai Bhobhi and mai Juru. Mai Bhobhi explaining her reasons why she wants to stop using PrEP when she has been using it for the past 6 months.

Role play 3

Then the third one is amongst three friends who are in school in their 20s and these ones they have got sexual relations with their partners, and they go to the same school so these ones there is, Peppa, Sky and Princess. So, they have been selected at their school to design a PrEP program for adolescents girls and young women that suits them and will be preferred by the adolescents girls and young women. So, on their PrEP program we want them to list that the PrEP program that they think will be preferred and improve uptake amongst adolescents girls, what does it look like. Saying that, aah we would want it to be given by a person who is like this, it will be accessed at such places, where it will be accessed it will be like this, things like that, that is what we want in role play number 3.

KC: So, those are the three scenarios so for now you are breaking and get into the three groups. You discuss and then we come back, we resume with our discussion. Let us give you the role plays but you can start splitting. [Noise people splitting into groups]

[Role plays discussions]

KC: Alright, now we need to come back and resume our discussion right, you are now ready right?

ALL: Uhu.

KC: Ok so you can go back and sit where you were sitted before and you…those who are acting out the role plays are the ones who will stand up and do the role play, so you can go. If you want you can go back where you were sitted before or if you are comfortable with remaining sitted where you are, it's still fine, no problem. Right, so group 3 we are now continuing right. So, when I asked in the beginning, I noticed that there is a part that I had left out when I was asking about if you have ever heard about PrEP. Right all that information that you were giving me about PrEP, where did you hear it from? Where did you hear it from? There is someone who mentioned that they heard about it from a Whatsapp group, one said from a workshop, that it was…uhm…it was during an HIV/AIDS workshop?

XXX: Yeah.

KC: Alright, others the information that you know about PrEP, 33, where did you get it from?

33: It is when I was doing research for a NAC quiz, so you get a… [Interruption]

KC: Oh, okay alright. Others PrEP information, 32?

32: In high school we were in a club for AIDS, NAC whatever, so yeah.

KC: Ok school clubs, 30?

30: I was preparing for a debate at a public speaking competition hosted by NAC.

KC: Ok, so NAC mostly, yes. 31.

31: I heard about it from my friend, explaining what happens in their group to say that is what is what…like a group for…for the sex workers.

KC: Ok.

31: Yes, so that is where PrEP was being explained.

KC: Ok, alright, uhm. 30, 29?

29: I heard from SAY WHAT when we discussed the blessee blesser issue.

KC: About what?

29: Blessee blesser issue.

KC: Ok, the blessee, blesser issue. Ok which issues were they?

29: Issues of children who are in institutions and are having blessers so some of them are taking PrEP to protect themselves because those blessers do not want to have sex using condoms of which some of them…most of them they are infected.

KC: Alright, what are blessers? [People laughing].
[Inaudible background discussions]

XXX: They are the people…most of them who are in tertiary institutions they take…because some of the girls who engage in those things will be people who are…or who have financial problems then those guys they take those ones. You are to satisfy them sexually and they provide for you financially.

KC: Ok, ok those are the blessers, ok great. Right, so we are now moving, we are now going to, role play number one, the one for Chido and Koko. So Chido and Koko's group can you…the ones acting out, the two who are acting, the Chido and Koko may you stand up and do your act, raising your voice for our recorder as well.

ROLE PLAY 1

Koko: Chido my friend [some whispering]

Chido: Koko my friend I have an issue; you know that sometimes we go to those parties that we attend.

Koko: Yes.

Chido: Plus, you see our blessers, right these blessers do not want to use condoms. You know that right, but these people are they okay healthwise?

Koko: Aah I don't think so but they might have it.

Chido: I have started using PrEP my friend, I was thinking of you saying to you Koko you should take PrEP and protect yourself because these people…we want the money but I don't think these people are okay healthwise.

Koko: Aah my friend these PrEP, are they not the ones that will make us end up having big tummies in the end.

Chido: But your health is important PrE…PrEP does not have side effects, its safe.

Koko: But where will I take it because you what my aunt works at the hospital.

Chido: No, it's taken from many places, it's taken from many places because your health is the one that is important because…

Koko: Alright Chido, I take the PrEP and I go home with it, where will I keep it, what if its seen?

Chido: Aah, PrEP you can move around with it, even in your bag, wherever you are you can move around with it.

Koko: Hey, will I not seem like I am sick.

Chido: No, you…it does not mean that you are sick but protecting yourself from HIV because you want the money, don't you want the money?

Koko: Aah I want the money.

Chido: Yes that is why I am telling you that if you want money then do what…let us protect ourselves. You want the money but you…well if you get sick, if you get sick, my friend. If you get sick you will die, if you get sick you will die.

Koko: I will think about it Chido.

Chido: There is nothing to think about.

Koko: Where will I keep it, the collection point? What about people, what will they say… [Interruption, speaking at the same time]

Chido: No, you are thinking about other people, so you want to get sick?

Koko: No, I don't want to be sick.

Chido: Yes, now protect yourself my friend, protect yourself.

Koko: But just looking at him.

Chido: Aaah that older man, you think it's just the money but ahh, on that one…eeeh… [laughing]

Koko: What then can we do when we want the money.

Chido: You want the money but protect yourself my friend.

Koko: Aah, alright my friend, I will think about it.

Chido: I have mine in the bag, I move around with them. I don't want to get infected.

Koko: But what you are saying makes sense, I thought of that time when I went to Gweru.

Chido: You see that time when we went to a party, where did you wake up at drunk? Now protect yourself.

Koko: Alright I will think about it my friend.

Chido: There is nothing you can think about, tomorrow we will go. Isn't it there is a party, that one "_+".

Koko: So see you tomorrow.

Chido: Exactly, that's fine my dear.

[People laughing and clapping hands].

KC: Thank you so much Chido and Koko's group, right, thank you for the role play. Thank you for the ideas that you have brought out that are quite many, right. So now we want to discuss Chido and Koko's role play right, starting with are there people who are in situations like Chido's that they have sexual relationships with older men in real life? Are there, young adolescent girls who have sexual relations with older men, around 50, 60, 70 going on?

ALL: Yes.

KC: Uhm, say there are there who does this, how does it happen. The details are the ones that we want to know, 30?

30: These girls who have relations with older men sometimes what happens is that you are told that I have a wife at home, I am not going to marry you. I just want to have fun with you, so you go to some lunch dates, sometimes you go to Vic Falls and sleep there having fun. Eeh you do not get to be part of the family, those are some of the scenarios because you have been told that I will not what…marry you.

KC: I will not marry you.

30: And when you have been told I will not marry you, you can even tell him that I am also seeing someone, they don't have a problem with that as long as you are offering each other the services. You are satisfying him sexually most of the time…most of the time, most of them they would want not to use what…protection.

KC: Alright, ok.

30: And then there are others who wants to use protection so if you satisfy each each. You will be giving him sex and he will be giving you money and spoiling you, going on like that.

KC: Ok, uhm. Others, 29.

29: Yea, the adolescents are having relationships with older men who are around 50, 60. Some are doing it because of peer pressure.

KC: Ok.

29: It's like we are living in a world that evolves around social media. We see a lot of things on social media, the iPhones that are trending, iPhone 13, iPhone pro whatever so someone might be coming from a poor background where the parents can't afford to give her such things. They just have the money to send her to school, she does not have the trending clothes but when she is at the campus, she wants a lifestyle. She is coming from a prison-like lifestyle, but she wants to live a champagne lifestyle.

KC: Ok.

29: At the end of the day that person it will affect them such that they will look for some blessers just because they want their financial needs to be fulfilled. So, at the end of the day if the blesser gives you some US dollars that you will be flashing around. There is no way you are going to have sex with him using a condom because at the end of the day he is saying I am giving you raw money so you must give me raw sex.

KC: Ok, ok.

29: So that is what makes most girls be HIV positive. Some then know there is PrEP, they are going to the clinics and say out their issues. They get a prescription to get PrEP, but some do not even know that there is PrEP. I did not know about it; I knew about it through SAY WHAT. Maybe if I did not join SAYWHAT, maybe I would not be knowing that there is something called PrEP.

KC: Ok.

29: You should be doing what you want because you have hired a…contract because these men you are not the only one, they are dating. He has a wife at home, you are a side chick [girlfriend]. You do not know the side chicks that he has had, currently how many he has, you don't know his status if he is positive or negative.

KC: Ok. Alright others, 34. I will come to 33.

34: And then that issue, what is happening is that the issue of people cheating. [People laughing] That ladies say it's better to cry whilst eating rather than crying without any benefits. [People laughing] The person does not love you; he gives you heartache and staff, but you will be getting money. Then you date the young ones who uses you, he does not give you anything and he dumps you so that's another issue.

KC: Ok.

34: And then another issue that happened where I stay at number XXX. [Saying out the address]

KC: Do not say the address.

34: Ok, it's called the car park.

KC: Ok.

34: Like at that house many cars come there honestly, I don't want to lie such that there will be a competition. Such that ladies say if they go out right, they don't take hours, maybe its 30mins and she will come back counting $600US.

KC: Ok.

34: And then the cars come looking for the young ones, they say they are the good ones, and they ask for an outing. Some have the links with the matrons there that they say we are asking for your children; they leave them some money.

KC: Ok so it's a house setting?

34: Yeah.

KC: Where students stay?

34: I don't know what we can call it, students stay there. That line from the corner in the evening starting at seven there will be cars but usually they will be parked at our house. So, I heard one saying guys we cannot have money, let us go out in the evening sometimes, around seven you will find someone.

KC: Ok, alright so they will be parked looking for those who want to go out and they go out with them and come back.

33: Yes.

KC: Ok, interesting, 33.

33: My comment is that the normal scenario is whereby you are gaining something as a lady but then these relationships have gone normal to the extent that there is someone out there who does it without getting any benefits. Such that you will say aah is she not the one with the blesser but what is she gaining.

KC: Ok.

33: To say what exactly will be happening.

KC: There are no benefits.

33: She is not at all benefitting.

KC: Ok.

33: And also it comes to a situation at the universities whereby in our classes we are mixed older ones and young students, and trust me sometimes these men can be very kind. Seeing that this one is not benefitting anything, but she is head over heels.

KC: Ok.

33: And because things… [Interruption].

KC: So, its genuine love?

33: Yeah, and because things like that you cannot share that you see that colleague of ours, the older one we are in a relationship because the moment we meet with him they expect me to say to them I am benefiting. So, when you find yourself in a situation that you are all there, you are head over heels, but you are not benefitting, you don't share.

KC: Ok.

33: And when you don't share it means that people will not share with you some information of saying you know you can try PrEP and what, what, what. So, because of such scenarios that's what makes most people to be at risk because they will be hiding the reality because if you are dating an older man the next thing, we expect you to be rich.

KC: Uhm, ok, okay, 30.

30: Also, there is an issue that was discussed in one of the lectures, in that lecture the lecturer said the absentee fathers, those ones…Fathers who are not present or if they come, they will be like a lion, you will be afraid of him.

KC: Uhm.

30: Those are some of the people who make the young ones to…to go to the blessers because if she sees a blessers, she sees a father figure.

KC: Ok.

30: So, her mindset is…is now seeing her father in that blesser. The blesser gives her comfort and does so, so, so.

KC: Oh, okay he gives her the love that she never received.

30: Yes, she is now getting…like of course we need our fathers, that's very true. Although you would have grown up with only a mother, something will be lacking.

KC: Yes.

30: So, if you see that sugar daddy you then see your father. You now enjoy being around him so that is when you end up staying without what…without any tangible benefits.

KC: Anything tangible.

30: Of which the blesser issue is that you will be…accessing, it's access to resources. [People laughing]

KC: Ok.

30: Plus, it is said genitals are an assets, using your assets to…because you have an absent father. That older man now acts like a father figure to you, you get used to it.

KC: Ok, 34.

34: And then there is another issue that we heard in one of the lectures like issue of financial abuse.

KC: Uhh.

34: That parents are abusing their children financially that for example when they come to the university you don't provide them with what they want. Like let's say food, there is a lady who ended up having a blesser because they were not giving her money for the rentals and money for food.

KC: Ok.

34: And then so it's called financial abuse because you know your parents they are able but they sort of ignore, like saying you are now a grown up, you eat a lot, what, what and staff.

KC: Ok, alright, 33.

33: Also, I would like to comment on, another thing that will affect us, you know that statement right, the issue of saying I want someone who is mature.

KC: Ok.

33: I think it's something that is trending, to say when you are still as girls you just say that aah those aged around 29 that have some immaturity.

KC: Ma 2000 [chuckles].

33: But when I am with an older guy, exactly but at the end of the day you did not last in the relationship except putting yourself at risk.

KC: Ok, alright. There is an issue that was mentioned that the blessers they don't want to use condoms when they are in these sexual relationships, why is that so? Do you have an idea why they want to use condoms in these relationships? 35.

35: Alright, uhm when it comes to this issue there is this statement which says…they give an example of a sweet. They say if you eat a sweet in its wrapper the taste is different than when a sweet is…

KC: If you remove the wrapper.

35: If you remove it from the wrapper so they opt to go for it raw than to use condoms because they say the pleasure is a bit different.

KC: Ok, is there anyone with…yes 27.

27: I also think the other issue is uhm, I am giving you money. My money, I am not giving you wrapped in a paper, so you are supposed to give yourself to me not wrapped in a plastic.

KC: Ok, alright.

KC: Ok, the maximum value, 28.

28: I think I am supporting what 27 has said that it's that entitlement that I bought you. You are my possession so you should not come and tell me what to do because I am the one giving out the money. You are living this lifestyle and then you come here and tell me what to do.

KC: So, no terms and conditions here.

28: Exactly.

KC: Ok, 31.

31: Ok there is an issue that these old men it was said that…this statement of saying if you sleep with the young ones…The young ones right, they say they look younger, they do not age quickly.

KC: Ok.

31: So a lot of men they don't…they don't want what…they don't want the condoms because they want…because they want to maintain their youthful look.

KC: Maintaining the youthful look [People laughing] 30.

30: I can say that statement was also mentioned by a young man not the older men. He said like the heat in the vagina if you put on a condom you take time to feel it. He said that heat is the one that excites them.

KC: Hoo.

30: So, they condom is not good. [Laughing]

KC: Ok, alright, ok 35, do you want to say something?

35: I also have a contribution that is different, uhm, I feel like these girls for the sake of saying I have a blesser, I will get money and stuff. I feel like very few of them bring about that issue to say let's use protection. They just go for it without asking any questions because what they simply want is what…

KC: Money.

35: Money.

KC: So, they will not negotiate for safe sex. Ok, alright, uhm so besides where it was mentioned in the role play that Chido might be at risk of getting HIV. How is Chido at risk, where is the risk in such relationships, 31?

31: Ok, as friends that this one dates an old man right, there is a risk that they will be together wherever they go. Since they are friends, they share a lot of what…a lot of things so she will be a risk because she may want the services that the old man is offering to this one.

KC: Ok.

31: Yeah.

KC: Ok, that's alright. Its ok. What about Chido who is in a relationship with an older man, where is her risk of getting HIV? 30.

30: The old man has…is now old, let's say maybe he started engaging in sexual activities at the age of 16. By the time I will meet him, he will be around 60, there is a high possibility that maybe once or twice he slept with someone who is HIV positive, and they infected him.

KC: Ok.

30: So, I will be at a risk of what…of getting infected.

KC: Of getting infected with HIV, alright.

30: And I am not the only one.

KC: Ok, multiple partners.

30: He will be having others.

KC: 28.

28: I think also blessers most of the time they are promiscuous. You will see someone who has just dropped off this person from the car. You can see the same blesser following another lady whom he has just seen but because of that money they have that power of saying why should I just have one girl when I can go to this one and they will come because they want the money.

KC: They want it, ok. 32.

32: Like I wanted to say that they talked about the parties right, those friends. So at those parties they can, I don't know should we say impromptu, I don't know…something. Things that are unplanned such that you can hook up with someone, but you do not know their background. You have just hooked at a party, and you are having fun or whatever. You now go together; the rest is history of whatever would have happened there.

KC: Ok.

32: So yeah, as friends that is what they were telling each other that we need to take PrEP because we are at risk we go to parties.

KC: Ok, alright so at such parties what will be happening? 27.

27: I think on the issues of the parties; the issue is on the alcohol. The issue of drugs and staff because usually people who are intoxicated do not think. They just follow their

needs, if you feel you want to have sex, you have it, you will not be having any protection.

KC: Uhm.

27: So, on that one HIV is quite easy to get.

33: I wanted to comment on the issue where you have asked that what about this one in a relationship, where is her risk? When we are young right and you are in relationship with an older person, it's not easy to stand and ask that person that hey do you have HIV or let us go and get tested. You will be afraid of him sort of so you will not feel…it's like "_+" with a boy but you will be afraid of that man saying what if he says this, what if he says this.

KC: Ok.

33: So, you don't have the freedom.

KC: You do not have that freedom to negotiate on such things, ok. 34.

34: And then the other issue is about people who have a system of testing for AIDS with their eyes. [People laughing]

KC: Alright.

34: It is just saying aah this one does not have, haa this one does not have and then of course sometimes one will be having a healthy skin but even if they do good things, but he will be infected. Someone who has money, most of the time it is not seen that he has it.

KC: He has it. Ok, 27.

27: And also, I wanted to add on the issue of the parties that there are some guys who come with the intention of raping because he can say today I want to spike her drink with such a drug. Even putting some drugs in the food without you knowing.

KC: Ok.

27: So, you will see that when you get drugged you can wake up not knowing what have happened of which the person would have done whatever they want and finished.

KC: Ok, alright so on the point raised by 27, from our previous discussions that we have done so far there is a point that is continuously emerging, and we want to find out what you think. Uhm the group that we first had was a combination of 15-19 and then 20-24 right. So, they were saying the blessers are there and most adolescent girls and young women most of them are hooked by the blessers and the…there is a point that emerged

that most of these blessers have cruel intentions of wanting to infect HIV to these young ladies. From another group there was a point of saying men…it was with married women now, the second group. They were saying men in these marriages they would want to punish you and make you get the HIV that he would have got so we want to ask why is it men…is it men who want to punish women by deliberately infecting them with HIV? Why is it that, what do you think? Why do men do such things? 29.

29: Uhm some men they…not saying men specifically but some people they say I was infected so I will not die alone, I will die with what…with others. That is why most people who test HIV positive some of them do not accept it easily. They will say aah I now have AIDS; I got it from someone that I don't know. I am going to infect mine because it is not my fault that I am sick, so I must go down with other people.

KC: By infecting others?

29: Uhu.

KC: Ok, 27.

27: I want to add on what was being said by 29 that it will be an issue of bitterness that someone is bitter. They would have started being innocent, eeh I remember this uhm confession that I saw in a confessions group. So, this lady was saying that I started out innocent and staff, so she was saying I am very bitter to the other gender because I ended up being infected unknowingly. So, she is spreading that bitterness in trying to feel better through spreading that bitterness.

KC: Hoo, ok. Alright, ok, 30.

30: You know if its people who are "_", if you get infected when you say you are dating or we are…Then you start thinking of telling me, it might be hard for you because you do not know how I will accept it.

KC: Uhh.

30: Will I understand you or I will…will start stigmatizing you maybe I will tell other people who are around you. I gang up and I will hotspot them. That is stigmatizing, so that is the other issue, [people laughing] and if you are man your virility…Yeah here in the African context you take pride in that, that aah I am a man I made her cry. So for me to start relaxing and say let us use condoms or protection because I am now sick it might be a problem.

KC: Hoo ok, we get it. Alright so moving on right we now want to move to the next question that is it common, is it a common thing that adolescents' girls and young women share their health decisions with other people? The health decisions that we are talking about is getting tested for HIV, I am sick with an STI, do they share their health decisions with other people? I will start with 28.

28: Uhm, it is not that common because they fear being judged that you want to go and get tested for HIV, what are you really doing in your life. What is happening, why do you want to take PrEP like someone would want to judge you that how is this person, what are you doing in your life.

KC: Ok, alright, 34?

34: And then the issue that is there is that older people like our parents, people in the society the elderly ones like our parents for us to be open to them, we cannot because they start scolding us. For you to tell your mum no I have an STI… [someone coughs] you cannot open up because of the scolding that eeh you are a sex worker, what, what, what and then sometimes they don't even know what has happened, sometimes one might have been raped or to share stuff like that.

KC: Ok, 30.

30: I have friends whom I am comfortable to discuss with, those friends especially the ones not from church. The ones from this college because here we understand each other; I know that such things happen right but for those from the church I do not do that.

KC: Uhm.

30: They will say hey sister are you not the one who sings in the praise and worship, you are now being judged, you are being labelled. You are doing this, this, this but they do not know how I ended up doing those things and why.

KC: Ok.

30: Yes, they will start judging me but if it is here I know that it is a possibility that it can happen. One day you might be unfortunate, not really being unfortunate but one day…they say some things we get involved in them and some get involved with us so I don't know that day something might involve with you or might make you get involved with it.

KC: Ok.

30: That will make you want to take what…PrEP or what…so there are some friends with whom you can discuss with.

KC: That you can discuss with but there are others you cannot discuss with like the ones from church, 35.

35: Alright on this issue I would like to say in the African society, African communities uhm I think the parents and the people in it are still backward. They do not want to believe kuti there is a generational gap, there are changes that are happening so for me to open up and talk even like to my parents or to my close relatives I can't because one they will start to judge me. They will discriminate me and staff, and it would be, it won't be good for me and on the side of friends I think that one is comfortable in telling someone whom they are in the same category with.

KC: Ok.

35: Because she knows that this one does not judge me because we are in the same group.

KC: Yes, we are the same.

35: Because if I share with number 34, she is my friend, but I go out with number 27. If I tell number 34, I don't know maybe one day we are going to have a quarrel, and you know what happens when people have a quarrel.

KC: Secrets will be disclosed.

35: Every secret comes out [People laughing].

KC: Ok, alright.

35: So, people will just keep their issues to themselves.

KC: To themselves.

35: And will affect them actually that's why people will start act like bitterness, suicide, psychological, suicide and staff.

KC: Ok, 32?

32: Uhh I wanted to say…it was not like its HIV related but uhm that person got like an infection, vaginal infection.

KC: Yeah STI.

32: So, she told…I call them best friends those people, they get along. They are young mothers so, so, so she was not honest with her friend that I have an infection, do you get me. So, like she was in the outskirts, so she wanted medication, and she went to the

hospital, and she was told go and buy medication, it was prescribed for her. I said to myself she was not told the truth; it was said the child has a ringworm. Aah, I rushed to the pharmacy, and they asked about the condition what, what. I told them a child has a ringworm, and I came back home, and lady came asking for the medication. She said iih sorry, it was like…aah she did not want me to know about it, yet she was afraid of being judged by her friend.

KC: Ok.

32: I had to go back to the pharmacy saying to the pharmacist sorry that mother did not want to say the truth, may you please change this medication for me. That medication was expensive, I had to top up so it might be hard to disclose such things, that are related to…I don't know it's just fear that how will someone judge you.

KC: Ok.

32: That how did you get it.

KC: Ok, fear of being judged. 33.

33: Yeah, just to buttress on that you will see that even an issue of sharing simple things like aah my dear do you go for HIV testing, let's go and get tested. You know if you tell…there are people who are still…of course these days our mind sets are changing but there is someone if you say to them aah lets go and get tested for HIV, they will ask you iiih did I have any sex? Or what because at the end we all want people to see us as people who are holy, who have never done what…

KC: Done anything.

33: So, it really makes it hard, you must first study that so number 32 what do they do. If you see that ooh aah we are in the same squad that is when you say aah where do you get such and such.

KC: Hoo, ok. Alright, alright. 31, 34, 29 and we move.

31: Aah looking at our society it usually talks about sex when one is married, sex before marriage it's not yet that acceptable. So, it makes a lot of ladies…for her to go and get tested she will be told so you are having sex. Being judged and the issue that even the parents if you say I want to go and get tested they will say so you are having sex. So, they are things that not yet acceptable because they believe that no sex before what…marriage.

KC: Before marriage.

31: Yet there are changes in the society because of generations so this is making a lot of girls to…this issue of being judged, aah.

KC: Not to speak out, to keep quite.

34: Then the other issue is of ignorance like you are told that the person you are dating is not good. Sometimes the elderly will be telling us, and we ignore that they are being jealousy. They don't know what, what, what, the issue of ignorance that's the other cause.

KC: Ok, alright 29.

29: The other issue that makes people keep quiet, not want to go to the hospital. If you get there, maybe something has happened to your private part. You go to the nurse, and she will say such a young child like you…it's like with our very own clinic if you go there, let's say you have issues related to these ones. I have got a friend who went there, she had a yeast infection, but it seemed like an STI.

KC: Uhm.

29: She was shouted at so at your age…I still remember we were first years and the nurse who was there said to her so at your age you want us to open your panties and examine you. Such a young person you are already engaging in adult things, being shouted at. So, you at the end of the day…she told me that aah the way I was treated at the clinic. If I get into a problem, I will never feel comfortable to go and get examined because you will say eeh they will judge me those women that this child has done this.

KC: Uhm.

29: So, I believe that nurses they should be friendly.

KC: Ok.

29: Even if I go having an STI they do not have a reason to judge me because I need help from them the nurse.

KC: Ok alright. Ok so still on the point that you raised, judgement from health care workers where is it centered on? On that adolescent girls are engaging in sex at young age, or they are engaging in risk sex of having sex without protection? Where is their judgement coming from?

29: The reason behind is that there are African parents, these nurses are also mothers in their homes. They still have an African thought of saying the girl child should engage in sexual activities after marriage. [People speaking at the same time] They don't believe in sex before marriage.

KC: Alright.

29: So, if you go there whether you have you used a condom or you have not used a condom just the issue of saying you have had sex only.

KC: Only having sex.

29: That's the one that arouses their feelings to shout at you because they feel that like in African societies, they believe that every child is your child. So, they will be saying my child could have been the one who have gone to the hospital and her genitals are looking like that, what will happen.

KC: Ok.

29: That is the issue.

KC: Alright, 35.

35: I wanted to add that I think there is a lot that needs to be done when it comes to these people who work in the health care sectors because let us say I'm here honestly many people no longer want to go to the clinic.

KC: Ok.

35: The campus clinic because you are being judged and the other issue, let's say I'm at my home area I stay in XXX then I want to go let's say I want to go to XXX hospital to get help. Then the person who is supposed to help me maybe she is a relative or she is a person that I know from church or in the area those people they are unable to hold my information confidentially.

KC: Uhm.

35: One way or the other, they are going to end up spreading, uhm so as young people we no longer feel comfortable in visiting those places.

KC: Ok.

35: So, people end up trying to find other ways maybe going to uhm those community midwives and staff to get ways of which some of the ways will not be what, will not be proper. They end up being a danger.

KC: Ok, 30 then we move.

30: The health workers tend to be different; I have my aunt who works at our hospital in XXX where I stay. So, my aunt, aah I have a friend who had an STI, and she told me, I don't know a notebook that she gave me. I do not know where she had gone to. She was embarrassed because she knew that… she knew that my aunt knows what, that she is my friend.

KC: Uhm.

30: But then knowing my aunt as I do, she is someone I talk to when we are home. Her argument when we are discussing sex issues, she will be saying that don't you know that there are condoms, don't you know that there is PrEP. Why do you get badly infected genitals when you know that there are condoms, when you know there is PrEP?

KC: Ok.

30: So, her argument it's not on why you have sex as such but why do you have sex … [Interruption].

KC: Not using the…things that are there to protect you, alright. So, someone mentioned that information like…for the adolescent girls to share health related information they can share with someone whom they are close to. Who are in similar situations with them so besides friends who are close to you, are there any other people whom the adolescent girls can share information with? The parents have been removed being said it will not work.

XXX: [Laughing] The parents we cannot.

KC: It will not work, are there any other who qualify in the category for sharing information? 30.

30: I think it depends on how they are, I have someone I know who got pregnant. Her mother shouted at her saying, but you know that's my line of work and I deal with such issues. You were supposed to be free to talk to me. Let's say maybe something might have happened that's why she said to her I was free to talk to you but at that time before it happened, I don't think she could approach her. Telling her that mum I am engaging in sexual activities. I want to use some protection; I don't think the mother would have tolerated her.

KC: Ok.

30: So, parents are out but someone you can talk to is your older sister.

KC: Alright, the siblings, ok.

30: Siblings you can talk to.

KC: You can talk to them, 35.

35: Yeah, I want to give an example, I once went to a workshop whereby uhm, let's say Zimbabwe was represented by people from let's say junior councilors and others. So, the argument is like you were raising ideas like we were saying parents we need to be free and we need to find a way like a user friendly uhm ways of, uhm, so that girls uhm, the people actually the young people can be free to go to that particular center and get help.

KC: Ok.

30: And the argument raised by one of the ladies was like, yes, I know it's my responsibility, but I won't take it as my child honestly, I will beat you blue and black. [Giggles]

KC: Ok.

30: So, that's most of the typical parents in Zimbabwe, they belong to that category. Only a few can understand because right now honestly there are, it's hard to, for me, let's say for me to go and tell my parents like I now have a boyfriend and staff.

KC: Uhm.

30: They don't want to hear such issues, they say school first, but I am now 22. If you had issues over a boy, just talking about it just general what more sex. [People laughing] You will be made to pack your bags, I don't know where you will go, so uhm…

KC: Alright, ok. Aah 27, then we move.

27: Alright I wanted to answer on the issue like who else can adolescents girls share with so I thought, uhm I was talking about this other workshop I attended. So, we were talking about HIV issues and staff so in that everyone was just sharing no judging or whatever. So, like people there most of them were not aware so suddenly people started feeling free to share because they didn't know the people and people were not judging them.

KC: Ok.

27: And also there were counselors I don't know maybe it's a skill, they learn how to talk with people and all that. So, I feel like as young girls we are more comfortable to share with people whom we don't know, people who don't judge us as well.

KC: Who don't judge, ok, great. Uhm, moving on there was a time when we were asking that the information on PrEP that you are talking about where you got it. You mentioned where you got it right. What if you have been asked to make a recommendation of the best places or the best sources that you can think of where PrEP information can be obtained from by the adolescent girls, which ones can you suggest? Where best can adolescent girls get information about PrEP, 28?

28: I think people maybe people of their age, maybe you can…for example you actually train people of their age. Maybe fellow classmates whom you are comfortable to go and talk to them compared to an older person who may not understand you, they can chase you away or they can harass you or something like that.

KC: Ok.

28: But someone who…maybe at a university there are people who are within our age range who can…they tell us the information the more we interact with them.

KC: Alright, from peers. Alright, 33?

33: I also think that eeh most of us here communicate on social media, nowadays there is creation of these platforms whereby you just enter in your question and then it will be replied. And then you can also get other questions and respond to them that sort of thing. So, like I think this is the best thing, because like I think we spend most of our time on social media anyways.

KC: Ok.

33: So, if those things are there it becomes easier to access that information, and we also feel more comfortable asking something from people you are not seeing. You don't know and knowing that you are getting your answers there and there.

KC: Ok, alright, are there any other? [Silence] Alright so moving on we want to look at the response that was given by Koko, I hope we still remember right. [Laughing] Koko's response when Chido gave her a suggestion that she should take PrEP. Initially she had some resistance right, she said that one I will have a big tummy because of taking PrEP. Uhm the other point that was raised was where will I take the PrEP from and if I take

it where will I keep it, these are the responses from Koko right. What do you think about the responses looking at a typical everyday life? 27.

27: I think these responses are mostly inspired by lack of education; people don't really know that what she was talking about to say I will have a big tummy. People don't know how it works; most people are afraid of the pill.

KC: Uhm.

27: Ahh I will get sick or what so I think more education should be shared so that people know more about it.

KC: Ok, uhh. Others, 33.

33: There is a point whereby she said that where we keep them think that's a real issue, because trust me you cannot … because the moment your parent is going to see them in that box in her home. You will be told so you are having sex, and it will be a big issue, like it's a really a big issue if you want to keep things like condoms and PrEP or anything. Where do you keep them and how are people going to react if they see them?

KC: Ok alright so on the fear that if people see them, where is the fear? Alright they have been seen in the house, the parent has seen that there is PrEP in the house, where is the fear, what is wrong with that, 29?

29: Our parents believe that we are 100% pure, we are not even sexually active. So, if you are seen with PrEP or condoms, you will pack your bags and be left at a guy that you don't love. [People laughing] As they say we cannot stay two grown up women in the same house.

KC: Ok.

29: You should be a child, a child in the home and I will be the mother. We cannot stay two mothers so PrEP you cannot stay with it. Maybe if there were the injection I would just go to the hospital and do what, get injected and have it stay in my body and go around having it in my body… [People laughing] aah because uhm…african mothers.

KC: Alright, 30? [Giggles]

30: The issue of where you will keep it, I want to give an example of what happened. In our home some condoms were seen, I am the older sister in our family so aah it was trouble. I think I went for 2hours just bathing, just bathing. [People laughing] My young sisters were…the person who saw them is my dad can you imagine.

KC: Ok.

30: So, it was a difficult one such that when I came out I was told I have called your mother, she is coming back from work just now. Now being seen with PrEP they would say you have started again, [people laughing], you have done it again.

KC: Ok, 32.

32: Aah I want to talk about what happened. So, like I can say we were in school then those things where you have a friend, and you will be walking together at school. She says let me put my things in your bag I will take them what, what. Then she forgot the plastic that had family planning tablets. We were closing the semester I took my bag and went home, then my mother was unpacking my bags. She saw them trust me I was in trouble as she was saying…some of the tablets had been taken so she asked me to go and throw them in the bin. Trying to explain that mum I…plus she will not hear you, she was bitter, trust me that is not possible.

KC: Ok, alright, ok 33 then we move.

33: Addressing that issue I would want to say that if you see most programs that are happening nowadays they are centering more on us the adolescents right and I think on that field there have been successful but it also comes to … they also have to go to those mothers hey and talk to them and say you know what don't think your children are not doing it, they are doing it. Sex is being done…maybe they will understand so that you will not find yourself in a situation that will… you have to explain … like someone who is not doing what, who does not know what is happening. I think it's like as for me and the family I come from and my uncles normalize always asking me do you have condoms, you get it?

KC: Ok.

33: I never told them that I do that…do you have condoms, hey do you need anything? So, I think we should educate more also the older people so that they come to understand this because on our own I think not everyone, but I think we are now coming to understand. We are now coming to accept that we are doing this ... so, I think we also need to educate the older people.

KC: Ok, alright besides the responses given by Koko, are there any other responses that can happen in real life if someone is approached, being offered…being offered PrEP by a

friend? That aah go and take PrEP, are there any other responses that have not been discussed from their role play? 33.

33: So, considering that these ones maybe they knew what they were doing together right, so there is a scenario that maybe you know that I do it and then I don't know that you know that I do it. So most probably if you say to me my dear go and take PrEP, she will tell you aah what have you seen on me, you get it?

KC: Uhm.

33: What do you think I do because I am trying to protect my image and reputation.

KC: Ok, is there another response? Alright, what about the benefits of PrEP for adolescents girls and young women, which ones do you see? 30.

30: I think the number 1 is the protection from HIV and all of us here we are afraid of it so that's the main thing.

KC: Protection against HIV.

30: Of protection.

KC: Alright, is there another benefit of PrEP besides protection?

28: Just buttressing what she said, you will be the one who will be saying I on my own. Its different from relying on someone, if they tell haa don't worry. I don't have it what, what but you will be knowing that I am the one who took it.

KC: I took it, so I am protected.

28: Yes.

KC: Ok, 30?

30: Yes, adding onto what has been said, uhm there is an issue of if we want to say we use the condoms. Eeh condom at most times the fermidom it's not often used. It's usually used the male condom so now I want to do it with this one and they say I don't want a condom so that's the end of it, I am now at a risk but if its PrEP I am the one who takes it.

KC: That I am protected. Ok, alright what about the…what we can call challenges or barriers that might prevent the taking of PrEP by adolescent girls and young women that we did not discuss when we were discussing Chido's role play? Is there anything else that might be a barrier besides the accessing venue, fear of being seen by the parents? Is

28: I think right PrEP we should take it in a consistent way, right?

KC: Uhh.

28: And as adolescents I think that might be a challenge because imagine our life is very busy and for you to remain being consistent that PrEP, I must take it at such times. You never know how that day is, maybe you have three exams in one day. Also, sometimes you get there and sleep, maybe you are supposed to take PrEP at 8 and you will not wake up so consistence might be challenging because of our schedules and staff.

KC: Ok, alright, 33.

33: There is this issue that I don't get where you say that for you to have PrEP you need prescription right. Which means I cannot just go "_+". I think that's…it should be something that we can buy.

KC: Ok, alright. 29.

29: PrEP is it given for free or its bought?

KC: Currently most set ups that are available…because at the pharmacies PrEP is not yet available. Currently its available at the clinics, public clinics and the ones that I know of they are giving it for free. Yes, that's the current set up but there are other set ups I 'am not yet sure if there is somewhere where its available and being sold and being sold for how much. Where I know it available for free, 28.

28: I think that also must be a barrier because especially clinic that will be local you can't enter clinic and get the service even for free without paying what is it called, that fee. What is it called that fee you pay when you get to...

KC: Consultation?

28: Consultation even if you know I want to go and get PrEP that is offered for free. They will tell you first pay that $5 then come and collect, even if you want to pay for something that is less than that, they will tell you first pay $5.

KC: Hoo for the consultation. Ok 27.

27: Right, adding on to what 28 said I think accessibility also becomes an issue because if its available at clinics and am at a place where there are no clinics, there are pharmacies and yet it's not available in the pharmacies. It's just at the clinics; it becomes a problem now so I think it should be easily accessible anywhere as long as there is a pharmacy or local outlet.

KC: Ok, alright. That's fine, that's great. Uhm, moving on right we want to ask, you have talked about the pharmacy that it should be easily accessible. Pharmacy, what are the advantages of getting PrEP at a pharmacy? Advantages and disadvantages of getting PrEP at a pharmacy? 30.

30: There are a lot of pharmacies than clinics so if I am going and buy my morning after that is when I will get the PrEP as well.

KC: And PrEP as well. Ok, uhh, 27.

27: There is an issue of time, it saves time because at the clinic usually there are long queues and you have to adhere to all the processes whilst at the pharmacy you just get there and buy and then you go.

KC: Ok, saves times, 29.

29: Another issue of the pharmacy there is need for money and most medication in the pharmacies is expensive, now at the hospital it will be what…

KC: Will be free.

KC: You see now.

KC: Alright what if we say in the situation where a program has been designed that pharmacies can give PrEP for free to adolescent girls and young women, will that work?

All: Uhh it will work [speaking at the same time].

29: We will all go.

XXX: I don't know that…those who move around in the community educating people. So, they should have some days or every month where they move around in the community giving people.

KC: Alright they will be moving around in the communities?

XXX: Exactly.

KC: Alright so they move around with the PrEP or just educating people about it?

XXX: They will be educating people at the same time giving them.

KC: Ok, alright, 27 then 33.

27: I still feel that even provided that its being given for free or its coming to the community with the educators, it's still comes down to whether an individual would feel comfortable to walk in and buy PrEP right because there are still many ideologies. Even if it's said its being bought in a shop someone might find it hard, knowing they need it

right now. They might find it hard just taking it and going to pay at the till. So, I think the human mindset has to be changed, the ideologies around those things.

KC: Ok, it needs to be changed a bit. Alright, are there any other places that we did not talk about where PrEP services can be accessed from by adolescent girls? 33 then 30.

33: Where it can be accessed as in where we think we can go…

KC: Ehh where you think you can go and get PrEP.

33: Like, you do get that schools do have got those dispensing for condoms.

KC: Yes.

33: I think they should also include for PrEP too.

KC: Ok so they have what…sorry I did not get that?

33: Dispensers for condoms and other staff.

KC: Ooh that it will be placed there.

[Noise and people speaking at the same time].

KC: Ok alright, 30.

30: I wanted to say something like that, that there are some places where condoms are placed in the toilets.

KC: Uhm.

30: Yes, you just put PrEP since it will be sealed, and staff and you know that you are safe. You are the one who have done what, you have started taking it but if it's an injection or tablets there might be a problem that I am not sure if they can be abused. We might have a problem of people who will be abusing, we are now creating another problem of substance abuse.

KC: Ok.

30: So maybe the vaginal ring if its…if it's well educated to us we can accept it and I think that's the safest one.

KC: Ok, what about storage how will it work in such a model of placing them where condoms are placed, our storage for example the oral PrEP because it has some conditions that it must be stored under?

30: Oh, alright storage might be a challenge.

[People speaking at the same time].

KC: Ok 32.

32: I think that they will end up selling to other people.

XXX: That's true.

32: Abuse the privilege that you would have been given so it won't end well.

KC: Ok, alright. Its ok. Uhm are there any other benefits, ok alright. So, if there is nothing lets now move to mai Bhobhi and mai Juru's role play number 2. Where they are young married adolescent girls and young women one is taking PrEP but now considering stopping taking PrEP but her husband has some girlfriends now we want to hear from mai Bhobhi and mai Juru then we move.

Role Play 2

Mai Juru: How are you mai Bhobhi?

Mai Bhobhi: Aah mai Juru how are you?

Mai Juru: Aah I have come with my issue that's troubling me.

Mai Bhobhi: Talk my dear.

Mai Juru: I don't know how you are going to handle this, when I was coming from the shops, I passed through Jongwe, and I saw baba Bhobhi with a young girl.

Mai Bhobhi: [Chuckles] Do you think that man will ever grow up, he is getting old still running around with young girls. No, I am tired my dear, an old man who cannot keep his zipper zipped up.

Mai Juru: What if he brings AIDS to you.

Mai Bhobhi: Iii my dear I am taking PrEP, one of these days when I was listening to the radio, I heard this program that started. There is something called PrEP my dear, I now have six months. I was taking it so that I am not infecting with AIDS by that man when he has returned from his girlfriends, they are not using condoms. I thought of protecting myself.

Mai Juru: That's the best, now does it not have any side effects?

Mai Bhobhi: Aah I now want to stop using it because currently I feel…I feel dizzy. Ahh but again if I think of not using it that man…but aah anyway I will stop using it. If he does not want to use a condom, I will just tell him no condom no sex because he is just getting it anyway. We just stay with each other for the sake of the children.

Mai Juru: What if you leave him "_", does he matter…You can just get divorced.

Mai Bhobhi: Umm my dear children need to grow up with both parents.

Mai Juru: Alright.

KC: Thank you. [People clapping hands] Alright thank you mai Bhobhi and mai Juru for the role play. Right, we want to discuss the role play for mai Juru and mai Bhobhi right. What do you think of the role play? In real life are there married adolescent girls and young women who are in situations like mai Bhobhi where they have a husband but the husband has many girlfriends, are they there? 31.

31: Yea most men have girlfriends, uhm tracing in our society from long back. A man for him to be called a "man" he should have what, many women so they believe that if you do not have a girlfriend, you are not a man.

KC: Uhm, uhm.

31: So, a lot of men have what…

KC: Girlfriends.

31: They will be referred to as dull, you do not have a girlfriend, you are dull, so it makes most men to do what…to have girlfriends.

KC: Alright, uhu, 35.

35: Yea, I agree that there are people who come across such an issue and the men most of them are the ones coming in as blessers.

KC: Hoo okay alright, 33.

33: I would say these issues especially considering the fact that these are young women around their 20s, most probably the man would be around 20s, they will be very young. Those ideologies that I came across that if you marry at a young age, it was said especially men he will not have been satisfied out there so its most likely that he would do what…he would cheat sometimes. Yeah, its centered around the issue of getting married at a young age, the husband… [Interruption]

KC: Would not be mature enough. Ok, 34.

34: There is an issue that people say the married women should concentrate with her home. I have a woman to go out with having fun, going to some holiday with her.

KC: Ok.

34: They believe that if a woman gives birth, she looks old. Her body will be worn out and then they need someone who is fresh, who does not embarrass amongst people.

KC: Ok, someone who has energy [people laughing]. Alright, do you think it is easy for a young woman like mai Bhobhi to decide that I am now taking PrEP? Is it an easy decision for young married woman like mai Bhobhi that I am starting to take PrEP? 29.

29: Aah it's not an easy decision because taking it its everyday, it's not easy. It will end up being called taking daily, you know that I must subscribe just because of someone, another woman's child who cannot control themselves. [Laughing] [Sound of something falling] It's just hard because it's not nice to be taking pills, just because you must protect yourself from someone who can live a normal life, just being a decent man without any associations.

KC: Ok, alright so it's not easy on…

29: Aah it's not… [Interruption]

KC: Idea of taking the pills.

29: It's not easy but at times circumstances forces you.

KC: That's what forces a person, ok is there anyone else. Is it an easy decision? [Silence] Ok what if we look at the barriers that might prevent married women, on this one we want to discuss about married women right. Is it the same as the other adolescent girls we were discussing that is it the same for them to take PrEP, married women, specifically for married women? Which barriers might be there for them that might prevent them from taking PrEP, 30?

30: Another thing that might prevent one from taking PrEP is that the husband if he sees them his reaction might be the same as the men who does not want condoms. He will be saying that you are making things up, it's not for yourself. You are lying to me that they work to protect maybe you want to harm me.

KC: Ok, alright that they might suspect that you want to harm him not that you want to protect yourself.

30: Or they will be thinking that way because they are ignorant, he does not know how they work. Sometimes he will be thinking that you are infected so even he gets to know that he is infected he will be saying you are the one who has infected me with your pills.

KC: Ok, alright, 29?

29: Some men they might turn it against you that so you are being promiscuous, you are taking the pills. He is now putting the blame on you.

KC: So, they cannot acknowledge that I am…I am behaving this way. This person wants to protect me, he wants to protect herself against me.

29: He will say why are you taking it, you know your issues.

KC: Ok, alright blame shifting, it's now coming to the wife. What about what can encourage these married women, adolescent girls, what can encourage them to take PrEP? 28.

28: I think you will be in a situation that…there was a girl who was discussing her issue that she got married right and the person did not leave behind his female friends. He would say I am saying who enjoys the company of females. Then he left his phone unlocked and she started seeing some nudes and staff, they had a meeting, and the husband said he had changed. Like I won't do it again but then she noticed after some time she had an STI when she was only sleeping with her husband. Then she confronted him, and she realized that he is continuing so I think it is better that you protect yourself because you can't trust what someone tells you saying I no longer do this.

KC: He has changed. Ok, alright. 33.

33: Then I am going to comment on that especially for young married women right. One can say whilst they have a boyfriend, I need consent, I need…

KC: Ok, alright.

33: And that issue for you to start it in the home that my husband I have thought of doing this, right. Its challenging because number one it means you are completely declaring that in this home there is no trust so it's not an easy decision to take because at the end of the day you see that it can cost your marriage. You will be saying I am staying for my marriage; I don't want to disappoint what…what…yea.

KC: Uhm, uhm. Alright so consent for you to start taking PrEP she might need it from the husband?

33: Yes.

KC: Ok, 30.

30: You can think what if my sister-in laws or my aunts hears about it, they will be saying we hear that you are discriminating against your husband, eeh his sisters saying aah you are being cruel to him.

KC: Alright.

30: Because he does not have money, you are now being told other issues that are not… [People laughing] when you were only afraid that this man might make me sick.

KC: Alright, ok 34.

34: Another issue is that people want to do something after they have seen many people doing that, writing a test with people. Like on getting the covid vaccine, they would wait if so and so has been vaccinated. If they hear they have been vaccinated and there are no side effects and then they can go so women wait to see who has used it, does it work.

KC: Alright, to see who have used PrEP, does it work before I use it?

34: Then you give a testimony that so, so, so. It happened to me, my husband is…what, what that is what they want in those marriages.

KC: Ok, alright. What about the issue of mai Bhobhi who is now considering that she no longer wants to use PrEP but she has used it for over 6 months. What do you think why…alright firstly does this happen, is it something that happen that one might want to stop using PrEP, but they have been using it for a while?

ALL: Uhu.

KC: So, anyone who would have said their point, they give the reasons that I think because of this and that. 28.

28: It's the same, mai Bhobhi might be working at a job that requires that 100% she is there of which it was mentioned that some side effects are dizziness, headache. Maybe mai Bhobhi is a surgeon and at work this has been happening for the past six months, you can't keep on making excuse that aha she is on PrEP, she is on PrEP. Maybe she is an engineer she must go underground, not sure how deep it is and there is no oxygen. She is already feeling dizziness; she cannot operate at the full capacity at her job.

KC: Ok so the issue of side effects might be a barrier, alright. 30.

30: Yeah, there is an issue that mai Bhobhi might develop some bitterness right. She might end up giving up and say whatever happens. Its just the same with our folks who take the HIV/AIDS treatment pills. Some of them you hear they have defaulted; they have given up. They are tired, just saying I don't know whatever happens.

KC: Ok, alright, 32.

32: Aah I wanted to say maybe like taking the pills, like what everyone has said that taking pills is boring. So, they would be feeling like they now seem like the people who are infected who take them daily so it's just the same with someone who takes them (ART). I don't know because if she withdraws then she is at risk so it's a challenge.

KC: Ok, alright 33.

33: I wanted to comment on the issue that maybe mai Bhobhi, maybe baba Bhobhi has managed to convince her that I have changed. I know this myself it is like a stereotype but the mindset of a woman mostly if you are in a marriage and you want things to work. If he says my dear what you used to hear about me, I have left that behind. If you want to see let's do it together, let's be one and you will not be aware of the person's motives but like I said us women sometimes when it comes to things of love we have a way of reacting so you can think that aah I am now doing what…aah let me stop maybe baba Bhobhi is now clean.

KC: He has changed, uhm.

33: Yes, of which you will not be aware that maybe the person got infected long back, maybe it was already bad.

KC: Ok alright, ok. What can be done to help the young adolescent girls and young women who will be on PrEP to keep on using it? Any suggestion on how can you motivate continuous use, 28?

28: I think we should go for counselling, it's the same as what was said about HIV that even an HIV positive person hesitates but then they are making some counsel groups. Such that here they come and counsel them, reasserting their goals that, that is why you are doing this and this. That they will be motivating them, at the end seeing other people who are like them who used to be in such a situation. Interacting and then they get motivated to do that.

KC: Ok yeah, 30.

30: I think PrEP here in Zimbabwe since it has some challenges, may you quickly make it possible that we get it in its different forms. Each one chooses that aah I as number 30 I want the injection.

KC: Ok.

30: That's the one that does not bore me because I will not get tired to do what…to take it or maybe this time I want the injection, maybe next time insert the vaginal ring in me.

KC: Ok, alright.

30: Yes, just like that because maybe the pills, honestly the pills are difficult. I might forget them in Gweru when I am travelling to Victoria Falls, for me to do this…things are now bad.

KC: It will not work.

30: Yea.

KC: Ok, alright 33 then we move.

33: What I will say is in line with what she said, I also want to say that even here not so far in future it will be ok to have a modified form whereby you can take it maybe at the beginning of the month. You are told you have a prescription of this, they can prescribe up to 30days then you take that way. You will not be always under the process that I am… [someone coughs] I am looking for PrEP, I am doing so, so, it will not be there.

KC: Alright so if I have understood you, so you are saying that you get a prescription that lasts for more days.

30: Uhh.

KC: If you get there, you go for 30 days then you go back again and you take.

33: Uhu.

KC: Alright, its fine. Now are there others with anything to add before we move to role play number 3 which will be our last role play? Eeh 32.

32: Sorry, I want to…there is something that was mentioned by that one that prescription needs be written down for you to go and get the pills. What about in this…how many pills will be there?

KC: There are about 30 I think, there should be about 30 tablets that are there because right now just like the way it is being given currently with the Zimbabwean set up. It's that you are given 1 month supply at first visit then on the second visit you get a supply to last even 3 to 4months, going on like.

33: Ok let me say it this way what I wanted to say is modification of the tablet that prescribes over a long period.

KC: That if you take it, it lasts in your system for 30 days?

33: Exactly.

KC: Ok, you don't need to be taking it on regular basis?

33: Yes, that's it.

KC: Ok alright so that has a long-life span for being in someone's system. Alright its fine, now we want to move to the third role play of the three friends who are designing their own implementation program. Such that if its designed and given to the adolescent girls and young women they will be free and want to take PrEP so they are assisting us by telling us so and so. Explaining it so group number three go ahead and do your role play and then we move.

Role play 3

Sky: How are you friend?

Peppa: How are you Sky?

Sky: I am fine, how are you Peppa?

Peppa: I am fine, did you hear about the programme that was talked about that us people who are in their 20s we design best ways, did you hear about it?

Sky: Yeah, I think it's a very interesting program. We must plan carefully and see that if we want our thing to succeed what can we do, which people can we invite.

Princess: What do you think?

Sky: I was thinking firstly since we are still young and are still energetic. We would want to invite peers to our training. If we invite even some artists at our event, don't you think that will be entertaining. Older people can even come to the program, the program will be successful.

Peppa: But if we invite many people, isn't it here at the XXX it's a small place, will people be comfortable enough? Imagine what can we do so that they think that the things that they are coming for…at least it will not seem like they are bad things. You know because if we discuss about PrEP, they might think that we are calling them for sexual issues, then it's portrayed in the wrong way.

Princess: Yes, that's true.

Peppa: So, we must know the crafting of our message for it to get to the people, what should we do?

Sky: Aah on that one we should plan; you see that it has some confidentiality issues involved. That if people want to discuss what…there might be some counsellors. A person gets PrEP and counsellors in privacy.

Peppa: Yes.

Princess: So, guys what do you think about the people who will be involved, age, age. Which ages do you prefer for the dispensers, giving awareness?

Sky: I think we can base by saying the law say one can have sex…so at least maybe if we say from consent, it's what…16 by the way, right. If we say from 16-24 those are the people, we are doing what…whom we are inviting in the program. Then not knowing what you think of inviting young men so that they are also present, or we do it a girls thing only?

Princess: Aah Peppa what do you think?

Peppa: Aah I think it's just inviting even both so that each person hears for themselves.

Sky: But won't that make other people to be shy?

Peppa: I don't know about that, we must really think carefully about that.

Princess: Then comes the issue that so the things that we would be distributing on our programme, what do we give them?

Sky: Like the fliers that they will go reading the fliers.
[Speaking at the same time inaudible].

Princess: Or that they will give to their peers who would not have come.

Peppa: That's true because it's not everyone who is going to make it, to come right.

Sky: Plus, since it's a program for PrEP right, then we include bringing in some condoms and so on.

Princess: The young people they like condoms, they will be saying we have found free access.

Sky: Yes.

Peppa: So I think our program if it's like that, then we invite influential people who make many people come, it will work.

Princess: Yea that's true.

[People clapping hands]

KC: Thank you group 3 Peppa, Sky and Princess right. Right so we now want to discuss role play number three. Right, firstly do you think adolescent girls and young women if given opportunities can they develop their own PrEP programs? Such that if they are

told design a program that they can suggest to the Ministry of Health can they design their own PrEP program? 30.

30: Haa we can do that.

KC: You can do that?

30: Because…because we are the people who are…who are at risk of getting what, HIV because of number one the issue of the blessers right. Then number two issue that we fear judgement from older people so if they come and be amongst us people who think in the same manner it can help us.

KC: Horaiti, 33.

KC: Alright, 33.

33: In politics we say nothing for the youths without the youths. So, I think if you say we design for ourselves our own thing, we are the ones who have the best capacity to come up with something.

KC: Ok, uhm 27 then we move.

27: Plus, aah as young adults we are the ones who know the environment that is most conducive and most comfortable for us so we are just able to recreate that environment for things to go on well.

KC: Ok, alright. Looking at the program that was designed by…by group 3 right what do you think about the program? Do you think it works well in reaching adolescent girls and young women for them to take PrEP or be interested in PrEP issues? 30.

30: Alright, there is something that I liked that was mentioned by group 3. When they talked about including boys as well or not.

KC: Uhm.

30: There is a type of feminism called…called ego feminism and that ego feminism it talks about the issue that you should negotiate right and form partnerships between issues that affect women. Involving what, men because these women when they live, they don't live as women only, they live with men so these men. We might have a problem if we educate women only about those issues, excluding the men because I believe that the majority of Zimbabwe right, we are in heterosexual what, relationships. We ascribe to heteronomy activity so obvious men are involved in such cases so if we leave out and say let's only tell the women. There will be problems like these ones of mai Bhobhi

that they will be saying aah if I take it and baBhobhi sees it, it will now seem as if I am discriminating against him so they should be present and be told why it is good to take PrEP, so that they know.

KC: Ok, alright. What about the program that has been designed, is there something that you think will not work well on the designed program? Is there anything that will not work well in reaching adolescents girls and young women? 30.

30: I did not quite get when they were talking about XXX that did they say it will not work to have it here. They want to do it somewhere that's private or what. I did not quite get that; can you tell me.

KC: I think it was about…they were saying that doing it at XXX maybe some might not be comfortable being seen coming to such a program that has been done at the campus. So maybe finding a venue where people will be comfortable going there, is that correct?

33: Yes.

30: Alright if it has been done like that it now means that there are some maybe who cannot go to that venue. They are now being left out so such a program should be done at the campus just like how the issues of morning after are discussed. Condom issues, and the issue of condomless sex, that is how PrEP issues should be discussed.

KC: On PrEP issues.

30: Let it not be something embarrassing that you have done so and so on that one I need it to be rectified.

KC: Alright, 34.

34: Then I think that the program is…is active in urban areas and not in rural areas. Of which people in the rural areas, the cattle herd boys and others should be targeting them.

KC: It should reach the rural set up as well, uhh very true. Alright, there are some things that came out of the program, there is an issue of confidentiality that it should not be missing. Are there others that…is there anything else that you think is important or if a PrEP program is for adolescent girls these things, for it to be successful these things should not be missing? Confidentiality that has been discussed before, is there anything that you think should not be missing at all, 28?

28: I think like being innovative, not to have it like a seminar. That they will be sitted and being told about PrEP, maybe having a quiz or debates or games that are informing at the same time, being…being interactive.

KC: Ok alright being innovative, 30.

30: Yea still at innovation, I think we all know that the circumcision program made use of Winky D. He said what…get circumcised and staff, such things so if they do like awareness campaigns or roadshows. PrEP will end up being known even with a primary school child.

KC: Uhu.

30: Because an old dog is not taught new tricks, it's now fortunate that we are now a bit flexible because of education but if today you want to teach me about PrEP now all grown up. Maybe I am too involved in these sexual issues, they will tell you that aah this is now something else that you are talking about.

KC: [Laughing]

30: So, if I start knowing about it maybe at the age of 10 years, before I am sexually active it helps so especially the road shows they are helpful.

KC: Alright, 32.

32: I wanted to second what has been discussed that involvement of the celebrities makes people to…to…accept information faster.

KC: Ok.

32: Like the one she was talking about of getting circumcised. Getting circumcised is known even with a primary school child so if its PrEP I think if there is maybe Amara [local Zimbabwean female singer] she is popular or someone…the young people, the young people quickly accept such information.

KC: Ok.

32: Such that it will not be an embarrassing thing to be talking about PrEP or people will stereotype me or something, but it will be…

KC: Alright, 33.

33: I am in support of this, but I would want to raise a point that with things like PrEP that involve…because you are taking it because you are having sex.

KC: Uhh.

33: Right, they then come ideology that they are teaching the children to be mischievous. I think most of aah campaigns that have been done to most things related to…to…to sexual issues right. There is also like fear in our communities that even to normalize that someone should take PrEP, it now means you are telling them start engaging in sex, everything is fine.

KC: Uhm. Ok 29.

29: But I think that PrEP issues and issues to do with circumcision and condoms are all the same things because why is circumcision being done?

32: It's a way of saying do it.

29: Yes, it's the same with saying from children that are in primary they are getting circumcised, so I don't see any problem of introducing PrEP to the world, to the children even from elementary school until people are in tertiary education. A child grows up knowing that because this new generation…yes in Africa our parents are older people, they are not ready to accept that children engage into sexual activities at a younger age but it's happening. I think it's better to prevent than for a child to come back being sick. The issue of condoms, children are getting impregnated, look at how many child marriages are happening early, children are getting impregnated whilst they are still young, how many issues are they?

KC: Uhm.

29: So, I think that prevention is better than cure because today you the parent cannot look after you when you are only one but they will have the burden of looking after you and your child.

KC: Uhm.

29: So, I just think it's the best way to educate them young, they say catch them young.

KC: Ok.

29: If we catch them young, they will grow up with…having a backbone to everything.

KC: Ok, 30.

30: The issue that I wanted to talk about it's the same with what number 29 that PrEP is the same with that ABC, for abstinence, be faithful, condomize. It's the same because it's a way of preventing HIV so it's not bad at all. When the discussion around condoms started, being said they want to be placed in schools what, what it was a problem right

but us the ones at the schools we were saying it's good for us and everyone has a choice. You use it if you want.

KC: If you want.

30: If you cannot, you wait a bit and then use when you see that its necessary.

KC: Alright, ok. Are there things that can make a PrEP program to be bad, to say this is a bad PrEP program it will never be accepted by the adolescents girls and young women? Are there things that if you see them, you say aah it will not be acceptable to the adolescent girls and young women? Are there things that if they are there you know this will not be acceptable, yes 30?

30: I personally do not think that PrEP is bad, there is nothing bad about it. The only problem I might have with it are the pills. That is why I was saying pills might be a challenge maybe if they say an injection.

KC: Ok.

30: But I think that people who regard PrEP as bad might be ignorant, they will not be aware of how it works.

KC: Alright.

30: So, it might be a challenge for them to accept it.

KC: Ok, alright so I was not asking about PrEP on its own but the program, a program that would have been designed for PrEP. There are things that if there are in a program, you say these are bad. They will make the program to be unacceptable, like the program that we have designed are there things that you can say these are unacceptable? 28.

28: If it only has older people who will be facilitating the program. Talking about your experiences but you can see that maybe all the people who are at the roadshow are older people; you can't even relate.

KC: Ok, alright so older people so the ages that would be preferred for a PrEP program to…to be preferred by the adolescent girls which ones are they, the age group? The ages that would be preferred maybe for the provider, the facilitator, of the people who are involved in the PrEP, which are the preferred ages groups? 32.

32: I think around 18 to 25 because they can provide youth friendly, they can understand better, we can relate.

KC: Ok, alright so 18 to 25 the young ones, alright.

32: Uhu.

KC: Alright, are there others with a different view? 35.

35: Yea, I think to add on 32 it's just that those young people need to be on the frontline then behind them comes the experienced.

KC: Ok.

35: But the most active one will be the young ones.

KC: The young people, ok uhm, 33?

33: I just want to support that in the background there is need for the elders because of diversity. Do you know that it's not everyone who feels free with their peers, for one it's very easy for her to relate to an older person, that's the one they can confide in.

KC: Uhm.

33: So, it's not always that young person feel free because it's a young person who is here I think there is need for diversity… [Interruption]

KC: Ok so if there is a combination but at the forefront there should be the young ones then there are at least the ones who are a bit old and mature, the elderly ones.

ALL: Yeah.

KC: Being there to give support. Alright, eeh what about looking at…for example let's say PrEP is being given right. A facility where PrEP is given, PrEP is the only thing available and a facility where PrEP is available, the family planning is available as well. Then STI testing services are available which one would be most preferable between these two facilities and why? Yes 33.

33: I think the one whereby you can get all the other services at one place because I think for a reason such as time…reasons because it's just convenient to access all the things at one place.

KC: At one place, ok alright. So, moving forward now we have finished our discussion on the role plays, we now want to ask just some general questions. In terms…we had a small discussion about male involvement in PrEP issues so that they will also be knowledgeable about this. What about the role of male partners in uptake of PrEP by adolescent girls and young women? The partners they will be having, do they play a role, the male partners in PrEP uptake?

30: Aah the male partners, these male partners the issue that happens is the one that has been discussed earlier that they might sweet talk you right. He says aah I will not do it, I will not do it, so he has influenced the uptake of what…of PrEP in that he has misled you.

KC: Okay.

30: Then you think that it's now alright.

KC: It has changed.

30: Yet we are together in making you stop taking PrEP.

KC: Ok, 29?

29: Uhm, some guys they affect girls from taking PrEP because he will start saying you don't trust me. Relationship is built on trust so if you are taking PrEP it means you don't trust me so what are we doing, we are not going anywhere. So other girls because of the love they have for the person, you know relationships, you will be head over heels.

KC: Uhm.

29: So, you are saying if my boyfriend sees me with PrEP what will he think, I am affecting my relationship. I am affecting my person so some other girls they might not take it because they will be saying it's like I am questioning my boyfriend's trust.

KC: Ok, alright 27.

27: Ok there is also an issue that has been raised by 34 that eeh usually some judge with the eyes so usually one might just look at their partner and say aah but this one can he do it. Then she does not take it and say aah this one might be alright because you would have judged by your eyes.

KC: Ok, alright, 32.

32: Uhm I wanted to take maybe let's say the relationship the guy is HIV positive.

KC: Uhh.

32: For example, so he wants to protect what, his girlfriend. There are people who are honest maybe who are not cruel. They can say that aah what's the matter my dear, I am HIV positive you can go and get tested and get PrEP. Maybe she will be his wife at the end of the day, and they still want to have some babies.

KC: Ok.

32: At the end of the day everyone wants a healthy baby so yeah, they can take part so that they…they…they have the knowledge.

KC: For them to have, alright 33.

33: The scenario I wanted to give is similar to hers but now let's say you are boyfriend and girlfriend and then your guy comes and says my dear I want to go and take PrEP. Aah, it has some effect that you might have to leave him and find someone else, you will not go and take PrEP

KC: Not having "_+".

33: It means they are telling you that you are…so for married people its ok that way of encouraging you but if you are not after he has told you, you are now leaving him and go for someone who is better.

KC: Ok, alright uhm, 35 then we move.

35: Alright on the issue of PrEP I just want to add that… [Background noise] the involvement of men is also important, why am I saying so? Because these people they support like it can, we can give an example of Barack Obama he is one, he is one of the most known men to be a feminist. He supports women rights, so we see that now this issue is no longer between me and my boyfriend and I but also that also that boyfriend has got a sisters and staff, and their health is what… is important to them.

KC: Ok.

35: So, it can start with some resistance but as time goes on people will need to accept it so men need to accept it and also teach be it their sisters and staff, so I think.

KC: Ok alright 34 then we move.

34: I wanted to add as well on what has been mentioned, nowadays there are ben 10s. It's not because you cannot find someone who is alright so guys see it being easy to find a sugar mummy who will be providing money and then I will do something then after that he will stand on his own but at first, he would have found "_+".

KC: Ok alright, so currently, let's say the current set up in Zimbabwe right, here we are looking at the family planning issues. Do you feel that adolescent girls and young women can easily access family planning services currently can easily get the family planning? Currently, uhm 28?

28: Aah no, there is a time when we went to…the someone wanted those services, on sexual reproductive health and they were shy. The way they treated that person it was in such a way that she wanted, they were asking why she was shy, speak loudly. You are the one who knows what you want, speak, such things that you will not free to go back.

KC: To go and collect.

28: And you will be talking to someone, and that person is 50years and it will not work.

KC: Ok. Alright 33 then we move, we should be finishing off now.

33: I think theoretically it is said its now easy to access but on the ground it's not that easy.

KC: It's not that easy.

33: But theoretically if you hear it being discussed, it is said haa we have created an environment that "_+".

KC: Ok. Alright, now our last question is that we mentioned earlier about a study that we would want to do later, in the next few months to come. So, it will be focused on adolescent girls and young women who are sexually active right so that we also get ways of developing ways of providing PrEP services that are preferred by adolescent girls and young women. So firstly, where do you think we can get adolescent girls and young women who are sexually active coming to the study and not feeling that they will be judged, they will be discriminated, they will be…they will be labelled, 28?

28: Social media.

KC: Aah they are supposed to be coming on the ground, then we have the set. We pitch a tent, it will be physical, seeing each other physically.

XXX: Tertiary, tertiary, tertiary.

KC: Tertiary institutions?

XXX: Uhu.

KC: Alright so is there a specific point in tertiary institutions where we can do the set up?

XXX: Yes, even here is okay.

KC: Ok so tertiary, is there anywhere else where we can go? 33.

33: In the high-density suburbs.

KC: High-density suburbs like which ones, places like…is it just getting in…in the high-density areas and we pitch out tent and say girls who are sexually active come here, come here. [People laughing] Which area, 29?

29: I think that you should go to XXX, there is a place called XXX.

KC: Uhm.

29: There are kids who are into sexual work who are aged 14, 15, 16 such that our ages they will be saying we are old, they are out of the market.

KC: Ok.

29: I think you should engage that place then you teach those children because a lot of these babies are exposed to HIV/AIDS.

KC: So, they can come and enroll into the study without feeling…

29: As long as…they will come because if you say at XXX's even if you go starting at 4, you will see hell on earth.

KC: Ok, alright. Uhm, 34 then we move.

34: And also, to go to Hopley clinic, yeah those are some who start at 8 years.

KC: Going upwards?

34: Uuh.

KC: Alright, so Hopley clinic is…can be another one. Alright, are there things that we can…we can…Uhm do you think that if we go to those places is there anything that we can do to make them feel comfortable enough to enroll into the study? Is there anything that we can do here as a study, is there anything that we can do?

29: I think that you should offer some incentives because they will say aha to go there for nothing, rather than me waiting at XXX's and get a client.

KC: Hoo incentive.

29: So, you must get something back.

KC: What kind of incentive?

29: Uhm maybe just to give them food.

XXX: Food, money.

KC: For money which range can we give?

29: Uhm they work for even $2, these children. [People speaking at the same time]

KC: Alright so $5 will it be, okay?

XXX: Even a coin because even if you see them doing this, [making a sign], they will be referring to a coin.

KC: Ok, alright, alright. So, in that study there is an element that they will be asked to self-collect some vaginal samples which will be tested for STI. So, the places that we have mentioned, will they feel comfortable enough to self-collect the vaginal samples? Will they be comfortable, they will be taught that you go in this tent, you self-collect, will they be comfortable doing that?

29: I think it will be comfortable as long [interruption]

32: Aah I think that if people do it like that you can face some resistance, so they need a proper approach maybe.

KC: Uhm.

32: There are people like this as we have mentioned that they can feel free.

KC: Ok, alright, 30.

30: Such that people like me I tell her go and self-collect a vaginal sample there might be a problem again because she can say a young person like you. If it's an older person, she may think that this woman might do what…might judge me but in places like the tower light, at that place people are not shy.

KC: Ok, they will be ok with it.

30: It is known that this is our trade so they will not be embarrassed.

KC: Alright, 29.

29: The Anthony issue as long as there is money, they will never say no.

KC: Hoo okay.

29: That is why we talked about the issue of incentives because they are money hungry. That is why they go and stand in the streets so if you come, that is what they like people who approach them and give them money, they want food and staff. If you approach them as long as you show them money or food, they will agree. If I am asked to sign a consent form, and do so and so, it's a one word.

KC: They will agree. Alright so these samples will be taken and get tested for STI so people will be asked if they would want their results or not. Those who would have said they want to see the results; how can we give them their results? How can we give them their results? The results are out, how do we give them the results? 29.

29: I think that maybe you should take their contact details because there are no proper house numbers where you can go and approach that at this house number stays this one. Maybe if you call them and tell them that we meet at the same place on this day.

KC: Alright.

29: Those who will be interested, they come and collect the results then those who will not be interested.

KC: Alright, 30.

30: Aah another thing that needs to be done when you issue them out. I think they must come in a sealed envelope not just to drop them off, then you say we know that number 35…

KC: So sealed envelope, what about the STIs, those who have the STIs where do they get treatment, the adolescent girls and young women? Where best can they be treated for these STIs? 35.

35: Alright, I think also it's your role now to get involved or to get engaged with the local clinics and hospitals and make a relationship and maybe try to find ways that suits best so that these children will be helped in a nice way without facing uhm, any problems.

KC: Ok, 28.

28: I think people are speaking…supporting the clinics. There was this other time when this other drug was introduced which they were…it was about them counselling the nurses. Going around talking to them that when you see a homosexual person you must treat them with dignity so, so. They were not as resistant as before when they would say aah aagh…They showed that they are accepting, then we said alright…

KC: Ok.

28: Actually, a glimpse of change that they can do…

KC: To… "_+" alright, okay so there is …yes 35.

35: I think I also have a suggestion on the issue of approaching maybe people from clinics and hospitals because we cannot keep on running away from them. I think we can also do what happens professionally let's say security guard if I approach a person and a person gives me reception that I don't like yet those people have badges, I take note of the badge then I report back.

KC: Ok, alright. Ok, alright. Then lastly, we talked about the vaginal rings, the injectables, the long-term that are being introduced for PrEP right. There was a small discussion about them, generally what do you think about them in terms of PrEP uptake? Will they be acceptable and why, 27 that is our last question?

27: I think they will be loved because when we discussed this almost everyone mentioned that consistent, to keep on taking the pills.

KC: Uhm.

27: If something is injected and stays in your body and also you are not worried about where will I keep it and staff.

KC: Ok, uhu.

33: [Background noise] The vaginal ring.

KC: Uhh.

33: That one it sounds, I don't think its easier to use. That's how I think.

KC: Alright, so the vaginal ring …

33: "_+".

KC: So, you get inserted, and it lasts for 28 days which is almost a month then you go, have it removed and its inserted again and lasts for 28 days.

32: Will it not have any effects?

33: Exactly that if you are inserted…

KC: Ok so worry about having side effects.

XXX: Will it not be painful?

KC: To, it is…, the insertion I'm not yet sure on that one but I'm thinking it's almost familiar with the female condom, how the female condom is inserted. So, I think it will be on the same scenarios. Ok is there anyone with something to add before we close our discussion, 35?

35: I have a question or suggestion I understood "_" are these programs, is this program only focusing on people who are sexually active like young adults or girls who are sexually active? Or it also goes beyond to those who are inactive so that when they become active, they have the proper knowledge.

KC: For now, this is like, for this study its mainly focused on adolescents' girls and young women who are sexually active. That is the main focus for this one.

35: But I think uhm you should also uhm involve those who are inactive maybe on different set up or different age groups not mixing up because they will be discrimination.

KC: Ok.

35: But since we are in this generation of the young people a lot is happening, and people are getting involved in sex. Maybe we might say haa 12 years it can't, but some are starting at 12 so I think that these children need to be aware.

KC: Involve them as well.

35: When you involve them so that they are aware of what they are doing.

KC: Ok, 30 and we close.

30: I think PrEP much awareness need to be done such that when people are being taught about ABC, PrEP should be there so that people know.

KC: Ok, so to involve it when they start educating about HIV let us involve PrEP. Alright thank you so much ladies, we have come to the end of our discussion today. Thank you so much for your views, for your contributions it was an exciting discussion time ran out, but it was an exciting discussion thank you so much, so our discussion has come to an end. So, now I think the remaining things we will discuss on how best to do it, the last part, the remaining one we discussed about but our discussion has come to an end. Thank you so much.

The End
