# Supplementary material for: Perceptions of pre-exposure prophylaxis among sexually active adolescent girls and young women in Zimbabwe–A qualitative study
Source: PLOS Glob Public Health. 2025 Dec 2;5(12):e0005396. doi: 10.1371/journal.pgph.0005396 (PMC12671731; doi:10.1371/journal.pgph.0005396)
Supplement: S1 File — (ZIP) [file pgph.0005396.s003.zip › S1_File/AGYW-FGD 05-Translation.pdf]

AGYW-FGD 05-Translation
Facilitator: KC
Note Taker: FM
Date Of FGD: 24/02/2022
Age group: 17-18 years
Translator: SM

KC: Alright, so as we start our discussion, I would like to thank you for having the time and opportunity to be here today for this discussion. Like I said before my name is Kudzai, this one is Fadzai we are from CeSHHAR Zimbabwe. Today we are going to have a discussion with you on PrEP which stands for Pre-Exposure Prophylaxis, to understand the acceptance of PrEP, the reasons why they are few people taking PrEP and issues to do with PrEP uptake with young girls and young women. This will give us important information on how we can come up with new programs for giving PrEP to improve the number of young girls and young women in Zimbabwe who are taking it and who continue to use it and those who want it who are free to use it. Our discussion will take between 1 and a half hour to 2 hours to complete, but like I said before it depends with the way you will be responding. In this discussion we are going to have a role play and discussions. On role plays I will ask you to be in three groups. Each group will have a role play on young girls and young women how they take PrEP issues and their views on the risk of getting HIV. Each group will be given a description of the role play they are supposed to act out and they will be only 3 minutes to discuss and agree how they will act out their role play, right. We will then ask each group to act out and then followed by a discussion looking on what would have been acted out. So, like we discussed before, do not tell us your names use those numbers that we gave you and place them where they can be seen such that when you want to talk I can see which number you are. Such that I can say number 41 and then you will give us your point, right and then uum anyone who wants to say something must raise her hand and I will know that this one and this one want to talk so that we can give each other chance, right. So, as we start our discussion from what we have said, and the ground rules are there any questions before we start? [Silence] Alright, if there are no questions, we are starting our discussion. Have you ever heard about PrEP? Have you heard about PrEP? On this question I have asked, right, the one who respond will say yes or no. One who would have said yes will then share what she heard on what PrEP is, right, eeh, 46?

46: Yes.

KC: Uum.

46: I heard that PrEP is a tablet that is given to someone when you have tested HIV negative.

KC: Uum.

46: Also, this tablet when you get it you use it every day. If you start taking it at 6 you make sure that every day you take it at 6, you don't change the time.

KC: You don't change time, uum so if someone is using it every day is there a time that they have to use it if it is every day, is it every day of the week. Is it every day of the month is it every day for life, how is the every day like?

46: I heard that this tablet is taken every day for life.

KC: For life.

46: Yes.

KC: Alright, uum it's okay others. Is there anyone else who heard something that they know about PrEP which was not said by number 46, something different?

XXX: Aah that's it.

KC: That's it.

SOME: Uum.

KC: You are all agreeing that it's, correct?

ALL: Uum

KC: Alright, aah, what about on types of PrEP are there some who know about available types of PrEP? Available here in Zimbabwe, that are not yet available here in Zimbabwe, 46.

46: I heard that there is PrEP uum, these tablets. Then there is another one that I heard of called PEP, that one I heard it is given to someone who would have been raped.

KC: Alright.

46: Emergency.

KC: Alright, alright, so 46 has mentioned 2 different things. There is PEP and PrEP, right.

XXX: Uum.

KC: So, PrEP is used by someone who is HIV negative, one who might be having unprotected sex right

XXX: Uum.

KC: PEP is usually used by someone, let's say who has been, aah, aah, who has been exposed to risk of getting HIV the example that she gave that someone who would have been raped is the one who is given PEP that maybe the virus is already in and it would be sorted inside in the system, right. But my question was saying, on PrEP that we are talking about, on types, right, she has talked about the tablet, are there any other PrEP types that you know of that is not in tablet form? Are there any other types that you know of?

SOME: Uhm uhm [Refusing].

KC: There isn't any?

ALL: Yes.

KC: You only know of the pills, 46.

46: Not knowing if it is a type of PrEP.

KC: Uum.

46: But I once heard about a ring being placed.

KC: Uum.

46: On our private parts.

KC: Uum.

46: And also, being injected.

KC: Uum

46: And the pills that I have talked about.

KC: That's correct, yeah, what 46 has said is very correct. So, so far in Zimbabwe PrEP is available in pills, right?

XXX: Uum.

KC: What she has said, the one she explained about, but there are other things still being manufactured that are not yet in Zimbabwe. There is a ring, right here in Zimbabwe the right was only approved to be used but it is not yet available. It is not yet being used but it was approved legally that it can be used. So, the ring is placed in the vagina. The ring is like, it is like, right, you see, just like the female condom.

SOME: Uum.

KC: Yes, right, there have that ring inside.

ALL: Uum.

KC: That's what the ring is like. So, it is placed in the vagina and it will be in the system of the female person for a month. So, it will be releasing medication that will help protect one from getting infected with HIV in the system. Then there is an injection, the injection has not been approved yet here in Zimbabwe and it is not available (Baby making sounds), that is another type of PrEP where one injected and it will remain in the system for 2 months. One will be injected after every 2 months if you want, you will then be injected after every 2 months. So, those are the different types of PrEP that we have, right, uum. So, now we want to do our role plays that we have been talking about. So, what we are going to do right, we would like you to group yourselves into 3 groups. You are 12 in here, so 12 people you will be 4, 4, 4, 4, that's it right.

ALL: Uum.

KC: So, you group yourselves, get into your groups, 4, 4, 4, 4, 4. so, in your groups 4, 4, 4,4 right, we will give each group a role play that you are supposed to act out. Your

play that you are supposed to act out is this, the story you are supposed to act out is this, right. So, group yourselves and be in groups 3, 4, 4, 4, [People grouping themselves]. Even when you are seated like that and say 4, 4, 4, 4 and the remaining 4, 4, the remaining 4, 4, going on like that. [People continue to group themselves] have you finished?

ALL: Yes.

KC: Alright, so let's go back a little when we were asking about PrEP. There is a question that I forgot to ask that the information on PrEP that you have that you know so far where did you get it from? Where did you get the PrEP information? 46 [Pause].

46: I heard about PrEP from CeSHHAR.

KC: Uum.

46: And at the clinic here.

KC: Where exactly for CeSHHAR?

46: KuCeSHHAR at Mbare and as well as Selous.

KC: Alright, Mbare and Selous, what's done there? What was the program all about, what was the program about that was talking about that? Eeh, those who want to help, help, 47?

47: It was a program of taking sex workers to school.

KC: Alright.

47: Eeh.

KC: Alright, sending sex workers to school?

47: Uum.

KC: Okay, alright so, what kind of schools are these?

47: Going to the college or going back to school.

KC: Oh, re-writing "O" Level (secondary education in Zimbabwe).

47: Uum.

KC: Ooh okay alright, uum, the one behind 47, what's your number?

38: 38.

KC: 38, yes.

38: I heard about it at KATSWE and CeSHHAR.

KC: KATSWE?

38: Uum.

KC: KATSWE what is KATSWE?

38: It is another organisation.

KC: What does it do?

38: It also works with sex workers.

KC: Oh, it works with sex workers, so, KATSWE, CeSHHAR is there another one? 47 there is another one you mentioned that is not CeSHHAR.

47: Here at the clinic.

KC: Oh, at the clinic here?

47: Yes.

KC: What kind of information was being given? Were they just giving PrEP information or there was something that was happening mainly focusing on PrEP.

47: No, when you want to get PrEP here at the clinic what I heard of.

KC: Uum.

47: They ask questions first; you don't just go as an individual and say I want to get PrEP.

KC: Alright.

47: Without any reasons of getting the PrEP.

KC: Alright. Okay is there anyone who heard about PrEP somewhere besides, KATSWE, CeSHHAR, at the clinic here, anywhere else? Yes, what's your number?

39: 39.

KC: 39 uum.

39: In the community.

KC: In the community.

39: Uum.

KC: What was being said in communities about PrEP?

39: They were just encouraging women to go and get PrEP.

KC: Who was encouraging you?

39: Sex workers.

KC: Alright, alright, okay, okay. Alright so, we are now getting into our role plays, right.

ALL: Uum.

KC: So, our role plays what we are going to do we have three role plays, so the first one is, it is a story of two friends, two friends right.

ALL: Uum.

KC: We are calling them Chido and Koko. Chido is 16 years old. She is having a sexual relationship with an older person over 50 years. She just started PrEP since she thinks that she might get infected with HIV. She also thinks that her friend Koko who is 19 years old is also at risk of getting HIV because she is having a sexual relationship with an older person as well, right. She tells her friend Koko to consider taking PrEP because of this and that. So, the role play between Chido and Koko is that we want to see Chido telling Koko about PrEP that aah I'm now taking PrEP because of this and that. I'm thinking that you could take PrEP as well, then we will hear Koko's

response what she thinks about taking PrEP. Does she want, if she wants it then she will give her reasons. Doesn't she like it, if she doesn't like it and give reasons, right.

ALL: Uum.

KC: That's the first one, the second one has two young women who are married, mai Bhobhi and mai Juru. Mai Bhobhi is 23 years and she is married, mai Juru is 21 years and she is married. Mai Bhobhi's husband sometimes have girl…girlfriends most of the time. So, mai Bhobhi is stressing that she might get infected with HIV. She heard about PrEP on the radio right, so, she started taking PrEP she now has 6 months taking PrEP. She is now thinking of stopping PrEP. So, we want a role play between mai Bhobhi and mai Juru, mai Bobhi bringing out the reasons why she is considering stopping PrEP. She had used it for 6 months, but she no longer wants to continue using it, that's the second one. The third one, is for 3 friends, 3 friends right. These three friends go to the same school, and they all have boyfriends. At their school they have been selected to come up with a program, a PrEP program that will motivate young girls to use PrEP. So, the program there are supposed to come up with right, what they are supposed to focus on, is a program that they will be looking at things like where they want the program to be, what kind of places. What kind of people should distribute PREP, on the issue of price what price do they want PrEP to be, something like that. They should come up with a program that when it's said here is the program the young girls will come for PrEP because of the program that will be in place right. So, those are the 3 stories that we have. So, that is how we are grouping each other on these role plays, so which one is group?

XXX: This one.

KC: [Laughing] No, just talk it's me who is going to give you role plays. Just do it, which one is group 1?

XXX: We are group 1.

KC: Group 2. Group 3.

XXX: We were group 1.

KC: So, group 1.

XXX: Group 1.

XXX: Group 2.

KC: Group 2, group 3. Group one up to where? All of you?

XXX: Uum.

KC: Then these ones like this, okay that's fine. Group 1 you are doing Chido and Koko, group 2 you are doing mai Bhobhi and mai Juru. Group three you have it you are doing the one for three friends.

KC: So, these ones like this, and these ones like this.

XXX: We have two.

KC: Alright, have this one for three friends, Peppa, Princess. Right, so we are now giving you 3 minutes, two to three minutes to discuss your role play that we are going to do this to…who is going to act out because the group…Chido and mai Bhobhi groups there are 2 people only who will act out. So, you choose who is going to act out, this one and this one and have a discussion that we are going to talk about this and this, right, so three minutes

[Talking in the background as groups as rehearsing their role plays]

KC: Right, so we now want to start with Chido and Koko's role play, we are asking you to come… to do the role play, so those who are also doing… the 2 acting out stand up and do the role play as you raise your voices.

XXX: Aah let these ones start, we are yet to finish.

KC: No, Chido and Koko you are the first group. Chido and Koko you do your role play first. As they do others we will be listening, right. If you have any questions that you would like to ask you will ask that part that did not come out well or where it is not clear, we will ask.

XXX: Okay, we are starting.

AGYW-FGD 05-Translation
Facilitator: KC
Note Taker: FM
Date Of FGD: 24/02/2022
Age group: 17-18 years
Translator: SM

Chido: Hi Koko.

Koko: Hi, Chido how are you?

Chido: I'm alright, it has been long what's up?

Koko: Nothing my dear.

Chido: You are not available my dear.

Koko: I'm available.

Chido: My dear, we are looking for you in the streets, but we are not seeing you. How is your old man?

Koko: Aah he is fine, that's another issue that is bothering me. He is refusing to get tested I'm at risk, I don't know what to do.

Chido: Uhmm but my dear, were you not seeing that old man is old?

Koko: Aah we just wanted money my dear what can we do?

Chido: Uhmm my dear do you have protective measures in place?

Koko: That's what I have been wondering what I can do.

Chido: My dear, there is PrEP, it is available nowadays, it's a pill.

Koko: Oh, what is it like, that one?

Chido: It is a tablet; it is a tablet my dear you can get it on monthly basis my dear.

Koko: Oh, if I want to get it today, I can get it?

Chido: Yes.

Koko: Oh.

Chido: You can go my dear.

Koko: Does it prevent me from getting infected with STIs?

Chido: Yes.

Koko: Alright.

Chido: So, I will see you.

Koko: Alright.

[Participants clapping]

KC: That's fine, thank you Chido and Koko right, right, so, we now want to look at Chido and Koko role play, right. We have heard their story, aah and their role play we have all heard it. The first thing that I want to ask is that in real life are there young girls who…or young women who have sexual relationships with men who are older than them?

Most: Uhh.

KC: Are there any?

Most: Uhh.

KC: Right, anyone who say yes, may you explain, there are there, who are like this and this they get into these relationships because of this and this. We want to know what causes them to be in those relationships. 42.

42: Other reasons for one to be in a relationship with older people it is because they want money.

KC: Uhh.

42: The sugar daddy are the ones with money so, she will see that if I fall in love with a sugar daddy, he will be able to take care of me.

KC: Alright, on the issue of money what do they want to use the money for?

42: Money to use and look after the family as a young lady you would have had a child while you are still young so you would like to look after your child.

KC: Alright, okay 46.

46: Sometimes it is not that I can fall in love with the blesser that I don't know. Maybe your father is late, maybe I am staying with my mother's brother.

KC: Uhh.

46: Your mother's brother (uncle) is the breadwinner at your home, then he says to you for him to do everything here, taking you to school, buy you food, you must have sex with him.

KC: Alright.

46: Uhh.

KC: Alright, so, those are some of the situations. Alright, yes others, what's your number?

45: 45.

KC: 45.

45: For us young girls falling in love with sugar daddies when we are on touchline, at beer halls.

KC: Uhh.

45: Sugar daddies want young girls they say they are the best.

KC: Best at what?

XXX: Everything.

XXX: They are still growing up.

XXX: They are still active.

KC: Alright, uhh.

45: So, due to the need for money if you get your sugar daddy that would be fine.

KC: Uhh.

45: If you say I want this he will do it for you, if you fall in love with a sugar daddy even your child will be able to go to school.

KC: Alright. Okay, you mentioned that when you are on touchline, what is a touchline?

45: That's where you wait for clients.

KC: Oh, alright, alright. When people are looking for their clients.

45: Yes.

KC: Alright, okay so this one you have talked about is linked to sex workers?

45: Uhh.

KC: Alright, is there anything else that makes them in… yes? 40 what… what's your number, 38?

38: The other issue that is causing that is staying in a one roomed house.

KC: Uhh.

38: Father, mother and children all in there, so the child will be exposed to everything that will be happening, and hearing what will be happening [Someone speaking from outside].

KC: Uhh.

38: It leads her to accept when another man propose love to her and they will fall in love.

KC: Alright, alright, 37.

37: Sometimes the friends that you play with, you may have a friend who is in love with a sugar daddy, right. She will be getting everything, yet you do not have. At times she will say let's go and see my sugar daddy, and the sugar daddy will have his friend.

KC: Horaiti.

KC: Alright.

37: You will be given to the other because good things will be done for you.

KC: Alright, okay. Alright, moving forward do you think someone like Chido is at risk of getting HIV looking at even the situations you have talked about that makes young girls get into relationships with older people. Is she at risk of getting HIV?

All: Uhh.

KC: Where is the risk, where is the risk shown? 46.

46: The risk is the sugar daddies, these sugar daddies.

KC: Uhh.

46: He may say that I'm doing everything for you so on…when we have sex, I don't want us to…use protection of which HIV can be passed through that when the fluids get mixed. You will definitely be infected no deals so she will be at risk because that's what usually happens, the sugar daddies do not want to use protection.

KC: Alright. Okay she said they do not want to use protection because they will be saying I'm doing everything for you. Where else is the risk that they are at these young girls that they will be infected with HIV, where else? Uhh, 46.

46: Sometimes one might say…one might say alright I'm now putting on the condom and then you will not…you will not check if the condom has expired or not…he just put it on. Or one might just break the condom, and you will be at risk of getting HIV and pregnancy on top of that.

KC: Uhh, uhh alright. Are there any other risks that are common in relationships like these for people like Chido? [Silence]. On the reasons that you gave, it was mentioned that some do not want to use condoms because they will be saying I'm doing everything for you, so I don't want to use a condom. Are there any other reasons why such men do not want to use condoms in such relationships besides that they are doing everything for you? Are there any other reasons why they do not want to use condoms? 46.

46: I just want to share what happened to my friend.

KC: Uhh.

46: My friend, I was learning in XXX, so she was staying in XXX, and she was in love with the sugar daddy.

KC: Uhh.

46: So, the sugar daddy when the time comes for them to have sex, the sugar daddy would say what, I don't want to have sex with you using condoms. Of which that sugar daddy was already HIV positive.

KC: Alright.

46: As we speak that girl is on ART plus, she has a baby with the sugar daddy.

KC: Uhh.

46: So, sugar daddies already know that they are infected and they would like to just fix you.

KC: Why, why would he want to fix you?

46: Just being cruel to you.

KC: Why being cruel [giggles], I'm asking everyone. Why do you think that let's say, maybe the blessers, these sugar daddies, or males they… yes 46.

46: Sometimes, if you…one may take advantage of your situation.

KC: Uhh.

46: That even if I infect you, I have money I use that to my advantage that I have money so there is no where you will report that case. If you report that case I can even you we go to court, a lawyer can represent me, and you will be on your own.

KC: Alright.

46: So, one will take advantage that I have the money, I'm a blesser with money. And you don't have even if I do anything there is nowhere you will report.

KC: Alright.

45: Uhh 45.

KC: Yes.

45: The sugar daddies may have been infected by others so he would like to spread to others, and he will for sure spread it.

KC: So, they want to infect others because they are infected…were infected.

[Baby crying]

XXX: Uhh.

KC: Alright, are there any other reasons that you think or that you know? [Silence]. Alright, it's okay. Aah, looking at the young girls those that are still growing up, right we would like hear how they share their stories personally. Are they the kind of people that are free or is it something that happens that young girls can share their health issues with other people? Or say, I have decided to get tested for HIV, I have decided to get tested for an STI, or getting treatment, is it something that they can do, can they share this information, is it easy to share those issues? 40, what?

41: Haa, it is not easy…41.

KC: 41, uhh.

41: Some will move around spreading people's stories.

KC: Alright, so it is not easy because they fear that their stories will be spread.

XXX: Uhh.

KC: Uhh, 46.

46: Sometimes, I would just like to support the point that has been raised by this one. At times if you mention that girls I have been to the clinic, I have gone to collect the condoms what comes to their minds is that, that is something that is done by sex workers.

KC: Alright.

46: It is usually done by sex workers.

KC: Uhh.

46: So, they will move around saying that…just like we are here, if I share my story, maybe it would be my secret, and I had just shared to seek help from people you might hear about it… it would be hot in the community. That, have you heard that, this one is like this, this.

KC: Alright, anyone else with other thoughts on this issue sharing health information? [Silence] But are there any other people that they share with, there are people that you…do they have their people that they say haa, on issue to do with health I have these ones. If there are there, what kind of people are they? Yes, what's your number, 38?

38: 38.

KC: Uhh.

38: For others it is easy if you have the knowledge of whom you must share your story with, if you get infected with STI, there are home based carers in here.

KC: Uhh.

38: You can go to those people.

KC: They…

38: And share your story.

KC: Are they easy to approach in the community those home base care?

38: Yes, there are easy

KC: Are they easy to approach?

38: Yes, they are easy.

KC: What about on confidentiality issue, do they keep the stories confidential?

38: Yes, they keep them confidential because that is their work.

KC: Alright, others. This one has highlighted that you can approach community home based care, are there any other people that young girls can share their information with such people who are not home-based care? What about friends?

All: [Disagreeing] Haaa noo.

KC: It doesn't come out.

All: Mumbling in disagreement.

XXX: They are the ones who spread the word.

KC: What about parents? 47.

47: Maybe I can be able to share with my mum.

KC: With your mum?

47: Or with my grandmother my mum's mother.

KC: Those are the people you can share with.

47: Not everyone else.

KC: Alright, uhh, what about people from church?

All: [Disagreeing strongly] Uhmmmm

XXX: You will be the one being preached at church.

KC: Alright, so church members are not an option.

All: Yes, there are not an option.

KC: Alright, alright, what can we say, others. Boyfriend, sister, brother from your family?

XXX: Aah it can't.

XXX: Those ones are not right.

KC: Alright, so we have come up with community home based care and parents only.

XXX: Uhh.

KC: Alright, laughs. Alright, looking on the issue of information, there is a time when I asked about PrEP information and where you are getting it, right, and you answered that. Let's say you have been asked to suggest where information and be found, what kind of places young girls can easily get information. Getting PrEP information, where can that information be found, somewhere you can suggest that this place, and this place provide PrEP information easily by young girls, which places, 38?

38: Uhh.

KC: Uhh.

38: Here at this clinic.

KC: At the clinic.

38: Uhh.

KC: Alright, what is good about the clinic?

38: The clinic is good in that if you come looking for information, they will tell you why PrEP is good for you to use it, and you will have enough information.

KC: Alright, okay at the clinic. Are there any other places, 46?

46: Doing roadshows moving around sharing information on PrEP because not all of us are able to come here to the clinic. All of us, XXX all of us, we don't manage to come here but let's say there is a roadshow at the XXX, doing your awareness campaigns, someone washing plates may pay attention to what is being shared at the road show.

KC: Okay, at the clinic and roadshows. What else can be used? [Silence] Alright, it's okay. Aah, ooh, 38.

38: Even in schools.

KC: In schools?

38: Uhh.

KC: In schools like which ones? Schools are different, yes, finish.

38: At colleges and secondary schools, at secondary they know about sex.

KC: Alright, uhh. Anyone else? [Silence] alright moving on right, aah we now want to look at… Chido and Koko's role play. Right, we have all seen the role play of Chido and Koko. Chido has told Koko about PrEP, right, what do you think about Koo's response when Chido was telling her about taking PrEP? 46.

46: I want to ask something from the point that they have raised.

KC: Uhh.

46: When they said PrEP protects one from STIs, what PrEP can only do is it protect one from HIV, but you can be infected by all other STIS.

[People talking in the background-outside at the reception]

KC: Uhh, that's correct. Yes, 38.

38: Plus, there is another point there, this one said its only taken per month.

KC: Uhh.

38: PrEP, it was said PrEP is for life.

KC: Alright.

38: This one said its only taken for a month.

KC: Is there anyone else with a point to add on [Silence]. Alright, so, I will… when we finish, I will give more information about PrEP, the way PrEP is being taken currently in Zimbabwe. When someone is taking PrEP how is it done, how long does one have to take it, right, but for now let's move on. Aah, looking at Koo's response, is it exactly as in real life, in real life Koko's response.

46: What did she say, we have forgotten.

KC: You have forgotten?

[Baby crying]

KC: [Chuckles] alright, Koko may you help me if I miss anything, so Koko on… when she was told about PrEP at first she was asking if PrEP protects one from HIV and she was told that yes, it protects from HIV and she showed that she is interested and she might want to take PrEP so that is the response we got on…from Koko so that is what I'm asking is that can happen in real life when someone is approached? 46.

46: Personally, I just want to add, yes, I want to get PrEP. I will not just accept, I will first ask how it is taken and how it works.

KC: Uhh.

46: Then you tell me.

KC: Uhh.

46: Then if I agree with it, we go, if I don't agree with that, I will stay like that.

KC: I will stay like that, yes, 38.

38: Most some people do not agree, if they hear about PrEP.

KC: Uhh.

38: Because at the reception where HIV treatment medicines is collected that is the same place where PrEP is given as well so some will not agree. She will take time refusing saying if I'm seen at the reception, people will say I have HIV. Haa, it can't PrEP no, she can refuse.

KC: Alright, she will be said she has what?

38: HIV.

KC: Okay, so, co-location of PrEP and HIV.

38: Yes.

KC: Alright, anything else? Even some, other responses that you can think of that have not come out in the role play, 46?

46: Personally…

KC: Others let's talk.

46: On the point that has been raised by this one.

KC: Uhh.

46: I once took out my PrEP container to my friends.

KC: Uhh.

46: I was saying do you know these pills girls they are called PrEP. And they said what kind of pills are these ones, these pills are just the same as the AIDS ones, and I said no, there are different from AIDS tablets. If you want them, I can give you one bottle, and you use.

KC: Uhh.

46: After that the following day I heard it being widely gossiped that aah she is taking HIV treatment pills, she even brought the AIDS tablets here.

KC: Okay, alright. Uhh, others, other responses that we can get from Chido and Koko situation that have not come out of the role play. The responses could be agreeing or refusing, reasons… let's say, let's start with agreeing. What are the reasons for one to agree after being told about PrEP? Any motivators for one to agree, are there any? 46?

46: At times, not agreeing, maybe you would have gone with your partner. And you are told about PrEP maybe he would have been told that PrEP is a tablet that is used by sex workers only.

KC: Uhh.

46: So, if you tell him about PrEP when he is your boyfriend he will ask if you are a sex worker.

KC: Alright.

46: But PrEP is given to everyone who uses, who has sexual relationships with more than one person.

KC: Uhh.

46: So, if you tell him that he will regard you as a sex worker because PrEP is taken by sex workers only.

KC: Alright, alright, so this word that…let's say when you heard about PrEP for the first-time what kind of people were you told are supposed to use PrEP?

XXX: We were told that sex workers.

KC: You were told that sex workers are the people who use PrEP?

XXX: That's what we heard.

46: I heard that sex workers.

KC: Uhh.

46: And also, by people, women who are married but their husbands are having other affairs outside their marriages. She has the right to use PrEP because she is able to… she has a chance; she is the one who is at high risk of getting infected with HIV because she doesn't know what her husband went out and did, she doesn't know what transpired. The husband has just come to have sex with her wife without protection because if you want to use protection when you are married you will be asked if you are doing something else.

KC: Alright.

46: Uhh.

KC: Alright, looking at what we call benefits of PrEP to young girls, what are the benefits for one to use PrEP? What are the benefits of using PrEP, what are the advantages of using PrEP? Why is it good in your opinion?

45: 45.

45: PrEP is good in that if I take it, it protects me from getting infected by HIV.

KC: Uhh. Alright, it is good in that it protects one from getting infected with HIV. Are there any other benefits of PrEP that did not come out here, yes 46?

46: I want to support that point.

KC: Uhh.

46: If you are taking it, you may take it at different times, that pills want to be used consistently at the same time.

KC: Uhh.

46: Because if I take it today at 6 o'clock in the evening.

KC: Uhh.

46: That pill will protect me until what…

KC: At 6.

46: Up to 6 in the evening, and I will start taking it again. It needs consistency and also it doesn't need to…

KC: Alright, so if you start taking it you continue taking it going on like that.

46: You continue taking it.

KC: Alright, okay. Uhmm what about barriers for the uptake of PrEP, what are they, especially looking at adolescent girls and young women, what can stop them from taking PrEP? 44.

44: Barriers, we once heard about it from people who are taking it.

KC: Uhh.

44: They said it you take it right,

KC: Uhh.

44: If you stop taking it and you are unluck and meet someone who is positive and get infected when you have stopped taking it.

KC: Uhh.

44: For you to then take HIV pills, they said the pill will not work in your body.

KC: Oh, they said they will not be working.

44: Uhh.

KC: Alright, 46.

46: I heard that these pills will affect you I don't know what is called gall bladders

KC: Alright.

46: They said if you continue taking it for the rest of your life you will end up not having a gall bladder because when you…when you take it, it will be destroying your gall bladder bit by bit.

KC: Alright.

[Some members chuckling

KC: Alright, others what hinders people from taking PrEP or to hear about PrEP what, is it, something that has not been said, 45.

45: 45.

KC: Uhh.

45: This pill they say if you take it and stop taking it and then have sexual intercourse with an HIV positive man you will get infected at once.

KC: Alright, so, when you have been taking PrEP and stop taking it you increase your chances of getting infected with HIV?

45: Uhh.

KC: Alright 47.

47: I heard that when you are taking PrEP and you leave it, you develop side effects, you will have a skin disease.

KC: What kind of skin disease?

XXX: It is called rough skin; rough skin is having two skins on one person.

KC: Oh, okay, alright, 46.

46: Sometimes, the reason why people stop especially in this community.

KC: Uhh.

46: I will be seen holding my bottle, people do not know that the bottle with a red line I don't know, is it maroon, its PrEP. The one with a blue line is for HIV.

KC: I mean HIV.

46: Someone who doesn't know, if they see me holding that container, she will spread that saying have you seen her, she is taking these tablets.

KC: Oh.

46: Plus, what I have heard is that if you take this tablet for three months without having sex. If you would have another month without taking it you are not at risk of getting HIV because that tablet will still be working in your body.

KC: Oh, it will still be working in the system. Alright, so, everything that you are saying that I heard this, I heard that, where did you hear all that. The people who were saying that are they the people who use PrEP, where did you hear all these PrEP issues? 47.

47: I heard from someone who has been using it because I saw her with a skin disease.

KC: Alright.

47: She said she was reacting to it.

KC: Oh, so, there were the side effects that she had.

47: Uhh.

KC: Okay. 46.

46: Personally, when I heard that it destroys the gall bladder.

KC: Uhh.

46: I thought of stopping taking it, I said I will live my life without a gall bladder because of a pill. I was told by someone who is taking that pill saying she was about to stop taking it because I'm always sick and I don't know why, maybe my gall bladder has been destroyed.

KC: So, she started getting sick after she started PrEP?

46: When she was taking it.

KC: When she was taking it.

46: Yes.

KC: That's when she started getting sick all the time, okay, alright. Aah, moving forward, right, we now want to look at adolescent girls and young women where can they get PrEP services. When can they go and get PrEP? Where could we get…currently we start by where it is available in our country, where do adolescent girls and young women get PrEP? 45.

45: At the clinic.

KC: At our clinic, 47.

47: At CeSHHAR, in Mbare there.

KC: At CeSHHAR, in Mbare?

47: Uhh.

KC: Are there any other places? [Silence]. Alright, any other places that you can suggest right, aah alright, we will start with the places you have suggested.

XXX: At Spillhouse (Hospital).

KC: Where?

XXX: At Spillhouse.

KC: Oh, Spillhouse. Alright, these places that you have mentioned, it is available currently right, is it going on well? At Spillhouse is there something that is not going on well that is stopping adolescent girls and young women to go and collect PrEP? Is there something that is not going on well, 45?

45: At the clinic.

KC: Uhh.

45: That's where they say PrEP and those other tablets, they give them at one place.

KC: At one place.

45: So, if we are seen in the queue umhh, it can't.

KC: It doesn't come out well. Alright, 46.

46: It has negative effects.

KC: Uhh.

46: It is because when you go and say I want PrEP.

KC: Uhh.

46: They will ask you your reasons for wanting to take PrEP and you remain silent.

KC: Uhh.

46: They will not give you PrEP, and also at XXX, they ask you if you are a sex worker, for you to what…

KC: To get PrEP.

46: To get them, if you keep quiet, you do not have a reason for taking PrEP. So why do you want to take it without any reasons?

KC: Alright, so if you don't answer the… the questions that are asked you will not get PrEP.

46: Yes.

KC: Alright. Is there anything else that is not working well? Alright it's okay, what about what is working well at those places that you mentioned… [Baby crying] CeSHHAR, Spillhouse and the clinic. What is working well on the distribution of PrEP, what do you see and what do you know that can encourage adolescent girls and young adults to continue coming to willing to use PrEP? 44.

44: Not paying for it.

KC: Not paying for it.

XXX: Asking questions and getting answers as well as getting information on how to take it.

KC: Sensitization about PrEP before you are given it. [Baby crying] Alright, okay. Now we want suggestions on places that you think are perfect for adolescent girls and young women to get PrEP [Baby crying]. What other suggestions can you give, that these places are good for people to get PrEP from. Yes 44?

44: Here at the clinic but it doesn't have to be on the same side where ARVs are being given.

KC: Alright, they have to be split.

44: Yes, that this one is that side, those on ART that side and this one this side, not at one place.

KC: Alright, it is okay.

44: And you will just say I'm here for this, and this.

KC: Alright, 46.

46: Like, you XXX people sometimes

KC: Uhh.

46: You just come even at XXX, moving around with your car.

KC: Uhh.

46: Educating people about PrEP those who would have liked it you then give them there and there.

KC: Alright.

46: Like I said that it's not the whole of Hopley that will be able to come to the clinic queueing to get PrEP, PrEP because…Right, at the clinic they will be doing it at their own time because they will be working with time.

KC: Alright, so we come, looking for a spot down there and tell people and anyone willing will then get PrEP there.

All: Uhh.

KC: Alright, it's okay. Are there any other places that we can think of? [Silence] What about at a pharmacy, pharmacy.

XXX: It will then be sold.

46: At the pharmacy it will be sold.

KC: Let's say there is a PrEP program that is being given at the pharmacy but not being sold, being given for free?

46: Aah local pharmacies to tell you the truth, you might buy it for dollar for container to get it.

KC: But what if it's said…

46: I bought it for dollar, there is nowhere I will report it because I will just think maybe that's what the pharmacy people do that PrEP is sold for dollar or what…

KC: Alright, but let's say it has been put in the pharmacy, it is not supposed to be sold but given for free. Is the pharmacy appropriate for the adolescent girls and young women to get it from the pharmacy?

46: Yes, it is appropriate.

KC: What are the reasons why it is appropriate? What could be the reasons to get PrEP from there?

XXX: No one will know that you have been to the pharmacy to get PrEP, they will just think that you are buying something else, and you go.

KC: Aright, so it is good in that it… it…

XXX: For privacy.

KC: For privacy no one will know your reason for going to the pharmacy, yes 45.

45: And there is no way the person in the pharmacy will move around saying I saw this person collecting PrEP.

KC: Alright uhh. Anyone else on pharmacy? [Silence] what if we look for, there is someone who talked about the community health workers, in the local area, is it fine getting PrEP from them in their homes?

All: Haa it is not possible.

XXX: They would rather sell it.

KC: Uhh, others. This one has said they might sell, 44.

44: It is not possible, if you go there to collect from them, they might have their friends and they will say, this young girl who just left so she is sexually active.

46: Yes, they would be saying why do you need the PrEP for, a young lady like you.

KC: Alright, okay so they will start talking about why you need PrEP as young as you are. Any other reasons why it is not appropri… appropriate? [Silence]. Alright, so we now want to move on to role play number two for mai Bhobhi and mai Juru, right. So, like I have explained before that mai Bhobhi and mai Juru are friends who are… there are married but mai Bhobhi's husband has girlfriends, she has started taking PrEP and she now has 6 months taking PrEP but she is thinking of stopping the PrEP right, so we now want to have the role play. We now want to have mai Bhobhi and mai Juru role play. We now want to do the role play.

Role play 2

Mai Juru: *Hi, mai Bhobhi.*

Mai Bhobhi: *Are you ok my dear?*

Mai Juru: *How are you?*

Mai Bhobhi: *Haa, I'm not fine, something is happening that I want to tell you.*

Mai Juru: *What is the problem.*

Mai Bhobhi: *I'm thinking of stopping PrEP.*

Mai Juru: *Why my dear?*

Mai Bhobhi: *My husband is not stopping being promiscuous; he is sleeping with many girls.*

Mai Juru: *So, that's what is motivating you stop taking PrEP?*

Mai Bhobhi: *Ah, I am just thinking of stopping my dear.*

Mai Juru: *No, my dear do not stop, keep your health. Let your husband do what he is doing while you take your PrEP keeping your health.*

Mai Bhobhi: *Ah but it is not easy, uhmm will do like that.*

Mai Juru: *Yes, do not stop taking PrEP.*

[Baby crying]

[Other group members clapping for them]

KC: Alright, thank you mai Bhobhi and mai Juru right. Aah aah, … [Some participants mumbling]. We are now doing a brief discussion on mai Bhobhi and mai Juru right. Looking at their role play and asking some questions that we want to come out on such situations right. So, the first thing that I want to ask is that do you think in real life, in real life are there… [Baby crying] Young women like mai Bhobhi who are in situations like mai Bhobhi's that their husband would have many girlfriends or other people they have sexual relations with, yet she is not her wife. Is it common?

All: Yes.

KC: Explain how the situations are like, 45.

45: Uhh, situations, situations like this one, it is rare for a man to have sex with one wife at his place.

KC: Uhh.

45: They will go out at one time to have girlfriends so that cannot stop anyone from taking her PrEP.

KC: Uhh, alright, others. Is there a difference, she said it is common a man can have girls friends going on like that? 46.

46: I just want to support that point raised by this one, sometimes I don't even stop taking PrEP because I will be aware that my husband is in love with this one. Maybe we will be aware of the status of that person he is in love with, her status, her status. Then you hear that your husband is going there without protection, I have the right to take PrEP every day because I will be… I will be fearing for my life so that I will not get infected with HIV.

KC: Alright, it's okay. Do you think it would be easy that a lady like amai Bhobhi who is married, adolescent girl who is married like mai Bhobhi would it be easy to get to a decision that I'm now starting to take PrEP. 45?

45: Yes, it is easy.

KC: Yes.

45: If the husband has many sexual partners, it is easy for her to take PrEP and protect herself.

KC: Alright, they said it is easy because she might want to protect herself.

44: I think it is not easy because our husbands are hardheaded.

KC: Uhh.

44: He will not understand that its PrEP because he has never learnt about it or anything.

KC: Uhh.

44: He will be saying that maybe you are now on ART in my house without my knowledge. You went out and got other partners so now you want to take your medication for yourself.

KC: Alright, he might not understand what PrEP is for.

44: They must learn first so that they know. Even if they become promiscuous but they must know what PrEP is for.

KC: What it is used for. Alright, others. One said it is easy, and the other one said it is not easy, is there anyone with a different view. [Silence] Alright, looking at married young women what could motivate them to take PrEP, what we haven't talked about, is there anything that can encourage them? Not what 46 has said that she will be saying my husband is doing this and that, so I want to protect myself. Is there anything else that can encourage them? [Silence] Alright, what about barriers, the barriers something that can hinder them from getting PrEP in their homes, 45?

45: I want to talk about what 44 said.

KC: Uhh.

45: Maybe the husband doesn't understand about PrEP.

KC: Uhh.

45: So, it might hinder her from taking PrEP, the husband would think that its ARVs.

KC: Alright, he will not understand. is there anything else, 46?

46: Some other man when he realises that you are taking medication, he will ask you why you are taking the medication did you hear that I have other partners. Did you hear that there is something that I'm doing, medication is not allowed in this house?

KC: Alright, okay. What about looking at that mai Bhobhi now wants to stop taking PrEP, right. She no longer wants it, but she has 6 months, but she is thinking of stopping it

right. What reasons could be there for… young women who are married or young women in their lives will it happen that one after taking PrEP decides that I no longer want to take PrEP completely. Is it possible? [Silence] Is it possible? [Silence] Is it possible that one who would have started PrEP is no longer interested, she wants to stop? 46.

46: Yes, it is possible because sometimes what causes me to stop like I said before that one person may come and say the medication you are taking has side effects. And another one would say, like I said that lady it causes skin disease.

KC: Uhh.

46: The other one will also come saying whatever she likes so I will be saying it is better to stop taking the medication rather than having…have other different diseases because of PrEP.

KC: Alright, alright, okay. Are there any other reasons, other reasons that can cause the…adolescents to stop PrEP besides side effects, are there any other reasons? [Silence]. So, side effects are the only reason for one not to take PrEP? Anything else that is not side effects.

45: One might not want people to know that she is taking PrEP, when people know about it, she will say I'm now stopping.

KC: Why but…does she want to stop because she doesn't want to be known?

45: You will be labelled as a sex worker.

KC: Alright, fear to be called a sex worker.

45: Uhh.

KC: Alright it's okay. What could be done to encourage or to help young girls who would have started taking PrEP to continue taking it, so that they will not go up to the stage of saying I'm now stopping? What can you suggest that if this is done it would help, 47?

47: If you threaten her that if you stop PrEP, if you have unprotected sex, you will get HIV.

KC: Alright.

47: She might be afraid.

KC: Okay. 46.

46: I would ask that before the PrEP is given, before you are given it, haa it has to be clarified.

KC: Uhh.

46: There is need for information giving from step one to the end so that I will understand because if I take it, even if I'm threatened, I will take it. If I go to my community or to my friends aah just talking about it they would say oh, you have started with those pills of yours. Yet I would have been given it here without any explanation, I will stop taking the pills.

KC: Alright, so if I get enough information on the pills, it will help me. Alright it is okay. We now want to move on to role play number 3, for the three friends who are making a program for PrEP that will make it possible for adolescent girls and young girls be able to get PrEP willingly. So, on this one we would like you to do it like you are listing like say, the PrEP program must have this and that going on like that, so role play three you can come now.

[Role play participants mumbling.]

XXX: Can we do it now?

KC: Yes, do it.

Role play 3

Princess: *How are you women.*

Sky &Peppa: *Good how are you?*

Princess: *I just heard that there are tins that make a sound, what are those tablets that you move around with, for?*

Peppa: *Aah they are AIDS tablets my dear.*

Sky: *Uhmm, guys wait let me tell you about this PrEP. This PrEP is good, we were happy that we now have something called PrEP here in Zimbabwe because if you go to the clinic or to our organisations like CeSHHAR, Katswe, that have been talked about. If you get there you don't just start taking PrEP, you first get tested to know your status, if you test HIV positive you will not be able to take PrEP. If you are HIV negative, you will be able to take PrEP. Also, when you know that you are HIV negative and your partner is HIV positive you can be together because you would also have tested HIV positive, that's not the end of life. You can as well go to the clinic or to CeSHHAR for your viral load check and also if it is low, we can… if it is low and your partner is HIV positive, and his viral load is low and I'm HIV negative using my PrEP we can survive without any protection. No infection will come to me, so women this PrEP is good because we protect from HIV. But I would like to tell you that it will not protect you from STIs, let's not move around lying to each other that PrEP protects us from STIs. It only protects from HIV so your life will be on the safe side.*

Princess: *So, you get the tablet for free?*

Sky: *It is given for free, no payment.*

Peppa: *Aah, I have heard that women, I will go and look for PrEP to protect my health.*

Sky: *Iih, women let's go in our numbers let's not affect our lives ... when we take the pills.*

[Other participants clapping hands for the group]

KC: Thank you, group three right, so we now want to discuss this is our last role play. We just want to discuss. Uhmm, do you think the program they have acted out will work

out for the young girls to get PrEP willingly? Will it work, will it be, ok? There are some who haven't talked yet, 47?

47: It will work because you would have been given advice by your peer.

KC: Uhh.

47: So, it will work out.

KC: Alright, you would have been given advice, having been told about PrEP, others, 45?

45: Uhmm, it… it will work out because she would have educated them correctly.

KC: Uhh.

45: And if you understand it you will think of taking PrEP.

KC: Alright, uhh. Others 46.

46: This PrEP program teaches because maybe I would be in love or my partner would be HIV positive.

KC: Uhh.

46: I cannot… to discriminate him because some people discriminate if they hear that someone is HIV positive, they will not even want to sit close to him.

KC: Alright.

46: So, I will be…when I go to collect PrEP, he will be going to check his viral load. If it happens that we manage to get married, if his viral load is checked and is low, I will be taking PrEP every day. We can have sex and have a child who is HIV negative.

KC: Alright, okay. Looking at important things that will make the PrEP program successful, PrEP program for adolescent girls and young women for this program to be successful. A program that will be liked by the girls, what would you say this and that should never be missing in the PrEP program. What would you tell us as important things; we want it in our PrEP program, what are those? [Silence]. Let me look at the age of someone who will be distributing PrEP, is it important?

All: No.

KC: It is not important.

All: Uhh.

KC: Alright, what about the price of PrEP?

XXX: It should not be sold.

XXX: It should not be sold.

KC: It has to be free.

All: Uhh.

KC: Alright, looking at privacy and confidentiality issues, are these issues important in the PrEP program?

XXX: Uhh.

KC: How important is it, would it…how would we get privacy and confidentiality? Who would we get it, 46?

46: Right, we are saying PrEP, this tablet you will not just take it before you get tested [Baby screaming].

KC: Uhh.

46: When I get tested, I should not go where there are a lot of people. I should go to a private place for me and the person testing me.

KC: Uhh.

46: At times it's not everyone who tests HIV negative.

KC: Uhh.

46: At times I may have tested HIV, what, positive.

KC: Uhh.

46: Then I will be told what we have been talking about the viral load.

KC: Uhh.

46: But if I had tested where they are a lot of people, haa everyone would move around saying, haa she is positive. So, privacy is important and being told that if you test HIV negative.

KC: Uhh.

46: You are then told the benefits of PrEP, and the rules that you must follow. If you test HIV positive.

KC: Uhh.

46: You will then be told that it is not the end of life, you can come for viral load check and other things. You will also be told when you test HIV positive you should not go around sleeping with people. You will have sex with someone who has his own HIV and we will be adding virus into the body.

KC: Alright, what about where it can be found, is it important? Where to find it, at the clinic, pharmacy, in the community, in the location, where you can get it or venue where PrEP is found, is it important? Is it something that is important that might affect the uptake of PrEP? [Silence] Is it important?

XXX: Yes.

KC: Alright, why is it important? Still on venue, is the distance important, like say it is 5 minutes, an hour, two hours? I have to board a bus, is it important in the uptake of PrEP, 45?

45: Like the… these PrEP, collecting them from the community?

KC: Uhh.

45: From the people in the community, if one from the community sees me collecting PrEP, what will she be saying, what do you want to use it for?

KC: Alright, okay 42.

42: I want to help this one, who, I don't know its 43.

KC: 45.

42: Even if it is in the community, it will not work, it can be placed somewhere like at Machipisa there. There is someone who is not able to walk to go to Machipisa on foot.

KC: Alright.

42: So, it will depend on the availability of transport money to go and collect PrEP or that someone doesn't have.

KC: Or one doesn't have. Alright, 46.

46: Just helping these two.

KC: Uhh.

46: They must have places that are like a clinic where PrEP has to be taken, like I said that some days you come to this clinic they close even at half past 3.

KC: Uhh.

46: And you will start from half past 3 going to what, there. Those who would not be able…those who will be at work, those who are not able to make time for what…being able to be there on time for what…

KC: Time for coming.

46: At the clinic, that's why the clinic is there, because if it was that everyone go there for treatment, go there for treatment, there was no need for us coming to this clinic.

KC: Alright.

46: Of which some people in the community will not be aware of how to use PrEP.

KC: Alright, at the clinic. What about a place like say for example like here where there is PrEP services, STI testing, pregnant testing, such places would they be…would they be good for PrEP distribution, yet they have other services available besides PrEP. Will it work out or you just want a place where there is PrEP only, 46?

46: We don't want a place that only gives PrEP.

KC: Uhh.

46: On giving PrEP, let there be family planning services, such that when one gets PrEP if she wants a Depo injection or jadelle they can get it.

KC: Uhh.

46: If they give condoms, we will get them but it will be a place for… for adolescent girls and young women.

KC: Alright. Looking at family planning, right, especially current set-up, do you think it is easy for adolescent girls and young women to just get in and get family planning? Is it easy to get family planning methods, is it easy? 40.

40: It is not easy, [Baby crying loudly].

KC: Why?

40: Because if you come here at the clinic in need of family planning, there is need for money for stamping the consultation book [Baby still crying].

KC: Alright.

40: Then they will write for you the type of family planning, it has to be paid.

KC: It is not for free.

40: Uhmm, uhmm.

KC: 45.

45: I see it easy because you are told the day to come. There is a car that comes and give jadelle…

KC: For free?

45: Yes, for free.

KC: Uhh. 42.

42: It is easy because these days family planning people they move around in streets and people will be coming to…encouraging people to get jadelle, or tablets or to get loop.

KC: Alright.

KC: Alright, it is okay. Aah, in the discussion that we have done so far there is an issue that is coming out that PrEP or family planning for adolescent girls and young women it is difficult to get because at the clinics and hospitals the workers are not friendly towards young girls. What do you think about that, they are not being treated well, they are judged that you have done this, why do you need it. Why do you need it, you have done this, what do you think about that? 45

45: Uhmm, that needs to be corrected because us young women…good things, we want to be treated well because it is not a crime even that I have a child when I'm still young I also want family planning. Or that I do not have a baby, but I want to protect myself.

KC: Alright, but is it happening, is it happening?

Some: Uhh.

45: It's happening.

KC: Alright, okay so my question is what will they be saying for this treatment? Is it because young girls are indulging into sex, or they are practising unprotected sex? Where is the issue, 46?

46: Sometimes I would have gone to the clinic, maybe I would have engaged in unprotected sex.

KC: Uhh.

46: I would have engaged in protected sex then I would go maybe at the clinic there.

KC: Uhh.

46: I have an STI and I want PrEP. Sometimes maybe I would be 15years or 16 years, they may call each other the nurses three or four. Come and see what is here.

XXX: Come and see this young child.

46: Come and see the genitals of this child.

KC: Oh.

46: They would start laughing, sometimes they would start scolding you.

KC: Alright, okay I got that. We are about to finish, right. I just want to ask that looking at PrEP uptake by young girls, do you think male partners, could it be boyfriends or su…sugar daddy. The partners you… they will have at that time do they have an influence on one taking PrEP or not? These males, these male partners if someone is in a relationship, do they have an influence on PrEP uptake for the young girls? 45.

45: Haa, on males, few are the ones without influence.

KC: Uhh.

45: But some of them do not want but if you say you can get your PrEP with your partner, both of you taking it,

KC: Alright, if you agree but most people refuse. Others, what do you think about male partners, do they have influence? [Silence] Alright, aah moving on to our next question right, there is… when we discussed at the beginning, we said this is our first part of our research. There is another one that we will do in future, there will be more young girls that we will be working with, right.

XXX: Uhh.

KC: So, we will be looking at places that they can visit freely coming to the study that we will be doing at that time, we may be staying for 2 to 3 days at a place working with them, right. So, I would like to ask from you, where can we go and stay or if we camp there, young girls will be able to come freely. If you can suggest places, it could a clinic, schools, it could be places that are well known in the community that at this place they will come freely without being afraid of being seen, 46.

46: Here.

KC: No, not that they will be sleeping there. Just coming in the way, you did they would come and participate in the study like that's it and they will go back to their homes just like that, 46.

46: I think this place here is safe.

KC: This place here is safe.

46: Uhh.

KC: It's safe in terms of what?

46: In that right now if would have been at…like that place I mention down there at XXX.

KC: Uhh.

46: Just parking the car, sitting…we would be sitting down. Different people pass by that area, we have different behaviours, if it were others they could be saying "you are coming here to educate children of sex work, it's you who is spoiling the children". Someone would also pass by shouting whatever he/she wants and there will be a disturbance, and we will be listening to what the people will be saying, and we will not be listening to what you are saying.

KC: Uhh.

46: But here we do not have any disturbances because we will be in our own place.

KC: Alright, so, this place here is good in that there is privacy. Which other places can be good as well?

XXX: None.

KC: So, this side it's here?

XXX: Yes.

KC: Alright, what about a place like say at the shops. Where there are beerhalls, will that be appropriate?

[Participants strongly disagreeing]

46: That's what we are talking about.

KC: That's not appropriate.

46: That's not appropriate.

KC: Its out, alright. What about in schools at colleges is it okay?

[Participants mumbling]

KC: That we go to a school, and we get our place and pitch our tents and people will be coming to our study, 46?

46: Some students in those schools know us. At times our image will be tarnished badly because we come to collect condoms. So, if they see me at their school, they will start talking saying, I told you that she is a sex worker, right now she has come to collect PrEP and condoms.

KC: Alright, so, the places that I'm asking for, like say here in XXX just that here. Let's say for example, where else can I think of, let's say we are in Harare maybe in Mrehwa, Mutoko. Places like those ones like in rural areas where can we have a study where they can come freely.

XXX: Mutoko centre.

KC: Are growth points ideal venues? You once said that people will pass by saying a lot of things, 46.

XXX: Rural areas and here is different.

46: In rural areas we can find an open space just like that ground.

KC: Uhh.

46: And you would do like you are calling for a meeting.

KC: Uhh.

46: People will gather there.

KC: Uhh.

46: At the shops people have different behaviours, you might hear it being said in a different way.

KC: Alright.

XXX: Or a road show.

XXX: No one knows us there.

46: Even a road show.

KC: It is okay, what would motivate… young girls to participate in the study? Something that when you say haa, if you give them this or if you put this they will come, 38?

[Knock on the door]

38: If they hear that there is a $5, they will come.

KC: Alright, so an incentive for $5, Uhh, 47.

47: Sometimes if you go to places like in rural areas, right.

KC: Uhh.

47: If you bring pads, some people are not able to buy them, so if you give them pads they can come in their numbers those young girls.

KC: Alright.

47: Especially in rural areas, haa there have a problem with pads they use cloths but then it causes bruises.

KC: Alright, so if they are given … sanitary wear.

47: Yes.

KC: Okay, is there anything that we can…that will motivate them to come to the study? 46

46: On the issue of money, they need money because one will be calculating that I have left my place maybe I did not do my work. She would have left other things that she would have left saying let me go for this study.

KC: Alright, it's okay, 45.

XXX: "_+".

45: It would have been long without having chicken inn

[Others agreeing, laughing as well]

45: So, chicken inn would be good for them.

KC: Alright, so if they can get some eats like chicken inn.

45: These biscuits like those ones being eaten by that baby and maheu (maize-based drink), haa it is far from them.

KC: Alright, here we are not only focusing… because we are doing it in both rural and urban. We are not only focusing on, even the urban ones. What you are saying does it also apply to urban areas?

46: In urban areas there is need for cash, in rural areas you give pads and food.

[Others giggling]

KC: [Laughing] Alright, so on the study that we are talking about, right there is another component that we will ask those who will be interested, those who want to take part, right. We would ask them to take their own vaginal samples, vaginal samples, it would be tested for STI, right.

XXX: Uhh.

KC: To those who would be interested, do you think young girls would be comfortable, just being shown how to take the samples and then she will go to a tent and collect the sample. Would they be comfortable taking their own vaginal samples? 38

38: They… they would be interested.

KC: They will be interested, why, comparing to the sample being taken by a nurse?

38: Haa, not with a nurse.

KC: Why?

38: The nurse will ask a lot scrutinizing you, a lot so doing it alone, and it would be tested when you are not there, I don't think that would be a problem.

KC: Alright, it is okay, so, they…eh.

38: Plus, being looked at your private parts can… one can be sued these days.

KC: Okay, if it is legal, it would be a qualified nurse.

38: Oh.

KC: She would be doing her work it won't be illegal, 41.

41: Some are shy to be looked at by someone so if they do it by themselves it would be better because you can't be shy to yourself, you will be alone.

KC: Aah, alright. It is okay, 45 then we go to…

45: Maybe when the nurse will be collecting the sample, doing it yourself you will not hurt yourself but then someone might be afraid of pain.

KC: Alright, uhh.

XXX: Being looked at my private parts because I would have…an STI

KC: Uhh.

XXX: In need of treatment, wanting to see how it is like, maybe that's when I would agree.

KC: But if it is just colleting a sample, I would want to do it myself.

XXX: I would want to do it myself.

KC: Alright, aah, we are now moving on to the second from last question. On these questions those who haven't said anything I will point you one, one and you just give me one point, one point, before you leave, right.

Some: Uhh.

KC: Right, so, still on that the…the samples that would have been collected will be tested for STI, anyone who would want to see her results.

XXX: Uhh.

KC: Right, will get her results but anyone who would not want to see the results will not get them especially the negative results. So, my question is on the issue of results. How do you think we can give the results, let's say one has tested STI positive, and she would have indicated that when the results come out inform me, I would want to see them? How will we share them, how can we give them these results, I will start with 37?

37: You just take the individual and share the results privately.

KC: Alright, so we go back where we would have found them and give them the results?

Some: Uhh.

KC: What's your number?

39: You take her…

KC: Number?

39: 39.

KC: 39.

39: You take the person aside then…do the counselling first and then give her the results. When she sees them, you counsel her again and she will know that's the outcome.

KC: Alright.

39: You ask her how she wants to be treated.

KC: Number, 40.

40: It may happen that when you test me, I test HIV positive.

KC: Uhh.

40: You take me and say XXX my daughter… [Others laughing].

KC: Haa, it's okay, proceed.

40: Alright, you can take me and sit me down…and I will sit down.

KC: Uhh.

40: Because it is difficult to accept that I tested HIV positive.

KC: No, an STI.

40: Ohh.

KC: The results will be other STIs we are not testing for HIV, we are testing for STI.

40: Alright.

KC: Uhh. 45 and 46.

45: You take me to somewhere private, give me counselling and the give me my results. Then you tell me the way forward on how to get treated, the…STIs.

KC: Alright okay, so, the point you raised that how to get treatment, where could be comfortable for young girls to go and get treatment for STIs?

37: I will go to CeSHHAR.

KC: At CeSHHAR?

37: Uhh.

KC: So, we will refer to CeSHHAR and they will get treatment there, anywhere else, 38?

38: Population service.

KC: Population services, do they charge for the services or its for free?

XXX: It's free.

38: There are people who write vouchers, and you will get the services for free.

KC: Who writes the vouchers?

38: KATSWE, they give you a voucher.

KC: Oh alright, then you will go with the voucher there.

XXX: Without paying any money.

38: Uhh.

KC: Okay…

38: The day you get your results you will get the voucher on your phone.

KC: 46:

KC: Alright, it's okay. Aah lastly, we said there are other types of PrEP that are available, injectables, vaginal ring, right, not tablets. Looking at these what do you think?

XXX: We don't know them yet.

KC: You don't know them yet; you haven't seen them yet but you just hear about them and the way they work. Can you…will they be liked by young girls?

46: I wanted to ask that on the ring, yes, I have gone to get a ring and I'm getting it every month, doesn't it have any side effects that suddenly, I will be told you have cancer. You have cancer of the womb; you have this and that.

KC: Alright, so, so far like I said before that it is not yet being used, right, so, they are countries that are still…that are starting to use who are doing investigations to see the side effects associated with it. The side effects that we know of are few, there are for oral, for tablets right. These other ones still need to be investigated on the side effects, but just looking at them how do you see them, 38?

38: Yes, they can be accepted but the one that will be most accepted is the injection.

KC: Uhh.

38: Because it is easy, you just get injected and its done. The ring haa, people are afraid. People are afraid of using a female condom. You said the ring is almost the same.

KC: Uhh.

38: So, one will just think that it's the same, they will not accept. An injection maybe accepted.

46: I wanted to ask that the PrEP itself, the tablet itself. I was asking before we finish you help us; doesn't it have any side effects that will affect us in the future if you continue taking it?

KC: Alright, so on PrEP, oral PrEP right, 90% doesn't have the side effects. Only 10% can be the side effects and the side effects mostly they affect you when you are still starting to use it. These are the well-known side effects that you can have a headache, nausea, feeling like vomiting. Those are the side effects known which can clear, as you continue using it will end.

46: What about the issue that it will destroy your gall bladder.

KC: Those ones I don't know them. Those ones are the ones that I know the minor side effects which are not life threatening because if they were life threatening true, they would not have approved it in Zimbabwe to be used. They will not approve something that is dangerous for people to use it.

46: And the other area that I want to ask about.

KC: Uhh.

46: I'm taking PrEP right; I have said that maybe I would be taking it at 6 0'clock in the evening.

KC: Uhh.

46: I have taken it on 6 o'clock, maybe I have a month taking it consistently.

KC: Uhh.

46: Then at one time I forgot that I have to take PrEP at 6 o'clock and then I take it at seven o'clock. And then at one other time I skip one day.

KC: Uhh.

46: Then maybe I take it, I want to ask that doesn't that have any side effects?

KC: Alright, so, aah just like all tablets that if you had…skipping that you have forgotten or what?

46: I would have forgotten.

KC: It is said that when you forgot say it as soon as you remember and continue. You will not say yesterday I forgot and then you take both tablets aah, aah. You just continue from where you remembered you will just start and continue adherence would be good knowing that if you are taking at 6, its 6 if you set an alarm or what that will help you.

46: Still on that one.

KC: Uhh.

46: I have taken my tablet, I …today I have skipped then I met my partner, and we have unprotected sex, do I… do I have a chance of getting infected with HIV. All the other time I was taking it but the day I skipped I had sex and then I will continue.

KC: On that one, I'm not so sure but I think you will still have the protection. Let's say 7 days before you had sex you have been taking your medication, and you have missed now, and the protection was there and you are continuing so chances are high that you are still protected. We can get more information from, right, you said at the clinic they give PrEP.

46: Uhh.

KC: So, they can also clarify that part that I'm skipping… I have been taking for 7 days and I forgot on the eighth day then I had sex with someone will I be infected with HIV. So that they can just clarify that for us, right.

46: What if you want to ask another question that is not related to PrEP? Related to condoms, both female and male and these male condoms.

KC: Alright, we will…

46: Can I ask?

KC: We will ask after the discussion.

46: Alright.

KC: Alright, is there anything else to talk about on injections and ring? [Silence]. Alright, if there is nothing aah discussion…I have no other questions. Is there anyone with something to say, is there anyone with something to add, our recorder…Is there anyone with something to add on to related to our discussion?

KC: No one?

XXX: Uhh.

KC: Alright, it's okay, if there is no one thank you for your time, thank you for your views. We want to come up with this program for young girls, coming up the programs for PrEP that will make it easier to get, being acceptable and people to continue using PrEP, especially young girls and young women. So, thank you very much for your time, our discussion ends here.

The End.
