# Supplementary material for: Perceptions of pre-exposure prophylaxis among sexually active adolescent girls and young women in Zimbabwe–A qualitative study
Source: PLOS Glob Public Health. 2025 Dec 2;5(12):e0005396. doi: 10.1371/journal.pgph.0005396 (PMC12671731; doi:10.1371/journal.pgph.0005396)
Supplement: S1 File — (ZIP) [file pgph.0005396.s003.zip › S1_File/AGYW-FGD 06-Translation.pdf]

AGYW-FGD 06- Translation
Facilitator: KC
Note Taker: LM
Date Of FGD: 04/04/2022
Age group: 20-24 years
Translation by: SM

KC: Right. So, we are about to start our discussion, right. So, I would like to thank you once again for…your time that you have taken for us to have this discussion. Like I said before, my name is Kudzai, with me is Lindiwe, we came from CeSHHAR Zimbabwe. So today we would like to discuss about PrEP, oral PrEP which is Pre-exposure prophylaxis, so that we understand more on acceptability of PrEP, reasons that are likely to explain why there are smaller numbers of adolescent girls and young women that are taking on PrEP in our country Zimbabwe. And also, how we can have the number increased, right. So, our discussion, just I like said earlier [baby coughs] will take a time that is between one and half to two hours, however we don't anticipate getting to 2 hours, we anticipate being done then. So, just like we said as we were starting, that we are going to have role plays and follow-up discussions and so forth just so as to have an understanding of PrEP, how it is dealt with in our daily lives, isn't.We have already discussed and set our ground rules, so do not forget also, not to say out your names but to use the numbers we have allocated you, the numbers are the names that we are using in this discussion, isn't. So, before we start, is there anyone with a question or anyone with anything to ask?

[Silence]

KC: No one? 55

55: This programme, especially for us young women, we have liked it.

KC: Mmh.

55: Then we get home, our husbands forbid us, maybe it won't end today.

KC: Mmh.

55: We may be asked of on a certain day, when you seek for approval to go, you may be denied, what should you do then?

KC: Alright. So these issues to do with oral PrEP, right, are some of the issues that we want to hear more about in the discussion, to understand what causes other people not to able to refuse, not to be able to use PrEP, just like what you are saying now, we want to understand the reasons and see how best it can be dealt with. So, the solutions are

coming from you, how best the issues of PrEP can be addressed for it's a programme in its infancy in Zimbabwe, started around 2016, so its still being perfected, verifying its users, as well as noting areas of improvement, isn't it.

So, as we start, right, my first question is that have you ever heard about PrEP, what is really PrEP, what have you heard and what do you know about PrEP? 48

48: PrEP is a pill.

KC: Uhm, you may respond whilst you are seated.

48: It's a pill that is used for prevention of HIV

KC: Would you know under what circumstances is the pill taken? What time it is taken, for how long is it taken in a person's life?

48: You may be given, perhaps to start with a month's supply.

KC: Mmh

48: You remain subject to regular testing and then be given perhaps a 3-month pack of the pills.

KC: Okay.

48: Then you collect every 3 months.

KC: Alright. Okay. Mmh, anyone, what have you heard about PrEP, what do you know? yes number 49.

49: PrEP is a pill used to prevent contracting HIV and AIDS.

KC: Mmh.

49: The pill is taken in the same manner as the HIV pill. If it is taken at 8, it will be taken at 8.

KC: Mmh.

49: The pill is taken by someone who is HIV negative but who would want to use it as a method of preventing contracting…

KC&49: HIV.

KC: Alright. Thank you. In this group we have here, how many know about PrEP? How many have heard about PrEP even if they have not used it, but have just heard that there is something called PrEP? If you may raise your hands so that we can see you, 1, 2, 3, 4 [counting]. Alright. The rest of you, you have never heard of PrEP?

Some: Uh, "_".

KC: Is it your first time to hear about PrEP? Alright. Its okay. Right, so those who have heard about PrEP, the information on PrEP that you have been sharing with us where did you get the information from, how did you get the information on PrEP? 49

49: We heard of PrEP when we came to the hospital, especially those who are married.

KC: Mmh.

49: That there is a pill called PrEP, it is not taken by someone who have AIDS, but by someone with the intention to prevent contracting HIV.

KC: Alright, alright. At this hospital?

49: Not at this hospital but at Howard.

KC: At Howard. Alright. How about others, where did you hear about PrEP? 48

48: I heard of it here at XXX Clinic.

KC: Alright.

48: About the PrEP pill.

Kc: What kind of platform was PrEP being talked of or what kind of audience was being told of PrEP?

48: Those who would have come to…for weighing.

KC: Regular weighing of babies?

48: Weighing and check-up of pregnant women.

KC: Regular weighing and check-up of pregnant women. Alright. Its okay. [baby sounds]. So, for those who have spoken, right, you said…, [baby coughs], they said that the pill is for prevention right, is there any other type of PrEP that you know? Even those that are not yet available, but you have heard that there is this type of PrEP coming. Is there any other kind of PrEP that you have heard of? 48

48: Have heard of the ring type, that is inserted into the uterus.

KC: Mmh.

48: Have heard also of the other type, the injection type.

KC: The injection type, that's absolutely right. So, she said of the ring type, the injection. So, there are 3 type of PrEP that are available but in Zimbabwe what we are currently using is the pill, the ring has been approved for use but its not readily available. Then the injection type in Zimbabwe is not yet being used and has not been approved for use as yet but there are 3 types, right. So let me show you the types of PrEP, we have 2 here, the pill and the ring, [background conversation]. So, we have the pill and the ring right, so the pills are put in this container, in this box, so I want to see what the PrEP pills look like, [background conversation].

XX: Let me open it for you. [Background noise].

KC: Thank you. Alright. [Background conversation]. Right, so this is how it is like, this is the oral PrEP that is currently being used in Zimbabwe.

Some: Mmh.

KC: Here is the ring that has been mentioned, right. Ring, in our country is not yet available, like I said earlier [background noise], but its already made. So, the ring, just like the explanation given earlier, it is inserted, [background noise], it is inserted by a nurse, [background noise], so you can circulate it around, touching and having a feel of it, [background laughs]

Circulate it, touch it and have a good feel of it, right. So, it is inserted in the vagina, inserted by a nurse, it stays in the vagina for a month having it in your system. Give it

to others…would want each and everyone of you to touch it [Chuckles] You stay with it for a month, after the month you go back and it is removed and inserted again, so this ring, right, have some medication within it, that it releases into your body, in your system, so this medication is the one that actually protects you from contracting the HIV virus, right. But all the research that has been done so far, in Zimbabwe, in other countries on the use of the ring as far as contracting HIV is concerned compared with the pill, the ring is less effective than the pill.

Some: Mmh.

KC: The pill has a greater percentage, then the injection has also a great percentage of preventing the contraction of HIV virus, right.

Some: Mmh.

KC: So, I just wanted you to see and know the kinds of PrEP that are available as we discuss you would have an idea of what we will be really talking of, right.

Some: Mmh.

KC: Right.

XX: Does it expire?

KC: After 1 month it is removed, and you get a new one.

XX: Is it?

KC: So, this one lasts for a month in the system, whilst the injection lasts for 2 months in the system then you go back for another jab that lasts for 2 months. The oral PrEP, just like it has been mentioned, oral PrEP is taken at those times one feels has he/she has greater chances of contracting HIV, so you may start a week before, then you continue at that time when you are exposed, at that time when you feel exposed to HIV. So, the oral PrEP can be used quite easily in the sense that you decide when to take it and when to stop it, its not a pill that you have to take in everyday of your life till death, right.

Some: Mmh.

KC: So, you take the pills for a while and then stop during times when you feel you are not at risk, then you resume as you wish, you may go for a refill at the hospital, right. Right, so, now we want to… we now want to start our group discussions that we mentioned earlier right. So, these groups have three scenarios. These scenarios are like this, our first scenario…so, we are to get into 3 different groups.

Scenario 1

So, the first group is made up of Chido and Koko. So, Chido and Koko are 2 friends, best of friends [background discussions]. Right, so you will be 4 in your group but only 2 of you will act out the role play, right. So, our first group is made up of Chido and Koko, Chido is 16 years old. Chido is having a sexual relationship with an elderly man in his 50s, right. Chido thinks of starting to take PrEP as she thinks she is exposed to the risk of contracting HIV because of the intimate relationship she is having with this elderly man. So she thinks of Koko, her friend. Koko is 19 years old and is also at risk of contracting HIV [coughs] because of a sexual relationship she is engaging in. So, she tells Chido, rather Chido tells Koko about PrEP, encouraging her to take it up because of several reasons. So, this first role play I want it to happen between Chido and Koko. Chido telling Koko abou PrEP, we hear Koko's response as it happens in real life in such instances where someone approaches you be it your friend telling you about PrEP, how would you respond, right.

Scenario 2

KC: So, the second scenario is of two young women, Mai Bhobhi and Mai Juru, just like the young women in this group we have here today, right. Mai Bhobhi is 23 years old and married, Mai Juru is 21 years old and also married. Mai Bhobhi's husband is having extra marital girlfriends, plenty of them. So, Mai Bhobhi is stressed she might contract HIV because of her husband's unbecoming behaviour. So, she heard about PrEP on the radio and immediately decided to use PrEP, it is now 6 months whilts she is using PrEP, but she now wants to stop using PrEP, so our role play is between Mai Bhobhi and Mai Juru right, we want to hear a conversation between the two, Mai Bhobhi explaining why she now wants to quit using PrEP. So, here we want you to think

what really happens in real life in someone's life what could cause a person who has used PrEP for 6 months suddenly thinks of stopping using PrEP, provide us the most probable reasons in the role play why Mai Bhobhi no longer wants to use PrEP, right.

Scenario 3

KC: Then the last one is about 3 friends who are still in college all in their 20s. The friends' names are Pepper, Sky and Princess right. The three all have sexual partners. They all learn at the same college. They have been selected at their college to design and come up with a programme about PrEP, such a programme should be making adolescent girls and young women want to take up PrEP and use it continuously and willingly. So, in this role play, we want you to list, what is likely needed in a PrEP programme for it to be liked by adolescent girls and young women. It must have easy steps like ABCD, I can say for example, if we are to erect a school for the ECD infants, what kind of things should be put in the… [cell phone ringing] …school such that it attracts and makes the infants want to come to school right. Having an understanding that if we put books with colourful pictures, stick pictures on the walls, making sure that those that require lunch are provided the lunch, creating a playground where they can play with swings and so-forth, then definitely the infants will come. So, that is what we want to do in that programme. You just list the steps of how the PrEP may be liked by the adolescent girls, what should be contained therein, the kind of places where they can collect the PrEP, who distributes the PrEP and what are their qualifications, so, these are our 3 role plays. So, like we said before, you are gathering into groups, the way you are seated, and you discuss. So, for now we are pausing for 3 minutes, and we discuss in our groups, after group discussions, we round up and continue with our overall discussion isn't it.

KC: Right, so, we now want to start with Chido and Koko's group. You may remain seated where you are seated but just raise your voices high so that the recorder can pick it up. Chido and Koko, you may start.

XX: Umh, in this instance Chido is having sexual relations with a man in his 50s right

KC: Yes

XX: What we see hear is that Chido should first do…she is supposed to...[interjection]

KC: Alright. What I want you to do here, you are acting out a play.

XX: Alright.

KC: Chido and Koko greeting each other, hi Chido, hi Koko, what what [background laughter], then you act out the play, outlining the points you discussed and wrote, so you just agree who is Chido and who is Koko, and you act it out.

[Background discussions]

Chido: Hi Koko.

Koko: Hi, whats your name again?

Chido: Chido

KC: Let us all listen as Chido and Koko act out the play.

[Inaudible discussions &Background laughter]

KC: Its okay, you may start again.

[Background laughter]

KC: Start over again because you are acting it out. You approach Koko, greeting her and start a conversation and we can hear you out

XX: Wait, we may need to discuss a little bit because we were just writing down our points.

KC: Alright. We ought to start with Chido and Koko. That's the order, we start with Chido and Koko because if we start with other scenarios, the flow is distorted, the scenarios are linked from Chido going to Mrs Bhobhi and then the third one, so they are somehow linked till the last one.

[Inaudible background discussions]

KC: Raise your voices

[Laughter and inaudible background discussions]

XX: So, we were saying "_"

KC: Mmh, act out what you discussed and wrote down, that's what we want to hear, how Chido converses with Koko, telling her this and that and Koko responding to the conversation.

Chido: Okay. Hi Koko, my friend

Koko: How are you Chido?

Chido: Okay. I have a story to tell you. I have a man I am having sexual relations with, his age…

KC: Others listen carefully.

Koko: Mmh.

Chido: Umh, I am being intimate with him without protection, how do you see it?

Koko: What? Chido my friend that's not ideal, you must protect yourself first before you become intimate with him.

Chido: Protecting myself, how…

Koko: You just go to the hospital.

Chido: Then what do I do there?

Koko: You get tested for HIV?

Chido: If I get tested, then what do I use?

Koko: If you get tested you may be given pills to prevent contracting HIV if you are positive, [background whispers], if you are negative rather, if you are positive, you may be given medication to protect… [background discussions]

XX: If you are negative

[Background discussions]

Chido: Alright my friend, thank you so much for sharing such an idea, let me visit the hospital, thank you once again.

KC: Alright. Thank you Chido and Koko, right. Right, so we now want to have a discussion about the role play of Chido and Koko. So, here, we want to discuss in detail, outlining even those details that didn't come up in the play, [background discussion]. So, we want to have a short discussion of the details that were raised in the play. So, firstly I want you to tell me if these kinds of adolescent girls exist even young women like Chido who have sexual relations with men way older than them, does this exist?

Some: Yes.

KC: Right. So, what I want you to do, those who would have said yes, will have to explain to me what will be really happening. Why would they be engaging in such sexual relationships, what exactly will be happening, how does it work in such relationships?

53: Some would have been lured by money, being told that I will give you money and her wanting the money she will give i. Or it maybe a case such that at her homestead there is abject poverty which leads her to fall for way older men.

KC: Alright. It is said to be because of the love for money or probably because of poverty. 57.

57: The other way I see it, [baby coughs] the two would have agreed to marry each and therefore engaging in unprotected sex.

KC: Alright.

57: "_".

KC: Alright. Mmh, what number are you?

54: 54.

KC: Ooh it is 54

54: It may also happen because of rape.

KC: Because of what?

54: Child abuse.

KC: What kind of child abuse would be this?

54: Being raped.

KC: By relatives or strangers or what?

54: By close relatives

KC: Okay. Mmh, how about others? What causes young adolescents into these relationships, is it because of what has been said about… yes 49

49: Chido might be forced into sexual relationships with older men probably because of the way she would be living at their home.

KC: Mmh.

49: So, she may just force herself believing its better to live in with an older man, thinking she would live a better life.

KC: Alright. Okay. Circumstances, situations, yes, anyone else with a contribution, yes 55, what is it?

55: 55.

KC: Mmh.

55: At times the child will be having the grandmother as the legal guardian.

KC: Mmh.

55: The grandmother would be very old

KC: Mmh.

55: The child starts being mischievous, until she gets into relationships with older men, her grandmother would be ... grandmother would be struggling to make ends meet, having no food.

KC: Mmh.

55: The grandmother will not be able… [Baby sounds] to discipline her grandchild, telling her that what she would be doing is morally wrong. Nowadays there are various sicknesses, what the grandmother would want is to eat whatever food that would have what…

KC: Would have come

55: Would have come, that's the greatest problem.

KC: Is there anyone else with anything to add?

[Silence]

KC: Alright. Would you think a girl like Chido because of her sexual relationship with older man, is she at a risk of contracting HIV, does Chido have a risk of contracting HIV?

A few: Yes

KC: Where does the risk come from? How is she at risk of contracting HIV, where exactly does the HIV infect her?

59: The major issue is that we do not know how this man behaved in his past life.

KC: Mmh.

59: So, that is the source of the risk

KC: That's where all the risk is. Mmh, how about others, what risk does Chido have? 54

54: We wouldn't know if they were tested for HIV or not or they would have just…

KC: Alright. Yes, 49

49: Chido may be engaging in sexual activities with an older man but being a person who is doing it for a benefit, the thought of being tested for HIV first does not come to her mind.

KC: Alright. In these kinds of relationships, would they use condoms? [baby coughs]

XX: Uh.

KC: These kind of relationships between young adolescent girls and older man, is there any use of condoms?

[Inaudible background discussions]

KC: Why are they not used? 40

40: Because of the money that you be given [chuckles]

KC: Yes.

54 He wouldn't be wanting to use a condom [chuckles], would be saying I need value for my money

XX: Need value for my money.

KCV: Alright.

XX: Most usually say, have you ever eaten a sweet in its wrapper? [background laughs]

KC: Alright. So, it has been said that if a man is giving you his money, he doesn't want to use a condom.

XX: Yes.

KC: He does want also to use a condom, noting that he does not want protection.

XX: Simply because he is giving you money.

KC: I am giving you something else.

XX: Plus, some of these men would actually know that they are HIV positive what they would want is just to have unprotected sex with you. If you contract HIV, you contract it, if it is an STI, then you just contract it also.

KC: Then you contract it.

XX: Yes

KC: Why is it like that? Considering the issue raised by…40, what is it?

48: 48.

KC: It was 48 isn't it [baby sounds]. It was once talked of in the discussions we have had before, it came out, this issue of some men who will be on an HIV spreading spree just because they are HIV positive. What would you think are the major reasons why men do this, they knowingly spread HIV, knowing that they have HIV, but they keep engaging in unprotected sex, vowing that they will spread it? 56

56: Most of the men "_"+.

KC: Alright. Okay. Mmh, is there anyone with anything to add? 54

54: One would want to increase the number of those taking ARVs.

KC: So that you are many who are taking anti retro-virals. Alright. Its okay, umh, how about when we are looking at adolescent girls and young women, are they people who can share with other people their health-related issues, here we are looking at issues like going and getting tested for HIV or when diagnosed with an STI would they seek medical help, are they able to talk to anyone that I have this condition, is it possible with the ages 15 to 24, is it possible? 49

59: In most instance it won't happen because what I begin to think is that if I share with so and so, how would they take it. It may so happen that if I tell one, that one person tells the next person and the next, meaning that I will remain walking around whilst every person now knows my condition.

KC: Alright. So, they are afraid that there is no confidentiality with their issues.

59: Mmh.

KC: Alright. Mmh. Are there any reasons besides the reason that they fear that the issues are not kept in a confidential manner by the person they tell? Are there any other reasons that make them not to feel comfortable sharing their health-related issues?

[Silence]

KC: Alright. If we are to look at those who can share their health-related issues, to whom do they share the issues with. Are there a kind of people that adolescents and young women feel free to tell that I have done this and that? Is there a type of people that

adolescents and young women feel free to approach and tell them, they might be family members or any other person they may be comfortable with to share their issues?

[Silence and baby sounds]

KC: Mmh, 56, yes.

56: Personally, I would choose to tell a family member rather than telling a friend.

KC: Family mambers.

XX: You would rather tell your parent.

KC: So, your parent…

XX: Or "_".

KC: Or who?

52: Community health workers.

KC: Number 52 said community health workers at the hospital, so, you mean these are people you trust right?

Some: Mmh.

KC: So, we are saying family members, parents, community health workers. Are there any other class of people who can be trusted, are there any?

XX: One may even tell her grandmother.

KC: Even her grandmother. What is number is it?

50: 50.

KC: Number 50 said even her grandmother. Yes.

[Background discussions]

59: Even at church, to a pastor

KC: Even at church, churches are fine as well to pastors. Alright, how about best friend, will a best friend do?

[Inaudible background discussions]

55: When you are done talking to her, just after parting ways, she will be the first one to tell other people.

KC: Alright, so 55 said best friend will not do, is not able to keep secrets [chuckles], feels obliged to tell the story, right?

Some: Mmh.

KC: Alright. Umh, what if we are looking at, there was a time we asked when we started that the information that you were talking about PrEP, where PrEP is being found, right. Which other places do you think that if information is put in those places, adolescent girls and young women will be able to obtain information, get the information easily. Which are the places that you may think that adolescent girls and young women will get information on PrEP…That adolescent girls and young women will get information on PrEP? 53.

53: Even in hospitals or school.

KC: In hospitals and schools.

53: Mmh, you can travel to…

KC: Number 52, mmh.

52: Places like XXX where there are plenty of adolescent girls and young women who work there.

KC: XXX what?

52: XXX XXX.

KC: XXX XXX is it. Mmh, are there any other places that you think are suitable?

[Silence]

KC: Alright. So, we now want to discuss the role play, we want to understand in more detail, so we want to start with…as we look at the role play, Chido was telling Koko about her

story that she is being intimate with an older man and they are not using any protection. Koko responded by telling her to visit the hospital and get some help [baby sound], how did you see Koko's response pertaining Chido's issue? Is it what happens in real life, lets start from there, is it the kind of response given by Koko that people get if they tell people in real life situations?

[Silence]

KC: Koko was speaking, suggesting that her friend visits the hospital in order to get some help on how she can protect herself from contracting HIV, after Chido told her that she is having unprotected sex. So, the response is it the right one we expect, is it what generally happens in real life discussions?

KC: Mmh 55.

55: At times such a response is given.

KC: Mmh.

55: At times it may not come out as this, probably because the advice you may give is to tell someone please go and do this and that, but however she may respond to your advice by saying AIDS is no longer a cause for concern since there are now antiretroviral pills.

KC: Alright.

55: But personally, you would be giving her sound advice, but she responds otherwise.

KC: Otherwise. Alright. Anyone else with anything to add?

[Silence]

KC: Alright. When we look at some of the responses that may be given during such a discussion, right. So, our role play was aimed at discussing Chido's issue right, that Chido has started using PrEP, but Koko her friend, is not using PrEP but at the same time she is having sex with older men without any protection.

So, let's say we have presented the situation like that, in real life would a person be approached and told to use PrEP and this and that, what would be the person's response? When one is approached, what is likely to be their response?

49: You maybe asked, what have you seen on me that gives you the audacity to tell me to use PrEP?

KC: Alright. So, one may even ask or become harsh asking why are you telling me that, what have you seen on me?

59: You are telling that, are you done minding your own business?

KC: Alright. Mmh, any other responses that may be given in such an issue, when one is told about PrEP by her friend? 55

55: One may just agree but will not practice it.

KC: She will not go and take up the medication, just agreeing so that the conversation is over. How about others?

XX: One may "_"

KC: Alright. 55

55: I am not in that need.

KC: I am not in that need.

55: Yes

KC: Okay, mmh, is there anyone else? What are your views on the benefits of using PrEP amongst adolescent girls and young women. What are the benefits of using PrEP, that you may tell to encourage someone to up take PrEP, what can you talk of? [background coughs and baby sounds] 48

48: The benefits that I see are that even if you have a husband who has many girlfriends and comes back to you to have sex. When you are using PrEP, you won't be at a risk of contracting HIV.

KC: Alright. Mmh you may be protected even when you have a partner who is promiscuous with plenty of women, you will be protected. Mmh 52, Is that what you wanted to say? Alright, anyone else with anything to add that is different from what 48 said?

[Silence]

KC: When looking at what we call barriers or hindrances that may cause adolescent girls and young women fail to take up PrEP. What are these barriers or hindrances that you think because of them, one who has been told of PrEP will not take it up because of that? 59

59: I think just being shy, one feels shy seen going to the hospital, fearing that she becomes the talk of the town, she in now taking pills, she now has AIDS…

KC: Alright. So, being shy, being afraid that people will say she is taking pills.

59: Mmh.

KC: Alright. 49

49: At times it is lack of knowledge.

KC: Mmh.

49: Lack of knowledge.

KC: Alright.

49: Why it is important

KC: For them to use PrEP. 52.

52: Wanted to say that one may not embrace it.

KC: May not embrace using PrEP.

52: Mmh.

KC: Alright. Others, 48

48: Some people don't even know what PrEP is, what the pills are used for. What do they protect against, so someone will just say PrEP are pills just like those for HIV.

KC: Okay, so I do not want to use it for those reasons.

48: Yes.

KC: Alright. Is there anything else that we may see or think as barriers, that may cause one not to use PrEP?

[Silence]

KC: Alright.

XX: What if…[interjection]

KC: Mmh.54.

54: If one is using the pills and her husband gets infected with HIV and you remain using the pills, will they protect from contracting the HIV if you are living together as husband and wife [laughs].

KC: Yes, that's what we are saying that PrEP is a personal choice that you would have made that you want to use PrEP considering that you are in…you have a partner. You will now be telling them that this is what you are doing, I am now using these pills, they protect me from contracting HIV, even if you go to the hospital, they offer you…Isn't that they give counselling at the hospital, narrating to you that you may use these pills for you not to contract HIV. They may even counsel you as a couple, telling you that this one who is HIV negative may use these pills to remain negative.

54: Mmh.

KC: Mmh, what if we consider…now we want to consider PrEP progrmmes, right, or where PrEP medication can be accessed. Which are these places that you may think it will be possible, that it will be successful if PrEP is put in those areas, adolescent girls and young women would come and collect the PrEP willingly and comfortably go to those places without fear or being shy. Which places are these that you may think it will be

possible at that place. Which places do think will do because currently, right now where is PrEP being accessed? 52

52: In private clinics.

KC: Private clinic. Alright, is it sold or its for free?

52: Uhm, it is free, it is availed so that people get helped.

KC: Alright. Okay. How about others, where is PrEP being accessed? 48

48: It is being accessed at clinics such as this one, Tsungubvi, it is available.

KC: Alright…

48: But for you to get the pills you may have a husband that is HIV positive and yourself being negative, that's only when you can get the PrEP.

KC: Alright, if you are a discordant couple, that's…What if, what if you are just an adolescent girl who is sexually active who wants to protect herself, is she able to come here and have access to PrEP? Will I be able to get it?

48: Uhm, concerning that, I am not too sure

XX: Yes, you can.

KC: Are you able to get it?

XX: Yes.

KC: Uh. Alright. Its okay. So, we are now saying that the places you may think of, places that PrEP can easily be gotten, which are these places that you may suggest that if PrEP is put there, it will be easy to access PrEP because of this and that. Which other places that you may think of that will make it possible to avail PrEP? 49

49: I think a place like the country club.

KC: Mmh.

49: "_"+. People can easily go there because what usually happens is that one may be shy to go to the clinic thinking that I may meet people that I know. [Chuckles] If this other

person sees me there carrying the pack of PrEP pills, they wouldn't understand that it is PrEP, they may suspect that they are HIV pills.

KC: HIV pills, alright. So at the country club, what is done there?

49: There are other programs that are usually done at the club, there is a clinic, they usually invite young adults to get treated of any sexually transmitted infections.

KC: Alright. How about others, is there a place that you may suggest that PrEP be put so that people have access, which is not the country club?

49: Places where we have community health workers.

KC: In the community? Community health workers?

49: Yes.

KC: Huh, alright. What makes them the best? Are they the ones to be given the pills and they distribute within the community?

49: Yes, giving people.

KC: Okay. Will this work?

Some: Yes.

XX: A person may actually tell them their problem, and she gives you privately whilst no one notices.

KC: Alright. Its okay, anyone else who has anything to add?

[Silence]

KC: Okay. We have talked of places, right, what if…how about in a pharmacy, will it work to put PrEP there, so that adolescent girls and young women can access PrEP at the pharmacy?

[Background discussions on whether it would be offered for free]

XX: One won't have the money to pay for the pills.

Kc: Alright. So, the pharmacy will not do because they will definitely sell.

XX: Yes, they are always in business.

KC: What if a programme is designed, that PrEP is being offered at a pharmacy free of charge and not being sold, will it work if it is taken to the pharmacy?

Some: Yes, it will work.

XX: If we are going there to take it free of charge at the pharmacy, if it is then sold when its supposed to be free, we have all the rights to go to the police since it is supposed to be free, they will be now stealing.

KC: Alright. Okay. What makes a pharmacy ideal for collecting …PrEP at the pharmacy, [baby sound], what advantage does it have?

54: Its ideal since a lot of other stuff is sold in there, so no one will suspect that you want to buy something in there.

KC: Alright. So, there is secrecy and privacy.

XX: Yes, no one will know what you would want to buy.

KC: Huh, alright. Its okay. What if we are looking at the person distributing the PrEP, right, PrEP so far is being given by nurses in most cases. If it is a person at the pharmacy, what kind of a person would you want, you may describe the kind of person, describe the kind of person, their age and so forth? 49.

49: Personally, I think an elderly person is ideal, they may be able to counsel since there may come a husband and wife. One being HIV positive and the other being negative, those people may start quarrelling, so they need an elderly person capable of counselling them, saying no, you (negative person) you may live without the HIV virus.

KC: Mmh. Mmh. Alright. There is need of an elderly person who is capable of counselling people as they come with diverse problems, anything else?

[Silence]

KC: So, when we consider the frequency of taking PrEP, like we explained ealier, saying that the ring may be used for up to a month, then the injection also lasts for 2 months, the pills are taken almost every day, right, would you want PrEP that…Suppose you are asked which PrEP you want. So, considering the time frame, would you want PrEP such that you will be taking it every day, will you be taking it once in 6 months or I will be taking…that is what we want to understand. Which one would…which one would the adolescent girls and young women be interested in?

49: They like what lasts longer because they do not want what keeps them busy because its like the pills will be needed everyday but at least 2 months, if they hear its for 2 months…

KC: Alright. So, the one which takes quite a lengthy time?

49: Yes.

KC: Okay, alright. Uhm, so, we now want to have the second role play, that of mai Bhobhi and mai Juru. So mai Bhobhi and Mai Juru may you act out the second role play and we get to discuss it and so forth.

[Inaudible voices, baby making sounds]

KC: So, you will be just doing like what the other group did, a short drama/role play, mai Bhobho has a problem with her husband, she was using PrEP, so she now wants to stop using PrEP. So, we want now to hear the probable reasons that are causing one to stop using PrEP, having used the PrEP for a good six months.

[Baby sounds]

KC: Raise your voices so that the recording will be audible.

Mai Bhobhi: *How are you mai Juru?*

Mai Juru: *Im well and yourself?*

Mai Bhobhi: *I am okay, uhm, my dear things are not well at my house, my husband is being promiscuous, so I am thinking of taking pills, I was taking PrEP, but I am now thinking of discontinuing use, I don't know what to do my dear.*

Mai Juru: *So, you want to discontinue when you have been using it, is it that it was not effective?*

Mai Bhobhi: *It was effective but uh, I want to stop using it and enjoy my life like he is doing.*

Mai Juru: *Uh, that's not good my dear, just continue using it and be strong maybe he will change*

Mai Bhobhi: *Oh, thank you so much my dear, let me do as you said.*

KC: Thank you mai Bhobhi and Mai Juru.Right, we now want to have a discussion around this issue. Uhm, firstly do we have adolescent girls and young women who are in Mai Bhobhi's situation where their partners have multiple sexual relationships? Are there girls who encounter such situations, and what is it like for those that are in the situation? Just describing the situation, are you tired already? 55

55: There are people who experience it, it happens a lot, your husband will be having multiple relationships leaving you. So, at times you think revenge is the best solution, but I personally think revenge is not the best solution. You just… like now we have this pill, we are actually happy that if he is promiscuous, I will just take my pills.

KC: Alright. Anyone else, there are young women and girls who are in mai Bhobhi's situation. Alright, is it something easy for a married woman in mai Bhobhi's situation to decide to take PrEP, is it easy for young women in marriage to decide to take up PrEP? 53

53: If someone gets someone who tells them about PrEP, if they comprehend it well, they can embrace it and use it.

KC: Alright. Uhm, 49.

49: Its not easy because one can say, can I go and collect PrEP as if im going to the clinic to get tested, I will just wait till it happens.

KC: Alright.

49: When I am infected, and l start from there.

KC: Alright. Anyone else? Do you think they might need… there is a question that was asked in line with a question that I want to ask on whether young married women would need approval from their partner to use or not use PrEP? Do you think this can happen, and will the husband allow their wives to use PrEP? 49

49: But speaking for myself as a woman. I wish my husband would agree but its not easy, he will never agree.

KC: Why, why would men be reluctant to have their young woem taking up PrEP? What could be the reason?

49: He will be questioning why I decided to take up PrEP, what made you want to do that.

KC: Alright.

XX: From what I observed, it depends on the male partner, for those men who drink beer, you can't afford to tell them you just take your things in secret.

KC: So married young women… [Baby falls down and cries]. Alright, so you are saying there is a certain type of men who can be told and those that cannot be told.

XX: Yes.

KC: So those who drink beer, who get into beer halls are hard to tell, you just do your things in secret, what kind of men can be told?

XX: Because if those that drink beer get to know about it.

XX: Those who are church goers, who are religious can understand and accept.

KC: Religious people can understand.54 says religious people might understand and accept, but others are saying all men will never understand that someone is taking PrEP.49.

49: Huh, just a few, men will just not understand this, because its just like if you come back from the clinic after testing positive for HIV, and he is aware of his deeds. He will never acknowledge that he is the one who brought the disease, he will start questioning where you got it from.

KC: Alright.

49: So, huh…here, firstly what can happen someone can use PrEP for six months then later say I do not want to use it.

KC: Okay, he won't agree. Okay, looking at the issue that mai Bhobhi no longer wants to use PrEP after 6 months of using it. Mai Bhobhi said she has decided to just go with the flow and do what he is doing, are there any other reasons why someone who has been using PrEP, around women who have been using PrEP can just decide to discontinue use? Are there any reasons that were not revealed in this role play, that can cause someone to discontinue use of PrEP, any reasons, or is this practical. Firstly, is it practical for someone to use PrEP for 6 months and then decide to discontinue use.

55: One will be unsure of when to use PrEP.

KC: Alright.55, one wil be unsure.

55: They will be suspecting that they might be infected with the disease.

KC: Alright, 53.

53: They might have taken up PrEP whilst already sick without their knowledge.

KC: And they discontinue use of PrEP?

53: Yes.

KC: Anyone else, what other reasons can hinder uptake? Okay, what can be done to ensure that someone who is on PrEP never gets to a point like mai Bhobhi did of wanting to stop. For them to continue using it, what can be done, what suggestions can you give? That if you do this and that it will work, what can be done to ensure that adolescent girls and young women continue using PrEP? 55

55: They need comprehensive counselling where they are informed of the advantages and disadvantages.

KC: Alright. Counselling where there are educated on the advantages and disadvantages, alright, anyone with a different opinion on what to do to ensure that someone continues using PrEP, is there anything else?

XX: Counselling can also come from these papers we are using; counselling can also come from there.

KC: What kind of papers?

XX: Like the ones we are reading.

KC: Oh, for information to be printed on papers so that people can read?

XX: Yes.

KC: Mmh. Alright, anything else? Alright, its okay. No, we want to move on to the 3rd role play with 3 friends who are designing a PrEP intervention. We are asking them to list the attributes of a PrEP program that will be embraced by adolescent girls and young women so go-ahead group 3.

[Inaudible coversation]

XX: We have a challenge, we did not understand our task, you have explained to us, let the girls…

KC: What had you written?

[Laughter]

XX: We had different thoughts one what exactly we should do, whether we should do a debate like others or… One was of the opinion that a debate is required like what was done by those 2, whilst another one who was giving an example that just like the way you came that's … is that's what we are supposed to do on this question?

KC: Alright so give us what you had prepared, and we start from there.

XX: What we prepared?

KC: Give us what you had written down. Even if you provide a list of things of things that constitute the intervention you had designed, because for your group its okay of you do not go ahead and do the role play.

[Inaudible voices]

Peppa. Firstly, in my opinion uhm I will referring to a paper talking to Sky and Princess.

KC: Tell Sky and Princess.

Peppa: How are you Sky and Princess?

Sky and Princess: We are well.

Peppa: Sky, I have a sexual partner who is of age, I think if we encourage each other to take up PrEP, what do you think about it?

Sky: Huh, that's a good idea my friend, what do we do to access PrEP?

Peppa: We can go to the hospital, and we encourage adolescent girls and young women who can learn from us if we are on PrEP.

Princess: But will it be successful, is it easy, does it work well?

Peppa: Yes, doctors and experts were informing people that ... like myself I have a sexual partner who is of age, I don't know what he has done in the past and how his family life is like.

Princess: My friends, if its possible we should go and seek prevention whilst there is still time you know.

KC: Alright. Thank you, Sky, Peppa and Princess. So, we now want to discuss this role play right. So, first of all if girls and young women are assigned to design a PrEP intervention that can be embraced by adolescent girls, do you think they will be able? Can adolescent girls and yound women design a PrEP intervention which they will use to access PrEP, will it work?

Some participants: Yes, its possible.

KC: Its possible, alright. Thinking about an intervention, for it to be a success, what sort of things do you say should… should never be left out. What sort of things do you think should never be left out for it to be a success. Here I am talking about the PrEP intervention right, looking at the place from which its offered, the service providers who offer PrEP, the distance you travel to go and collect PrEP. The kind of people in

that area, the waiting hours you must endure before receiving PrEP. We are asking about the things you think should be part of the PrEP intervention. For a PrEP intervention to be ambraced by adolescent girls and yound women what should never be missing?

59: The pill, (…), the injection, everything is supposed to be available, such that if people… If girls and women get there, they won't be told that its unavailable come on another day, its unavailable.

KC: Alright. So, she said PrEP is not supposed to be in short supply, it should always be available. Anything else, is there anything else you think is essential for the PrEP intervention to be a success? Anything else? Nothing? 48.

48: You are not supposed to… let's say you collect monthly, then you fail to take it up because its not available.

KC: Alright.48 is supporting what 59 said that it should never be in short supply.

XX: The site from which PrEP services are offered should never be closed, because people are available at different time points. Some are available on Mondays whilst others are available on Tuesdays.

KC: Alright.

XX: So, it shoud never be closed.

KC: So, when you say that it should never be closed, what time are you referring to, 24 hours?

XX: Yes. For 24 hours.

KC: So, it should be a 24-hour program where one can go to any time they want to access PrEP.

XX: Some people might go to work and come later.

KC: Alright, anything else, what else do you think is essential on this PrEP project that would make it popular with adolescent girls and young women? Anything else,

anything else? Alright, looking at things currently, we asked where PrEP services are currently being offered, they are being offered here at the clinic. Is PrEP service provision going on well for those that know about PrEP, is it going on well or not, if not, what are the current challenges in PrEP service provision currently, lets start with the challenges ?49

49: What happens is that if people are told that, like at the moment it is available at clinics, they do not come because they know that there are… (+…)

KC: Alright.

49: So, someone may fail to come because of that, if there is a designated place from which the services is offered, especially a place that is meant for you women. I think someone would be free, it will be conveninent.

KC: Alright, alright. So, a place that is frequented by many people seeking different services does not work well for adolescent girls and young women.

XX: One may be shy to be seen, if you are HIV negative, you won't be shy…

[Inaudible background discussions, desk being pulled]

XX: We don't want that at the moment, later, one will end up loosing patience and decide to just leave it.

KC: I won't take it up anymore.

XX: Yes.

KC: The way you are treated when you get to a place where PrEP service is offered, anything else. Alright, uhm, looking at the places from which PrEP services are offered right, does it work or is it something that would be an advantage for adolescent girls and young women. Say a place has been set aside where PrEP services will be offered from, they will be accessing PrEP and family planning at the same time. If they want to access family planning services, they could also acess STI testing and treatment if they are willing, they can get tested for pregnancy also, if they are pregnant, they get a referral or get assistance. Does it help to have such a place like a one stop shop where there is

PrEP and other services, or PrEP services should be a stand-alone initiative where no other service is offered, what is your view of that 49?

49: I think it's a good idea, those who desire to access family planning and those who want to be treated for STIs, yeah, I think it can work well.

KC: Alright. Okay. Alright, why?

49: Because it is the young women who will be taking family planning.

KC: Oh, alright, okay.

49: Uhm, STIs also its primarily us young women, so I think…

KC: It works.52.

52: That is what I wanted to say that everyone can benefit.

KC: Alright, okay. She said that everyone can benefit from coming to this place. Alright, we briefly discussed this issue, the aspect of male partners involvement in uptake of PrEP. So just asking for the last time how male partners can be involved in uptake of PrEP services. We briefly discussed the issue of PrEP for adolescent girls and young women, but from our discussion married women like those that are here might encounter a challenge that their husbands might not allow them to take up PrEP. How can the husbands be involved in uptake of PrEP services. What kind of support can they give to their partners regarding uptake of PrEP services. We have been told to design a programme for couples who desire to take up PrEP, how can we involve them in uptake of PrEP?

49: I believe that when they come, especially for us expecting others, there is a time that they request that you both come, that is when such kind of counselling should be done.

KC: Alright.

49: Getting advice.

KC: Alright. So, you are saying if they are just educated.

49: Yes.

KC: Alright. Anything else? If there are just educated on what PrEP is, is there anything else? Is there need to ensure that when she takes PrEP for the first time he will be by her side, collecting PrEP whilst he sees the PrEP and everything is explained to him, is that support required by adolescent girls and young women? Or you just want them to have an appreciation of what PrEP is so that when he sees you taking PrEP he will not have many questions. I would like to understand the kind of support you require, only what 49 suggested or you want him to be available when you collect your PrEP fo r the first time?

XX: If he didn't understand, we should take him to the hospital for further explanation.

KC: If he didn't understand he then comes to the health centre for further explanation. Alright, anything else? Alright, we are close to the end. So, I would just like to ask about family planning services, is it easy for adolescent girls and young women to access family planning services? Be it at the clinic, a private facility or elsewhere, is it easy for them to just get there and request for family planning, is it easy to access family planning?

XX: Its not.

KC: Its not easy? Why, what are the challenges faced in accessing family planning?

XX: Its like at the clinic, if you come to the clinic, you might be told that its (family planning) unavailable. You proceed to the pharmacy, and you are told that it costs $3, yet you will not have the money.

KC: So, on the issue of non availability in hospitals and being sold in pharmacies, okay. So now we would like to ask, we have… on this study we are doing, we are conducting focus group discussions as part of the study. There is another study that we are going to conduct later on, the same study sorry, a survey that we will conduct. So, we would like to conduct this survey amongst 900 adolescent girls and young women. So, we will carry out the study in Harare, Mashonaland East and Mash Central and will be asking a few questions regarding PrEP, their sexual life and how they live in general. So, I would like to hear from you, I would like to get suggestions on how we can structure the survey. So firstly, we would like to ask about places were we can find adolescent

girls and young women. So, the way we will roll out the survey is such that we will actually visit a place say here at XXX, and we will be stationed here for about 5 days, and adolescent girls and young women will be coming to participate in the study. So where can we find adolescent girls and young women, which places can you recommend that adolescent girls and young women will be free to visit because we would want adolescent girls and young women engaging in sexual activities in or outside marriage. Which places can you suggest that you would be free to vist?49

49: I think places like the country club, because there is an initative that was once rolled out, I observed that even sex workers were free to go there.

KC: Alright.

49: They were just saying we are going to access free treatment for STIs and so on.

49: I think they would prefer a place that offers privacy.

49: Yes, because at that place nurses offering treatment would stay there for a week.

49: You would just go there; you access treatment and leave.

KC: And you leave, alright. So, sex workers… what of those that do not necessarily sell sex, will they be comfortable?

49: They were actually going, they can go.

49: Because they mentioned that services are offered for free. There is no need to queue and there are no concerns of lack of medication, everything was for free.

KC: Alright. Is there any other place you can suggest, she mentioned that the country club is private, is there any other place? Is a place like this one okay? Here at XXX. Will young girls and women who are sexually active and other people who are not married be comfortable coming here?

XX: In my opinion, the hospital is good. Its a good venue considering those people from rural areas who do not know where the country club is who just come straight to the clinic.

KC: They can come here for instance, what do others think, will this be a good venue?

XX: At the stadium grounds.

KC: At the stadium grounds? So, we just get there, and people set up tents, will people be free to visit such places?

XX: Yes.

KC: Alright. Its okay. What do you think we can do as the study staff for them to feel free to and be willing to participate in the study. What would you suggest that if you do this and that they will be free to come. What would you say if its included the study would be a success. Considering that our study seeks to recruit girls and young women who engage in sex? Our primary question is how it can be structured to ensure that they are free to come, is there anything that we can… uhm 49.

49: I think if you just… and you indicate that you are offering free treatment they will feel free to come.

KC: Alright.

49: And then when they come to access free treatment, that is when you then introduce

KC: The study.

49: Yes.

KC: So, what kind of treatment will they be receiving for free?

49: Like STIs ad so on.

KC: Uhm, alright, okay.

49: Yes.

KC: Anyone else, is there anything else that would facilitate participation on the study, anything else? 55

55: I think I encountered this program at some point, but I didn't show interest. When the program was being done, when they were given a bottle of drink and biscuits, the next week even those who wouldn't have come initially would be interested in…

KC: Going.

55: Yes.

KC: Alright, if refreshments are offered.

55: They will say ladies if the program is running next week, please call me.

KC: Okay. Alright, alright. So, as part of the research, on the study right, as part of the survey, we would want to test for STIs, however, its not everyone who will be tested, only those who are willing will be tested. So, after testing for STIs, the tests will be done in such a way that an individual will collect their own sample that will be tested. The study staff will just take them through the procedure and there will also be pictorial instructions, so they will collect their own sample in privacy. Do you think this will work, will adolescent girls and young women be interested, would they be comfortable collecting their own sample, would they be interested?

XX: They will be comfortable.

KC: Why?

Xx: Because its just like a pregnancy test right, it has a sample (instructions).

KC: Yes.

XX: Such that you know that if I collect my urine sample l then take these next steps.

XX: I think its better than someone saying I want to carry out this procedure on you.

KC: Alright. So, its better to carry out the procedure yourself than to have it done by a nurse.

KC: Or the study staff. Alright, its okay. Uhm, so looking at the issue that those who would have been tested will get their results. Those who would have consented to receive their results primarily those that would have tested positive for STIs, and they will also be encouraged to get treated for these STIs, because if left untreated they can cause

undesirable effects. So, we want to firstly hear from you how we can give them their results, the results are out and we have them, how do we give them their results? We have the results, we were here in Mazowe conducting our study, then we return to Harare and collect the results from the lab when ready, how do we communicate these results to an individual? 49

49: I think they can leave their phone numbers.

KC: Phone numbers, and we call them?

49: Like we then… you come back. .

49: Then you start communicating with them maybe through text messages or whatever, you send a message.

KC: Alright. So, we will come back and give them their results. Okay, so the results will be given in person.

49: Mmh. At the same place at which you met initially.

KC: Okay. Alright. What of places where you can access treatment of STIs, which places can you recommend for treatment of STIs, like here in Mazowe, if you take the results to such a place and people receive treatment there, it will work. Which places would girls and young women feel comfortable accessing STI treatment from?

[Silence]

55: I would suggest Concession and Howard.

KC: Concession and Howard hospital?

KC: So, is there a specific department or we just get to the outpatients department and we… someone just states that they have come for treatment of an STI. Is there… is there a special department that specialises in offering services to girls and young women?

55: Uhm, I'm not sure, but if the card (clinic card) has been written… because if its written when you visit another hospital you are given a new card upon arrival.

KC: Alright. Okay. Uhm it's okay. Are there other places excluding the hospitals that have been mentioned, are there other places that you can suggest that are better?

KC: What 55?

55: Clinics may lack medication

KC: For treatment?

55: Yes.

KC: So, hospitals are better.

55: They are better.

KC: Anyone else.

XX: What if you don't have the money to go to the hospital?

KC: Treatment will be covered by the study; the study will cover the costs.

XX: Alright.

KC: So, we would want suggestions of places to approach so that when results for girls and women who participated in our study are out, they can receive treatment there and we pay. So, the places that have been suggested so far are hospitals, any other suggestions? [Baby crying], none. So, our last question is that we were discussing different kinds of PrEP right, we mentioned the injection and ring right, so these are referred to as long term methods that work for a long time before accessing the service again. Its different from the pill which one might be taking everyday, you go for a month or 2 right. So, we want to get your opinion of this, how do you view these long-acting methods, can they be embraced by adolescent girls and young women, is the ring and injection method acceptable?

48: I think the injection is acceptable because if you are injected you will not be seen holding anything, and you will just return after 2 months.

KC: Alright. Mmh, anyone else, are rings and injections acceptable [baby sounds] and can they be embraced by adolescent girls and young women [coughs] 55.

55: With regards to acceptability, they… the injection as she said is more acceptable than pills. Someone who sees me coming from this place with a small container (with pills), say I have collected a 2 months' supply, if they hear a shaking sound from the container, they will not know the type of pills I had come to collect. They will start spreading gossip saying I heard a shaking sound as that of pills in a container in her bag, she must be on the program (ART).

KC. Alright, its fine, I had left out 1 question. Uhm, from discussions we had with other groups, they indicated that parents haven't accepted that girls and women, say girls who are aged below 18 are engaging in sexual activities in their lives right. They said parents need to be engaged and educated on PrEP issues so that they are aware and accept that young people are engaging in sex to ensure that the intervention is successful. So, looking at the issue of parents, what kind of support do young women and girls require from parents with regards to PrEP or initiatives on sexual issues?

[Background discussion]

KC: For them to know about their child specifically that they are engaging in sexual activities, or for them to just have an idea that there are initiatives to protect people from being infected by HIV. There are such and such initiatives for people who have HIV to take medication just for their knowledge or they should know every little detail about their child, such that the child will be able to approach them and say mum I am on PrEP without them taking offense?

XX: Yes, children should know, because I once witnessed "_" children being given family planning, they were collecting it also.

KC: Alright.

XX: Yes. So, it is acceptable

KC: Its is acceptable.

XX: Yes.

KC: Alright. What do others think of this, 55?

55: Its good for parents to know, if they discover that their child has the pills, they will not be shocked. They will; not be worried, they will not panic.

KC: Anyone else?

[Silence]

KC: Alright. If there isn't, we have come to the end of our questions, I don't know if anyone has a question, is there anyone with something to add to this discussion. Does anyone have something to add?

[Silence]

KC: None. If there isn't thank you so much for your time, thank you for… [Interjection]

48: May I…

KC: Yes.48, its okay.

48: I want to ask that if I am on PrEP right.

48: And my husband is positive, can I take my pills today and skip other days or I must take them everyday?

KC: Alright. Uhm, alright l will respond to that. I will respond to that, is there anyone else who has something to say?

[Silence]

KC: Alright. If there is nothing our discussion has come to an end right, so when we are done, we then move on to the issue of incentives that we read in those papers we signed. Then we will be done right, thank you our discussion has come to an end.

End of interview
