# Supplementary material for: Perceptions of pre-exposure prophylaxis among sexually active adolescent girls and young women in Zimbabwe–A qualitative study
Source: PLOS Glob Public Health. 2025 Dec 2;5(12):e0005396. doi: 10.1371/journal.pgph.0005396 (PMC12671731; doi:10.1371/journal.pgph.0005396)
Supplement: S1 File — (ZIP) [file pgph.0005396.s003.zip › S1_File/AGYW-FGD 07-Translation.pdf]

AGYW-FGD 07- Translation
Facilitator: KC
Note Taker: SM
Date Of FGD: 13/04/2022
Age group: 16-19 years
Translator: KC

KC: Alright so we are now starting our discussion right, as I mentioned earlier my name is Kudzai, this one is Sharon, we are from CeSHHAR Zimbabwe. We want to have a discussion with you about your views on PrEP issues. [Someone clearing throat] [Someone speaking loudly on a phone in the background] What can be done for PrEP to be used, what can motivate adolescent girls and young women to take PrEP. This gives us important information on the types of programs that can be designed. [Someone speaking loudly on a phone in the background] That can make PrEP to be taken and accepted by adolescent girls and young women in Zimbabwe, right. As we mentioned before our discussion might take between hour to 30minutes to complete. [A vehicle passing by] We will have role plays in our discussions as I have explained before. As we have agreed do not use your names, the numbers we will give you are the ones representing your names. If there is anything you would like to ask you are free to ask as we are discussing. So, as we start asking our questions, is there anything that you want to ask before we start? [Someone coughs] Is there a question?

XXX: Aah no.

KC: There is none? There is no question.

XXX: Uhh.

KC: Alright if there are no questions, my first question in our discussion is that have you ever heard of PrEP? Have you ever heard of PrEP?

ALL: Yes.

KC: Alright, all of you, you said yes you have heard about PrEP. Are you able to explain what it is? What is PrEP? Number…number 65.

65: Alright when I heard about PrEP it was said that PrEP, PrEP are pills that are taken by someone who is HIV negative. They reduce the chance of getting HIV.

KC: Alright.

65: Uhh.

KC: She said they are pills that are taken by someone who is HIV negative to reduce the chance of getting HIV. Others, what is PrEP? 64.

64: What I understood is that PrEP are pills or medication that is taken by someone who is at a risk of getting HIV.

KC: Alright.

64: Also they will not be aware of their partner's status or what…having many sexual partners and not knowing their status as well.

KC: Alright, uhh. Aah one will…will not be aware of their partner's status or the partner knowing that they have many sexual partners. Others, is there anyone with a different view? [Silence] Alright what about if we are looking at PrEP, is there anything you know about the types of PrEP which are available? Which might be readily available or not in Zimbabwe. What types of PrEP might you know? Number 62.

62: Uhh injection and ring.

KC: And what?

62: And ring.

KC: Injection and ring. Alright, others. Number 67.

67: And pills.

KC: Pills, alright. How do the pills work?

67: The pills you start taking them seven days before you sleep with a person who has a virus.

KC: Uhh.

67: Then if you are more…you have finished the seven days you can take here and there. You are supposed to take them at a certain time, the time that you would have started taking them, that is the same time you must keep on doing what, taking them.

KC: So, she said seven days before you have sex with someone that you know they are HIV positive so is it only for the person that you know is HIV positive or what?

67: And the person that you would have slept with.

KC: Alright. Aah what about after, how long do you take the pills? You mentioned before you have sex, what about afterwards, after having sex, how long do I keep on taking the pills? 65.

65: 28 days.

KC: 28, 28 days after. What happens after 28 days? Does one stop taking them, do I…is there a time that I will continue, that's where I want to understand, 61?

61: You continue.

KC: So you continue taking them.

61: Yes.

KC: So, can we say they are pills that are taken for the whole life or there have a certain time that you can take them, I take them and then stop for a while? Yes, 61.

61: As we were taught, they said you can stop them, you take and stop.

KC: But following that regulation that you take seven days before, you go for 28 days and you stop. When you are starting again, you repeat the same cycle.

61: Uhh.

KC: Alright, its okay. Aah what about the ring that you mentioned, is there anyone who has seen the ring? Are there any who have seen or used it? [Silence] There is no one?

XXX: Uhh.

KC: Alright, is there anyone who understands how it works? [Commuter omnibus conductor shouting-looking for customers] There is no one?

XXX: Uhh.

KC: What about the injection, is there anyone who understands how it works? [Commuter omnibus conductor shouting-looking for customers] [Silence] Alright, its okay so for ring right, ring is used by the health care workers, the nurses are the ones who insert it at the clinics, they insert the ring so the ring will be inserted below there. [Pointing towards the vaginal area]

XXX: Uhh.

KC: Then its inserted there for one month after every one-month they go back, and have it removed and the cycle repeats so the way the ring works. When its inserted it has some medication that will be released in the body so that you are protected against HIV. So, we have some samples of the ring that I want to show you so that you have an idea of how the ring is. So, this is the ring, vaginal ring for PrEP so just pass it around so that you feel how thick it is, how it looks like. That is the ring, that one.

XXX: That is the original one.

KC: That is the ring.

XXX: Just as it is.

KC: So that is how it looks like, so that is the one that will be used, it is inserted inside. It stays in your body releasing some medication, releasing some medication. After every one-month it is removed and have another one inserted so that is the dummy ring. [Bus hooting] So in Zimbabwe the ring has been approved so that it can be used but it is not

yet readily available like the way oral PrEP is. It is still being worked on, conducting research on the possible side effects but it has been approved to be used so I just wanted you to see the ring. The injection, injection here in Zimbabwe has not yet been approved for use but in other countries they are preparing to start using the PrEP injection. So, the PrEP injection the way it works, when you get injected it stays in your system for two months, after every two months… [Bus hooting] you go back and get another injection. After every two months you go and get your injection, that is how the injections work. Looking at the efficacy levels amongst the three, pills, injection, and ring we can say the injection has high efficacy in preventing HIV, it works…it has a high percentage of preventing HIV comparing with the ring. [Sound of a bus moving] Ring percentage is not that high, it is a bit on the average in preventing HIV. Then for the pills, the pills I am sure all of you have seen the oral PrEP.

All: Uhh.

KC: You all know them, right.

All: Uhh.

KC: Alright is this the container? [Showing participants, the container for oral PrEP]

All: Uhh.

[Someone clearing throat]

KC: I will not show you these ones, you are familiar with them right. What about information that you were explaining to me when we were asking about PrEP. All that information that you were telling me about PrEP where did you get it? Information about PrEP where did you get it, to get to know about PrEP for the first time, where did you know about it? [Someone clearing throat]

KC: Number 63.

63: We were told at the clinic, when we came to be told about PrEP.

KC: Here at the clinic, in Nzvimbo.

63: Uhh.

KC: So they would…that will be information being given to everyone or there is a type of people who will be given the information, what kind of people would receive the information on PrEP? 66.

66: They…they…you would have come wanting to get treated, wanting to get tested then you tell them of your problems, that is when they will tell you to take PrEP.

KC: Okay. Others, besides here is there somewhere where you would get information on PrEP? Number…68.

68: We were told by others who had started taking it.

KC: Alright those who had already started taking, using PrEP.

68: Uhh.

KC: Alright, okay 68.

68: Uhmm there we some campaigns that were done in school on taking PrEP.

KC: Who was doing them? Was it an organization?

68: It was an organization.

KC: Ohh it was…what was the name?

68: It was for the DREAMS.

KC: Ooh the DREAMS.

68: Yes.

KC: Alright, 65.

65: We heard about it in the community hall.

KC: Uhh.

65: People from PZAT came.

KC: Ooh people from PZAT are the one who gave you that information. 69.

69: I heard it from sister XXX.

KC: You heard it from who?

69: From XXX.

KC: What does she do?

69: She works here.

KC: She is a nurse here.

69: Uhh.

KC: Alright, 61.

61: I heard it also by XXX, she works here as well. She told me about it after I had told her about my issues.

KC: She is a nurse who works here?

KC: PrEP champion, alright its okay. Now we are getting to…before we get into the groups that I mentioned before. I understand everyone here is using PrEP.

XXX: Uhh.

KC: Alright, looking at your use of PrEP are there challenges on using PrEP? On accessing it, taking it, are there any challenges there? 65.

65: Personally, from the time I started taking it I don't have any challenge that I have encountered.

KC: Alright.

65: But we were asking that if it is possible for us to have our corner that is only meant for PrEP.

KC: Alright, currently where are you accessing PrEP from?

65: We are getting it from OI (Opportunistic Infections).

KC: Here at the OI department.

65: Uhh.

KC: Alright so what challenges are there that happen with taking it from the OI?

65: Like that side everyone who is…will be going there, some will not be able. Some are embarrassed to take PrEP because the room that we use is one.

KC: Alright.

65: Yes.

KC: Uhh.

65: So for others if you are seen standing in that line, the day will end having false information about you being spread in the community.

KC: Alright, alright. Aah others, are there challenges? Are there any side effects that are there on PrEP so far from using it? [Silence] Does it have any side effects?

65: Uhmm uhmm.

KC: It does not have any side effects that you have experienced?

XXX: Uhh.

KC: The reason why I am asking is that from the discussions we have done so far, from the ones we have discussed with some were using and some were not using but they would mention that we hear that it has some side effects so because of that we would not want to take PrEP that is why I was asking if you have experienced any side effects from

taking PrEP? [Silence] There are none? [Silence] Alright its okay. Aah now we want to split into our groups I mentioned earlier. Our groups are three, they are three, so we have group one, group two, and group three. So, our scenario right…I left them in the car, alright. So, I will explain each scenario then we split and get into our groups. We can split according to our sitting positions but if you want you can split the way you want, it's not a problem. So, our first scenario, it's about two friends named Chido and Koko so we would want two people who will act out the scenarios, bringing out Chido and Koko's issues. Chido is 16 years, but she has a sexual relationship with an older man around 50, 60 years right. he is older than her, so Chido recently started using PrEP because she is worried of getting HIV because of her relationship with this older man, right.

XXX: Uhh.

[Someone clears throat]

KC: She thinks of her friend named Koko, her friend is 19 years, and she is also at risk of getting HIV as she also involved in a sexual relationship with an older person right. So Chido goes to her friend Koko telling her about PrEP, advising her to take PrEP because of this and that. So, you are doing the role play, having the discussion, bringing out this issue. Chido telling Koko about PrEP and we hear Koko's responses. In this scenario we want to know if someone is approached by their friend, and they are told about PrEP and asked why they won't take it. How will one react after being told this issue in real life right? The second one is for two women, one is mai Bhobhi and the other one mai Juru. Mai Bhobhi is 23 years, and mai Juru is 21 years old, and they are both married right. Mai Bhobhi her husband has got a habit of having too many girlfriends right so mai Bhobhi is worried that she might get HIV with the way her husband is behaving. So mai Bhobhi heard about PrEP on radio, she went and took PrEP at the clinic near her. She now has six months using PrEP, but she is now considering stopping the use of PrEP, she does not want to use PrEP, so we want a role play between mai Bhobhi and mai Juru discussing this issue. Mai Bhobhi saying her reasons why she wants to stop using PrEP so we want you to tell us that in real life what can make someone wants to stop using PrEP.

KC: After using it for six months, she is married, she is in a situation where you can get HIV, what can make someone want to stop using PrEP. The third one is about three friends who learn at the same school. They have their sexual partners, at their school they have been selected to design a PrEP program, the program when its implemented it will make adolescent girls and young women want to use PrEP and continue using it so they will be discussing listing the things that they think are important, that should not be left out in a PrEP program. For a PrEP programme to succeed for adolescent girls and young women it should be like this. The pills will be available at such places, being dispensed by people who are like this. The PrEP is like this and that, that what we want to hear in the role play so these are the three role plays right. So for now we are pausing our recording then we break into the three groups. Each group have their own discussion after three minutes we come back, when we come back, we are no longer pausing, we are not breaking. We will be giving each other chances to say this group does their role play, then we have a discussion on that. The next group does its role play and we discuss until we finish. We will not break for now until we are done so we are now pausing our recorder.

[Recorder paused as the participants are discussing their role plays]

KC: So we are resuming where we left off, Chido and Koko do your role play. Raise your voices.

Role Play 1: Chido and Koko

Koko's mum: Koko.

Koko: Mum.

Koko's mum: I am going at my friend's place, I will be back shortly.

Koko: Alright mum.

[Koko singing as she sweeps her mum's yard]

Koko: Aah hey Chido, you could not wait until later?

Chido: No Koko I could not wait.

Koko: Uhm, my dear are you missing me that much, so early in the morning when I have not finished sweeping the yard you are already here.

Chido: I had to come in the morning when your mother is not around, is your mother around?

Koko: Is everything ok?

Chido: Yes, everything is okay.

Koko: Why do act like my mother, is there a latest news?

Chido: Yes.

Koko: Are you alright?

Chido: I am alright, how are you?

Koko: How is life?

Chido: My life is good my friend.

Koko: Yesterday I almost got in trouble.

Chido: What happened.

Koko: Its hard my friend.

Chido: Aah is there a secret here.

Koko: Aah I will tell you because Dad might wake up and beat me up.

Chido: Hooo, aah it's alright. How is your boyfriend these days?

Koko: Uhmm these days, its hard my friend.

Chido: Your boyfriend do you think you are the only one there?

Koko: Aah these days its hard, he does not even want to use condoms, he does not even want.

Chido: Why don't you refuse.

Koko: Ahh its because I love him, what can I do my dear.

Chido: You know that I am dating that older man.

Koko: Yes.

Chido: That old man, you he has a wife.

Koko: Yes.

Chido: So, I heard about the PrEP programme on the radio. Do you know what PrEP is?

Koko: Uhmm I heard about it in radio, aah aah I heard about it at school but I don't really…I did not quite understand how it is.

Chido: That's the problem of not buying radios. You should have heard for yourself on the radio, you will be informed.

Koko: Alright, yes so…let me give you somewhere to sit. Sit down but make it fast before my mother comes back, I want to sweep.

Chido: Alright, PrEP are pills that…its available in different forms, three types. It can be pills, injection, or ring, do you hear me.

Koko: Umm.

Chido: But currently most people are using the pills. You go to the hospital you…if you get there you will be told in full because if I tell you, with the way you forget, you would have forgotten by end of day but if you get to the hospital and you let them know I have come to collect PrEP, you will be given all the information.

Koko: My friend I want to ask about the pills, injection, and the ring. Ring do you put it on the hands, do you use them all three at once?

Chido: Aah Koko you have started.

Koko: Yes.

Chido: Ring is the one that protects the uterus "_+". [Wind noise]

Koko: That's why I ask.

Chido: No, its inserted down there.

Koko: Because if we hear of a ring, we will be thinking of the one for the hands.

Chido: It's inserted in the vagina.

Koko: Ooh its inserted in the vagina.

Chido: You do not use all three of them, you choose one that you want to use. Like myself I choose the pills.

Koko: Why did you choose to use the pills?

Chido: Because then pills when I went to the hospital I was told about the pills. [Baby coughing]

Chido: Plus, about the ring I was afraid thinking that it can affect me, it can go deeper getting inside each time I will be sleeping with a man so I was afraid.

Chido: So, I opted to use the pills.

Koko: So, if I go now the PrEP will be readily available.

Chido: Yes, it will be readily available.

Chido: If you go to the hospital they will explain well.

Koko: Alright.

Chido: So, I am now going before your mother comes.

[Other participants clapping hands]

[Wind noise]

Koko: It is okay let me quickly sweep the yard before she comes back, I will be in trouble here.

KC: Thank you Chido and Koko, thank you for your role play. We are now proceeding to the questions for the role play but before we go to our questions, I have one question. Here at the clinic PrEP is given to what type of adolescent girls and young women?

65: At XXX (local clinic) they give from 16 years to 24.

65: Those who are sexually active, 16 to 24 it is the DREAMS age group but they give everyone who wants PrEP.

KC: Alright, those who want PrEP and are sexually active.

65: Yes.

KC: Are the others with a different opinion from what 65 has said? [Silence] We now want to discuss issues related to Chido's role play right. My first question is that in the life we live or staying in our community are there girls who are like Chido who have sexual relations with men who are older than them?

XXX: Uhh.

KC: Does it happen?

All: Yes, uhh.

KC: If it happens you explain to us the situations, the reasons why one gets into such relationships. 63.

63: That is common here because if we start dating early, that person we will be thinking we are going somewhere and he dumps you. After dumping you, you then date an older man who has money, you now after the money saying aah he has money let me date him. Then we have a meeting point, where he gives me money for living since, I will not be doing anything. If I want to buy some sugar and paying some rentals, I will be seeing that things are well for me, but it will be something affecting my life. That is why I realized PrEP will be helpful since we sometimes do things that are not well-planned out in life.

KC: Alright, others relationships with older men, 65.

65: The issue of peer pressure if we look closely at those ages from 16 to 24.

65: I would have seen Koko maybe she has a new phone bought to her by her older men.

KC: Uhh.

65: Since I will not want to be left behind, I ask Koko where she got the phone. Koko tells me that it was bought for me by…by my older man.

KC: Uhh.

65: Sometimes it does not mean that in our home they cannot do it for me.

KC: Uhh.

65: But it is an issue that I now want my older man who buys a big phone for me. Maybe at home they can afford a small one.

KC: Uhh.

65: But because I saw Koko with a big phone, the Itel type (cellphone brand) I now want mine as well.

KC: Alright.

65: I now want…its now making me look for an older man that I will be sleeping with, now doing everything for me that I cannot do.

KC: [Baby crying] Alright so peer pressure, want…wanting money. Is there anything else? Is there anything else, yes 68?

68: Poverty, poverty on its own sometimes pushes you to do that.

KC: Uhh.

68: Like at home you can see that aah we have eaten sadza and pumpkin leaves in the morning. In the evening we get maybe some…some veggies and you see the ones at your next door are eating good food. Then you say aah let me do this maybe it can work out well.

KC: Alright, wanting to provide better things for the family. 60…its 60 what? 69.

69: At times you would have been abused as a child so your brain will always be thinking about those things.

KC: Alright, abuse as a child. 61.

61: Let say in our home we will be struggling; we will not be getting anything. So, if I get my older man who has money, he gives me money for…I will be able to provide. Sometimes I might be going to school, he is now giving me money to buy books, to go to school.

KC: Alright, are there others with a different view from the ones shared? [Silence] What about looking at…the relationship…looking at Chido and her older man's relationship. Can we say Chido is at risk of getting HIV also her risk is coming from…how does she get the risk of getting HIV? 67.

67: Uhmm Chido might be a child, a child who is staying with her parents' right. Going to school, she has met the older man and sometimes she might forget to take what, her pills. So, she can come across that challenge and she sleeps with that older man when she has forgotten her pills here.

KC: Alright, okay. It is okay, 65.

65: Chido can…sorry come again.

KC: We said the relationship that Chido has with an older man who is around 50, 60 years is she at risk of getting HIV? Also how does she get the HIV in such a relationship?

65: Alright.

KC: 65.

65: Chido looking at her age, she is 16 years, the older man maybe is around 45, 50. This older man is not…Chido is not the only one dating this man maybe they are one, two at the time when they have sex. Chido cannot tell the older man to wear a condom because he is much older than her, there is no way she will tell him to wear a condom. So that gives her a big risk of getting HIV because she cannot decide on things since she is still young.

KC: Alright.

65: The older man is the one who will be doing whatever he pleases with her.

KC: Others, are there any with a different response from the ones mentioned? Her relationship, she does not have control over her relationships so if this man says he does not want a condom, there is nothing she can do.

XXX: Chido is at a risk of getting HIV.

XXX: Because she does not know this older man's status.

KC: Uhh, alright. She does not know the status.

XXX: Yes.

KC: From the other discussions we have done, it emerged an issue that number 65 has mentioned that they will…the older men will not want to use condoms. Are there other reasons that you might know or that you hear in the community, as to why they do not want to use condoms in such relationships? 63.

63: Sometimes what makes the older man not want to use condoms, he will be having a lot of money.

KC: Uhh.

63: Since I am after the money, to me he can tell me I do not want to wear a condom because I love you.

KC: Aah alright.

63: So, I want to sleep with you without protection for you to see that I love you. Plus, what is it that you not having, I am giving you all the money.

KC: I am giving you.

63: Since I also want the money, I will then say let's just do it, that is when you will get infected.

KC: Okay, 65.

65: Alright, to add on what 63 has said, he is providing me with everything, and I now want to say to him wear a condom. They usually say you do not eat a sweet in its wrapper, it's not tasty.

KC: Uhh.

65: So, we will be doing it without protection, you are no longer thinking of saying to the older man wear a condom.

KC: Alright, uuh is there anything else? [Car hooting] Ehh 67.

67: From what I hear, condom use has its own price and without a condom it has its own price so sometimes it will not be the older man pushing you to wear what, to wear a condom. You will not be pushing him, but you will be pushing yourself to say I…if with a condom its $50 and without a condom its $100.

KC: Uhh.

67: I have seen him with a $100 note, so I now want that $100.

KC: Ok I now want money more that protecting myself.

All: Uhh.

KC: Aah proceeding to our next question right looking at adolescent girls and young women is it a common thing or it is something that happens amongst them to share with others. In families, in relationships, sharing with others their health decisions. For example, I went and got tested for HIV and I come back telling someone at home or I have an STI I tell someone from home that I tested positive for an STI. I went and treated, I have

done this, is it a common thing for adolescent girls and young women to share their health decisions with other people. 68.

68: Uhmm its not common.

KC: It is not common?

68: Uhh.

KC: Why?

68: It is your secret because if I tell 64 and tell her I don't want this to be known. Plus, one does not want their status to be known by many people. You tell 64, she will tell 63, its now spreading. When I move around, I will be labelled as someone who is on the pills, she is on the pills.

KC: Alright, okay. 61.

61: Maybe the person I will be telling they will not be able to uphold the confidentiality so you might hear about it spreading in the community. At times there might be someone who is interested in me, and he is now asking me that what about the things you told so and so.

KC: Hoo…

61: This how you are.

KC: Alright. Are there others who have other reasons why they cannot share. So, you are all saying they will be afraid that this issue that I have told this person it might spread to the whole community.

61: Uhh.

KC: Alright, it is okay. Are there people, amongst the few ones whom you can feel comfortable sharing information. One person whom they can share their personal issues that I did this. Are there people who fit in there, what type of people?

XXX: You can tell your mother.

KC: Your mother?

XXX: Yes.

KC: Okay, 65.

65: As for me there is no one I will share with.

KC: There is no one you will share with.

65: Uhh, because sometimes I can tell my mother who will tell my sister. When you fight, she says that is why you were treated for an STI, you were rotten. So, if I am sick with my STIs, I will get treatment quietly and hide my card.

KC: Alright, 68.

68: Aah as for me the person who can know my status is the nurse where I will go for treatment not to tell anyone else.

KC: So, nurse.

KC: Alright, 63.

63: As for me I cannot tell anyone because if you tell someone that I went and got treated for an STI. I am supporting 68, if you have a misunderstanding with that person, they will reveal the secret. That is why you went and got your STI treated so I think that if it is about these diseases, I will keep it to myself, we will discuss other issues which are not these ones.

KC: Alright, 61.

61: Uhmm if I do that I will go and tell my mum.

KC: Uhh,

61: If it's the time when I would have received my pills, when I am taking my pills she will remind me to take the pills.

KC: Alright so women are the ones who have been mentioned, all the others aah it's just keeping it to yourself and knowing it yourself. Alright, aah what about friends, do they work?

[Wind noise]

XXX: Uhmm.

KC: Best friend.

XXX: Ahhh.

KC: It will not work.

All: It will not work.

KC: Why?

65: Because best friend also has her best friend somewhere.

KC: Oooh.

65: She will go and tell her; I have told her and say do not tell anyone. This one will tell 62 and say do not tell anyone, that one tells someone and say do not tell anyone.

61: Aah as for me telling someone is not proper even if they are your best friend.

68: The best has their own best.

KC: Alright, best has their own best so it is a problem. [Giggles] 67.

67: Like this play that we did for Chido and Koko right, I told my best what happened. We were in the same situation.

KC: Uhh, uhh.

67: I…Koko is not in the same situation, Chido is the only one who is in this situation. Obvious I am now going to tell others that aah Chido did this and that. That is what she is doing.

KC: That is what she is doing.

67: Or she is the one who is…I am now going to tell her blesser that she is taking pills.

KC: Alright.

67: I now want her to be dumped.

KC: Alright, I get it. Aah what about the ones from church?

XXX: Uhmmm.

KC: The pastor's wife, the pastor, the elders. Do they work?

65: They are the same.

68: It is the same because elder has a wife at home. As he gets home, he will tell his wife. Aah that wife will spread it and tell someone, her friend. My friend, aah that child did this and that, if you were not yet married you will not get married. This one is not a marriage type, this one did this and that, leave her, she not a suitable candidate.

KC: Alright so it is a no for the church ones.

68: Yes.

KC: Alright, what if we say…there was a time when we asked about your knowledge of PrEP, where you got the information. It was mentioned that from here, from PrEP champions, from some awareness that were done in the community right. Are there any other places that you can suggest if PrEP information is available at such places adolescent girls and young women can easily access the information? Are there any other places that have not been mentioned before where PrEP information can be

accessed from? Suggestions to say at so and so, if its available at such places it will work.

61: The village health care workers.

KC: Uhh.

61: Because "_+".

KC: Alright, 65.

65: Even on TVs, having some dramas talking about PrEP. If you do not have a TV, then on a radio.

KC: On TV, radio, from the village health workers. Are there any other places? Or other platforms that can be used to spread the information in people? 63.

63: In schools.

KC: In schools.

63: Yes.

KC: Which ones? Primary, secondary.

KC: Such information is included.

63: Uhh.

KC: Now we want to look at what happened between Chido and Koko right. Aah what are your views on Koko's responses when she was given a suggestion being told about PrEP. What's your view on the response she gave?

63: She wanted to refuse as she did not know how it works so she was asking why you said I should use it. Why do you want me to do it, then she was told that you should…you are doing it for this and that.

KC: Uhh.

63: Then she reasoned a bit and said so let me do what you have said.

KC: Alright, is that what happened in real life? Such scenarios, a situation like this one. Is that what happens in real life? 68.

68: Uhh that is it.

KC: Uhh.

[Someone shouting in the background]

68: Also if you are telling someone that you know that, that is what they do. If you tell them, one might agree but for those ones whom you might not be sure of telling them.

Most of the time, the first time she can respond in a rude way that amongst all the people why did you select me.

KC: Uhh, others, is there a different view? [Silence] What other responses can we expect from a situation like this one. Chido has told her friend Koko about PrEP, are there other responses that have not been mentioned and not said by 68 that can come out after someone has being told about PrEP, being encouraged to take PrEP. Are there other responses that can be brought up? [Silence] Nothing.

63: Uhh.

KC: Alright, it's okay. What about PrEP looking at advantages of PrEP to adolescent girls and young women, what do you think is good about PrEP? Starting from…alright let us take it back a bit. Let us start from what do you think can encourage adolescent girls and young women to take PrEP? The reasons of taking PrEP, what are they? Let us start from there, 63. If we state the reasons, then we mention the…the advantages of taking PrEP, 63.

[Sound of a motorbike]

63: The advantages of…of taking PrEP is that I will be having boyfriends even five whom I will be dating. Amongst all the five, no one would have initiated getting tested together, all of them I want them because I want the money. So, I think if I use PrEP I can meet someone who might say they do not want to use condoms.

KC: Uhh.

63: I can meet someone who can say I am protecting my health, I am using condoms so if I use PrEP it can help my health. If I keep on adhering, it can help me health wise and it makes me live in a good way.

KC: Okay, others. The situations, the reasons that makes someone to take PrEP and the advantages, 61.

68: One might be…might not be faithful to their partner like you are married.

KC: Uhh.

68: Your husband his behavior will be…you will not be understanding it so it's better to protect yourself.

KC: Alright, uhh. In marriage you will not be understanding your partner's behavior. Yes others, 61.

61: When you have been separated and reconcile, you will not be aware of his status, if you say to him let us go and get tested, one might refuse. I will be the one who will be getting tested frequently alone so it's good in that even if he comes with his diseases, they will not affect me.

KC: Alright. Others, are there others with other reasons that are different that might make someone want to take PrEP? [Silence] Alright, it is okay. [Sound of a moving vehicle] What about what can hinder, we were discussing about what can encourage right. What about what can hinder adolescent girls and young women to take PrEP, what is it? 65. [Sound of a moving vehicle]

65: Some young women especially the…the married ones.

KC: Uhh.

65: Sometimes the way we say it.

KC: Uhh.

65: Let us say I heard…I heard about PrEP during a session, we have just had a session.

KC: Uhh.

65: Then it's said men who…men cheat and if you have had some sexually transmitted infections, the way I speak to my partner when I get home. I will get home and say I am tired of getting injections for the STIs, you are to blame so I am at a risk, and I am now taking PrEP. It might affect my partner such that he can stop me from taking PrEP.

KC: Alright.

65: So, it will be good to discuss in a good way with our partners, in a good way such that they can accept it. If they can, some might even come together to take PrEP.

KC: Alright. Uhh others. The reasons, eeh 68, what can be barriers to taking PrEP.

68: "_+" that you are not being faithful to me, you are being faithful to other people "_+" because I don't know if you want PrEP or you want to stay with me. [People speaking in the background]

KC: Alright so not being allowed by your partner, he is the one who can refuse for you to take PrEP saying you are not trusting me. Alright, what if you are honest with him that you are not trusting him?

XXX: One might tell you that I don't love you anymore.

KC: He can dump you.

KC: Alright, 60. Yes 60.

60: Some of the things on PrEP, it's like I will be coming to collect here. I am currently staying in Harare.

KC: Uhh.

60: Each month coming here, my boyfriend will ask me that why is it every month you will be coming to the hospital.

KC: Uhh, uhh.

60: Then he will say you are on treatment, aah its difficult since you will be known that every month you come from Harare coming to collect here. [Sound of a moving vehicle]

KC: Alright so the most emerging issue is that the partners are the ones who might not be understanding.

XXX: Uhh.

KC: 61.

61: Personally, my husband will then say you are taking some pills, are you the one who has been behaving in a way that makes you get infected, why are you taking pills.

KC: Alright. What if you explain to him, explaining that PrEP works like this and this. It is taken by people who are like this.

61: For my husband to understand it, he was told by my mother that your wife that is how she is for her to start taking the pills. Know that there are many things happening in life, that is how he got to understand.

KC: Okay, 65.

65: Alright sometimes in the communities we stay in, comes wrong information. So, if you tell your friend that I have thought of taking PrEP because I am at risk in this way. One will tell you that it is better to die with the disease after having infected because those pills they trigger the disease to come out so sometimes the information that we get from our peers in the villages it can make someone not want to take PrEP.

KC: Alright, 68.

68: Sometimes one will still be going to school, for you to tell your mother that I am now going to take PrEP its difficulty.

KC: Uhh.

68: It can be a barrier to taking PrEP plus you will not be having a place where you take it.

KC: Alright, what will be the problem with your example. A child who is going to school for her to take PrEP living in that home, what is the problem around these issues? 60.

60: Aah they will wonder that you are engaging in sexual activities. Is there someone you are engaging in sex with, at my age I cannot say mum aah I have a boyfriend that I am dating.

KC: Why?

60: It will be…it will be hard; they will be saying you are still a child. They will not be suspecting that you are already doing it, but you would have started it a while ago.

KC: Alright, 65.

65: Even in the hospitals, let us say I am 16 years or 15, but I know fully that I am already engaging in sexual activities.

KC: Uhh.

65: I come to the hospital and say I want PrEP; they will start saying at your age why do want PrEP, but I know that I am having sex every day.

KC: Alright, okay. 63.

63: I want to support on PrEP, very true information…I was once not interested in PrEP because it was said it triggers the release of the disease. Sometimes you might be going to get tested and testing negative, not testing positive. So, if you get on PrEP you will quickly test positive.

KC: Coming out.

63: So, I heard that information three times, I would start using it and stop, start using it and stop, I did not want to test positive. Then I was told that that is not how it works like. Some of the information that we will be told will be too exaggerated, even if you go and get tested the explanation that you get. Aah you will be realizing that you will be having a bad record.

KC: Alright so the information will be incorrect. Alright, there is an issue that was raised of partners who will not be supporting the other partner to take PrEP and parents, especially mothers. They will not be understanding enough to support their children to take PrEP. On those issues how can it be resolved, starting with the partners together with the parents? How can it be resolved for it to be acceptable to them, to the men, 65?

65: For men I think that when we have those DREAMS sessions.

KC: Uhh.

65: If they can gather men so that they hear for themselves.

KC: Alright.

65: That what is PrEP, its better rather than for me to explain to him.

KC: Uhh.

65: Because if I say I want to take PrEP he will say so you are saying I am being promiscuous.

KC: Uhh.

65: But I will be seeing that the situation at home, I have been sick with some STIs so and so. I am seeing that I am risk, my partner is being promiscuous, but they do not understand so if there are meetings for men alone. Asking what they want to know, I think it will be helpful.

KC: Alright so that is the support. 69.

69: If we take PrEP, the work that some of us do our boyfriends will be saying PrEP is for AIDS.

KC: They will be saying PrEP is for AIDS.

69: Yes.

KC: Alright so what can be done for men to fix that information? To know what PrEP is for PrEP is not for AIDS, for them to know what can be done, what has not been mentioned by 65? Is there anything else? [Silence]

XXX: Hmm hmm.

KC: Alright, yes 68.

68: When you come to collect the PrEP pills it is better to come with your husband, he gets a full explanation and gets to hear it for himself.

KC: Alright so you bring the husband along. Here at the clinic, getting my PrEP and having an explanation of how it works and its purpose so that when I get home I will not be in trouble.

XXX: Uhh.

KC: What about the parents? To the parents what kind of support might be needed? What can be done to resolve the issues with the parents that…to understand their views on sexual health issues? 65.

65: For the parents, it is the same with the men. They need to be gathered around be it in schools mostly.

KC: Uhh.

65: For them to be told that yes, we the 16 years old are having sex.

KC: Uhh.

65: Maybe it will make them allow their children to take PrEP because for me to her that mum I have thought of taking PrEP, whilst I am 16 years. She will…she will not agree because in her mind she will be seeing me as a virgin who is not yet doing those things.

KC: Alright.

65: So, if they…they are educated that your children are doing it, maybe it will work.

KC: Alright, sitting down with them and they get information about these issues. So that they understand that these ages are already engaging in sex.

61: [Sound of a motor bicycle passing by] I will go to the community health care workers.

KC: Uhh.

61: And explain to them, the health care workers are the ones who will come from where, from her home and go and explain to my mother. Maybe she will quickly do what…

KC: Understand.

61: Understand.

KC: Okay, 68.

68: When the parents are meeting, it will be good for us not to be around because if they…if they are addressed in our presence, they can say so they want to encourage promiscuity, for us to let you be promiscuous. So, it needs to have them alone, they are told on alone, if they see the things at home they will not be surprised.

KC: Alright, it's okay. We have understood, thank you. [Car hooting] Looking at the venues, you mentioned PrEP is available here at the clinic, are there other places where PrEP is available in this community? [Silence] There is none?

63: Uhh.

KC: Alright, we now want to hear your views about where else do you think PrEP can be available? That can work well, if PrEP is available at such a place adolescent girls and young women can easily get it. Which other places can you think of? 67.

67: Closer to the clubs.

KC: Which types, clubs that are in beerhalls?

67: For the beer, if there is a space for PrEP access point, things can be well.

KC: So, it will be an office setup or outreach who come on some days and give PrEP. How will it be like?

67: It…it needs to be an office.

KC: Alright, to have a set-up in such places.

67: Yes.

KC: Alright, are there any other views? 68.

68: And in schools that…there are some teachers who are part of the…the DREAMS.

KC: Alright, teachers who are part of the DREAMS. They give PrEP through that channel.

68: Uhh.

KC: 65.

65: Even at the clinic it will be safe but having a point meant for PrEP only.

KC: Alright not integrated with the HIV ones.

65: Because sometimes I will get there and there is long queue so I would prefer accessing PrEP at its own point.

KC: Alright, 61.

61: Even having it in the communities because some of us where we will be coming from it will be far, when you get here you will be tired. Then you stand in the line, you leave this place when its already dark.

KC: Alright so in the communities so in communities that are…where people stay where would be ideal to have the PrEP, in the places where people stay? 62.

62: At the village health workers' houses.

KC: Houses of what?

62: The village health worker.

KC: Alright, the village health worker's houses. Alright, are there others? What about looking at the pharmacies, is it okay to have PrEP in pharmacies? That PrEP is now accessible at the pharmacies, 65.

65: Buying them or being given for free?

KC: Let us say being bought.

All: [Mumbling- it will not work with buying]

63: Buying, it will not work.

61: We will not be getting money.

KC: It's not possible to buy.

63: We will not have the money.

KC: Okay, its fine. Let us say…so is there an amount that you can say is affordable?

XXX: None.

KC: By the adolescent girls and young women, is there an amount?

XXX: If we start on the amount…

XXX: That is when…

XXX: People will not take it because they will be saying we need to pay.

XXX: People will not take PrEP because they will be looking for someone to pay for them.

XXX: They will not take PrEP also we will be saying we are going back to the older men who give us money. Sometimes if it's accessed at the clinic, it will not be available now being told go to the pharmacy and buy so it will not work as it will be costing us and we leave it.

KC: Alright, okay. What if its available at a pharmacy at no cost? Let's say a system has been designed for such and such pharmacies where it's accessed for free to the adolescent girls and young women, does that work?

61: Yes.

XXX: It will work that is if at the pharmacy they give you what you came for without asking a lot of things.

KC: A lot of things like what?

XXX: Asking you are not yet 16 years, and you are coming to get PrEP. You will be asked what you use it for, do you have a husband.

KC: Alright, 67.

67: I am disagreeing with the pharmacy thing; PrEP should be accessed from the hospital and places like that. At the pharmacy they will end up being greedy with the PrEP, wanting to do a business.

KC: Even if they initially said they are giving it for free, they will end up selling it.

67: Selling it, yes.

KC: Alright, to those that said in a pharmacy it will work. What will be the advantages of giving PrEP in a pharmacy? 61.

61: Because at the pharmacy its…at the pharmacy it will be different from the hospital. Where you have the OI (Opportunistic Infections) taking their pills for HIV treatment and us who are taking PrEP we are all meeting there. Now at the pharmacy I am just going to take my PrEP and go home.

KC: Alright, that will work. Looking at those who give PrEP, the person who gives PrEP to adolescent girls and young women, what kind of a person will be preferred? Let us say you have been asked to describe the type of person you want to give PrEP, starting with the age, character. What kind of a person would you want? [People talking in the background] 65.

65: We want a young person.

65: Haa someone who is friendly, when you approach them, they are not angry, who is …is not moody. Sometimes we notice that one will be stressed over their personal issues at home. They come here and they are unfriendly towards us, you even think it is better if I stop using this PrEP whatever happens, happens so we want someone who will be smiling and happy. Who does not say today they are is smiling, tomorrow they are moody. The way we see them today may it remain consistent throughout.

KC: Alright, alright. Aah what if we are looking at the venues of PrEP dispensing right, would you prefer a venue where PrEP is available, family planning is available, STI treatment is available or a venue where PrEP only is available? Would you want …what kind of a venue will be preferred, 69?

69: Everything should be available.

KC: Everything, how good will that be?

69: That will be good for us since we will be tired and we come here and we wait in the queues, then you want to get the injections again, it will not work.

KC: Alright so if you get everything at one place. 63.

63: I think that there should be STI treatment because if we get treated for our STIs. I get treated, then I am going to be initiated on PrEP, I get my PrEP which will be a secret between how many people, the two of us.

KC: The two of us.

63: The two of us such that it will not come out that aah this time around she is affected by the STIs so I think it will be alright because the one office that we will be accessing is only…is only one.

KC: Its only one. Alright, I understand. Aah now we want to have the role play number two for mai Bhobhi and mai Juru. Mai Bhobhi and mai Juru go ahead and we move on, we almost done.

[People talking in the background]

[Sound of a moving vehicle passing by]

KC: Move closer to the recorder.

Role play 2

Mrs Bhobhi: How are you Mrs Juru?

Mrs Juru: Aah I am well Mrs Bhobhi, how are you?

Mrs Bhobhi: I am well.

Mrs Juru: How is home?

Mrs Bhobhi: We are well.

Mrs Juru: How is Mr Bhobhi, aah we can see it. You are being looked after well.

Mrs Bhobhi: Aah Mr Bhobhi has troubled me.

Mrs Juru: What's wrong?

Mrs Bhobhi: Uhm the issue of promiscuity

Mrs Juru: Uhh your husband still does that.

Mrs Bhobhi: I do not know what to do my dear.

Mrs Juru: Aah it will be okay my dear, that's how most men are, none of them is better.

Mrs Bhobhi: Uhm its now too much, I don't know what to do Mrs Juru.

Mrs Juru: Isn't it you said you were taking PrEP Mrs Bhobhi.

Mrs Bhobhi: Yes I was taking it but now I thought let me stop using it.

Mrs Juru: Why are you thinking of not using PrEP my dear, it was protecting you. With your husband who is promiscuous, my dear you have forgotten about the STIs that you were treated.

Mrs Bhobhi: Aah I just thought its not working because the pills my husband is taking them and throwing them away, then I thought aah better I leave it.

Mrs Juru: *You hide them Mrs Bhobhi, you put them in a secret place, but you know that when its time. Isn't it seven o'clock your husband will be drinking beer? If its seven o'clock before he comes, you take your PrEP rather than for you to stop using it. The way you are, you get diagnosed with HIV, what about your child my dear.*

Mrs Bhobhi: *I have stopped using it for a while, I am not infected already.*

Mrs Juru: *No, my dear, go back to the hospital and get tested again. You tell them that I still want PrEP because I am at a risk my dear. Isn't it recently you said you got some injections for an STI, are you thinking about that.*

Mrs Bhobhi: *Aah I am now thinking that whatever happens, happens.*

Mrs Juru: *Aah its better to protect my dear, if the chance of protecting is there, protect my dear.*

Mrs Bhobhi: *Aah so I will go to the hospital and get tested and then take PrEP.*

Mrs Juru: *Aah its alright, you would have done a good thing. Looking after your health and that of your child is an important thing.*

The End.

[Other participants clapping hands]

KC: Horaiti.

KC: Alright.

KC: Thank you, Mrs Bhobhi and Mrs Juru right. Looking at the role play, does it happen in real life for adolescent girls and young women. Are there any who are in such situations like Mrs Bhobhi's, who meet men who are like Mrs Bhobhi's husband? [A vehicle passing by] Does it happen? Is it common?

63: Uhh.

KC: How will it be like; may you explain that its…it will be like this and that.

61: Like what Mrs Bhobhi was saying she was saying it's…I used to take family planning, but my husband would take those family planning and throw away. I realized its better I come and do what, I come to the hospital and change, not take the pills, and have the depo injected.

KC: Alright.

61: But after getting the depo I told him. For me to keep quiet about it, after getting my date for me to disclose that I am now going to get my depo I did not know how I was going to say it. That is when I said my husband at the hospital, I am now getting what, depo but aah there was no joy in it as someone who had gone to get the injections without him knowing.

KC: Uhh, alright, alright. Uhh she said it happens, are the others? [Sound of a moving vehicle] In general not looking at you personally. Just in general because our discussion wants general things. [Silence] [Sound of a moving vehicle] Alright, what about…

67: I want to support what has been said by 61.

KC: Uhh.

67: Yes, it happens because it's said if the family planning pills are exposed to heat, if they are exposed to high temperatures.

KC: Uhh.

67: They will not work.

KC: Uhh.

67: The…the husband can take those pills from you and expose them to the sun whilst you are working, you are not aware of that.

KC: Oooh.

67: He will leave them in the sun when you are thinking I am taking my pills correctly. Then you realize you are pregnant when you are taking the pills.

KC: Alright, I understand so where I wanted to ask is that are the adolescent girls and young women who have male partners, but their partners have other affairs with other women whilst they are married. Are they there who are in such situations, such situations do they happen in here in our community?

65: They are

KC: Those are the majority.

65: Uhh.

KC: Alright. Are there reasons? Assumptions, facts that can allow such behavior, make someone to do that, 67.

67: Reasons will be there.

KC: Yes.

67: We get married in a good way right. We have settled in well, the first year there is no child. The second one, there is no child. The third year, you start talking, the husband starts saying ahh what is happening here so he will be having extra-marital affairs, wanting to see if it will work. Not knowing that maybe he is the one with a problem, I am not the one with a problem. To tell him lets go to the hospital he will be saying no you are the one who is what, barren.

KC: Alright, 63.

63: Some of the things that happen, like you would have been married at an early age, at 16.

KC: Uhh.

63: Then you are good in your marriage, the husband doing everything for you. Sometimes you will be…sometimes you will not be having anything. Everything is what, is not available. The husband sometimes he is getting money, he is not bringing the money where, in the home. The wife cannot look after herself well, she cannot maintain herself. She is now just someone whom if she tells you that I am 18 years, you will not think she is 18. You might think she is 25 years.

KC: Alright.

63: But she is still young, she is just not getting good things in her house. The husband is now living his own life, coming home is just for two minutes but he now promiscuous where.

KC: Outside.

63: Outside, yes that happens.

KC: Alright, alright. 68.

68: Some men follow that thing to say our culture you cannot have one wife, so I am supposed to have many. Culture and religion from churches we attend.

KC: Alright so culture and at the churches that's how they may end up having many. Alright, aah what about looking at adolescent girls and young women who are married. At that time, we were discussing in general that…in general. Now we want to specify that for the married one will it be an easy decision that women…married adolescent girls to decide that I am now taking PrEP? Is it an easy decision? Is it easy to decide that ahh I am now starting to take PrEP? 65.

65: I can say its easy because the risk that you will have you are the one who knows it.

KC: Uhh.

[People talking in the background]

65: Like the husband is not being faithful, you…he does not want to get tested. If you get tested, he will tell you that your result is my result, if you are negative, he will say aah I am still alright. You are fed up with getting treated for STIs, he does not want to come and get treatment, so your risk is the one that pushes you.

KC: Uhh.

65: To take PrEP.

KC: Alright so she said its easy because you will be seeing the risk that you have so it makes it easy, you are now taking because of what you have seen. Is there anything else, is it easy? [Silence] So we are all agreeing that its easy for women to take PrEP.

65: Uhh.

KC: But there is a point that we mentioned at the beginning. [People talking in the background] When we were saying men do not support their partners to take PrEP so let us say in a marriage set-up how does it work? The wife wants to take PrEP is it possible? Will she get the support from her husband, is it possible? Wont there be conflict with her husband if she starts taking PrEP in the home, 63?

63: I think that if I want PrEP and my husband is not interested.

KC: Uhh.

63: I will try my best for me to get a day I will take my pills whilst my husband does not, does not know. For me to look after my health because if I…if he sees that I will not make any progress. He is being promiscuous, but he is coming and infecting who, me.

KC: You.

63: Saying I do not want PrEP in my house so I will look by all means, if I can even put them at the next door if she is someone I can trust. I would rather be there at seven o'clock each day or put it under the bed, there is no man…I am the one who makes the bed, I am the one who cleans. There is no way he will lift the bed, that is where I will be taking from and drinking them because if I keep on telling him, I will not be making any progress when he is the one who is bring the infection.

KC: 61.

61: I am supporting what 63 has said that we will be a three months' supply.

KC: Yes.

61: Its better to be coming here and get a month's supply, each month you come and collect.

KC: Ohh so you get it each day, coming to collect, 65.

65: From my side when I was taking PrEP, before I took it, I told my partner that I learned about this and this. At first, he said so are you saying I am being promiscuous, then I told him I never said that but its just good for me to do what, to protect myself, you never know what happens. Then he refused, then we went for about three months, before I would have some STIs. It was not easy to get the injections, I was in pain.

KC: Uhh.

65: That is when I realized my husband is not changing, I came to the hospital secretly and took PrEP. I did not tell him that I have taken, I went and hid it. Where I hid it was in a bag with baby nappies, then I saw a message in his phone saying aah moyo (a totem) you are good in bed. Aah I am now thinking that aah these people that are enjoying each other, they are not using protection.

KC: Uhh, uhh.

65: Aah with anger I packed my bags, he started saying ahh sorry babie what, what. During the argument, the bag had a loose zip the pills rolled over, and he just stood there holding the bag. Then he looked at the pills and asked what they were for. I just managed to tell him its that PrEP that I told you about, I saw myself as being at risk. From that time my pills are now staying in the open. He now reminds saying have you taken your pills XXX, then I say no I have not, I take and drink.

KC: Alright, okay, alright. Aah…aah are there others with a different view "_+"? [Silence] What about looking at…so far, the points that have been raised are that partners might difficult for someone to take PrEP. Looking in marriages is there something else that might be a barrier to it being taken by adolescent girls and young women? There is…barriers to taking PrEP? [Silence] Alright, its okay. What about on our role play right, there was an issue that was coming that why do Mrs Bhobhi now wants to stop using PrEP. Mrs Bhobhi gave an explanation that Mr Bhobhi takes the pills and throw them away, so I have thought to stop using them and just wait for the day it happens, right. Are there any other reasons that can make adolescents girls and young women

who would have started using PrEP to stop using them, they say aah it's enough, I don't want? Are there any other reasons? [Someone coughs] That will make one to stop using PrEP, not the one explained. Are there any other reasons? 61.

61: One might say all along I was not taking pills so to start taking them now, its better to stop taking the pills as I am someone who never lived taking pills.

KC: Alright, one is not used to taking pills, they want to start taking the pills now, they say its better I stop. Are there any other reasons that can make someone stop using PrEP when they have started using it? 65.

65: The other issue is…the issue of side effects. One might say aah the pills are making me vomit or nauseous then someone says I am not stopping it.

KC: Ok, alright but do they have the, do they have any side effects?

65: Uhh.

KC: Uhh which ones?

65: You can feel nauseous, vomiting or have a headache.

KC: Uhh, vomiting, headache. Is there anything else? 61.

61: Not have an appetite.

KC: Not have an appetite.

61: You will be weak, in the morning when you wake up you will be feeling hungry but for you to eat the food, you will not be able to eat it.

KC: You will not have an appetite for you to eat.

61: Yes.

KC: What can be done to encourage someone who would have started using PrEP to continue taking PrEP. For us to encourage them to keep on taking PrEP, to keep on taking PrEP "_+".

[Wind noise making it difficult to hear]

65: From my view if my friend has started taking PrEP I can take her number and regularly send her a message. Its almost time do not forget to take your medication or even call. Also telling her that if you keep on taking your chances of getting HIV will be less.

KC: Okay, is there anything else that can be done? "_+". Alright we are now going to the role play number three, with three friends. [Someone coughing] They are designing a

PrEP program that can make adolescent girls and young women want to take PrEP so role play number three, may you come.

Role play 3

Princess: Hey my friend since we have been asked to design a program for us to take PrEP, what do you think we should do?

Peppa: I don't know girls what should we do because I don't understand yet how it works this program. May you explain it to me so that I know.

Princess: This program, PrEP are pills or medication that protects us from getting HIV. Since we have partners, I am now having sexual partners and some of us have many. You don't know your partners' status, we would want to do what, to be able to protect.

Peppa: Oooh.

Princess: Yes.

Peppa: It seems like a better thing, what do you think Sky about this program since we are having many boys here at school but what I think is that since we are aware of this programe. Maybe we should educate our partners since we are still young. I don't know what do you think?

Sky: Haa I was thinking that its alright so what we should do is we look for other girls "_+". [Wind noise] Then we go to the hospital get tested, after getting tested "_+".

Peppa: Now if we take PrEP what will our mothers say? "_+".

Princess: Haa that will be a problem, is it not possible to "_+".

Peppa: But if its my mother she can look for them but if I go and put them in the bush. I will go and hide them under a stone.

Sky: In the bush it will not work because where you will put them there will be sunlight. They will be affected by the hear, they need to stay in a good temperature, don't you see that it will not work.

Peppa: No the sun does not matter, I can put them in a bush in a closed area where there will be a shade.

Princess: That will not work my dear because they say the pills its like when you are ill, isn't it they say they don't want to be exposed to the sun too much. These ones that you are now putting in the bush how will they work. I think if we have somewhere private where we can put them so that we hide them from our mothers since we are now sexually

active. Our mothers do not know yet that we are sexually active, we have to look for somewhere to hide them if they see them we can explain but we are still young.

Sky: Now having to explain its difficult, personally I...or you ladies go and collect first. When you take them, I will be making a plan, if you take them and I see that it has worked for you then I will follow suit.

Peppa: I think so.

Princess: Haa let us do it and see how it goes.

[Other participants clapping hands for the group]

KC: Thank you, thank you. Right so we now want to have a PrEP discussion right. Firstly, do you adolescent girls and young women if they have been asked to design a PrEP program are they able to a PrEP program? Are they able? [Silence] To say they have been given a chance, adolescent girls and young women do design a workshop of designing a program, are they able to design such a workshop? Is that possible?

65: Yes its possible.

KC: What about if we want to look at what things do you think are important on PrEP being taken, you have mentioned that you are using PrEP right.

65: Uhh.

KC: What do you think is important, that should not be missing in a PrEP program? Such that you say a PrEP program should have a, b, c, d for it to be successful. To be preferred by adolescent girls and young women, what is important that makes a program to be successful? We start with that one, 63.

63: I think for most people to understand it, its still a problem for them to understand that a PrEP program is good because they are thinking that if you start using it then the disease will come out.

KC: Hoo.

63: For them to see your status so maybe if there is something that makes most people…like most people like something that has been influenced by someone. He is coming with this; everyone gathers around saying today we will have this and that, so I think if they come saying today, we have such a program. Everyone who wants to let them do what, let them come, who is aged between this and that age you will see a lot of people coming

then you explain to them. You tell them how it is used; I think many people will quickly accept it if they understand.

KC: Uhh, others. What do you think makes a PrEP program to be successful? She said if people get information about PrEP many can understand and quickly accept it, is there anything else? [Silence] What makes a PrEP program not successful, the bad things that you say aah if these things are there they will not accept the program, the adolescent girls and young women they will not come to take PrEP because of this and that, 63?

63: If they keep on giving us the pills in the same room with people who have HIV, people will not agree. They will think that we are on ART treatment, they will not agree. Most people do not want because you are asked to go and get tested. Then you get there and wait in the queue, of which in that queue we will be mixed with us who want PrEP, some want pills for a stomachache, some for a headache so we will be all mixed. So, some do not want "_+", aah its difficult because of PrEP because they do not understand yet, they will say what I am benefitting from this.

KC: Okay, others. She has mentioned that as long its still combined with where ARV are given it is a problem, association people assume that you are HIV positive. Is there anything else that is bad which makes PrEP not successful? [Silence] Alright, uhmm what about looking at the last question, we are almost done. So, this one is the first part of the study which we explained that it's a study of developing a program that is acceptable to adolescent girls and young women when they will be using PrEP. So, the discussions we are having is the first part. After we are done, we will conduct a survey, our survey the way we will do it right, we look for places where we come. Where adolescent girls and young women can go to such places, those that are willing who will not feel uneasy because we will want the ones who are sexually active. Be it selling sex, having sex in marriages or relationships with boyfriends right. We want them to be comfortable at such places right, so my question is that for example in this area. Where can we go and stay maybe for five days, girls coming to such places. Which places may they feel comfortable enough to come and enroll in our study? 61.

61: I think here at the clinic is good.

KC: At the clinic, why is it good?

61: That "_+".

KC: Alright, 63.

63: I think that out there like we commute, we carry our clothes, and we go because here if we are told to come here. Everyone will know what will be happening. Some people might have a bad record of being labelled as sex workers, did you see them what they were doing in that programme. They were doing this and that so I think that going out, we commute and have five days then we come back I think it will be alright.

KC: Okay, are there others? 65.

65: Kana pacommunity hall panoita.

65: Or the community hall, it will work.

KC: At the community hall, there is a community hall here?

65: Uhh.

KC: What is done there?

65: Meetings are held there but its not all the days such that sometimes it will be free. Also down there, there aren't a lot of people, its free.

KC: Ooh there are no people who will be moving around there.

65: Yes.

KC: "_+".

XXX: "_+".

KC: Alright so aah what else do you think can be done in the study to…so that people come, they enroll in the study? For them to feel comfortable to enroll in the study, is there anything that can be done? [Silence] Is there anything that can be done by the study to make people feel comfortable to enroll in the study? [Silence] Is there anything that can be done by the study? [Silence] 67.

67: Uhh.

KC: Yes.

67: Like entertainment, when you break you have some dramas what, what and food.

KC: What type of food?

67: Any type of food that is satisfying that one…one might go away because they will be hungry.

KC: So, giving them something to eat, 63.

63: One might be interested in coming for five days there, but one might say aah I go there for five days what will I benefit. I am having a setback; I want to pay for my rent since I stay alone. I want to pay for rent, food I will be looking for it on my own so if I go for five days what I am benefitting. Aah so let me leave this alone, we might be a group so what are we benefitting, they want to use so why do not we just leave it.

KC: Alright.

63: So, I think if you say we can assist them with something as if we have listened to what…it will be understandable.

KC: Okay, alright so the ones enrolling into the study they will come like the way we did here.

KC: Its just coming and spending some hours and go back but the team, us who would have come are the ones who will be staying at a place maybe for five days or six days depending on the number needed at that place, has it been reached or not but participating in the study it will be just for some hours. You just come and participate, then go back, that is it, only for one day.

XXX: Alright.

KC: So, in the study right, later we will be asking in the same group to self-collect some samples, samples to test for some STIs. They will be self-collecting in the vagina right, that's where I want to ask that do you think that is acceptable? Will they want, adolescent girls and young women to self-collect because usually the nurses collect the samples that are tested for STIs. Will they want to collect on their own, self-collecting the sample, 65?

65: Haa we would like that a lot.

KC: Uhh, why?

65: Because I am not going to open my legs to someone. Sometimes when I open them, I am told some hard words being said aah is that how a woman's genitals should look like so even if I have too much pubic hair I will self-collect.

KC: Alright, uhh. Are there others with a different view? [Others giggling] So all of you do you think the adolescent girls and young women might prefer this?

All: Uhh.

KC: Aah alright, with the same reasons?

All: Uhh.

KC: Alright, its okay. So, the…these samples will be tested for STIs. After testing the STI results, where I wanted to ask is that how do the adolescents who would have self-collected their results receive the results? Their results, how do they receive their results, how do we communicate them? 63.

63: Aah I think when you take them, if you cannot come you collect each person's phone number. Then you tell me my results rather than publishing for everyone to see.

KC: Alright so telling each person on their own.

63: Uhh.

KC: If we cannot come back then we do it over the phone.

63: Uhh.

KC: Others, 61.

61: Or here there is sister XXX, you can just send to sister XXX then she just puts them in my card, then I see them in my card. [Others not agreeing with her point]

KC: She is the one who gets the results from this area?

XXX: Aah that is not proper.

61: Only mine, I am the one who would want them to be delivered to her.

KC: Oooh.

65: Only for yours if you are comfortable.

63: For me over the phone is okay.

XXX: "_+".

KC: They bring them back in person.

65: Uhh.

KC: Alright so most of you are saying coming back with the results in person, you tell me the results, or you tell me the results are at this point.

XXX: How they should be processed.

KC: Alright, alright. Aah let us say for example "_" when we do the survey, we will collect your phone numbers and notify you that they results are out. The results have been delivered at such a place; will this work let's say the results have been delivered here. Each person coming to collect their results here, does that work?

63: Aah as for me it will not work.

KC: It will not work.

63: How will I get treatment, what if I…I have gone to collect, after collecting I realize I am positive. How will I get treatment?

KC: That is where we want to ask, that's where my next question is. Let's say one has tested positive, one is supposed to get treatment, every STI has to get treated.

63: So, at the hospital I think it will be alright because that's where the medication is available.

KC: Alright so here which places would you recommend for STI treatment?

63: At the hospital.

65: At the clinic.

KC: At the clinic, hospital. Alright, it's okay. Our last question, there are those forms that we mentioned. The injection, the ring for PrEP, these are being called long-acting formulations. They have a long dispensing frequency; they are different from the pills where one need to them on every day.

XXX: Uhh.

KC: Its inserted and you stay for a long time with them, then its changed and have another one so as I have mentioned before they are not yet available in Zimbabwe. To say we now have them, and people are using them but maybe later the ring will be available. Maybe soon the injection we will have it as well. My question that I want to ask is that what are your views on the injection, the ring? Are they things which can be acceptable, will they be accepted by adolescent girls and young women, 63?

63: Yes, I think they are acceptable, if you want the injection, you then say let me have the injection. Not to take them every day, I might skip the days so let me have the injection. I will just know that its expiring on this date, I come back where…

KC: At the hospital.

63: Here.

65: Yea those types are good because let's say it's a woman who is in a situation where her pills are burned or thrown away. If I get the injection, I will be safe, no one affects it. If I have the ring inserted no one will remove it.

KC: Okay, that is how its good. Others, is there someone with a different view on the injection and ring? [Silence] That is the same.

All: Uhh.

KC: Alright, its okay.

XXX: I want to ask on the ring, will it not fall or the size… [laughs]

KC: Alright.

XXX: The size it can fall.

KC: Alright on the…the ring falling I know it will not fall because when its inserted I know it will be well positioned. It's designed in a way that it fits well, it will not fall and from the study…there is a study that has been done, I once attended their meeting. It was done I think by Pangea who were saying there are some who have used, wanting to hear their views so most people…Questions that were there were when I am having sex, with the other person not feel it, will it not move but from that study they were saying it does not affect.

XXX: Its not painful.

KC: Its not painful "_+", it just positions well.

61: What if it misses and it moves like this.

[Others laughing]

KC: Maybe we will get more information when its now being used here, right.

XXX: Aah alright.

KC: But so far the information that I read, that I heard.

63: That is the one.

KC: Haa…there is nothing that happens on it. That makes it to be positioned well, being "_". Are there any other who have a question before we are done? [Silence] None.

Some: Uhh.

KC: Alright, if there are none, the questions that we had are done. Thank you for your time, thank you for your responses. Thank you for coming and for the discussion that we had, we have come to the end now.

61: Thank you for coming.

The End
