# Supplementary material for: Perceptions of pre-exposure prophylaxis among sexually active adolescent girls and young women in Zimbabwe–A qualitative study
Source: PLOS Glob Public Health. 2025 Dec 2;5(12):e0005396. doi: 10.1371/journal.pgph.0005396 (PMC12671731; doi:10.1371/journal.pgph.0005396)
Supplement: S1 File — (ZIP) [file pgph.0005396.s003.zip › S1_File/AGYW-FGD 08- Translation.pdf]

AGYW-FGD 08- Translation
Facilitator: LM
Note Taker: KC
Date Of FGD: 22/03/2023
Age group: 16-19 years
Translator: SM

Thank you for taking your time to have this discussion with us. If you still remember my name is Lindiwe and my work mate is Kudzai. Today we are going to discuss your views on PrEP, which is called Pre-Exposure Prophylaxis, (someone coughing) (baby making noise) for us to understand its reception, reasons why they are a few people using PrEP, and the issue on how it is being received by adolescent girls and young women. Ehh this is going to give us important information on PrEP programs to be designed, so that we increase the number of adolescent girls and young women in Zimbabwe who are using PrEP, and those who are willing to use it. Our discussion will take about 1 hour 30 minutes to 2 hours for us to finish. Ahh in this discussion we will have a discussion and role plays. Ahh when we are doing the role plays, we will ask you to be in groups of 3. Each group will do a play on how adolescent girls and young women will respond to different situations about PrEP and their views on risky behaviours that can lead someone to contracting the HIV virus. Each group will be given a scenario and it will be explained to them and they will be given 3 minutes to discuss what they are going to act. We will ask that groups give each other time to act their play. This will be followed by a discussion to discuss the play that was done. Before we start this session, we would like to have ground rules that will make us have a meaningful discussion. So here we are talking about rules, right?

Mmmhh

Ehh what do you want, what do you want us to talk about? What are our rules? (silence) Which ones? My first one is that everyone now has a new name.

ALL: Mmmmhh.

Yes, our numbers are our names. Let us not call each other with our home names. I know we know each other so it becomes easy to say ahh Zanele is saying, or what was said by Lesedi or so and so things like that.

ALL: Mmmmhh.

LM: So, we are now using our new names the numbers like saying as 77 said, as 73 said. Yes so, another rule that we can have on those who have phones they should. Yes, they are saying phones on silent.

XXX: And there is no wrong answer.

LM: Yes. There is no wrong answer, right?

ALL: Yes.

LM: All your answers are excellent, they have stars before you start talking.

XXX: If you want to answer a question do not speak to yourself, raise up your hand and they will pick you and speak loudly so that we can all hear you.

LM: Yes, what are others saying? (Baby crying) Rules that you can say? If someone wants to go to the toilet you do not say miss miss (laughing) you just leave and come back and sit down, right?

XXX: Yes.

LM: What are the other rules? Are those the only ones you have? I will also ask that we speak loudly right, so that we all hear each other since we are a group. So alright. Like our agreement we are not going to call each other with our names during this discussion but we will use the numbers that we gave you. Those who are going to act, they will use the names provided on their plays. The words that are going to be recorded are not going to be used with your names but will be written the number that will be allocated the group. Do you have any questions before we start? (Baby making sounds) You don't have any questions? Alright. We can now start our discussion, right? Yes. Have you ever heard of PrEP? That we call PrEP, Pre-Exposure Prophylaxis?

XXX: Yes.

LM: You can talk number 77.

77: I have heard about PrEP but l have never gotten information about it.

LM: What did you hear? Where did you hear that?

77: I heard them talking that nowadays there is PrEP (baby noise) that you take protecting yourself from the HIV/AIDS virus.

LM: Mmmhh

77: So, l had not yet received information on how you go about PrEP

LM: Mmmhh

77: I need to know.

LM: Alright where did you hear about it?

77: From people.

LM: From people talking.

77: Yes.

LM: Alright. What about others, can you tell me have you ever had about PrEP? Yes number 71.

71: I have learnt about PrEP from DREAMS CeSHHAR.

LM: Mmmmhh

71: PrEP are pills that you take every day at the same time.

LM: Mmmmhh

71: They protect against the disease in case you get raped, it helps to not contract the virus. (Baby sounds)

LM: Mmmhh thank you. Ehh are there others who have heard about PrEP? Or those who think they have heard about it, or who have questions about PrEP? Are they there? (silence) No one?

XXX: Can l ask?

LM: Yes.

XXX: That this PrEP when you take it, are you drinking it for the rest of your life or what?

LM: Okay. Is there anyone who wants to respond? Mmmhh do not be scared. Alright so l will explain to you what PrEP is right. Yes 71 had explained and said PrEP are pills that you drink when you are HIV negative to avoid contracting the HIV virus when you see that you are in danger of being infected. Ehh this PrEP can be pills, can be an injection or vaginal ring that you can use, and you are supposed to take it for the rest of your life so that you do not get infected with the HIV virus. Ehh l will show you how the vaginal ring looks like. You can pass around this paper, and you can also pass this side. So, this vaginal ring will be inserted in a woman's vagina and goes to stay at the cervix. It is the one that protects you from being infected. There will be some medicine inside. Ehh this one for now l do not know if you have seen it?

ALL: No.

LM: You have never seen it?

ALL: Yes.

LM: Alright. Then these are the PrEP pills, that are currently available and being used right. (Sound of pills in a container) If you open inside you will see them. If you open inside you will see the pill (sound of pills in a container) they will be like this. Do you see

them? You can pass around. You can hold the bottle and see them then pass it to others. So, these pills you drink them every day at the same time. (Baby noise and coughing). So, have we seen them?

ALL: Yes.

LM: Alright. Ehh so what do you think about these PrEP pills? You have seen the pills, what do you think about them? Let us hear your views. 75 what do you think about this pill?

75: (Inaudible).

LM: What?

75: It is okay because it works. It protects you from getting infected with the virus.

LM: Alright she said it protects you from being infected with the virus. Someone else? What did you think about just seeing it? By seeing maybe, the container, what did you think? 78. Just seeing the pill. (silence) Okay 72, what did you think when you saw the pill? How is it?

72: Its big. (Everyone laughs)

LM: She said its big (chuckles) is that what you all wanted to say? She said its big, what about others then? 70.

70: If it can protect from HIV, we can use it.

LM: What about others, what are you saying? (silence) Ehh what about the ring? The ring paper what does it say? (Everyone laughs)

XXX: Ahh the ring…

LM: You can talk 71 let's hear.

71: No no the ring no (laughs)

LM: How is it?

71: It is painful for me.

LM: It is painful for you?

71: Yes.

LM: 77 what are you saying?

77: Ahh l also wanted to, for me the ring no, l would use PrEP…

XXX: The pills.

77: I would use the pills not the ring…

LM: Because of what?

77: Ahh being poked

LM: Background noise from participants) You do not want to be poked?

XXX: So, this ring, you will stay with it for the rest of your life?

LM: No, you stay with the ring for a month, then you go and have it removed and put a new one.

XXX: Okay. (Background noise)

XXX2: If you do not feel it that it is there while you have it, then you can use it just because you can forget to drink pills.

LM: Mmmhh alright. So, you can forget to drink the pills but if the ring is inserted, it will help you. What about others. What do you say?

XXX: The injection is better.

LM: Mmmhh. How is the injection?

XXX How long is it?

LM: We do not have it yet. It is not there yet but it is something that will be there soon. So, you asked about the injection, how do you see it?

XXX: I think it will be best because with the pills l can forget the time to drink them and l fear the ring.

LM: With the ring are you scared of being poked? Okay what about others? What do we think about the ring or pills? Your views, looking at what you think? 81 you saw the ring and you saw the pill.

81: I think the pills are better.

LM: Why do you think they are better? (silence) With what reason? (Baby noise) They are better in what way?

81: Because you will just take them and it passes.

LM: Okay. 70.

70: No l think the ring is better, just because you cannot go to town if you are going to stay there for longer days carrying pills.

LM: Okay (baby crying) what about others? Alright if we do not have anything to say, we can now go to our play, right? Yes, so l will ask that you go into three groups. I will give each group their play and you will discuss it before acting. So, we have our three plays right. So, l will ask that you have three groups. We will be in fours right. Yes, so

this group will take the first play of Chido and Koko, the four of you. So, l will first read it and then give it to you then read the second one and give you. Read the third one and give you. Then we will break while you practice. Then we will come back again and gather then you start acting in front of everyone right.

ALL: Yes.

LM: So the first play is called Chido and Koko. Chido is 16 years old and has a sexual relationship with a 50-year-old man. She recently started taking PrEP because she is worried about getting infected with the HIV virus. She thinks her friend Koko who is 19 years old is also at risk of getting HIV by dating people and having sexual relationships with them, so she is encouraging Koko to also start taking PrEP. Do a play on Chido and Koko, putting together what you think will happen in life if Chido comes with this plan. (Baby crying)

We now come to the three friends, Peppa, Sky and Princess. All of them are above 20 years and are having sexual relationships with their partners and they all go to the same school. They were chosen to come up with a PrEP program for adolescent girls and young women for an organisation called CeSHHAR that deals with sexual health and population. They are preparing to deliver their views. Do a play on their conversation talking about things that they think should be there in the program. So, you are going to be the three friends, right?

Yes, we now come to the last one. NakaBhobhi and NakaJuru. NaBhobhi is 23 years old, and she is married. NaJuru is 21 years old, and she is also married. NaBhobhi's husband has a tendency of having multiple sexual partners. She is worried that, NaBhobhi is worried that she might get HIV. She heard about PrEP on the radio and then she started using PrEP and it has been six months since she started using it. However, she is thinking of stopping using PrEP. Do a play about the conversation between NaBhobhi and NaJuru putting together reasons that are making NaBhobhi think about stopping using PrEP. (Baby sounds) You all heard right?

ALL: Mmmhh.

LM: So right now, I will ask that we go to our groups and gather while practicing right. Yes, when we are done practising then we come back, l will give you 3 minutes, 3 minutes to gather and practise your play then we come back right.

(Recording paused as participants are discussing their role plays)

LM: Ehh now let us discuss about our plays that we did. Let us look at the play between Chido and Koko. May you please ehh ahh do your play. (Baby crying) you can now act. (inaudible)

XXX: We were not done.

LM: You were not done?

XXX: Yes.

LM: How long do you need? Another minute? Alright.

(Recording paused)

LM: Alright can you act for us. (Background noise) No one wants to act? Just do what you did. (Baby crying) I want you to do a drama if you want to stand up you can. (Babies crying)

Role Play 1: Chido and Koko

Chido: I want to have sex with a man who is 50 years. I recently started PrEP since l am worried that l might get HIV. I also think that you my friend Koko who is 21 years old, you are also at risk of getting HIV because of having sexual relationships (baby crying) so l encourage you to take PrEP.

LM: Do the play let us hear how your conversation. How will Koko respond when you tell her about that? (Background noise)

Koko: I am Koko l agree with Chido that l should also take PrEP pills so that l protect myself from the HIV virus.

LM: Are you done?

XXX: Yes

LM: Alright. Let us clap hands for them. (Participants clapping hands) Alright ahh looking, do you think people like Koko are there, like Chido who can have sexual relationship with old people? I am asking you who were acting. (Baby noise)

XXX: Yes, there are there.

LM: On what reasons, can you explain for me? (inaudible) (baby noise). Can we all come together. Do you think Koko will agree to take PrEP by being told by her friend? (Baby noise) What did you say?

XXX: I said yes.

LM: Can you please explain to me?

XXX: Because she also thinks it is a good idea to protect against HIV.

LM: She also thinks it is a good idea to protect against HIV. Alright what else? Do you really think Koko will agree? Or looking at all of us generally, are there people like Chido who will be in love or have sexual relationships with old man who are 50 years? (Baby noise) Mmmhh 77.

77: Yes, it happens, that us who have younger years (baby crying) to date old man and have sexual intercourse with them because some say old man can take care of you if you date them but young man will play you.

LM: Alright (baby crying) What about others? (Baby crying) old man can take care of you, what about others? (Baby crying) number 70. Can you move around with the baby outside? Please can you move around outside with the baby. 70 you wanted to say something, what did you want to say? What about others do you think there are people like Koko in life? Like Chido who fall in love with old man. You said they take care of you while young man will play you. What about others, what do you say? 75.

75: Yes, they are there. Some just wants money (chuckles) from the old man.

LM: What?

75: They will be saying old man have money.

LM: Alright okay. She said some will be saying old man have money. What about others, what are we saying? 73.

73: Yes, they are there.

LM: Yes.

73: They will be knowing that they are going to take care of them till they reach their age.

LM: Mmmhh she said they can look after you. What about others what are we saying? 78 are there girls like Chido young, young girls who fall in love with man older than them? (Baby sounds) (silence) Okay. What do you think? Chido is at risk of getting HIV. Do you think she is at risk of getting HIV because she is dating an older man?

70: Yes, she is at risk if she is using the pills because she might forget to drink the pills then get HIV.

LM: Mmmhh alright. So she is at risk, its 70 who was talking. So, she is at risk of getting HIV if she is taking pills and she forgets to take them. So here you are saying that there are other ways that can help her not get HIV if she is at risk? (Baby crying) Let us talk. Others, do we think Chido is at risk of getting HIV? Chido is the one dating a 50-year-old man. Do you think she is at risk? Alright is it common for adolescent girls and young women to share health decisions with other people? Do you think in your community or other areas (baby crying) do you think adolescent girls and young women can share information or they can talk about health issues with other people? Are we together?

ALL: Yes.

LM: Do we understand the question? Did we understand? (Baby noise) 81. Do young woman tell each other about health issues, or you here have you talked with each other that ahh life is like this or about getting health care.

XXX: Yes.

LM: Number ahh, what do they tell each other? Things like what? What do they talk about?

XXX: That we should prevent.

LM: In general, what do you talk about as young people in this community if it is about health issues that is what we are looking at? (Baby sounds) Have you ever discussed. Do you only ask your friends, or you go and ask your mothers, aunts or do you ask from the hospital? What have you done? You have never asked about health issues? (silence) Mmmhh never ever? Alright let us hear it. What have you asked? What have you asked each other as young women? You spoke about prevention. Can you tell us more about prevention so that we look at that?

XXX: Prevention so that we do not get the virus.

LM: Okay so how will you be discussing? Mmmhh there is no wrong answer. Ehh 75 have you ever discussed about health issues and who do you ask about health issues? Like l have asked my sister about where family planning pills can be found. Who do you ask about health issues as young people?

XXX: Health workers.

LM: What 72.

72: Health workers.

LM: You ask health workers?

72: Yes.

LM: Yes, 72 said health workers. What about others, who do you ask? 70… its 70 who?

XXX: 79.

LM: 79 who do you ask?

79: Friends

LM: You have asked friends?

79: Yes.

LM: Okay. This one said she asks friends. What about 70, who do you ask?

70: We have asked each other as friends.

LM: You have asked each other as friends only? Alright what about others? 73

73: Sisters.

LM: Sisters, she said sisters. Ahh alright so what about information on PrEP? Where do you get it? Some of you said you have heard about PrEP from people talking. Ehh where do you get information about PrEP? Mmmhh where do we get it?

XXX: From the hospital.

LM: She is saying from the hospital. 71 what did you say?

71: There are chosen members from the community and also from the people of CeSHHAR.

LM: Alright can you please explain. Do they go around talking? Can you explain how they give information on PrEP?

71: There will be visitors who would have come to teach us about PrEP on how we use it to protect….

LM: Mmmhh.

71: How we use it to protect ourselves in sex.

LM: Alright thank you. We have heard that there are visitors who come working together with others from the health sector who are working in this community to teach about PrEP. Others have we heard about PrEP before you came here? Have we heard about PrEP before? (silence) Where can adolescent girls get information on PrEP? Where do you want to hear it from, where do you want to get it? Or others who are not here, where do you think they can get information on PrEP. Mmmhh that what you want to say 77.

77: Since we have learnt about it, we can now discuss it with them.

LM: She said she has learnt about it so they will discuss. What about others, where can adolescent girls and young woman get information on PrEP? Where can they get PrEP information? (Baby noise) Mmmhh 75 where can others hear about PrEP?

75: At DREAMS

LM: What?

75: At DREAMS.

LM: Do you have it this side?

75: Mmmhh.

LM: Have you heard about it? Do you attend it?

75: Yes.

LM: Okay what do they teach? About prevention, STDs, diseases and how you can help yourself. Alright. What about others, how can it be heard? How can people access information on PrEP? We now know what PrEP is?

ALL: Mmmhh

LM: Yes, so how can others know about it? Looking at even those that you will not be able to reach, looking at those who are in other villages far from here. How do you think they will get the information on PrEP? 73. (Silence) You do not know?

73: Yes.

LM: What about others, where do you think they will get it? Don't you have radios?

XXX: Even WhatsApp.

LM: Yes, you are now starting that is what l am looking for. You said where?

XXX: On WhatsApp groups.

LM: Yes, WhatsApp groups. What about others, where can they get it?

XXX: From the TV.

LM: She said the TV, who said that? Say your numbers please do not forget the numbers.

XXX: 81.

LM: Yes 81 on TV, what about others, where can you get the information?

XXX: Newspaper.

LM: Yes newspaper. Those the answers that l am looking for, very easy. What about others, where can we get the information? So, moving forward, what do you think about

Koko's response when Chido told her about using PrEP? What do you think is the reason that made her to respond that way? We saw Koko saying yes Chido l also want to go get PrEP. Yes, what do you think about that response that she gave? (silence) what do you think about it? Do you think it's something that's going to happen? Do you think Koko will go and get PrEP? Do you think she is just responding so that they stop talking about it?

XXX: Mina…

I…

LM: 77.

77: I think she will go and take the PrEP and protect herself because her friend is drinking it. (Baby noise)

LM: Okay alright. 77 thinks that she will go and take it because her friend is also drinking it. Others what do we think about this response? Do you think she responded truthfully when she said she will go and take it or she will have other plans? Let us discuss.

XXX: I think she responded with all her heart because she loves her friend, and she wants her to be protected always.

LM: Mmmhh which number is this?

XXX: Number 71.

LM: 71 yes what about others? Here we are looking at Chido and Koko's conversation, we see that Koko easily agreed. She did not even refuse or "say ahh Chido do you really want me to go and take PrEP." She quickly said l will go and take PrEP. What do you think, what do you think about her response? Mmmhh who is this? 75.

75: I think she just agreed so that her friend will not be talking too much about it.

LM: Mmmhh alright. She said she thinks she just agreed so that her friend will stop talking too much. What about others, what do you think? Looking at other responses that can be given by someone like Koko in life, what are they like? We are saying in life, Chido has gone to her friend, what other responses do you think can be made? We see here that she quickly agreed, what other responses were you expecting? (Baby noise) that you think were going to be made here? Mmmhh.

XXX: I think Koko agreed but in her heart, she thought Chido was saying the person she is dating has HIV.

LM: She is saying maybe Chido's partner has HIV that is why she wants her friend to also go and take PrEP. What about others, 70? How do you expect other responses given by people like Koko to be like? What would they say if approached by Chido?

XXX: She can refuse because she will not have any proof that she is doing that think.

LM: Okay.

XXX: If she is protecting herself or not.

LM: Or not. Alright. What about others, what are we thinking of? 71.

71: I think she could have agreed because she had explained how the pills work.

LM: Okay she is saying she had explained about the pills. Others there? What will others say seeing seeing Chido telling her to go and take PrEP because she is also taking it. (Baby sound) If it was you, how were you going to respond? 80 how would you respond? (Baby sounds) Mmmhh? Let us say l am Chido and l say l wanted you to go and take PrEP what would you say? (Baby noise) (inaudible) Truthfully? (Baby noise)

XXX: No.

LM: Yes (chuckles) so give me the right response that you would give your friend truthfully. That one that you are thinking of? (Baby sounds and noise) the one that you are thinking of. (silence) Alright umm what do you think is good about using PrEP, being used by adolescent girls and young women? What is good about using PrEP?

XXX: It protects that even if you meet with someone who has the virus you will not get it?

LM: Yes 72 is saying it protects even if you meet with someone who has the virus you will not get it. What about others, what is good about using PrEP? (baby noise) 71 what are you saying?

71: Even if you get forced to have sex by someone who has the virus if you are taking the pills, you will be protected.

LM: Yes, even if you are forced to have sex, she said you will be protected. What about others, what are saying? What is good about PrEP? 80 what are you saying? What is good about PrEP? (Baby sounds) Mmmhh.

80: It is that even if you are in the middle of your journey and you get raped you will be protected.

LM: What?

80: Even if you are in the middle of your journey and you get raped you will be protected. (Participants talking to themselves)

LM: Even if what? Sorry can you please raise your voice a bit. Mmmhh.

80: Even if you get raped by someone you do not know by force.

LM: Alright. Yes, that is another answer. What is good about, about taking PrEP? 75.

75: Because you will not get the virus.

LM: Yes. Alright it is okay. Ehh looking back, is there anything that we did not talk about that is disturbing the use of PrEP by adolescent girls? What is making adolescent girls to not take PrEP? What is making them to not use PrEP the adolescent girls? What is making them not use it? 70.

70: Some will be maybe positive and see that it is best to not use it.

LM: She said some will be already positive and see no use in taking it. What about others, what are we saying? What will stop people from using PrEP? 73.

73: I think she will be knowing already that, she is positive.

LM: Okay. What about others? What is making others not use it?

XXX: Some will be having doubts that it will not help them.

LM: She said some will be having doubts that it will not help them. What will be the reason for thinking like that?

XXX: She will be saying she does not have the virus so there is no need for the pills.

LM: Alright she is saying she will not be at risk of getting HIV right. Yes, others? What will make, 81 others not to use it? (silence) What will make, what is stopping you from using it?

81: They will be seeing that they will forget, especially the pills.

LM: She is saying they will be seeing that they will forget. 75 you wanted to…

75: They will be influenced by friends who are already positive to not use it.

LM: Huh?

75: They will be influenced by friends who are already positive to not take them so that they die together.

LM: Yes, that's another point, she is saying she will be influenced by her friend, the friend is now positive so she will say don't use this so that they will be both positive rights. Ahh what are others saying? (Baby sounds) Alright l can say ahh is there anything else

that encourages people to use PrEP? What makes people want to use PrEP? What will encourage? What will make them to say, no we want to use PrEP, what would have encouraged them? What pushed them to use it? (Baby crying) Alright so where can adolescent girls and young women, l can say your age, get PrEP? Where can PrEP be found? Mmmhh (silence) Where can it be found? Let us think of places where they can get PrEP? Who is this one? 81.

81: At the clinic.

LM: She said the clinic. What about others, where can it be found? 71 where can PrEP be found? (silence) Okay. What about others, where can it be found?

XXX: At DREAMS.

LM: Ehh 71 and 70 ehh this one is 70 what by the way?

XXX: 72.

LM: Ehh 71 and 72 said it can be found at DREAMS. What about others, where can it be found? (silence) Ehh so what do you think about PrEP being given at the clinic? There are those who said it is found at the clinic, all of us what do we think about it being found at the clinic? (participant talking to the baby in the background) 70 what do you think about PrEP being found at the clinic? (silence) Mmmhh, is it a good place, is it not a fit place? What do you think about it? 71 what are you saying?

71: Because the clinic is where we get all our help, and the people who give us PrEP work hand in hand with the clinic.

LM: Okay what about others? What do you think about the availability of PrEP at the clinic? Is it good or not?

XXX: It is good.

LM: 77 is say it is good. Can you explain to me why you are saying its good? (silence) How is it? (Baby crying)

77: (baby crying) It is a place where we get help from (baby crying)

LM: Its where we get help every day.

77: Mmmhh.

LM: Okay. Ahh alright that is it (baby crying) ahh alright so what about DREAMS? What do you think if you were to get PrEP from DREAMS? Is it a good place? (silence) Alright so, you wanted to say something 75.

75: DREAMS is okay but they do not come always.

LM: Mmmhh.

75: So the pills will be finished without them coming and you will stay without.

LM: Alright.

XXX: I wanted to ask that PrEP, as at DREAMS they will say they want 15-24, girls between 15-24 so where will others get it? Those who are 24 and above? How will they get the PrEP since they will not be going to DREAMS?

LM: That is what l am asking, that where can adolescent girls and young women get PrEP?

XXX: At the clinic.

LM: Yes, she said the clinic. How good is it from getting it from the clinic? Is there anything that will stop people from going to get it from the clinic? So that is what we want to know so that if it comes, we know that adolescent girls want PrEP to be at the clinic, and they want this, and this is what they do not appreciate. So, clinic and DREAMS are the only 2 places that have been mentioned. Is there anything else that we want to talk about, about these places? So, we can now move forward and look at NaBhobhi and NaJuru's play. You can stand up and act your play.

Role Play 2:

(Shuffling in the background)

LM: You can come near. (Baby sounds) (participants chatting on the background)

XXX: I was taking the pills so l have decided to… (other participants laughing)

LM: Where did you meet? We want to see some action.

NaBhobhi: NaJuru l was taking pills so l have decided to stop.

NaJuru: No do not stop the pills, pills help.

LM: I cannot hear you. Can you please speak louder the recorder will not hear you?

(Someone coughs)

NaBhobhi: NaJuru l was taking pills so l have decided to stop.

NaJuru: You were taking pills for what? Do not stop.

NaBhobhi: I was taking PrEP pills.

NaJuru: Do not stop PrEP it helps.

NaBhobhi: *Ahh me drinking PrEP for what (chuckles) (inaudible) can l drink these pills (baby crying) and the ring also it is not good. (Baby crying)*

XXX: *I can hear you talking about the issue of pills. I am going around teaching about CeSHHAR and these PrEP pills. These pills (baby crying) you were not supposed to stop them and continue using them because they protect you from getting the HIV virus. It is important NaBhobhi that you do not stop these pills continue using them as you are saying your husband is acting up.*

NaJuru: *You hear NaBhobhi do not stop the pills.*

LM: We want the reasons that are making NaBhobhi to say she wants to stop the pills.

NaJuru: *Tell us why the reason why you do not want to drink the pills*

NaBhobhi: *The reason that makes me want to stop the pills is that sometimes l forget*

XXX: *Okay since you are forgetful take the ring or the injection.*

NaJuru: *Since you are forgetful take the ring and the injection.*

LM: *(Sound of a recorder) you tend to speak lowly.*

NaJuru: *Since you forget to drink the pills NaBhobhi take the injection and the ring or just set an alarm.*

NaBhobhi: *Okay l will do that.*

NaJuru: *It is okay then.*

LM: *Are you done?*

NaJuru and NaBhobhi: *Yes.*

LM: Alright lets us clap hands for them. (Clapping of hands). NaBhobhi's group ehh, so what do you think about the play? In life are there any adolescent girls and young women who are in the same situation with NaBhobhi of drinking the pills because their husband loves a lot of women? Can you please explain to me? Are there and young women who go through this?

XXX: Yes.

LM: Can you explain? What do you think will be going on? Do you think it will be easy (clears throat) a woman just like NaBhobhi to start using PrEP? This is a married woman, she is a young woman but married. Do you think it is easy for her to use PrEP, to just start using PrEP?

71: It is hard because she is married, she first has to agree with her husband.

LM: 71 says it is hard because she is married, and she has to communicate with her husband first.

77: And her husband will ask what type of pills she is drinking every day meaning she now has a virus.

LM: 77 is saying her husband will say she now has the virus why is she drinking pills. What about others?

XXX: The woman will refuse because she will be thinking she already has the virus, and she wants them to drink together.

LM: Mmmhh what about others? Do you think it is easy for NaBhobhi, for a woman like NaBhobhi to start taking PrEP? (silence) So what else can, we can say that will encourage her to start taking PrEP? What can encourage to the point that she will say l want to take PrEP? 75. What can make NaBhobhi to say l want to take PrEP?

XXX: If she gets someone who encourages her.

LM: When she has the virus?

XXX: Encourages her.

LM: Okay. Mmmhh 77 what do you say?

77: No l also wanted to say she would have found someone who encourages her.

LM: Mmmhh. What about others? (silence) Okay. So, we are saying someone will be encouraged if she finds someone who encourages her, who warns her. Alright. Does it happen in life that adolescent girls and young women will want to stop using PrEP when they have started it? That when someone has started PrEP and they stop it? (Baby sounds) what would make someone stop PrEP when they have already started it?

XXX: Because she will not have found anywhere else to get it.

LM: May you please raise your voice, l did not hear you.

71: She will not have found any place where she can take it.

LM: Alright. (Empty bottle drink falling on the floor) the place that she was taking from before what would have happened to it? Can you explain.

71: Where she was taking it all the time, the people are no longer coming…

LM: Yes.

71: They used to come to our community.

LM: They used to come here?

71: Yes they would say that, each and every month they would come… (baby crying) (inaudible)

LM: Alright. What about others? What will make someone to stop taking PrEP when they were taking it? Yes 75.

75: Her friends will have influenced her to stop.

LM: She said she would be influenced by friends to stop. What about others? What would make someone to choose to stop PrEP when she had started?

XXX: Some would have found, she would be HIV positive. Like maybe she would have come when her pills were finished, or she would have forgotten to drink them.

LM: Mmmhh

XXX: She would have gotten HIV by that time when she was not drinking.

LM: Mmmhh. When she did not drink. So, this will make someone to stop because she would have gotten HIV, right? Alright from your own view, what do you think should be done to help adolescent girls and young women who use PrEP to continue using it? (Baby noise) What can be done to help adolescent girls to continue taking PrEP? Taking it and using it? (Baby noise) Mmmhh 71 what do you say?

71: We can do workshops where we will discuss about PrEP.

LM: Mmmhh do workshops. Where can they be done, in what places?

71: In secure places like this one, secure places like the clinic.

LM: Yes. 77 you said like where?

77: Schools.

LM: She said school. Schools…

77: At the hall.

LM: Alright. Do you think it will encourage those taking PrEP to continue taking it?

ALL: Yes.

LM: Alright. What about others? 75 you wanted to say what can make them to continue taking it. Alright if there is nothing else, let us look at the third play. Ehh may you please do your play then we move forward.

Role Play 3

AGYW-FGD 08- Translation
Facilitator: LM
Note Taker: KC
Date Of FGD: 22/03/2023
Age group: 16-19 years
Translator: SM

(Paper shuffling)

XXX: *We will do our play for 2 minutes*

XXX: *Princess did you hear about PrEP pills?*

Princess: *Yes, l have heard about it but l don't know how they are used.*

XXX: *There are PrEP pills that protect you from getting the HIV virus. That you will not get it. If you take them for the rest of your life you will not get this disease and even if you meet with someone who have the disease while drinking PrEP pills you will be protected.*

Princess: *PrEP pills are right because you can sleep with someone who has the virus without them telling you and you will get the virus. PrEP pills help you from getting the virus.*

LM: Ehh what about the program? We now want the program that you are presenting. That you have chosen to come up with PrEP program for adolescent girls and young women for the organisation called CeSHHAR. So here you are preparing to present the PrEP program that you have come up with. So here you will be doing your discussion looking at the things that you think should be included in this PrEP program. That this PrEP should be like this and that. (Chatting on the background) It is written there. (Baby noise) (participants chatting on background) Alright is there anything that you would like to add, or we should move forward? Alright let us move forward. Ehh so what are the important things that will make this PrEP program a success? What do you think will make PrEP a success? (silence) Nothing? Nothing? What can make this PrEP program to fail? (silence) Anything that you are thinking of.

XXX: Those who give us PrEP will stop donating to us the pills, ring, and injection because they will be seeing that people, adolescent between 15-24 are not coming in their numbers, they are not liking it…

LM: Mmmhh.

XXX: That can make it to be not successful.

LM: Not go well, not move forward. Yes, what about others? (Baby sounds) Or the programs that are currently available giving PrEP, what makes them not successful?

XXX: It is that people started taking and then they are stopping.

LM: Mmmhh. Stopping because they are no longer coming?

XXX: Yes.

LM: Alright. So, this PrEP ehh you once spoke about places where it can be found, clinic or DREAMS. Are there any other places where you want it to be found? Not yours here, that l am saying you are the ones who should take it, l am just saying you giving us advice that if we were to go in Masvingo we will want that, we will be knowledgeable on what young people like. Do not think we now want you to go and que outside so you can get PrEP. No, we will not give you PrEP. (chuckles) Mmmhh we are now looking at the person who gives it, you want PrEP to be given by who?

70: By a woman.

LM: 70 says by a woman. What about others? Who should give PrEP?

81: By a woman

LM: It has been said. (Baby noise) Who is like?

81: A woman who can keep your privacy not someone who will go around telling everyone. Someone who will go around telling that l was going to take PrEP, then l will look like a bad person.

LM: Yes. What about others? 73 should it be given?

73: That is what l also wanted to say.

LM: Alright. Mmmhh. Ahh others, who do you want to give out PrEP? So, you want a woman who can keep your privacy? Ehh okay looking at age, how old do you want her to be? (silence) So alright, about provision of other services, looking at PrEP right, should it be given at a clinic that gives other services like here that they also give family planning?

XXX: Mmmhh.

LM: Why do you want it to be given there? (Baby crying)

XXX: Because (someone patting a crying baby) so that it will be a woman so that you will be able to take the pills. (Baby crying)

LM: What did you say, sorry l cannot hear properly?

XXX: She will not go around spreading that you are drinking PrEP pills.

LM: Yes. 70 you wanted to say something.

70: I was saying it is good. It will be easy to those who want to take their family planning, get the depo shot, they will do everything at once.

LM: Mmmhh. She is saying she would mix everything, if she wanted to take family planning, she will then mix and get her PrEP right. Yes, ehh what else? Or where the pregnant woman goes, what do you think about that place? (Baby crying) (silence) ehh looking at the role of male sexual partners, what is their role in taking PrEP? What is the role of men in PrEP? What is their role? What will they be doing? (silence) Nothing? Alright ehh so we are planning on moving forward with the survey of adolescent girls and young women who are having sex so that we see how we can come up with new ways to give PrEP programs to young people.

ALL: Yes.

LM: Are we all hearing? (chuckles) I now see people looking outside. Alright. Ahh where can we find young girls who are having sex? (People talking outside) (silence) we can call it being sexually active, where can we find them? If we want to do our survey, where can we find them? (silence) where are young girls who are having sex found so that we can do our survey of PrEP? 75. (someone clears throat) Where can we find them? A place where we can find them.

XXX: In their communities.

LM: She is saying in their communities. Where will we go? We do not know your place. I only know Madabe clinic. So, if l was to come here looking for girls that, where we can do a PrEP program ehh where can l find them? Where will l go? (People talking outside) (silence) Where did we find you?

XXX: (inaudible)

LM: laughs) Okay others place. 75 you want to say something.

75: It will be announced that they should meet at the hall or clinic.

LM: At hall or clinic alright. Ahh so do you think young girls ehh will come to the awareness that needs young people here? Do you think they will come if we ask them to?

XXX: Yes.

LM: What will make them come?

XXX: Because they will want to come and hear what is being taught.

LM: They will want to hear what is being taught. 73. What about others? (silence) Alright so as part of this awareness we would like to test for Sexual Transmitted Infections that we call STIs, you have heard about them, right?

ALL: Yes

LM: Ehh so this will include adolescent girls and young women taking samples from their private parts where we will test them for STIs viruses right? Ahh, do you think these girls will be comfortable to take these samples in their private parts, taking them by themselves? Do you think they will be comfortable? (silence) Do you think they will be comfortable?

70: Yes, some can be comfortable because they know what you will get you will be the only one knowing about it.

LM: Ehh she is saying what you will find is for you only by 70. What about others what do we say? Do you think they will be comfortable to take their samples on their own then they will be taken to be tested for STIs? (Baby sounds) Or what are you encouraging so that it will be simple for them to take the samples alone? (Baby sounds) (silence) So we will give results after testing and helping with treatment for those who would be found positive for STIs. The results should be given health workers who are trained to do their job. What are you suggesting on how we could reach adolescent girls and young women who would have been tested to get their what do you call? To get their results. Or where they can be treated if it happens that their results are positive? What are you suggesting? 81 you want to say something? (silence) Alright ahh okay. Nothing? So, Zimbabwe just allowed the use of Dapirivine which we can call vaginal ring, we showed you the pictures. Ehh so this is a silicone ring that can be inserted by a woman in her private parts so that she would be protected against HIV for the whole month. This ring was created to give women a proper way to protect HIV for a long time. It has medicine called Dapirivine that protects against the virus, it gets released a bit by bit to lower the risk of getting HIV in women. What do you think about long term methods like this that encourages the use of PrEP by adolescent girls? So, we said this vaginal ring works for the whole month, right? What do you think about this method that works for a long period? You do not use this one every, everyday right?

ALL: Yes

LM: What do you think about it? That works once per month, you only take it once a month, but it works for the whole month. (silence) Ahh here you are supposed to have an answer. We started from here. What do you think about something that works the whole month? (Baby sounds)

XXX: It is okay because…

LM: I do not hear you.

XXX: The ring is alright because you take time, a whole month without taking pills

LM: Mmmhh. Alright that is exactly it. What about others?

70: It is okay because even if you are travelling and you get raped no worries because you will be having the ring.

LM: Mmmhh 70. What about others? Can we move forward. What do you think about long term things to prevent HIV?

73: Ahh because you would have protected yourself for a long time so that you do not get HIV

LM: Okay, 73. What about others? 75.

75: It is alright because it is a daily thing, and you can forget.

LM: Mmmhh she is saying she can get tested. Alright ehh do you have questions or anything relating to this discussion that you would like to talk about. Questions? (silence) ahh if there is nothing we have come to the end of our discussion. Thank you for your time and for your views. Thank you very much we are done.

All: Thank you.

The End
