# Supplementary material for: Perceptions of pre-exposure prophylaxis among sexually active adolescent girls and young women in Zimbabwe–A qualitative study
Source: PLOS Glob Public Health. 2025 Dec 2;5(12):e0005396. doi: 10.1371/journal.pgph.0005396 (PMC12671731; doi:10.1371/journal.pgph.0005396)
Supplement: S1 File — (ZIP) [file pgph.0005396.s003.zip › S1_File/AGYW-FGD 09-Translation.pdf]

AGYW-FGD 09 -Translation
Facilitator: LM
Note Taker: KC
Date of FGD: 23/03/2023
Age group: 20-24 years
Translator: SM

LM: We would like to thank you, the time you took to have this discussion with us. Ehh if you still remember my name is Lindiwe and the one l'm with is Kudzai. Today we will have a discussion on your views about PrEP which is Pre-Exposure Prophylaxis, to understand its reception, reasons why they are few people using PrEP and also the issue of ehh how well is it being taken by adolescent girls and young women. This will give us important information on how best can we come up with new PrEP programs so that we increase the number of adolescent girls and young women in Zimbabwe who are using it and continue using it looking at those who need it and are willing to use it. Our discussion will take about 1 hour 30 minutes to 2 hours to finish. So in this session we will do a discussion and role plays. When we are about to do the role plays, we will ask that you get into 3 groups. Each group will do a play on how adolescent girls and young women will respond to different situations that are about PrEP right and their views on risky behaviors that put people in danger of getting HIV. So each group will be given a scenario that they are going to play and it will be explained to them and they will have three minutes to agree on what they are going to talk about on their role play. So just three minutes to practice then you will come and act for the whole group right. Yes, so we will ask all the groups to give each other time to act, and this will be followed by a discussion to analyze the role plays that were done. Ehh l hope we have spoke about our rules right. Our ground rules especially the one about our numbers (chuckles) the ones that we have, our names that we have right.

ALL: Yes.

LM: Mmmhh alright. Ahh we will not call each other using our names. I know we all know each other but l will ask that we call each other using our numbers right. Don't forget and end up saying ahh Lindiwe said, that will mean you would have used her name. The words that are going to be recorded will not have your names but will have these numbers right. So, do you have any questions before we start? About this discussion? (silence) Can we move forward. So, as a way to start, have you heard about PrEP? 89.

89: Yes l have heard about it.

LM: Ehh can you tell me where you heard it from, what did you hear?

89: Okay the time when l heard about PrEP, l had seen a car that was written DREAMS...

LM: Mmmhh.

89: I was at the shops at XXX just visiting and l saw that car with some ladies that l didn't know and they told me their names. They told me about PrEP, that what is PrEP. They told me that PrEP is a pill that you are given after you have tested your blood, if you find your blood HIV negative you will be given that pill called PrEP that will protect you even if you were to have sex with someone who is HIV positive you will not get the HIV virus. I saw that this thing will help me because l am also involved in risky behaviors. I then took that pill, l then tested my blood, after testing l then took that pill called PrEP. They told me that this pill if ever you don't trust your partner you just take the pill everyday at the same time but if it is, since we are young people you can have your partner and they say, then he goes to South Africa or he goes to look for work

somewhere, if you know that he is going to come, you drink the pill 7 days before he comes.

LM: Mmmhh.

89: Then when he comes, you can have sex with him even if you do not condomize, you won't be at risk of getting the HIV virus. Then when he has gone back where he looks for money...

LM: Mmmhh.

89: You will take for 24 days after he has gone. Then you can stop it. I also took that pill and (child speaking) drank it. After my partner had gone l saw that this thing really works, and l went back and tested my blood and l found out that my status is still the same. So l saw that this thing is right it won't put me in risk that l will end up not trusting myself thinking l have the disease.

LM: Mmmhh.

89: Even now l am still taking my pills, if they get finished l go and collect more.

LM: Alright.

89: That is where l heard about PrEP.

LM: Ahh thank you. Ehh can we have others, have you heard about PrEP? Tell us where you heard it from, what was being said? (silence) it is our secret. Yes 83.

83: As 83 l heard about PrEP, the day l heard about it l was just walking like 89, l didn't know anything about DREAMS, l always heard there is DREAMS there is DREAMS but l did not care about it as a person who liked being at home. So when l was just walking in the shops l heard there were DREAMS people and they came with a pill called PrEP. I asked myself what kind of pill is that and thought maybe it's for family planning or something else. I went there to understand more, l got there and told them that l heard there is something called PrEP but l don't know anything that is why l came like others. They then explained to me how PrEP works just like what was said by 89 and l also saw that it is best to try it. I then took that pill and used it for the first month. I went back to test my blood and everything was still alright. Then l saw that the pill works then l also started telling others but l had not understood that the pill protects from the HIV virus not pregnancy. So people ended up asking me why l am always going to take it since personal only protects from HIV not pregnancy and l said no they said it protects everything because l had not understood...

LM: Mmmhh.

83: And people said no let us go back and ask. We got there and asked them what this pill protects from. They then explained which made us to continue and the number of people taking PrEP to increase.

LM: Alright thank you. What about others? Where did we hear about PrEP? Are there any who have never heard of it? Who started from here? We all know it?

ALL: Mmmhh.

LM: Is there anyone who doesn't know it? Alright can you tell me that you have never heard about it a bit. (someone coughing) 85 said she has never heard about PrEP. Is there anyone else who has never heard of PrEP? (silence) okay. Are there others who want to explain where they heard about PrEP? Can we get 2 more. Ehh 90.

90: About PrEP, l was going to the clinic and l found DREAMS at the clinic, they asked if we had joined DREAMS and we said no. There was a meeting and they said we have come to tell people about a pill that we have that protects from HIV/Aids that is called PrEP. So this PrEP is taken by someone who have tested and saw that they are negative...

LM: Mmmhh

90: They gave us and we drank it and the first month ended. They came back again at the shops at another place, l heard there was DREAMS and a little gathering and l went there. They asked me if l had used PrEP before and l said yes, they asked if l wanted them and l said yes and l took them and drank for 3 months.

LM: Alright. Thank you. Are there others who have never taken PrEP? Those who want to tell us, maybe that they heard but they did not like it? (silence) Okay we move forward. Ahh now l want us to do our plays that we spoke about. So l would like to ask that we get into 3 groups right.

ALL: Mmmhh

LM: Can you choose each other that we want... Alright before we do the plays, there is someone else who said she doesn't know PrEP right. Even those who know it we will show each other ehh l see this, is this the PrEP you have?

SOME: Mmmhh

LM: Alright so those who don't know PrEP this is the PrEP that is drank, those who take PrEP are using this one. Inside there is a pill (sound of pills in a bottle) that is like this. Just pass it around there are some who don't know it so that they see how it is. Then there is ehh it is called vaginal ring, it is like this...

XXX: I did not hear what you said.

LM: What?

XXX: What did you say.

LM: It's called a vaginal ring

XXX: Okay.

LM: That is put in a woman's private parts. So it is inserted, this one is done by someone who is qualified for it right. It can be a nurse or a health worker who is qualified to insert it. It works for a month. It will be inserted then stay there, at the cervix, right. There is medicine that will be released bit by bit that last for the whole month. So that is the other type of PrEP that is available right.

ALL: Mmmhh.

LM: Yes. S, what do you think about these pills and the ring? (someone clears throat) Yes 87.

87: I was thinking the ring is the answer. (everyone laughs)

LM: Mmmhh 86. Alright l will come back to you why you are saying it's the answer.

86: 86. I agree that the ring is right, but l have a question, what if you are on your periods? Does it not affect you? Does it not get affected?

LM: Mmmhh. Okay. Is there anyone who can think, who wants to respond to that and let's see what people are thinking? (silence) Do you want to respond 89?

89: I had an idea as 89. I had heard that ring will stay at the cervix so meaning that when it is there, when you are bleeding that blood that will be coming out will be flowing and not disturbing, the ring will not be disturbed because that blood that will be coming out will not touch it. It will not drag it, the blood will be flowing and the ring will still be in place. That's my opinion l don't know.

LM: Thank you. 87 you said it's the answer. (everyone laughs) (someone coughing).

87: PrEP has pills and some of us we tend to forget. You find that l have started drinking them, today l skip, tomorrow and the day after l skip...

LM: Mmmhh.

87: The day after tomorrow l skip. The other thing is l discovered that these pills are 30 inside and the thing of drinking 5 days before, 27 days after your partner is gone so it won't be enough for those other days.

LM: Mmmhh okay. Is there anyone else? (someone clears throat) alright 86.

86. The disadvantage of pills is that maybe sometimes the boyfriend won't trust me that these pills are for prevention (baby coughing) not HIV, we will end up fighting him saying LM taking HIV pills while l'm not.

LM: Mmmhh.

86: So with the ring he won't see what l am ppreventing. It won't be noticeable but pills everyday taking and taking them it will end up looking like LM taking HIV pills. People cannot differentiate between PrEP and ARVs. They think we are taking pills for, so l think the ring helps.

LM: Mmmhh. Then 82.

82: As 82 it is my first time to hear about the vaginal ring. I wanted to ask how long do you stay with it?

LM: So, you put it, it is inserted for a month, after a month it then gets removed then insert a new one right. The medicine inside will have been finished or expired and no longer working. Yes 89.

89: As 89 l will speak on both things, PrEP, and the ring. Personally, l think PrEP is better because if you teach yourself something you will know that by this time l am supposed to do this...

LM: Mmmhh.

89: (clears throat) you will be knowing that by this time l should remind myself because you will not be dealing with someone else's life, you will be reminding yourself about your life so that you will not be at risk of living with the virus. Then can l ask about something from back, sometimes you find that (someone chuckles) maybe l have inserted the ring, l don't know that the ring (someone coughs) when it's inserted, how long should l stay without having sex with my partner? So if l am to have sex with my partner maybe... (inaudible)

LM: Mmmhh

89: Does it mean he wont... (everyone laughs)

LM: He won't what?

XXX: He won't feel it? (chuckles)

89: Does it not shake (everyone laughs) to the point of him feeling it? (still laughing) you will end up being asked that what is going on.

LM: What is going on. Mmmhh.

89: Then with PrEP, if you realize to us who are using PrEP we were given small containers that look like lip balm that you will put your pills inside. Then when your time is up maybe your partner is there you just take your lipbalm container then you put that thing inside and close your container and put it safe.

LM: Okay.

89: Yes.

LM: Alright. So what is the reason for you to be given these containers of lipbalm?

XXX: We will be doing it for, l think they will be doing it for, you see this container, you also see that, can you please give it to me (bottle of pills making sound) (people laughing) l am now at my partner's place and time is up in my handbag, there is nowhere l can (shaking the bottle of pills) you hear that (everyone laughs) it's making noise (everyone laughs again) the container makes noise so the lip balm container when you open it there is no sound totally even if you open it with your partner there won't be any sound...

LM: Mmmhh.

XXX: And he will think you are carrying your lip balm.

LM: Mmmhh.

XXX: You moisturize your lips, some lips crack. So here there are some dangers, other marriages can be destroyed...

LM: Mmmhh.

XXX: Due to the pills making noise.

LM: Mmmhh.

XXX: Yes.

LM: Thank you. Ehh is there anyone else who want to talk about the pill and the ring? (door making noise) Alright so (door making noise) there is another type of PrEP called injectable or injection, where one will be getting injected right. It is either she will be injected once a month or once in three months right. Yes, so what do you think about this type of PrEP? (someone pulling a chair) How is this type of PrEP? Yes 86 then we go to 83.

86: I think it is alright.

LM: Mmmhh.

86: Especially to those who say they will forget pills or if it is the ring and it has shifted or what what. I think the injection will not disturb you and it will take time. You just have to master the expiry date and go get injected again.

LM: Alright okay. 81. (sound of a chair being pulled).

81: I also wanted to agree with 86. I think it is right because like vagina, the vaginal ring, we do not know if it has side effects or not. Some of us would be having allergies...

LM: Mmmhh.

81: And when you have inserted it then you hear that your womb has rotten because of that medicine (participants laughing) and that is painful. So l think the injection is better. (baby sounds)

LM: Okay alright. So with the injection, you spoke about forgetting pills right but if you get the injection, when you get the injection it will already be in your system then you also spoke about, you said the ring can have side effects then you end up having some problems right. So, those are the fears that you have (baby crying) ehh the advantages of the injection. What about others, what do you say? Looking at the injection, the vaginal ring, and also looking at the pill. (baby crying) Yes 87.

87: I had a question (baby crying) that lets say we have taken the virginal ring and the injection will we find it when we go back to the clinic, or we will be told that something like that is no longer available?

LM: How would that make you feel? You had gone and you were given the injection, or you were also given the vaginal ring and when you go back, they tell you it's no longer available?

XXX: Or not finding a doctor who will remove the ring (participants laughing) when it has expired.

LM: How would you feel? What, which feelings will be evoked? 82 then 89.

82: It's painful when you get told that the doctor is not available. You will be thinking that since this thing is still inside me what is it doing since it has expired (someone chuckles) because they also tell us that expired food is not good for people.

LM: Mmmhh.

82: So you will be asking yourself that since it's still inside what is happening because you can stay up to a year being told that the doctor is not available.

LM: Okay. Alright. Yes 89.

89: Okay as 89 l think (baby crying) as 89 l think that if l have been inserted that ring (someone coughing) and l agreed that l will have it inserted, now the time for it to be removed is up then l don't know who will remove it. I think that will be pain that will make me to lose weight (someone chuckles) for a long time. (everyone laughs)

LM: Alright.

89: Because l will be thinking of emotional and physical stresses.

LM: Mmmhh. Alright we will end with these two then move forward.

86: Let's say l had the injection or the ring then they say the doctor is not available what what and they start saying stories, this will be like abuse. What if me and my partner want to have a good time and now they say they are no injections. How will l have sex when the prevention that we were using is no longer working?

LM: Mmmhh.

86: Mayne l had not told my partner that l have something like this...

LM: Mmmhh.

86: So he will be wanting us to continue with what we were doing, what will l do for us to have sex whilst that person... It will be hard for me to have sex with my partner maybe end up fighting.

LM: Thank you.

XXX: The other thing that l wanted to suggest to 83, how would the health people see... (door making noise) because you will cry saying these people came to play with our minds knowing that they will go with their thing, so us we no longer go to the clinic, better we have the virus and this will make the disease to be everywhere.

LM: Mmmhh.

XXX: Because people will now tell themselves that going to the clinic is worst of time.

LM: Okay. Alright so pills are always available? Because l am seeing that we have pills here right?

SOME: Mmmhh.

LM: Mmmhh those are the ones you know. Have they ever been not available that you have time without finding them? 90.

90: Yes we were given sometimes if we hear that DREAMS will be having soccer matches where there will be games, then they will give is 1 or 2, then you take them if they get finished you won't know where to get them.

LM: Mmmhh. So, they would have come if there is an activity?

90: Yes, sometimes there will be ball games or tournaments.

LM: Okay. Alright. Will you be tested there?

90: Yes.

LM: Alright. 89.

89: I am 89 and l have never not found the PrEP pill.

LM: Mmmhh

89: Because Mac comes. Maybe every after 2 months or 3 months. Maybe they come and ask...

LM: Who?

89: From Mac.

LM: Ohh. What is that?

XXX: Matabeleland AIDS Council.

LM: Alright.

89: Yes they come and ask us, they give us family planning pills. They have Depo that they will giving to us who do not want to be mothers yet (someone pulling a chair) they give us and those pills they also give us. They ask if we still have them and we tell them yes. They give us their phone numbers…

LM: Alright.

89: Like right now, as we are a group they will give us their phone number and tell us that if the PrEP pills are finished we should call then so that they come and give us more because we should not stay without them.

LM: Alright.

89: So if they would have come like today the date is 23, all of us will be knowing our review date. Then we tell them or we ask that may you please come on certain date. Those who need services they get them, everything that we will be needing like pills, Depo, PrEP. We get it because they will be helping.

LM: Alright it's okay. So, l want us to get into our plays, right?

ALL: Mmmhh.

LM: So l would like you to get into groups. Shuffle each other and mix, right?

SOME: Yes

LM: Ehh so that… so l would like to have one group having 4 people and others will be 3 (chairs making noise) that's it right?

ALL: Mmmhh.

LM: Yes. So if it's like that (chairs making noise) put each other in groups before l read for you. (chairs making noise) (participants talking) Alright so l would like to mix you here. You, go and sit there. 84 come this side, then 85 come here, 82 go that side. (chairs making noise) you, l will put you here. Alright so our first play (chairs making noise) is about Chido and Koko, you are the ones who are going to do it right? So l will read for you: Chido is, so you will, l want you to act a dram, a small drama for about 3 minutes. Yes so you should see how you reach each other, what are you saying? It should be interesting, right. So your play will be looking at Chido who is 16 years old. She is dating a 50-year-old man. Those are the blessers right?

All: Mmmhh.

LM: Do you still call them sugar daddy?

ALL: No.

LM: They are now blessers right? Yes. What do you call them here?

XXX: Blessers.

LM: Blessers. Mmmhh so she has her blesser. She recently started taking PrEP since she is worried that (child shouting) she will get HIV. She thinks that her friend Koko who is 19 years old is also at risk (child shouting) of getting HIV by having sexual relationships. (child shouting) She is encouraging Koko to also take PrEP. So here Chido has already started taking PrEP right?

ALL: Mmmhh.

LM: So she is also encouraging her friend Koko who is 19 years old to ahh since she is dating people and having sexual intercourse with them she is at risk so she should also take PrEP. (participants chatting) So do a play between Chido and Koko, putting together what you think would happen in life if Chido comes with this plan. So the second play: NaBhobhi and NaJuru. NaBhobhi is you here, so you will look for two people who will act right?

Group 2: Mmmhh.

LM: Yes, come up with an interesting play mmmhh. So NaBhobhi is 23 years old. This is your age mate, she is also married. NaJuru is 21 years old and also married. So NaBhobhi's husband has a tendency of dating other girls (child shouting) so she is worried (chuckling) that she will get HIV. She had about PrEP on radio and she went and started taking PrEP and its been six months since she started using PrEP. What does she have, 6 months since she started using PrEP, NaBhobhi because her husband is dating other people, right. However she is thinking of stopping using PrEP. Do a play on the conversation between NaBhobhi and NaJuru putting together reasons that makes NaBhobhi to want to stop using PrEP, telling us why she wants to use, why she does not want to use PrEP.

Then we go to our last play, the third one: Three friends, Peppa, Sky and Princess. They are all over 20 years. They are over 20 years right. And they are having sexual relationships with their partners, and they go to the same school. They were chosen to come up with a PrEP program for adolescent girls and young women for an orgainastion called CeSHHAR that looks at sexual health and population. (participants talking on the background) So can you stop talking too much l am still reading for others. They planned, they are planning on presenting their ideas for this program that they planned. Do a play about their conversation looking at the things that they think should be there in this program. So they a planning a PrEP program that the program of PrEP should be like this, be offered where, be offered by who and what is going on right. So, here you should act showing us that this program that you prepared for us is about what and how is it going. So you will be three people those friends.

XXX: Okay so since we are 4… (someone laughing on the background)

LM: You would be three those who are going to act. Mmmhh l will give you 3 minutes.

(recording paused)

LM: Ahh alright. So we can move forward. Can we please have Chido and Koko's play.

Role Play: Chido and Koko

Narrator: *Hello. We will give you a play. Our characters are Chido, Koko, Narrator and the Nurse. Thank you.*

Chido: *Koko my friend did you here that there is a car that was here from DREAMS that was giving people PrEP pills because we have sugar daddies maybe they*

can help us and give us the pills to protect against HIV because l do not trust that old man.

Koko: *I will see about that.*

Chido: *Friend you will say l told you so when you are lying down sick.*

Koko: *We will see about that.*

Narrator: *After three months we meet Chido and Koko.*

Koko: *Hello my friend.*

Chido: *Yes friend, what do you want?*

Koko: *Yes. Did you hear that Thabo is sick?*

Chido: *I told you about your Thabo that he is sick. What is wrong with Thabo?*

Koko: *He now has the HIV virus.*

Chido: *Then what are you thinking of?*

Koko: *I am thinking of going to take PrEP pills also.*

Chido: *Lets go and see a doctor about this Thabo of yours. But l told you. (sound of a recorder)*

Dokotela: *Hello ladies. How can we help you?*

BOTH: *Hello doctor.*

Dokotela: *Hello.*

Koko: *Ehh Chido once warned me about the PrEP pill so l just said l will see because l did not have enough information and l also wanted to enjoy the world. So now l am sick may l please have help with PrEP.*

Dokotela: *This pill is taken after we have tested your blood so l will first test your blood.*

Koko: *Okay it is fine.*

Dokotela: *Come this side. Koko your blood looks clean so l will give you this PrEP pill. PrEP protects from the HIV virus. You drink it 7 days before you have sex, then you will drink it 24 days. Do not forget Koko.*

Koko: *Ehh it is okay.*

Narrator: *Thank you, our play ends here. (everyone clapping hands) (participants laughing)*

LM: Thank you to Chido, Koko and their doctor right. (chuckles) Ehh so let us look at the play between Chido and Koko. Are there people like Chido who have sexual

relationships with old man? Blessers? (participants murmuring) Mmmhh can you explain it for me. 86.

86: Yes they are there but a lot of times it will not be according to our will. Let's say me as an example I am young with age but l can find myself having sex with someone who is 50 something years old. It would be because at first l dated him because l wanted his money but him at the end, he also wants payment. He will say because maybe l have given you money for clothes, he clothes me. I am at home maybe he is now the one helping us, so he will say since l have given you all these things how are you going to pay me? There is nothing that l can pay him with but to sleep with him.

LM: Mmmhh. So payment is for doing things for you right. 89

89: Okay as 89 l think this is common a lot. You will find out that at home l am the one, l am a child but l am now the parent to other children. When l look at the home situation there is no food, l have my little siblings and there is no food, there is no body lotion at home, there is no sugar. Maybe when l am just walking l find a man driving a BM, this person and money, he does not show the money because l have asked for it or l have told him about my reasons at home, but it would be here and when l also see it l would tell myself that this person has what, money. I date that person knowing that if l date him and he gives me money l would be able to buy my siblings food at home, be able to have a good life at home not poverty.

LM: So you will be dating him for money right.

ALL: Mmmhh.

LM: Because the situation at home will be bad. What about others? Do you think there are people like Chido? That she would date people who are 50 years old, old people? Alright 91 then we go to 84.

91: 91. I agree with 89. Ehh that things like that ahh, young people at most, young people now that is what we are coming across and what we are warning that maybe if you are there, l am at the programme, l will jus say this, if there are there who see maybe their friend or their neighbor ehh in a situation like this you can get in touch with me. I can, l am at a program where l can counsel them and discuss about life, and show her how best she can live.

84: Ehh as 84 l think this is common. I even look at school, some children get tempted when they see other children eating nice things because of her boyfriend and she will also see best (someone coughing) that an older person will have money

LM: Mmmhh.

84: Yes.

LM: So we are saying this dating, this dating they are doing it for money?

XXX: Yes money so that you will also look better.

LM: Alright (someone clears throat) lets hear 88. Your voice.

88: As 88 l agree with this, l do not dispute that they are dating for money. But there are there those who tell themselves that they can not stay without having sex with a man, that is why they date a lot of different people and end up dating old man.

LM: Mmmhh. Alright ehh what about others? Mmmhh let us see 83.

83: As 83, some of us date so that when they see me coming out of a GD6 my friends will be shocked, as 83 they do not have a say anymore and also l will be giving my friends orders, that you saw that car, it is mine.So, right now l want you to go to the shops and buy me a drink and you will run because you know that l have got everything.

LM: Mmmhh. Alright coming to 89.

89: Okay me as 89 the other thing that l think us as children, that l think we, we, that makes us end up dating man older than us is fashion.

LM: What?

89: Fashion.

LM: Mmmhh.

89: Right now when someone sees me wearing maybe what do they call it (chuckles)

XXX: Crop top.

89: These clothes of these days that are available…

LM: Crop top.

89: Crop top and what what, she will not know what made me buy them. Maybe l do my piece jobs going to fish fish and selling it and get money right, and she also wants to look like me but she does know that how l got that money. When she sees me drinking a drink, maybe it is a drink that l got here today at CeSHHAR, she does not know how l got it and she is also hoping to be seen carrying what, that drink because she saw me carrying it.

LM: Mmmm…

89: Just competition.

LM: So now it is a competion?

89: Yes.

LM: Alright so do you think Chido is at risk of getting HIV? Chido is the one dating an old man. Do you think she is at risk of getting HIV? (someone clears throat) Yes 87.

87: Ehh as 87 l think Chido is at risk of getting HIV because this man is old and she doesn't know that he, he has slept (participants laughing) he has slept with how many girls before coming to her and that if these girls were positive or negative she does not know.

LM: Mmmhh coming to 90 your hand was up.

90: And that this person she will be dating is older than her and even with the mind he will have more power over her. You can tell her that let us protect using a CD or something else but he will refuse wanting you to do what he wants. Then you will find out that the person has infected you with the virus because you will not be knowing his status.

LM: Mmmhh alright. (door making sounds) ohh so he can refuse to use protection because he is older than her? Alright ahh is it common for young women ahh, those who share health decisions, to tell each other about health issues, we saw Chido and Koko discussing that you should go and look for PrEP my friend. Do you think that happens in life? That these age mates advices each other about health issues? 83 then 89.

83: As 83 l say l think it happens but it comes down to who is advising you on how they take it. Some are those that do not want to be told, they believe about seeing so it is up to her on how she takes when you have told her.

LM: Mmmhh.

89: Okay as 89 l think it is common especially us as young women in these days we do not hide secrets like right now talking about PrEP, some of us here we know PrEP and we even called it that 'we do not want to have tetanus', if it is the day to collect PrEP we come in our numbers…

LM: What do you say?

89: We say we do not want to have tetanus. (participants chuckling)

LM: Why does it have that name?

89: We will be advising each other that friend if you take that pill it helps that you do not get the virus. Yes and she will say ahh friend. Each person will know her sides where she goes. So, the other one will say ahh friend l wish to get my blood tested so that l see that the time l went l did not get that thing right (baby crying) (inaudible chatting) and the other even my friend when she sees me taking PrEP you will find out that embarrasses her that she did not what, she did not take that PrEP, then she goes to test so that she also sees her status then she start taking PrEP because as friends we have that thing of telling each other secrets and other things that are natural that comes. And the other person will also say my friend let me go and test. And then you find out that they tested and when the results are good, she will tell you that ahh my friend l did not get tetanus l thought l will get tetanus (laughing)

LM: So people ask each other only as close friends? Or she will ask other people? As young as you are, have you asked each other, ask or (clears throat) or advise each other who are not your friends? (clears throat) 86.

86: I 86, yes we talk anywhere, especially to us who play ball now some girls play ball. In sports programs like these are always spoken about we discuss about such. Even if DREAMS come and find us at the ground, they can come and ask for that time to discuss.

LM: Ahh alright. Ahh so where do you get this PrEP information? PrEP information in your community, where can a person get PrEP information? Mmmhh 83.

83: In this community we have DREAMS facilitators so every time when they have their sessions, they talk about what, PrEP. And also at the clinic we have OPHID nurse, she also advertise, she advises sex workers when they come that there is a pill called PrEP that is there, or there is a change that is there where at the clinic. So every time we get this knowledge.

LM: Mmmhh. Alright. So DREAMS facilitator then nurses, there is an OPHID nurse here at the clinic right who tells people about PrEP. Are there any other places where people get knowledge, information about PrEP? Mmmhh 89

89: Okay l as 89 there is, there are other from MAC…

LM: Mmmhh

89: I once spoke about MAC…

LM: Yes.

89: Yes. They also explain to us about PrEP all the time. It's either or if they find you walking in the road they stop their car and tell you about PrEP. If you do not know it they ask you if you are comfortable that they test your blood and then you start using PrEP. It is now your opinion in your heart that you will feel comfortable or not. Then we have, others who sometimes tell us a bit even if they are not 100% yet National Aids Council…

LM: Mmmhh.

89: Yes. They tell us a bit about PrEP about (someone coughing) what they have.

LM: Okay. Alright alright. So you are saying adolescent girls, young women that is where they get information about PrEP? Is there anything else, other places where they can get information? Mmmhh.

XXX: So far now we are getting it from where we mentioned…

LM: Alright.

XXX: If there is anywhere else, you can tell us so that when we do not shy away when we see the responsible people.

LM: Mmmhh. So what do you think about Koko's response when Chido told her that she should use PrEP? So we saw that the first she was told she did not like it, right? Yes then we see her coming back and saying she now likes it. What did you think about Koko's response? Mmmh 87.

87: It is because she had that her partner now has the virus so that is why she rushed to Call her friend and tell her that they should run to the hospital.

LM: Mmmhh. So her partner's sickness is what pushed her to go and look for PrEP? What about others? Let us hear 92.

92: Ahh my opinion is that l think from the beginning she heard that there is a pill called PrEP but she did not understand and then it happens that there was this problem that she saw that if you do not protect yourself, you can get affected. Then she now starts to think that no my friend was telling the truth then she starts to call her.

LM: Mmmhh. 85 what do you think of Koko's response? She first refused, the second time she is the one who ran to speak to Chido. (children making noise outside) Do you think this happens in life?

85: It does happen that if you trust your person (door making sound) you regret. So even if you are being told by your friend to go and test so that you know your status because sometimes some men do not agree

LM: Alright so she will be testing, trusting your advice…

85: He will be knowing his status so your friend maybe tells you that my friend go and test so you know how clean you are…

LM: Mmmhh.

85: Ehh so that maybe you find out that you now have the virus so you have to treat yourself because your man that gave you the disease (noise from chairs being pulled) will have disappeared, you alone have to treat yourself.

LM: So is Koko's response common, does it normally happen? (noise from chairs being pulled) (someone coughing) Would you say it is a common response that was made by Koko? (door making noise). 86.

86: Yes 86 it is common. A lot of people do not agree when we tell them about PrEP they say it is an HIV pill. So they are few people that when you tell about PrEP they understand that it is not an HIV pill but a pill to protect against HIV. So when you tell them about PrEP they tell you that we do not want people with AIDS. (baby sounds)

LM: They say we do not want people with AIDS. (chuckles)

86: They really refuse and say we do not want people with AIDS, where have you seen something that is drank daily that is just like ARV it is the same as that you are drinking it, they have containers these days they are rigging (someone coughing) they are exchanging containers (child talking) but what is inside is the same (child talking)

LM: Okay. (laughing) Alright so are there any other answers that are, that you expect to be made by someone like Koko? That when Chido approached her are there any other answers that you think can be made by someone like Koko when being told about PrEP? We are told, we have been told the other one. 82.

82: As 82, Koko, 86 can advise 84 that she can take PrEP pills then she will agree and take them and just keep them at home without drinking them. Then it will happen that she get the disease, she will not be protected from AIDS.

LM: Alright what about others? Are there any other responses that you expect to be made? 88. (someone coughing)

88: I as 82, there are some who do that. Those who refuse even those who are already drinking ARVS, they are there who do that, that at the end when someone is seriously ill they will start to think if only they took the pills (child talking).

LM: Alright. (child talking).

XXX: Some when you try to advise them about that if they want to protect themselves there is a pill called PrEP to protect from HIV. Some will respond that it's PrEP for what, how does it help? Its now like you drinking these pills for everyone.

LM: Mmmhh alright. So yes 89.

89: Okay. The other point that l had about the drama that l saw (someone coughing) 84 and 86 giving each other advises, sometimes even if 84 called 86 and told her that she heard that her partner was diagnosed with the HIV virus, he is positive, some would tell themselves that they are also what, HIV positive and then not go to take PrEP, to go and get tested because you can find out that her immune system was high for her not to get the HIV virus the time, she was dating her sugar daddy. She would tell herself that since she is still fresh, she would see that PrEP does not work because she will be seeing herself fresh to the point that she actual gets the HIV virus…

LM: The she gets the virus.

89: And other people cannot share their emotions with others. She would have heard that her boyfriend is sick then she would tell herself that it is the same she is still fresh she will not get sick. She will stay scared of going to the clinic to test, afraid of telling her friends to the point that she will be at risk of getting the virus because she will be afraid that if she tells her friend she will spread the information.

LM: Information alright. What is good, what do you think is good about using PrEP? (silence) 86.

86: The good thing about using PrEP is that you can date someone who is positive, HIV while you are not (clears throat) there is no problem. Let us say l love my boyfriend with all my heart but he is HIV positive or maybe it is someone who is courting me and l also want him, and he tells me his story that he is on ART while l am not but seeing

that me and this person we have a connection so it will be easy if l am using PrEP to live with that person and do what happens while in love.

LM: Alright. Let us hear 91. What is good about using PrEP to you young people?

91: I think using PrEP is good because there are other people men who are born positive so l think it makes, it is good by that when you have sex with him you will be knowing what type of person he is, his statues and he will also feel that he is not being discriminated. And you even if you know you will accept it because you would have protected yourself with PrEP.

LM: Alright. Ahh yes 87.

87: I think it is good because men do not know how to tell the truth. A person will come to you and tell you that he is okay, and then you say let us go and test then you hear him telling you, he will refuse. Sometimes if you are dating sugar daddies that thing that he is older than you he will tell you what to do because he is just like your father (someone coughs)

LM: Alright.

87: So if you take PrEP and drink it even if he refuses you will know that on your side you are safe.

LM: Alright so is there anything else that is stopping young women from using PrEP? What are the reasons that are making others to not use it? Let us hear from 89 then we go to…

89: Okay as 89 l think the reasons at most that make us to be afraid to use PrEP is that thing that you have to test your blood first.

LM: Mmmhh.

89: Because you will be knowing yourself that maybe from the past you did somethings wrong but you do not trust yourself because you are still seeing yourself as what, fresh. Then you think that when you test your blood obvious l will be what, I will be tested HIV positive. So the PrEP that l wanted l do not want it anymore l will go to the other group that is not…

LM: On the other group. So someone will be afraid that they will be found with a positive result.

89: Yes.

LM: Alright what about others? What is stopping or l can say that is disturbing the use of PrEP? 85 you want to say something?

85: The thing that makes us not to take it l will say it starts from men like the men that you stay with that he will see me maybe drinking those pills then he will ask me like why am l doing that.

LM: Alright, I see you 86. (someone coughs)

86: The other thing that makes us afraid to take PrEP, l will be seeing that maybe let us say that you would have came like this to give us PrEP pills, I will be coming from taking them l will think maybe the people from my same group will go around seeing that XXX partner has this status. So l will be scared of taking PrEP telling myself that maybe (chairs being pulled) so who and who will go around saying so XXX's partner is on ART. So people end up being scared of taking PrEP scared of the fact that if you take it maybe l do not have 1 boyfriend but l have 2 3…

LM: Mmmhh.

86: Or in those boyfriends that l have there is one of my friends who is friends with that boyfriend so when she gets there she will tell him that l saw so and so taking pills, so are you like this or she is the one who is like this? Then the boyfriend will say he doesn't know any of that…

LM: Alright.

86: So when l get there we start fighting with that person you see? That is what making us afraid of taking PrEP.

LM: Alright. So… ehh 83 and then we will move forward.

83: I have met a situation where this other girl told me that her religion does not allow them to go to the hospital, they do not allow such things. Then l asked her what was the reason she said that God is there and God has power if you are sick you just pray

everything will be alright. So l asked her that even in HIV she said yes that is their belief and l should not argue with her because that is her belief. So l think the other thing is that we are supposed to go to churches, churches should get advise like these to allow people to go to hospitals. They should allow young people to what, to deal with their lives.

LM: So does she see her self at risk of getting HIV or she does not see herself at risk?

83: She said even if she is at risk she will not go against the will of their church because she is a believer of their church.

LM: Alright. She believes in a church like which ones? (Participants laughing)

83: 89 seems like she knows the other one.

LM: Which one 89.

89: It is the SDA.

LM: Alright she said it is the SDA.

83: Then those ones who wear white, those who do pots.

LM: Mmmhh alright. (clears throat) where can adolescent girls get PrEP? Where can they get these PrEP services? Looking at your community (chairs being pulled) where can they be found? Someone who has never taken PrEP where can she go and take it in these different places? There are other places that you mentioned but they were for information right. Is that where they can also get PrEP? Where would they be getting it from? For someone to go get PrEP services? Yes 89?

89: Okay. I as 89 some times it is being influenced just a bit that makes that maybe like now say we are being given PrEP by MAC maybe l see that it is finished so l have to go to the clinic, when l get there at the clinic to take my PrEP when l go out from the clinic l find that maybe there is my neighbour or my friends maybe from afar, in my bag the pills will be making sounds. They will not know that it is PrEP, I will hear it everywhere that XXX is on ART but l will be taking my PrEP. (laughing)

LM: Laughs) alright. So are you now saying that the clinic is not the right place? What are you saying about the clinic?

89: It should have PrEP so that we get it easily…

LM: But we would be hoping that maybe it should have like here that there is class1, there is class2, there is class 3, there is class 4. They should know that that is the room for what, for PrEP…

LM: Mmmhh.

89: Because everything is coming from 1 room…

LM: Alright.

89: So when you go around going to take your PrEP and putting it in your bag they will be saying XXX is now on ART but no it's just PrEP.

LM: Alright.

89: So when you leave about to exit the gate all eyes will be on you…

LM: Looking at you (everyone laughing) yes 86.

86: I agree with 89 she is telling the truth especially when you enter the clinic you will see even us when we come to the clinic, we look at people. If you see someone getting inside with a bag she/he is going to take that. So if you see someone getting inside with a bag even if going to take family planning (laughing) by the time you exit the other side we say they are ARV pills…

LM: Mmmhh. So which are other places where PrEP can be offered? (silence) Where is DREAMS giving you PrEP? Yes 83.

83: DREAMS used to give us here at the shops but l realized that every time when the car comes all eyes will be there at the car wanting to see that who entered, the time you leave they will be looking at you with another eye. It would be like they have a lot of questions whether you are taking ARVs or what is going on because some say that DREAMS car is the hospital car that will be carrying pills. So obvious the first pills that come to people's mind are ARVs.

LM: Yes…

XXX: And the other challenges that we have, it happens that let's say the DREAMS car came and gave us our pills and what not, each and every one will be getting in the car taking what they want but l am saying that us right now at the club we do not have secrets…

LM: Mmmhh,

XXX: So we will be wishing that if there is another plan or plan B that can come so that we take them from there and that there is a bit of a distance that for example let's say maybe the car will have each person with their own there because we have other adults, these adults when they see us testing blood and you come out carrying a box they spread the wrong information to our parents, when you get home you find your parents already not wanting to talk to you because of PrEP but you will be trying to protect what, your life.

LM: Mmmhh.

XXX: Yes so some it's because of the wrong information that reach our parents.

LM: Alright. Okay it is okay let us look at NaBhobhi and NaJuru's play. Can you please act your play. (background chattering)

Role Play 2

XXX: *We greet again this afternoon. We greet you.*

ALL: *Hello*

XXX: *Our play has NaBhobhi and NaJuru. Let us hear what they brought us. Then there is NaBhobhi's husband. (footsteps) (someone singing and sweeping)*

SaBhobhi: *Don't you see that someone has entered?*

NaBhobhi: *Good afternoon my husband.*

SaBhobhi: *Good afternoon good afternoon good afternoon for what, what is this?*

NaBhobhi: *What is wrong my husband?*

SaBhobhi: *What is this NaBhobhi? What is this?*

NaBhobhi: *Ahh my husband l don't, this (sound of pills in a bottle)*

SaBhobhi: *What is this man (NaBhobhi screams)*

NaBhobhi: *I am sorry l don't know how the children maybe... What is it called l don't know.*

SaBhobhi: *You always say l like women when you are drinking pills...*

NaBhobhi: *Ahh l'm really sorry...*

SaBhobhi: *You want to kill me...*

NaBhobhi: *Really l am sorry.*

SaBhobhi: *You want to kill me NaBhobhi?*

NaBhobhi: *is not me let us ask the children. Let's ask the children. I am really sorry my husband, l am sorry.*

SaBhobhi: *I will deal with you today. (NaBhobhi screaming and shouting)*

NaJuru: *May l come in friend.*

NaBhobhi: *(shaky voice) come in neighbor.*

NaJuru: *You are crying what is happening?*

NaBhobhi: *Neighbor it's hard on my marriage it's like it will end tomorrow. It will end today evening, it is ending.*

NaJuru: *What is happening?*

NaBhobhi: *I don't know where my husband found my box. I had hidden it, the one for PrEP. When l was like… l found him, l don't know where he took them from. The he goes to the bar to drink alcohol to come and beat me because he is saying l am drinking AIDS pills. I did not tell him that l am taking PrEP.*

NaJuru: *Ahh my friend but this box of yours, you are now supposed to look for something small that is closed like lipbalm container.*

NaBhobhi: *Lipbalm container?*

NaJuru: *Just like lipgloss, like lipbalm and just remove them from this box that makes noise (sound of pills shaking in a bottle) you hear that it happens.*

NaBhobhi: *Neighbor really, why didn't you come with it to give me?*

NaJuru: *Ahh l didn't know that your husband is angry.*

NaBhobhi: *Ahh it's just that, today evening l am throwing away this PrEP. All the bottles l am throwing them away today because l dont want him to find them and because l dont want my marriage to end.*

NaJuru: *No don't throw them away, just take this thing and find a smaller container and remove them from this bottle. This bottle throw it away, throw it in an open space so that he will think you threw it away while you did not. And put the pills in container like that of lipbalm that smells nice.*

NaBhobhi: *Ahh neighbor l no longer believe in PrEP. I don't believe in PrEP, take it alone.*

NaJuru: *It is the same even if you stop, you will get the virus because you also know that SaBhobhi is fast...*

NaBhobhi: *He is really fast.*

NaJuru: *A skirt that passes him by he is there.*

NaBhobhi: *What did l do really neighbor for him to beat me for PrEP. Right now he almostkilled me because of the pills better l just have the virus then.*

NaJuru: *No what did they say at the radio, mmmhh you did not hear?*

NaBhobhi: *I did not hear.*

NaJuru: *They said there are now injections. There are now injections and rings. Just go to the clinic and tell them that your husband now knows about the pill they should give you an injection.*

NaBhobhi: *An injection?*

NaJuru: *Yes exactly injection.*

NaBhobhi: *Where is it offered so that l go and get injected? We will wake early and you will accompany me.*

NaJuru: *Yes we will wake up early and go this container has problems just throw it in the toilet.*

NaBhobhi: *This is better neighbor because the pill almost ended my marriage.*

NaJuru: *Get the injection and hide the card, or you can leave the card with me you will take it when going to the review.*

NaBhobhi: *Thank you so much. (everyone clapping hands)*

LM: Thank you to NaBhobhi, NaJuru and SaBhobhi's play right. (laughing) Alright so let's look at their play. What do you think about this play? In life are there young women who are in the same situation like NaBhobhi? NaBhobhi is the one that we saw sweeping and her husband came and saw the box of PrEP and things went bad right?

ALL: Mmmhh.

LM: What do we think about this play? Are there people who have this same situation? Do we think there are people who are in this situation? Mmmhh 83.

83: I 83 think they are there even if it is not your husband but there are other parents who do not have knowledge about why we go to DREAMS. When you go home with the container you will hear them say you are now dating, you are a sex worker, you now have the virus that is why you are bringing that container. They will not be understanding that PrEP, even if you try to make them understand what PrEP is about they won't understand because our grandmothers do not know a lot of things about school, all these things were not there. So the only pill that they know is the HIV pill.

LM: Alright. What about others? Are there other people who are in the same situation as NaBhobhi? 94.

94: Yes these kind of situations a lot of people do meet them. Because even if it's not your husband just your boyfriend when he had come to see you then he discovers the container, it will be a problem you will fight a lot.

LM: Mmmhh.

94: Because he won't be having information (someone coughing) that there are things like this, there are pills like these.

LM: So do you think it is easy for a woman like NaBhobhi to decide that they want to start using PrEP? This is a married woman. Do you think it is easy? To say that she wants to start using PrEP? Yes 91.

91: I don't think it is easy because l have been in that situation before.

LM: Mmmhh.

91: I had my PrEP and when l had that PrEP l got a visitor. When l had that visitor, l just took my PrEP container and my mind was far away. I just took out the container while the visitor sleeping on the side, it was not a secret and l did not know it was supposed to be a secret. I took out my container when l was about to open it he then asked me what the container was for. I said it is PrEP and he went out saying he is going to look for protection. He went out as someone who was going to look for protection and he never came back until today (everyone laughing) we never met after that.

LM: You never met…

91: Yes, and it was painful because l loved that person…

LM: Yes.

91: PrEP got between us.

LM: It is because of PrEP (laughing). What about others? (chuckling) What do you say? Do you think it is easy for women like NaBhobhi who are in this situation? Yes 88.

88: I don’t think it is easy because your husband will not trust you that why are you using it and he will tell you that since he is not taking HIV pills why do you need it, do you want to be unfaithful and bring me HIV.

LM: So do you think in life it happens that there is someone who would want to stop using PrEP while they had started it like NaBhobhi who is saying she is stopping PrEP? Do you think this happens? That someone will say l am stopping it? Yes 91.

91: The person who will have that mind will not be having enough information. She would not have learnt and got knowledge and accepted it because these ARV pills, some of them, you will see some people leaving the hospital carrying them in their hand without any care because they know that thing helps their life. So, if a person is committed to PrEP knowing that it will protect them and it is something that they should there is no need to say you do not want it anymore.

LM: Yes 89.

89: As 89 l have came across the situation like NaNana's situation, l think it is best for me to continue using PrEP because a man is not my relative. If l say l am stopping taking this PrEP, he is well known that he is a man who likes woman, tomorrow l will be the

one sick. When l am sick, he will be knowing that he is the one who looked for the virus and brought it home. So, l think for me to continue using PrEP is better because l will be taking care of my life, l will not be looking at his life because he is not my relative. You find out that him doing all these things to me my parents did not receive anything from him not even his R1 but he gives me viruses. When l get sick he will be the one to chase me away saying he doesn't want a grave in his home.

LM: Mmmhh.

89: So, it is best to continue with PrEP.

LM: Alright. 90 do you want to say something? Alright so according to you, what do you think can be done to help people who, who will be using PrEP to continue using it? (Someone clears throat) Because we saw that there are reasons that can make someone to stop it right?

ALL: Mmmhh.

LM: What can be done so that they continue using it no matter how hard it gets? 89.

89: I as 89 l think they say a secret does not help at home. That you should just explain to your person and say "you see my friend it is not that l do not trust you and it is not that l don't trust myself…"

LM: Mmmhh.

89: "I think it would be better for you to let me use PrEP" because you will find out me saying l do not trust myself l will be knowing that l have some people (laughing) and he also has some people.

LM: Alright. 83 your hand was up.

83: I wanted to say l think they should not only tell us. They should also do man's campaigns and sensitize everyone that there is a new program, we have come with PrEP so if you see your wives or children with it just try to understand. I think that will be better.

LM: Mmmhh alright. Ehh 86.

86: I also agree with 83 because situations like these that if you are not in agreement, you will fight and it will be very hard because this type of situation l have seen it back at home our neighbours fighting over family planning pills…

LM: Mmmhh.

86: The woman was taking pills, and the thing is let us say the woman was having an affair with someone's husband, but she also had her husband. So, her husband was in South Africa and then the man she was having an affair with also had his wife but he wanted her to get pregnant for him. So, the woman said how can l have your child while l have my husband at home, she once had a child for this boyfriend so now he wants another child in some man's home. So, the lady went to the clinic and got her pills and continued to drink them. This other day, she used to hide them in the kitchen, this other day l don't know how it happened but the pills fell while they were at the kitchen drinking tea, the man beat her up and threw the pills in the fire (participants laughing) right now the pills are burnt and it is now in the evening…

LM: Yes.

86: She would take them around 7, she now did not have the pills. So, she wrote a letter and sent a young child to run to their neighbour and tell the neighbour that she is facing a such a situation and she can not miss the pills because she knows they can not be missed. So she said to her neighbour may you please help me even if you give me a pill for 1 day, l will tell you the whole story tomorrow because this man is here and he

wants a child. The neighbour said no problem l will come as someone who wants to borrow soda, she told the child to tell the mother that she is coming there. The neighbour came asking for soda. The man was still there sitting in the kitchen, and she said l am asking for soda what…what. When the lady stood up going to look for soda the neighbour then gave the child and said she should go and give her mother, the child then went to give her mother the pill and she drank it. And the next day, she said we will see each other at the well there is something happening while she was lying. In the morning they met at the well and the neighbour said there is no meeting l wanted to give you the pills and this other thing. They are for 1 month. So, these pills since your boyfriend beats you for using the pills its best if we come up with a plan like you dig a hole here besides the well and put these pills in the plastic and close the hole. When you come here to fetch water everyday because we will come you take your pills and drink them. So that lady did that, that is how she lives now she will go to the well and drink her pills just because the boyfriend does not even want to listen, he wants a child.

LM: And what if it is now PrEP.

86: So now when it is PrEP at least it will be better if you have a friend like maybe 89 is my friend who l will give the pills to, to keep them for me l will come and drink them from you because if l go with them at home l will be beaten.

LM: Alright. Let us move forward. Thank you. Let us go to 3. Let us look at the third play. Can you please act. Okay, Princess and who by the way? And Peppa. (Child talking) (people moving around)

Role Play 3

XXX: *Hello.*

ALL: *Hello.*

XXX: *We are good.*

XXX1: *Hello friend.*

XXX2: *Hello.*

XXX1: *Are you okay?*

XXX2: *I am good.*

XXX1: *I am okay. I am here to educate you about PrEP, or you know it?*

XXX2: *Ahh what is PrEP?*

XXX1: *PrEP are pills that are taken to protect yourself from getting the HIV virus.*

XXX2: *Ahh so how can l get those pills my friend since l love my man and also have a lot of them.*

XXX1: *My friend PrEP is this pill that l just got from the doctor. You see how fresh l am.*

XXX2: *Yes.*

XXX1: *1, my friend l love my boyfriends. 2, you see that one who has a BM? Ahh they always tell me about him that he is sick but l…*

XXX2: *You are drinking it*

XXX1: *Yes, l am drinking it.*

XXX2: *Does that mean if l also take it, it will work well?*

XXX1: *Yes friend. You will be beautiful like me. You will never get sick.*

XXX2: *So where will l get them my friend, l feel like l have to look for these pills?*

XXX1: *For you to get them my friend you have to get in touch with health people.*

XXX2: *Alright.*

XXX1: *When they give you, they explain to you, teach you how you use these pills the right way…*

XXX3: *Let me tell you my they say this pill my friend when your partner calls you, you have to take it 7 days before…*

XXX2: *Okay.*

XXX3: *Before...*

XXX2: *If I take it today and have sex with him, does it…*

XXX3: *You will be late my friend. 7 days drinking it. Today you drink it, tomorrow you drink. Till the 7 days.*

XXX2: *When he goes l stop them?*

XXX3: *No, you do not stop. 24 days still drinking them.*

XXX2: *Okay.*

XXX3: *When you know my friend that you have got someone else, you do not stop these pills.*

XXX2: *So, tomorrow l will not go to school.*

XXX3: *Yes.*

XXX: *Thank you, our play ends here.*

LM: Thank you for the play (someone coughing). About Peppa and Sky right.

ALL: Yes.

LM: So, is there any program that was brought up that we can say PrEP is found there, they spoke about it right? So, what do you think about this program that was brought by this group? What do you think about this program? (Background chattering) So which is a good program? That you can say if such was created for young women to get PrEP? Which program are you thinking of? (silence) We are looking at PrEP program.

ALL: Mmmhh.

LM: You talked about that the car comes here that is a program, right?

ALL: Mmmhh.

LM: So we want you to think of a program that you think is good. That you see as a good and should be done. (Everyone laughs) (someone coughs) looking at important things that you think if there are there in PrEP program things will go well. That if this was there at the PrEP program this would be good. 89.

89: Okay as 89. Not teaching (someone coughing) in our DREAMS program I think if PrEP is always available, we will not have chances of getting the HIV virus because if we

closely look at this thing it is good if it is that they come to where we are and give us PrEP…

LM: Mmmhh.

89: We are not the ones who go to them like, l heard 87 when we were talking about the ring that maybe you find out that we took the ring and now the ring has expired and there is no one to remove it…

LM: Mmmhh.

89: For example, some of us had jadelle inserted here at the clinic, ehh at times at the clinic (participants laughing) you insert the jadelle (someone coughing) she will be inserted at the clinic. Then you find out that that jadelle is giving her problems and she does not want it anymore, when it is time to go to the clinic, she hears that the nurse that can remove the jadelle is not around she is in Plumtree. The time when you were inserted that jadelle they explained to you that jadelle will work for you and if you want to remove it you come back to what, to get it removed. When you go to the clinic you will hear that the nurse who is responsible for that is not available now you are supposed to go to Plumtree. When you go to Plumtree you will hear that more money is needed, and you do not have that money. So, if DREAMS keep coming and MAC l think it is better by that PrEP will always be available and if we know that the DREAMS car comes every month we will know that our health is good.

LM: So, the person that should give you, what type of person is that?

XXX: Type of person like?

LM: Ehh it is important (sound of a chair being pulled) let us say maybe it is a nurse, do you want it to be someone who sells at the store, you want to be (participants laughing) 87.

87: We want it to be a nurse who is friendly not the one who will be in moods who when you are trying to explain your problems, she shouts at you and then you feel out of place.

LM: Mmmhh alright. You said a friendly nurse, right?

ALL: Mmmhh.

LM: Yes, with a smiling face not the one who will be…mmmhh 89.

89: Okay ehh l wanted to say that and that we want the people who come to give us these services to be people with love, who knows how to smile.

LM: Mmmhh.

89: Someone will judge me because of my body not knowing that l am grown. Then they will shout at me that at your age you now have sex but l already have 2 children.

LM: Mmmhh.

89: Yes so we just want a sweet person.

LM: Alright. Yes, what about others? Looking at this place also, the place that we once spoke about that the person giving PrEP should be like what? (silence) Should it be given together with other services like family planning? What do you think about this? 90.

90: Yes, so that when others hear that there is family planning (door making noise) (noise of a chair being pulled) then they will say there other services together with PrEP.

LM: Okay. Alright looking at, someone once spoke about, l think it is 83 who spoke saying that everyone should be educated about PrEP right. Yes, so here ehh what do you think is the role of men in the use of PrEP, PrEP program? What could be the role of men? Looking at our sexual partners. (Door closing) Mmmhh 80/

80: I think if he knows about PrEP, he is the other person who could help me with what, with not forgetting to drink the that PrEP and also to remind me that they are about to finish so go to the hospital. I think that is their role if they have the information.

LM: Mmmhh. What about others? What is the role of man in using PrEP programs? What could it be? It could be, like here that he would be encouraging you (noise of chairs being pulled) could it be about how your relationship is? That you are really in love or what? (silence) what are they supposed to do when looking at the PrEP program? (Noise of chairs being pulled) yes.

XXX: I had a question here that are men allowed to take PrEP?

LM: Men can also take it but here we are looking at adolescent girls (noise of chairs being pulled) and young women.

XXX: It will be better if you do not hide it from your person that you live with like us who have boyfriends. You tell him because he will be seeing you always going to the DREAMS car maybe he will tell me he does not want to see me taking the pills because they now know that if they see the DREAMS car it has come with family planning pills. Then l will have to explain to him that it is not about birth control…

LM: Mmmhh.

XXX: There is a new program that has come of PrEP, a pill that works like this and that but l think we also have challenges that when you tell your partner that you are taking the pills to protect yourself from getting the HIV virus he becomes a very dangerous wheel. (Everyone laughs)

LM: How would he be here? (Participants laughing)

XXX: He will be very fast from speed 60 (laughing) to 120.

LM: (Laughing) Ahh alright. Ahh so moving forward we a planning on having a survey on adolescent girls and young women who have had sex right so that we find out how we can come up with new ways to give PrEP programs that are suitable for adolescent girls and young women. These are some of the programs that we want to come up with so that we see where people can get PrEP (someone coughing) in comfortable and private places right?

ALL: Mmmhh.

LM: So ehh when can we find young women if we were to come and give people PrEP or coming to do a PrEP program where can we find young women? Yes 83.

83: We have DREAMS groups that we created, like we will be having sessions. So, we can maybe let us say we are having our session in any day we can combine everything and meet with MAC people, they will come and give their services…

LM: Mmmhh.

83: There and there.

LM: Alright. So, do you think these adolescent girls and young women will come to do our survey?

ALL: Yes.

LM: Mmmhh. What can be done so that they will come? If we are saying they should come and do this survey what can be done for them to come? Mmmhh 84.

84: We used to tell that there will be biscuits and Maheu they come (everyone laughing) l do not want to lie, it will be full. They will come so at least those who are coming if they come with something l think they will be many.

LM: Mmmhh.

84: Because they want to benefit.

LM: Okay. Ahh alright. 89.

89: Okay l think the other thing that makes us to have that thing like, maybe when someone sees me wearing a hat written DREAMS, then they ask where l got it and l tell her that there were DREAMS people asking questions and l got it. Next time if we call them or they call us to come someone would want also…

LM: Okay.

89: Because she will no longer feel the sun.

LM: It will hit her (chuckles) alright ahh is there anyone else who wants to add? (silence) So we would like to test for STIs that we call Sexual Transmitted Diseases. This is part of the survey, it is not what we are doing now. Ahh so it will include these young women taking samples or samples in their private parts right.

ALL: Yes.

LM: Vagina. Ehh that we will test to see if they have STIs. So, do you think young women will be comfortable to take samples of their private parts?

XXX: Let us see others.

LM: Is there anyone who has another opinion? Let us see 89.

89: As 89 l think if you are doing it yourself it is better, than being taken by someone else. Ahh that is hard. And then may l please ask that the thing that you are going to use to test yourself will it only test you or it will also be used on others? Because l once stopped checking my womb some day because l had seen that thing they use is used in lot of people. (laughing) So that is why l ended being scared and said not this. So, l would like to see if that thing is only used on me.

LM: Mmmhh. Okay.

89: Yes.

LM Ahh alright. Yes, it would work like, each person will have their kit that they would use and throw away after using it, it does not work twice. So that is what will make someone comfortable if they know that she will use it alone and throw it away after.

89: Yes, because some other time l got scared to test my womb because l had thought that the same thing, they used to check someone else they are going to use it on me because l had tried to peek inside and l saw it and when l got inside l saw it again.

LM: What about others? Do you think they will be comfortable to take the samples alone? Taking their samples? 91. (everyone laughing)

91: When it comes to people taking their own samples l will not advice it a lot because some do not know how the cervix looks like. Just like, how the cervix looks like, how it feels like l only got to know it recently when l was taught about it. So, from this testing they will be taking dirt from the womb?

LM: No, we are mainly looking at the inside. Ehh you will put it inside yes and turn it around the cervix, but you can also…you don't reach there anyways.

91: Ahh if it is like that l think it is comfortable…

LM: It just doing it ehh not too far.

91: I think it will be easy to test ourselves that way.

LM: 88.

88: I think it is better because as girls here some will be shy to be tested by other people or those who are helping, they will be shy because maybe sometimes they will not want to be tested.

LM: Mmmhh alright 80.

80: Okay l want to help 91, l was thinking that if they say someone must test themselves and put a hand, you do not put your hand to the point that you will reach the intestines (everyone laughing) they just want that thing that a woman even if you are coming from bathing and they say put your hand inside you will find some dirt. So that dirt or that clean thing is what will be wanted. I do not know if that is it.

LM: Mmmhh that is exactly it. What about others, what do we say, 82 what do you say about this? Do you think they will be comfortable to test themselves?

82: Yes, we will be comfortable doing it ourselves. Can l ask after testing yourself where do you take it?

LM: Ehh there will be a procedure for that.

82: Okay. We will be very comfortable.

LM: Mmmhh so here a person has taken their samples, what you do is to test yourself being told how to do it. When you are done a nurse will take it and pack it then it will be tested right. Mmmhh 89.

89: Okay may l please ask l always get confused a bit about PrEP l will take you back a bit. I have seen two methods being used in testing blood…

LM: Mmmhh.

89: There is self-test, then there is that of injection, to be injected…

LM: Mmmhh.

89: So, if you test blood using self-test, then the self-test says you are negative can you use PrEP?

LM: Alright l will answer you when we are done l have written you down.

89: Yes.

LM: I am doing it so that we move forward. Ahh alright (children playing outside) Umm let us move forward l will come back to you. So, we would give if we had tested, we will give the results of the tests and ehh also help with treatment to those who have viruses related to STIs. Ehh the results should be given to professional health workers. Ehh what are you suggesting on how we can get in touch with the young women who would have been found positive for STIs? Do you think they should go to the clinic or hospital? How should they get their results? Mmmhh 89.

89: I was thinking that we, we get, after testing we get our results…

LM: Yes.

89: Is that maybe we can be called, and each person be given, after testing, behind your box you write your name when the results come you guys will be the one knowing that bad results are these ones and right ones are these ones…

LM: Mmmhh.

89: You should give each person their box because at the clinic l can find a nurse who knows my mother and l do not want my mother to know this thing (laughing) or just seeing XXX at home with his bike. Ahh people will say if you see XXX testing XXX2 it means she is now sick she has HIV.

LM: Is XXX the village health worker?

89: No, he is a doctor at the hospital.

LM: Okay does he do home visits?

89: Yes. If he hears about you…

LM: So (laughing) So do you think people will, where would people want to be treated? STIs to those who would be found positive.

XXX: It will be better if you get to the clinic and ask for a place and be the only ones and not other nurses that we know.

LM: Mmmhh. Alright. Why? (Door making noise) because of that reason you mentioned?

XXX: Yes.

LM: Alright. So, l hope we spoke about how the vaginal ring works, how long does it stay. so, this ring is silicone not metal, it is just silicone. You have seen, what can l compare it to? It shrinks it does not enter like this so that it will go where it is supposed to go. It will be, there is a way how they do it because it is silicone it can come back right. Yes, so what can l say, what do you think (child talking) about long term methods? We said this ring works for the whole month right. So, what do you think about methods that work for a long time to protect HIV? These PrEP methods that work for a long time (silence) how are they? That you will be staying the whole month without going or taking pills. You will be staying the whole month without using anything else, you will only be having the ring. (Door making noise) (silence) How is it using a long-term method? (Child making noise) We once spoke about this, l think this is now a recap to see that we spoke about everything.

XXX: Just that we had asked that if you have started with the ring, will your partner not move it?

LM: We will talk about moving (everyone laughing) Ehh alright is there anyone else who want to say something? (silence) Ahh if there is no one else l will ask for questions ahh if there are questions about this discussion that we just heard you can ask. (silence) you do not have any questions?

XXX: If there are no questions you can now give us a vote of thanks.

LM: Yes, if there are no questions ahh my questions are finished. We are at the end of our conversation. Ehh l would like to thank you for your time that you took to have this discussion with us. Thank you. (Clapping of hands)

The End.
