# Supplementary material for: Perceptions of pre-exposure prophylaxis among sexually active adolescent girls and young women in Zimbabwe–A qualitative study
Source: PLOS Glob Public Health. 2025 Dec 2;5(12):e0005396. doi: 10.1371/journal.pgph.0005396 (PMC12671731; doi:10.1371/journal.pgph.0005396)
Supplement: Attachment — Submitted filename: Responses to Reviewers.docx [file pgph.0005396.s005.docx]

Dear Dr Guillaume Fontaine

Thank you for giving us the opportunity to submit a revised draft of the manuscript for publication in PLOS Global Public Health. We appreciate the time and effort that you and the reviewers dedicated to providing valuable feedback on our manuscript and we are grateful for the insightful comments. We have incorporated most of the suggestions made by the reviewers in the manuscript and below in blue are point-by-point responses to the editor’s and reviewers’ comments.

**Editor’s comments**

First, consider selecting and clearly articulating a qualitative or implementation-science framework such as the Social Ecological Model or the Theoretical Domains Framework to organise and interpret your themes.

**Response:** Thank you for pointing this out, we had structured our work according to the social ecological model, but as you rightly pointed out we had not included it in our Methods and Results section explicitly. We have now introduced the Social Ecological Model in the Methods section as follows (for tracked see lines 229-246, for clean version see lines 213-230)

**Theoretical Framework**

There is growing recognition in the literature that, besides individual behaviour, broader environments in which people live and work in largely contributes to their decision-making in utilising health services. The social ecological model (SEM) illustrates interactions between characteristics of the individual, community and the environment [41] that influences health behaviour. This framework recognizes that health experiences and outcomes are often influenced by characteristics situated within and beyond the individual [42, 43]. The SEM levels used are described as; (i) individual (intrapersonal) level such as knowledge, attitude and beliefs; (ii) interpersonal level describing the influence of social interactions on health decisions. This may include the immediate physical environment and social networks in which an individual live including family, friends and peers ; (iii) organisational level such as formal, informal organisations and health provider aspects and (iv) community level where communities may share cultural, ethnic and religious characteristics [44, 45]. We used this construct to understand barriers and facilitators to PrEP uptake among sexually active AGYW. The SEM framework served as a guide addressing how different elements influence PrEP uptake across multiple levels and was incorporated in our analysis. In refining the identified themes, we examined how the themes were linked to the theories and constructs of the SEM framework.

We presented our barriers and facilitators using the Social Ecological Model in the Results section as follows:

“We present the main results on the perceptions of PrEP among AGYW, highlighting important elements that influences PrEP uptake amongst AGYW. Guided by the Social Ecological Model framework, we present these elements as intrapersonal (individual), interpersonal, organizational and community. For tracked see lines 256-260, for clean version see lines 240-243.

Figure 1 shows how the various levels of influence, from individual to community, interact to affect an individual’s decision to use PrEP.”

**Intrapersonal (individual) level**

Issues that served as barriers and facilitators to PrEP uptake at intrapersonal level included: HIV risk perception, PrEP knowledge and oral PrEP packaging. For tracked changes see lines 262-264, for clean version see lines 246-248.

**Interpersonal level**

At interpersonal level lack of support from family and sexual partners were perceived to be barriers to PrEP uptake amongst sexually active AGYW. For tracked changes see lines 395-397, for clean version see lines 358-360.

**Organizational level**

At organizational level concerns related to health facilities and health facilities consultation fees hindered PrEP uptake amongst sexually active AGYW.

For tracked changes see lines 441-443, for clean version see lines 404-406.

**Community level**

At community level the association between PrEP and risky sexual behaviour and PrEP being misconstrued as ART mentioned at the interpersonal level were perceived to be barriers to PrEP uptake amongst sexually active AGYW. For tracked changes see lines 489-491, for clean version see lines 450-453.

Under the Discussion section, see lines 526-527, 568, 597, 621 for tracked changes and lines 465-466, 483, 509, 533 for clean version.

Second, it would also be critical to expand the Methods section, including specifying age ranges and HIV-risk criteria, explaining recruitment through DREAMS and other programmes, justifying the one-year gap before the third province’s focus groups, describing reimbursement and moderator details, and outlining exactly how coding led to your final themes.

**Response:** We have expanded the Methods section as follows.

We had specified the age range: aged between 15-24 years, for tracked changes see line 146, for clean version see line 140.

We specified the HIV risk criteria as follows. “Purposive sampling was done to ensure representation of sexually active AGYW within the ages of 15 and 24 who are enrolled at universities, those who identify as female sex workers, pregnant and lactating AGYW accessing antenatal care and youth centres services. Youth centres serve vulnerable young people, providing them with vocational training skills, education on integrated HIV prevention, sexual and reproductive health rights and sexual and gender-based violence services [41, 42]. According to literature these groups of AGYW are perceived to be at a high risk of getting HIV”. For tracked changes see lines 149-156, for clean version see lines 143-150.

We explained recruitment through DREAMS and other programmes at lines 158-161 for tracked changes and lines 151-154 for clean version: “We engaged these programmes/organisations/ clinics in the recruitment of the FGD participants as they were already working with sexually active AGYW offering them different sexual and reproductive health services. This was done to maintain the confidentiality of their sexual behaviours”.

We justified the one-year gap as follows, “during data analysis discussions it emerged that it would be helpful to get insights from another province (Matabeleland South) where there is higher vulnerability to HIV among AGYW [35].The over one-year gap before the focus group discussions in Matabeleland South was due to unforeseen delays in obtaining required community entry approvals.” For tracked changes see lines 165-169, for clean version see lines 158-162.

We have now added that: “Each participant received $5 as reimbursement for the time they took participating in the FGDs”; see line 213 for tracked changes and line 197 for clean version.

We provided the following moderator details: “The FGDs were moderated by trained female social scientists with experience of conducting FGDs and working with young people”; for tracked changes see lines 185-186 and for clean version see lines 169-170.

We provided more detail on how coding process led to the final themes. “The team discussions and the field notes were used to code the data, to identify specific pieces of content relevant to the research topic and later to develop and refine a coding framework [39, 40]. This was used for coding the transcripts in NVIVO 14. The identified codes were organised into themes connecting related concepts. Themes were then reviewed to ensure understanding of the meaning of each theme and finally refined.” For tracked changes see lines 220, 224-228 and for clean version see lines 204, 207-212.

Third, it would be important to provide essential context on Zimbabwe’s PrEP landscape, including funding sources, eligibility rules, consultation costs, and how the 2024–25 budget crisis may affect access; this material belongs in the Introduction, not buried in the Results.

**Response:** Thanks for pointing this out, we have now provided the following essential context on Zimbabwe’s PrEP landscape, including the funding sources, eligibility rules, consultation costs and how the 2024-2025 budget crisis may affect access to PrEP. “The country’s 2022 guidelines for HIV prevention outline the eligibility rules for PrEP use as follows: i) HIV negative and sexually active AND has any of the following: a) Vaginal or anal intercourse without condoms; b) A sexual partner of unknown HIV status; c) A sexual partner with one or more HIV risk factors; d) A history of sexually transmitted infection by lab testing or self-report or syndromic treatment of sexually transmitted infection; e) Any use of post-exposure prophylaxis (PEP) [19]. In Zimbabwe, the primary donors for HIV programming including PrEP provision at the time of the FGDs were The United States President's Emergency Plan for AIDS Relief and Global Fund [20]. Oral PrEP is accessed through public and not-for-profit private sectors in Zimbabwe [20]. In most health facilities oral PrEP is accessed at no cost, however there are some council-run health facilities that requires an adult to pay a clinic consultation fees, usually $5, before accessing health services [21]. In Zimbabwe, dapivirine vaginal ring and CAB-LA were being rolled out under implementation research to inform policy and scale-up [22, 23], with prioritisation of subpopulation at high risk including AGYW. CAB-LA was being implemented in 15 sites, with 6 being supported through the catalyzing access to new prevention products to stop HIV (CATALYST) and 9 through PEPFAR. However, the CATALYST trial has been stopped due to United States Agency for International Development (USAID) funding cuts [24, 25] as Zimbabwe is amongst the countries that have been recently affected by the USAID funding cuts [26]. In 2025, PrEP services have been severely affected due to heavy reliance on USAID funding, with availability primarily focused on pregnant and lactating mothers [27]. For Zimbabwe this has already affected access to PrEP to non-pregnant or lactating sexually active AGYW at risk of acquiring HIV as most organisations providing HIV services have stopped working. In particular the Determined, Resilient, Empowered, AIDS-Free, Mentored and Safe (DREAMS) program has been halted [26, 28]. DREAMS served some African countries representing over half of all infections occurring among AGYW globally including Zimbabwe. Its core aim was to address structural drivers to HIV including poverty, gender inequality, sexual violence, and lack of education and promoting HIV prevention (including PrEP access) amongst AGYW [29, 30]. The government of Zimbabwe is making efforts to mitigate the impact of reduced international funding for HIV programs including PrEP [31, 32]. World Health Organisation has approved Zimbabwe, along with other African countries including South Africa, Zambia, Uganda, Tanzania, Nigeria, Kenya, Botswana to begin receiving lenacapavir by 2026 [32, 33]..” For tracked changes see lines 84-115 and for clean version see lines 81-112.

Fourth, revise the participant table to include risk factors, partner status, and prior or current PrEP use, and present any motivations or facilitators alongside the barriers you already report.

**Response:** We are unable to provide the requested data as we did not collect data on risk factors, partner status. For history of PrEP use, we had included data for ever used PrEP only, we have now specified that one person was currently using PrEP.

Finally, please streamline the writing: spell out most acronyms, correct spacing and phrasing issues, reference policy or efficacy claims, and ensure the Data Availability statement does not jeopardise participant confidentiality.

**Response:** We have spelt out acronyms and reduced the use of acronyms to help increase clarity among an international audience.

SRH-Sexual and Reproductive Health (For tracked changes see lines, 44,157, 318-320, 322-323, 612-613, 629, 661 and for clean version see lines 43,150,302-304,306, 524-525,541, 571). We have also spelt out the following acronyms, OI-Opportunistic Infections (For tracked changes see line, 635 and for clean version see line 546), HCW-Health care worker (For tracked changes see lines, 445, 447, 534, 622, 624, 627, 658 and for clean version see lines 407, 409, 472,533-536,538,568) STI- (For tracked changes see lines 465 and clean version see line 427), KP-Key populations (For tracked changes see lines, 304-305, 307 and clean version see lines 288-289, 291), SAYWHAT-(For tracked changes see line 309 and clean version see lines 292-293), ZMoHCC-Zimbabwe Ministry of Health and Child Care (For tracked changes see line 634 and clean version see line 545), LEN-Lenacapavir (For tracked changes see line 595 and clean version see line 507), ASRH-Adolescent and Youth Sexual and Reproductive Health Strategy (For tracked version see line 616 and clean version see lines 527-528).

We have corrected the spacing throughout the manuscript.

Checked reference policy and efficacy claims

We have made a change to the data availability statement; it now reads as follows.

**“Availability of data**

Unidentifiable data will be submitted as supplementary information”

We have added the inclusivity in global research statement, for tracked changes see lines 138-140 and for clean version see lines 131-134.

**Reviewer 1 Comments: Thank you for the opportunity to review this manuscript reporting on AGYW's perceptions of accessing PrEP in Zimbabwe. The use of scenarios to elicit discussion worked well for this study.**

1.The paper would be strengthened by use of theoretical framework or lens with which the data can be interpreted.

**Response:** Thank you for pointing this out, we had structured our work according to the social ecological model, but as you rightly pointed out we had not included it in our Methods and Results section explicitly. We have now introduced the Social Ecological Model in the Methods section as follows (for tracked changes see lines 229-246, for clean version see lines 213-230)

There is growing recognition in the literature that, besides individual behaviour, broader environments in which people live and work in largely contributes to their decision-making in utilising health services. The social ecological model (SEM) illustrates interactions between characteristics of the individual, community and the environment [41] that influences health behaviour. This framework recognizes that health experiences and outcomes are often influenced by characteristics situated within and beyond the individual [42, 43]. The SEM levels used are described as; (i) individual (intrapersonal) level such as knowledge, attitude and beliefs; (ii) interpersonal level describing the influence of social interactions on health decisions. This may include the immediate physical environment and social networks in which an individual live including family, friends and peers ; (iii) organisational level such as formal, informal organisations and health provider aspects and (iv) community level where communities may share cultural, ethnic and religious characteristics [44, 45]. We used this construct to understand barriers and facilitators to PrEP uptake among sexually active AGYW. The SEM framework served as a guide addressing how different elements influence PrEP uptake across multiple levels and was incorporated in our analysis. In refining the identified themes, we examined how the themes were linked to the theories and constructs of the SEM framework.

We presented our barriers and facilitators using the Social Ecological Model in the Results section as follows:

“We present the main results on the perceptions of PrEP among AGYW, highlighting important elements that influences PrEP uptake amongst AGYW. Guided by the Social Ecological Model framework, we present these elements as intrapersonal (individual), interpersonal, organizational and community. For tracked changes see lines 256-260, for clean version see lines 240-243.

Figure 1 shows how the various levels of influence, from individual to community, interact to affect an individual’s decision to use PrEP.”

**Intrapersonal (individual) level**

Issues that served as barriers and facilitators to PrEP uptake at intrapersonal level included: HIV risk perception, PrEP knowledge and oral PrEP packaging. For tracked changes see lines 262-264, for clean version see lines 246-248.

**Interpersonal level**

At interpersonal level lack of support from family and sexual partners were perceived to be barriers to PrEP uptake amongst sexually active AGYW. For tracked changes see lines 395-397, for clean version see lines 358-360.

**Organizational level**

At organizational level concerns related to health facilities and health facilities consultation fees hindered PrEP uptake amongst sexually active AGYW.

For tracked changes see lines 441-443, for clean version see lines 404-406.

**Community level**

At community level the association between PrEP and risky sexual behaviour and PrEP being misconstrued as ART mentioned at the interpersonal level were perceived to be barriers to PrEP uptake amongst sexually active AGYW. For tracked changes see lines 489-491, for clean version see lines 450-453.

Under the Discussion section, see lines 526-527, 568, 597, 621 for tracked changes and lines 465-466, 483, 509, 533 for clean version.

2. Given the drastic and appalling changes to global funding this year, is this currently happening (see lines 315-316)? It would be useful for the authors to add some contextualisation re current funding environment within the manuscript.
**Response:** We have provided the following essential context on Zimbabwe’s PrEP landscape, in particular on how the 2024-2025 budget crisis may affect access to PrEP. “In Zimbabwe, the primary donors for HIV programming including PrEP provision at the time of the FGDs were The United States President's Emergency Plan for AIDS Relief and Global Fund [20]. Oral PrEP is accessed through public and not-for-profit private sectors in Zimbabwe [20]. In most health facilities oral PrEP is accessed at no cost, however there are some council-run health facilities that requires an adult to pay a clinic consultation fees, usually $5, before accessing health services [21]. In Zimbabwe, dapivirine vaginal ring and CAB-LA were being rolled out under implementation research to inform policy and scale-up [22, 23], with prioritisation of subpopulation at high risk including AGYW. CAB-LA was being implemented in 15 sites, with 6 being supported through the catalyzing access to new prevention products to stop HIV (CATALYST) and 9 through PEPFAR. However, the CATALYST trial has been stopped due to United States Agency for International Development (USAID) funding cuts [24, 25] as Zimbabwe is amongst the countries that have been recently affected by the USAID funding cuts [26]. In 2025, PrEP services have been severely affected due to heavy reliance on USAID funding, with availability primarily focused on pregnant and lactating mothers [27]. For Zimbabwe this has already affected access to PrEP to non-pregnant or lactating sexually active AGYW at risk of acquiring HIV as most organisations providing HIV services have stopped working. In particular the Determined, Resilient, Empowered, AIDS-Free, Mentored and Safe (DREAMS) program has been halted [26, 28]. DREAMS served some African countries representing over half of all infections occurring among AGYW globally including Zimbabwe. Its core aim was to address structural drivers to HIV including poverty, gender inequality, sexual violence, and lack of education and promoting HIV prevention (including PrEP access) amongst AGYW [29, 30]. The government of Zimbabwe is making efforts to mitigate the impact of reduced international funding for HIV programs including PrEP [31, 32]. World Health Organisation has approved Zimbabwe, along with other African countries including South Africa, Zambia, Uganda, Tanzania, Nigeria, Kenya, Botswana to begin receiving lenacapavir by 2026 [32, 33].” For tracked changes see lines 84-115 and clean version see lines 81-112.

Also, please read through manuscript and correct spacing issues - sometimes two spaces between words, a space between a word and punctuation, or no spacing between word and reference.

**Response:** We have corrected spacing throughout the manuscript.

Other comments/suggestions which I believe, once addressed, will strengthen the quality of the manuscript.

Define ages of AGYW in first sentence of both Abstract and Introduction.
**Response:** Defined the ages of AGYW in both the abstract and introduction as follows, “aged 15-24years”. For tracked changes see lines 19 & 54 and clean version see lines 18 & 53.

Lines 26-27: What were the risk factors among participants - e.g., sex and/or injecting drug use, other? Were all participants sexually active or at risk of HIV exposure?

**Response:** Given the abstract word count, we felt that there was no space to provide an explanation. We have added the following explanation of the risk factors among participants, “Purposive sampling was done to ensure representation of sexually active AGYW within the ages of 15 and 24 who are enrolled at universities, those who identify as female sex workers, pregnant and lactating AGYW accessing antenatal care and youth centres services. Youth centres serve vulnerable young people, providing them with vocational training skills, education on integrated HIV prevention, sexual and reproductive health rights and sexual and gender-based violence services [41, 42]. According to literature these groups of AGYW are perceived to be at a high risk of getting HIV. We engaged these programmes/organisations/clinics in the recruitment of the FGD participants as they were already working with sexually active AGYW offering them different sexual and reproductive health services. This was done to maintain the confidentiality of their sexual behaviours. For tracked changes see lines 149-155, 157-161 and clean version see lines 143-154.

Line 29: How many participants enrolled in either of these?

**Response:** In the abstract it says “92 (8-12 per group) sexually active AGYW”; we felt that it was not possible to include in the abstract the number of participants to each FGD due to the word count. We have added the following breakdown of the participants in the Results section. “We recruited AGYW who were enrolled or engaging with the National Key Populations program (2 FGDs-16 AGYW), the DREAMS program (2 FGDs -21 AGYW), universities (1 FGD- 9 AGYW), youth centres (1 FGD-12 AGYW) and antenatal care clinics (3 FGDs-34 AGYW).” For tracked changes see lines 250-253 and clean version see lines 234-237.

Lines 30-31: It would be helpful to know their risks factors.

**Response:** We felt that given the abstract word count there was no space to provide an explanation. We have provided more clarity in the Methods section: “, “Purposive sampling was done to ensure representation of sexually active AGYW within the ages of 15 and 24 who are enrolled at universities, those who identify as female sex workers, pregnant and lactating AGYW accessing antenatal care and youth centres services. Youth centres serve vulnerable young people, providing them with vocational training skills, education on integrated HIV prevention, sexual and reproductive health rights and sexual and gender-based violence services [41, 42]. According to literature these groups of AGYW are perceived to be at a high risk of getting HIV. We engaged these programmes/organisations/clinics in the recruitment of the FGD participants as they were already working with sexually active AGYW offering them different sexual and reproductive health services. This was done to maintain the confidentiality of their sexual behaviours. For tracked changes see lines 149-156, 158-161 and clean version see lines 143-154.

Lines 87-89: Specify "in Zimbabwe" in this sentence

**Response:** We have added “in Zimbabwe”, see line 125 for tracked changes and line 121 for clean version.

Lines 106-107: These are 4 very diverse groups - were FGDs held with participants from specific demographics or were AGYW from across these 4 groups included together in FGDs?

**Response:** We have added this sentence at lines 180-182 for tracked changes and lines 164-166 for clean version: “Each FGD was conducted with a group of AGYW with similar demographics, for example young women selling sex participated in their own FGD separate from the young AGYW recruited from the antenatal clinics.”

Lines 110-112: These needs explanation. Why was there nearly a year gap between completion of FGDs in the two provinces and conducting two more FGDs in the third province? Was the third province added later? If so, why?

**Response:** We have now provided an explanation: “During data analysis discussions it emerged that it would be helpful to get insights from another province (Matabeleland South) where there is higher vulnerability to HIV among AGYW. The over one-year gap before the focus group discussions in Matabeleland South was due to unforeseen delays in obtaining required community entry approvals. For tracked changes see lines 165-169 and clean version see lines 158-162.

Lines 112-120: This should be moved to sub-section above - Study Population, Location and Sampling

**Response**: We felt that the number of participants in each FGD needed to go in the Results not in the Methods. This is so as so we did not have this knowledge before the study was conducted. For tracked changes see lines 250-253 and clean version see lines 234-237 with the following moved paragraph, “We recruited AGYW who were enrolled or engaging with the National Key Populations program (2FGDs-16 AGYW), the DREAMS program (2 FGDs - 21 AGYW), universities (1 FGD - 9 AGYW), youth centres (1 FGD - 12 AGYW) and antenatal care clinics (3 FGDs - 34 AGYW).”

Lines 135-136: Who are Shona and Ndebele? It appears they are not included in the author list.

**Response:** Shona and Ndebele are the two most common spoken languages in Zimbabwe. We have specified the Shona (KC) and Ndebele (LM) moderators, see line 210 for tracked changes and line 194 for clean version. We have added the Ndebele moderator, who was not included in the author list, as co-author. See line 5 for both tracked changes and clean version.

Line 148 "four qualitative researchers" - Are they co-authors? If yes, include their initials.
**Response:** We have added the initials of all the four qualitative researchers as follows, “All FGD audio records were transcribed verbatim and translated to English by four qualitative researchers (KC, LM, FM & SM). See lines 215-216 for tracked changes and lines 199-200 for clean version. We have also included them as co-authors, see line 5 for both tracked changes and clean version.

Table, History of PrEP Use "Not asked" - Is this not asked or didn't answer? If not asked, does that mean question was not asked in 2 or more FGDs. Was this intentional? I acknowledge that sometimes an interviewer can forget to ask but curious if there was a considered reason for not asking.

**Response:** For history of PrEP use, we started collecting the data on PrEP use after conducting 3 FGDs. After iterative discussions the team saw the need to have information on PrEP use. Hence a total of 36 participants from 3 FGDs were not asked the question of PrEP use.

Lines 157-159: What proportion of participants indicated they were involved in this?
**Response:** We do not have data on the proportion of participants who were involved in condom-less transactional intergenerational sexual relationships. We did not ask personal experiences as the FGDs were meant to provide generalised views. We have included that relationships with blessers were believed to be mostly associated with sexually active AGYW in colleges and those who identify as female sex workers. See lines 270-271 for tracked changes and lines 254-255 for clean version.

Add to participant demographics how many participants had previously used (or were currently using) PrEP.

**Response:** We have provided the following data on the participants currently using PrEP.

| Currently using PrEP | 1 (1%) |
| --- | --- |

See demographics table, history of PrEP use. We had provided data on how many participants have ever used PrEP before.

Line 193 "prove": This kind of statement should be referenced.

**Response:** We have referenced the statement, “Cowan FM, Musemburi S, Matambanadzo P, Chida P, Steen R, Makandwa R, et al. Using a Programme Science approach to substantially reduce the risk of HIV transmission and acquisition in sex transactions among female sex workers in Zimbabwe. J Int AIDS Soc. 2024;27 Suppl 2(Suppl 2):e26262”, see line 308 for tracked changes and line 292 for clean version.

Line 203: Please provide a reference for this statement.

**Response:** Provided reference for this, “Gyan SE. Passing as “Normal”: Adolescent Girls’ Strategies for Escaping Stigma of Premarital Sex and Childbearing in Ghana. SAGE Open. 2018;8(3):2158244018801421.See line 321 for tracked changes and line 305 for clean version.

Line 378: This is a long list of refs that appear to be superficially included without much substance as to why they are referenced.

**Response:** We have reviewed the list of references, and we were aiming to provide the relevant literature identifying barriers to PrEP uptake amongst AGYW. However, we have noted your concern, and it seems we might have provided many references. We have reduced the number of references from eight to five by removing some references that were reporting similar findings as others and some that were not really outlining the barriers to PrEP uptake. See line 560 for tracked changes and line 476 for clean version.

Lines 423-424: This is personal preference, but I believe this sentence could be strengthened through modifying language - perhaps, "Our results align with findings of other studies, indicating a strong need to increase the efforts being done to resolve these barriers"

**Response:** Yes, we do agree that it is a personal preference and thank you for the suggestion. We have made the following change, “Reflecting, our results align with findings of other studies, indicating a strong need to increase the efforts being done to resolve these barriers” See lines 637 for tracked changes and lines 548-549 for clean version.

**Reviewer #2: Thank you for the opportunity to review this article. Please see below some general and specific comments that I hope will be of assistance to the authors to help increase clarity for international readers.**

Entire manuscript: Would remove "factors identified" or use of "factors" which is more often identified with quantitative research/epidemiology analysis

**Response:** We have removed factors, identified or use of factors in most parts of the manuscript. For tracked changes see lines 34, 267, 628 and for clean version see lines 32, 251, 540.

Introduction: Might want to rephrase slightly: “older men, who are most likely having risky sexual relationships…” I do not think that an international audience would assume this per se, and so I would recommend that the authors rephrase.

**Response:** We have rephrased the sentence, it now reads as follows; “engagement in intergenerational and/or transactional sexual relationships with older men, who in most cases engage in risky sexual relationships”. For tracked changes see lines 62-63 and for clean version see lines 61-63.

Introduction: “Inadequate schooling” – please elaborate. Does this mean poor sexual health in school? Or fewer educational opportunities. Please rephrase.

**Response:** We have rephrased to low levels of education. For tracked changes see line 66 and lines 64-64 for clean version.

Please provide some (brief; 1-2 sentences) of how PrEP is provided in Zimbabwe – is this through the Global Fund? National Ministry of Health?

**Response:** Provided the following information on how PrEP is being provided in Zimbabwe, “in Zimbabwe, the primary donors for HIV programming including PrEP provision are The United States President's Emergency Plan for AIDS Relief and Global Fund. Oral PrEP is accessed through public and not-for-profit private sectors in Zimbabwe.” For tracked changes see lines 89-92 and lines 86-89 for clean version.

Introduction: Is there any understanding/evidence of why the uptake of PrEP is low? Do people have to co-pay? Is it only available to those considered high-risk for HIV? Has any other research been conducted in Zimbabwe in this area?

**Response:** We have provided some evidence of why PrEP uptake is low, see lines 118-119 for tracked changes and lines 115-116 for clean version. “This is largely due to challenges with oral PrEP adherence, unfriendly youth PrEP services and HIV related stigma.”

We have provided context on the current PrEP landscape in Zimbabwe, see lines 84-119 for tracked changes and lines 81-116 for clean version. The country’s 2022 guidelines for HIV prevention outline the eligibility rules for PrEP use as follows: i) HIV negative and sexually active AND has any of the following: a) Vaginal or anal intercourse without condoms; b) A sexual partner of unknown HIV status; c) A sexual partner with one or more HIV risk factors; d) A history of sexually transmitted infection by lab testing or self-report or syndromic treatment of sexually transmitted infection; e) Any use of post-exposure prophylaxis (PEP) [19]. In Zimbabwe, the primary donors for HIV programming including PrEP provision at the time of the focus group discussions (FGDs) were The United States President's Emergency Plan for AIDS Relief (PEPFAR) and Global Fund [20]. Oral PrEP is accessed through public and not-for-profit private sectors in Zimbabwe [20]. In most health facilities oral PrEP is accessed at no cost, however there are some council-run health facilities that requires an adult to pay a clinic consultation fees, usually $5 before accessing health services [21]. In Zimbabwe, dapivirine vaginal ring and CAB-LA were being rolled out under implementation research to inform policy and scale-up [22, 23], with prioritisation of subpopulation at high risk including AGYW. CAB-LA was being implemented in 15 sites, with 6 being supported through the catalyzing access to new prevention products to stop HIV (CATALYST) and 9 through PEPFAR. However, the CATALYST trial has been stopped due to United States Agency for International Development (USAID) funding cuts [24, 25] as Zimbabwe is amongst the countries that have been recently affected by the USAID funding cuts [26]. In 2025, PrEP services have been severely affected due to heavy reliance on USAID funding, with availability primarily focused on pregnant and lactating mothers [27]. For Zimbabwe this has already affected access to PrEP to non-pregnant or lactating sexually active AGYW at risk of acquiring HIV as most organisations providing HIV services have stopped working. In particular the Determined, Resilient, Empowered, AIDS-Free, Mentored and Safe (DREAMS) program has been halted [26, 28]. DREAMS served some African countries representing over half of all infections occurring among AGYW globally including Zimbabwe. Its core aim was to address structural drivers to HIV including poverty, gender inequality, sexual violence, and lack of education and promoting HIV prevention (including PrEP access) amongst AGYW [29, 30]. The government of Zimbabwe is making efforts to mitigate the impact of reduced international funding for HIV programs including PrEP [31, 32]. World Health Organisation has approved Zimbabwe, along with other African countries including South Africa, Zambia, Uganda, Tanzania, Nigeria, Kenya, Botswana to begin receiving lenacapavir by 2026[32, 33]. Before the recent funding cuts, low PrEP uptake was being witnessed amongst AGYW regionally and locally in Zimbabwe [34], with only 6% of the surveyed sexually active AGYW at risk of HIV found to be on PrEP in some provinces[35]. This is largely due to challenges with oral PrEP adherence, unfriendly youth PrEP services and HIV related stigma [36, 37, 38, 39].

Other research conducted in Zimbabwe in this area has been presented in the Discussion when comparing the findings. Particularly from Zimbabwe though there are limited studies reporting on barriers to PrEP uptake amongst sexually active AGYW, two studies have explored views on barriers to PrEP use amongst AGYW, [36, 59] similar insights on the oral PrEP package and unfriendly health workers were reported. This study provides new insights on the vulnerability of unmarried sexually active AGYW and exposure to high risk of getting HIV. See lines 561-567 for tracked changes and lines 477-482 for clean version.

Introduction: I would suggest revising the last sentence to state the aim of the study. Please also make clear that this is a qualitative study.

**Response:** We have revised the sentence, bringing out the aim of the study and that it was a qualitative study. The sentence now reads as: “The aim of the study was to inform the development of an acceptable, context-relevant program for facilitating uptake of PrEP for AGYW. To achieve the aim of the study we conducted a qualitative study exploring perceptions of sexual HIV risk among AGYW in Zimbabwe and generated broad overviews of PrEP acceptance and barriers”. See lines 122-125 for tracked changes and lines 119-122 for clean version.

Ethical statement: “This was part of a mixed-methods study…” If another section of the study has been published (the quantitative portion), then would cite here.

**Response:** Thank you for the recommendation, the quantitative portion of the study has not been published yet. We have provided reference for an oral and poster presentation, which presented some of the quantitative findings. See line 143 for tracked changes and line 137 for clean changes.

Methods: Were these focus group conducted on online? Please make this clearer (i.e., it’s not clear at first if they were physically conducted in Harare or with people residing in Harare but online).

**Response**: We have provided more clarity about where the FGDs were held. The sentence reads, “all FGDs were held in-person with both the participants and moderator being physically present in each of the province stated above during the discussions.” See lines 148-149 for tracked changes and lines 141-143 for clean version.

Methods: Please explain what is means to be “currently engaged in HIV/SRH programs” – does this mean that the participant is a client of these programs? Or has attended these programs at least once? It’s also not clear what the DREAMS program is. Please clarify for an international audience.

**Response:** We have explained as follows, “We engaged these programmes/organisations/clinics in the recruitment of the FGD participants as they were already working with sexually active AGYW offering them different sexual and reproductive health services. This was done to maintain the confidentiality of their sexual behaviours.” See lines 158-161 for tracked changes and lines 151-154 for clean version.

We have also provided more information about the DREAMS program, “DREAMS served some African countries representing over half of all infections occurring among AGYW globally including Zimbabwe. Its core aim was to address structural drivers to HIV including poverty, gender inequality, sexual violence, and lack of education and promoting HIV prevention (including PrEP access) amongst AGYW.” See lines 106-111 for tracked changes and lines 104-108 for clean version.

Methods: Were participants paid for their time? If yes, please state how much reimbursement/vouchers, etc.

**Response:** We have now stated that each participant received $5 reimbursement for the time they took participating in the FGD, see line 213 for tracked changes and line 197 for clean version.

Methods: Please clarify what the criteria for participation in this study is quite broad? Were all participants at a high risk of HIV and hence, should/would know about PrEP?

**Response:** We had clarified the criteria for participation in the study as follows. “We defined the criteria for participation in the FGDs as sexually active AGYW, defined as having had sex with a man in the past 12 months (with or without a condom).” See lines 144-146 for tracked changes and lines 138-140 for clean version. “Purposive sampling was done to ensure representation of sexually active AGYW within the ages of 15 and 24 who are enrolled at universities, those who identify as female sex workers, pregnant and lactating AGYW accessing antenatal care and youth centres services.” See lines 149-52 for tracked changes and lines 143-146 for clean version. “According to literature these groups of AGYW are perceived to be at a high risk of getting HIV.” See lines 155-156 for tracked changes and lines 149-150 for clean version.

Methods: Are vaginal rings and CAB-LA available in Zimbabwe? If yes, please add to Introduction.

**Response:** We have added this sentence in the manuscript: “In Zimbabwe, dapivirine vaginal ring and CAB-LA were being rolled out under implementation research to inform policy and scale-up [22, 23], with prioritisation of subpopulation at high risk including AGYW. However, the CATALYST trial has been stopped due to United States Agency for International Development (USAID) funding cuts [24, 25] as Zimbabwe is amongst the countries that have been recently affected by the USAID funding cuts [26].” See lines 95-97, 99-102 for tracked changes and lines 92-97 for clean version.

Methods: Who transcribed the interviews? Were the transcripts de-identified?

**Response:** We have provided the following response on the people who transcribed the interviews and explanation on how the transcripts were de-identified. “All FGD audio records were transcribed verbatim and translated to English by four qualitative researchers (KC, LM, FM & SM), then deleted from the recorders. All audio-records and transcripts were de-identified by assigning unique study participant numbers.” See lines 215-217 for tracked changes and lines 199-201 for clean version.

Methods: Did the focus groups follow an interview guide? If yes, what sort of questions were on the interview guide?

**Response**: Yes, the focus group discussions followed an interview guide, see line 201 for tracked changes and line 185 for clean version. The interview guides are in Appendix A. We have added a summary of the questions that were on the interview guide, that reads as follows: “The questions centred around perception of HIV risk, PrEP knowledge and understanding, identifying barriers and facilitators to PrEP uptake and the preferred PrEP service delivery models amongst sexually active AGYW.” See lines 210-204 for tracked changes and lines 186-188 for clean version.

Methods: The analysis is not clear. The authors describe the coding process and discussions, but how did the authors go about the analysis? How were the results grouped into themes?

**Response:** We have provided more information on the analysis process. “The team discussions and the field notes were used to code the data, to identify specific pieces of content relevant to the research topic and later to develop and refine a coding framework [39, 40] which was used for coding the transcripts in NVIVO 14. The identified codes were organised into themes connecting related concepts. Themes were then reviewed to ensure understanding of the meaning of each theme and finally refined.” See lines 224-225, 227-228 for tracked changes and lines 207-212 for clean version.

Results: It’s not clear from the Table. Did the participants have a stable partner (e.g., boyfriend)?

**Response:** Thank you for seeking clarity on this, we noted that we only collected data on whether one is married or not, therefore we do not have data on the single status if they have a stable partner or not. We have acknowledged the importance of collecting such data and we will ensure to collect for future similar studies.

Results: “transactional sexual relationships” – Could the authors briefly elaborate on this a bit more. Is this a result from the sex workers? Or from all participants? Or are the participants in relationships with the “blessers”?

**Response:** Participants were sharing generalised views basing their responses on what they have seen happening in their communities, with their peers. We have provided more information on the types of AGYW mostly believed to be associated with the blessers. “Relationships with blessers were believed to be mostly associated with sexually active AGYW in colleges and those who identify as female sex workers.” See lines 270-271 for tracked changes and lines 254-255 for clean version.

Results: “This lack of understanding was most pronounced among AGYM…” – Among the 5 participants currently pregnant? Please explain.

**Response:** We have made it clear that the lack of understanding was most pronounced amongst breastfeeding and pregnant AGYW recruited through the antenatal and postnatal clinics. See line 296 for tracked changes and line 280 for clean version.

Results: Would spell out SRH throughout (and avoid the use of most acronyms to be help increase clarity among an international audience).

**Response:** Thank you for recommendation, we have spelt out SRH-Sexual and Reproductive Health (For tracked changes see lines, 44,157, 318-320, 322-323, 612-613, 629, 661 and for clean version see lines 43,150,302-304,306, 524-525,541, 571). We have also spelt out the following acronyms, OI-Opportunistic Infections (For tracked changes see line, 635 and for clean version see line 546), HCW-Health care worker (For tracked changes see lines, 445, 447, 534, 622, 624, 627, 658 and for clean version see lines 407, 409, 472,533-536,538,568) STI- (For tracked changes see lines 465 and clean version see line 427), KP-Key populations (For tracked changes see lines, 304-305, 307 and clean version see lines 288-289, 291), SAYWHAT-(For tracked changes see line 309 and clean version see lines 292-293), ZMoHCC-Zimbabwe Ministry of Health and Child Care (For tracked changes see line 634 and clean version see line 545), LEN-Lenacapavir (For tracked changes see line 595 and clean version see line 507), ASRH-Adolescent and Youth Sexual and Reproductive Health Strategy (For tracked version see line 616 and clean version see lines 527-528).

Results: “Unfriendly and unprofessional health care worker attitudes…” “Judgmental, unfriendly and unprofessional attitudes by some HCWs…” Is this based on experience or are the participants anticipating that when accessing PrEP, they would be judged. Please clarify.

Response: We have clarified this statement as follows, “This was mostly reported by AGYW based on their peers or their experiences with health care workers when accessing PrEP or sexual reproductive health services at public health facilities.” See lines 449-451 for tracked changes and lines 411-413 for clean version.

Results: “pay a consultation fee of $5…” – I think this should be mentioned in the Introduction instead (to help give the reader some context). So, is PrEP only free in certain public health facilities and not others?? How many donor funded institutions are in the country? How many people do they service?

**Response:** We have now provided more context in the Introduction, including explaining the cost of accessing PrEP in Zimbabwe. “In Zimbabwe, the primary donors for HIV programming including PrEP provision are The United States President's Emergency Plan for AIDS Relief and Global Fund. Oral PrEP is accessed through public and not-for-profit private sectors in Zimbabwe. In most health facilities oral PrEP is accessed at no cost, however there are some council-run health facilities that requires one to pay a clinic consultation fees where an adult normally pays an equivalent of $5 before accessing health services.” See lines 89-95 for tracked changes and lines 86-92 for clean version.

Discussion: “Unmarried AGYM…had the least knowledge…” Least knowledge of PrEP? Please provide explain.

**Response:** We have further explained that unmarried AGYW who were not attending HIV/SRH programs had the least knowledge of PrEP and HIV risk. See line 531 for tracked changes and line 469 for clean version.

Discussion: “This study contributes to the growing literature…” Is this the first study on barriers to PrEP that has taken place in Zimbabwe? If yes, would mention here.

**Response:** We have indicated that even though there limited studies there are other studies that have been done in Zimbabwe exploring views on the barriers to PrEP uptake. Particularly from Zimbabwe though there are limited studies reporting on barriers to PrEP uptake amongst sexually active AGYW, two studies have explored views on barriers to PrEP use amongst AGYW, [36, 59] similar insights on the oral PrEP package and unfriendly health workers were reported. This study provides new insights on the vulnerability of unmarried sexually active AGYW and exposure to high risk of getting HIV. See lines 561-567 for tracked changes and lines 477-482 for clean version.

Discussion: “rattling sound from the bottles”? Was this mentioned in the Results? Please clarify.

**Response:** Thank you for picking this up. No this was not mentioned in the results section. We have added this sentence in the results section: “There were also concerns of the rattling sound of the oral PrEP packaging that draws unneeded attention making it difficult for one to take the pills discreetly”. See lines 355-357 for tracked changes and lines 319-320 for clean version.

Discussion: “discreet, fun and engaging” – Could also state that it’s important to change to prevent stigmatization from others (and cite research to indicate this the harms of stigma towards people with HIV and/or people who take PrEP).

**Response:** Thank you, we have added this sentence along with the reference: “It is important to differentiate packaging of PrEP from ART and make oral PrEP packaging to being more discreet, fun and engaging to increase its uptake amongst the AGYW, to preventing stigmatization”. See lines 582-583 for tracked changes and lines 495-497 for clean version.

Discussion: Would also spell out HCWs throughout the manuscript. Would consider reducing the use of most acronyms in the article

**Response**: We have spelt out acronyms for HCW-Health care worker (For tracked changes see lines, 445, 447, 534, 622, 624, 627, 658 and for clean version see lines 407, 409, 472,533-536,538,568) We have also reduced the use of many acronyms by spelling out the following acronyms, SRH-Sexual and Reproductive Health (For tracked changes see lines, 44,157, 318-320, 322-323, 612-613, 629, 661 and for clean version see lines 43,150,302-304,306, 524-525,541, 571). We have also spelt out the following acronyms, OI-Opportunistic Infections (For tracked changes see line, 635 and for clean version see line 546), STI- (For tracked changes see lines 465 and clean version see line 427), KP-Key populations (For tracked changes see lines, 304-305, 307 and clean version see lines 288-289, 291), SAYWHAT-(For tracked changes see line 309 and clean version see lines 292-293), ZMoHCC-Zimbabwe Ministry of Health and Child Care (For tracked changes see line 634 and clean version see line 545), LEN-Lenacapavir (For tracked changes see line 595 and clean version see line 507), ASRH-Adolescent and Youth Sexual and Reproductive Health Strategy (For tracked version see line 616 and clean version see lines 527-528).

Discussion: Would highlight more in the Methods section how the study involved participatory qualitative methods (In what way is the study participatory?).

**Response**: In the Methods section (lines 186-207 for tracked changes and lines 170-191 for clean version) we describe the approach used: “A participatory approach was used where participants firstly engaged in setting up the ground rules where issues of confidentiality, respecting each other’s views and speaking through the moderator were raised and agreed upon. As a common practice in participatory approaches, PrEP information was provided to the participants in the first instance before the actual discussion commenced [36]. This ensured that participants have a shared understanding of PrEP which led to a more focused, richer discussions. The discussions were also open, taking a less structured approach with the moderators ensuring a free-flowing natural, but focused communication. A participatory approach of having interactive group activities was achieved using role plays that stimulated and encouraged discussions amongst the participants.

Three role plays were conducted in each FGD, creating 3 groups for each role play in each FGD. In each FGD, two to three participants acted a scene depicting conversations AGYW would have with friends or male partners about taking oral PrEP, which was followed by detailed discussion to explore views/understanding of the group [37, 38]. Discussions in these small groups for role plays lead to some shy participants opening up and makings contributions. Appendix A presents the discussion guide with details of scenarios that were acted out. The questions centred around perception of HIV risk, PrEP knowledge and understanding, identifying barriers and facilitators to PrEP uptake and the preferred PrEP service delivery models amongst sexually active AGYW. Detailed discussions were made after each role play to elicit views, identify different views within the group [37, 38]. Participatory approaches enable participants to open up, relax, interact and contribute comfortably and can avoid domination of discussions by a few people [37, 38].”

Discussion: What were some of the facilitators to PrEP uptake? It seemed that most Results related to barriers?

**Response**: We have included some facilitators to PrEP uptake.

“High risk perception was thought to influence one’s decision making in using PrEP as they acknowledged that they were at a risk of getting HIV. See lines 297-299 for tracked changes and lines 281-283 for clean version.

“Good knowledge of PrEP was perceived to facilitate PrEP uptake as AGYW indicated that understanding PrEP’s effectiveness in HIV prevention increased one’s confidence in using it.” See lines 311-313 for tracked changes and lines 295-297 for clean version.

“Most of the AGYW shared that changing the current oral PrEP packaging could be helpful and making it more appealing and improving PrEP use among AGYW. Some suggested using the plastic pill bags that are commonly used for dispensing pills in the country.” See lines 350-353 for tracked changed and lines 320-323 for clean version.

“Participants seemed to be quite positive about the CAB-LA and vaginal ring. These were highly preferred and were cited as potential facilitators to improve PrEP uptake as they offer privacy and convenience, as one does not need to take them daily and has a longer dispensing frequency.” See lines 375-378 for tracked changes and lines 338-341 for clean version.

“Access of PrEP at different entry points at health facilities was viewed to be a potential facilitator to PrEP uptake.” See lines 407-408 for tracked changes and lines 370-371 for clean version.

“Participants suggested having community PrEP campaigns, disseminating correct and accurate PrEP information and combating community PrEP misconceptions.” See lines 497-498 for tracked changes and lines 459-461 for clean version.

Discussion: “used participatory approaches…” Again, it’s not clear how the researchers/Methods were participatory? Please explain more in the Methods.

**Response**: In the Methods section (lines 186-207 for tracked changes and lines 170-191 for clean version) we describe the approach used: “A participatory approach was used where participants firstly engaged in setting up the ground rules where issues of confidentiality, respecting each other’s views and speaking through the moderator were raised and agreed upon. As a common practice in participatory approaches, PrEP information was provided to the participants in the first instance before the actual discussion commenced [36]. This ensured that participants have a shared understanding of PrEP which led to a more focused, richer discussions. The discussions were also open, taking a less structured approach with the moderators ensuring a free-flowing natural, but focused communication. A participatory approach of having interactive group activities was achieved using role plays that stimulated and encouraged discussions amongst the participants.

Three role plays were conducted in each FGD, creating 3 groups for each role play in each FGD. In each FGD, two to three participants acted a scene depicting conversations AGYW would have with friends or male partners about taking oral PrEP, which was followed by detailed discussion to explore views/understanding of the group [37, 38]. Discussions in these small groups for role plays lead to some shy participants opening up and makings contributions. Appendix A presents the discussion guide with details of scenarios that were acted out. The questions centred around perception of HIV risk, PrEP knowledge and understanding, identifying barriers and facilitators to PrEP uptake and the preferred PrEP service delivery models amongst sexually active AGYW. Detailed discussions were made after each role play to elicit views, identify different views within the group [37, 38]. Participatory approaches enable participants to open up, relax, interact and contribute comfortably and can avoid domination of discussions by a few people [37, 38].”

Discussion: A limitation might be the broad participant group? (i.e., as mentioned, included people who had never used PrEP). Please clarify in the Methods if all participants were at high risk of HIV.

**Response:** We indicated this as a limitation, “Another limitation is that a minority of participants had not heard of PrEP, hence their views may have been more hypothetical.” See lines 651-653 for tracked changes and lines 561-564 for clean version.

We clarified in the methods section, as follows: “Purposive sampling was done to ensure representation of sexually active AGYW within the ages of 15 and 24 who are enrolled at universities, those who identify as female sex workers, pregnant and lactating AGYW accessing antenatal care and youth centres services. See lines 149-152 for tracked changes and lines 143-146 for clean version. According to literature these groups of AGYW are perceived to be at a high risk of getting HIV.” See lines 155-156 for tracked changes and lines 149-150 for clean version.

Discussion: Please state/clarify in the Methods the % of participants in the study who had experience using PrEP.

**Response:** In table 1, last column we have stated the percentage of participants who have used PrEP. 19 out of 92 have used PrEP before, with 1 person using PrEP at the time of the FGDs. See Table 1 Characteristics of study participants, last column (History of PrEP use).

Discussion: I think more is needed in this section of what can be done to increase awareness of acceptability of PrEP in Zimbabwe. Are there any global studies that have conducted interventions/programs that have helped to increase the acceptability of PrEP?

**Response:** We have provided the following information on what can be done to increase awareness of acceptability of PrEP in Zimbabwe. “A scoping review by Ekwunife 2022, offers valuable insights that can guide strategies to promote PrEP acceptability [81]. It included interventions designed to create demand, facilitate initiation and continued use of PrEP using PrEP uptake strategies like social marketing. Such strategies can be piloted in Zimbabwe as tapping into social media, a mode of communication that appeals to young people has potential to increase interest in PrEP. See lines 640-644 for tracked changes and lines 550-554 for clean version.

Discussion: What future studies are needed in this area? What's next/would be helpful?

**Response:** We have explained and added this sentence, “Future research is needed on how PrEP interventions can be tailor-made to suit AGYW at high risk of HIV; identifying delivery approaches that resonate with young people while addressing barriers to access.” See lines 663-665 for tracked changes and lines 572-575 for clean version.
